# Synthesis of Sulfoximine Propargyl Carbamates under Improved Conditions for Rhodium Catalyzed Carbamate Transfer to Sulfoxides

**DOI:** 10.1021/acs.joc.2c02083

**Published:** 2022-11-15

**Authors:** Zhenhao Zhong, Julian Chesti, Alan Armstrong, James A. Bull

**Affiliations:** Department of Chemistry, Imperial College London, Molecular Sciences Research Hub, White City Campus, Wood Lane, London W12 0BZ, U.K.

## Abstract

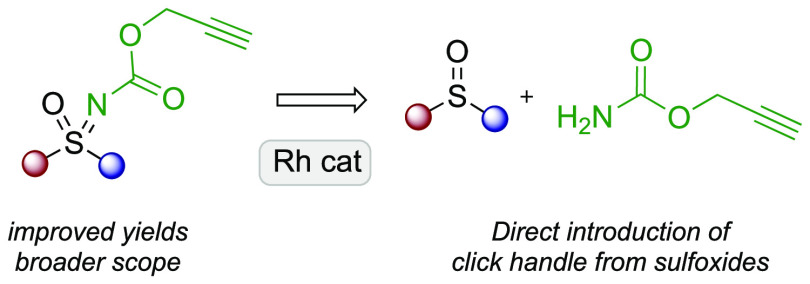

Sulfoximines provide aza-analogues of sulfones, with potentially improved properties for medicinal chemistry. The sulfoximine nitrogen also provides an additional vector for the inclusion of other functionality. Here, we report improved conditions for rhodium catalyzed synthesis of sulfoximine (and sulfilimine) carbamates, especially for previously low-yielding carbamates containing π-functionality. Notably we report the preparation of propargyl sulfoximine carbamates to provide an alkyne as a potential click handle. Using Rh_2_(esp)_2_ as catalyst and a DOE optimization approach provided considerably increased yields.

Sulfoximines, the mono aza analogue of sulfones, have become increasingly important motifs in medicinal chemistry (Figure 1a).^1^ The incorporation of sulfoximines in place of sulfonamides or sulfones has afforded attractive biologically active compounds that have entered clinical trials for example as anticancer agents, including roniciclib^2^ and BAY1143572^3^ (Bayer), and ceralasertib (AstraZeneca).^4,5^ Sulfoximines are chemically and configurationally stable^6^ and have been found to exhibit improved physicochemical and metabolic properties.^7^ They also provide an additional vector which is valuable for the attachment of a range of functionality through the sulfoximine nitrogen.^8,9^

**Figure 1:**
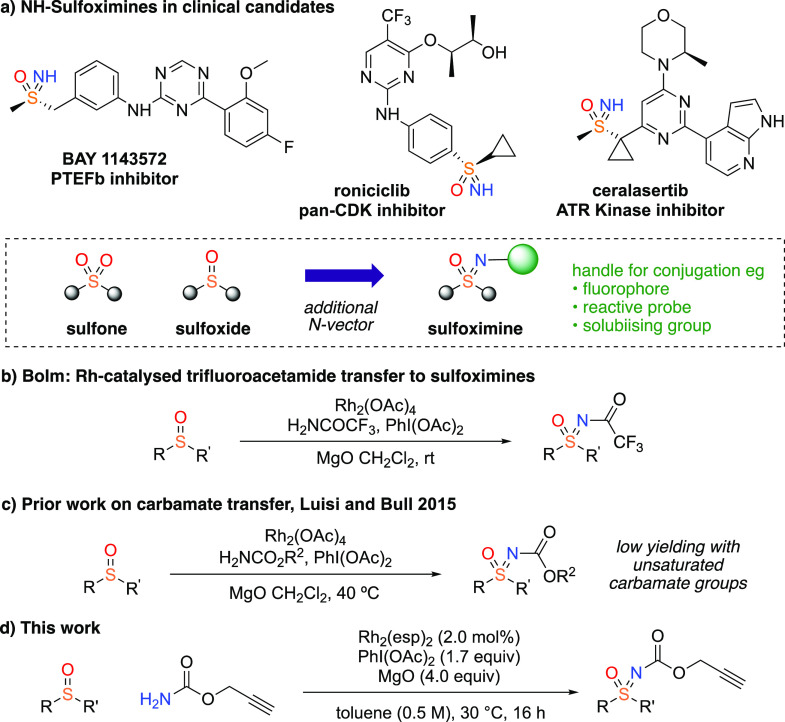
Chiral sulfoximines as clinical candidates and rhodium-catalyzed sulfoximine synthesis.

Methods for the synthesis of sulfoximines have expanded greatly in recent years.^8,10^ Bolm has pioneered numerous methods for the metal-catalyzed transfer of N-functionality to sulfoxides,^11,12^ including notably powerful methods for Rh-catalyzed transfer of trifluoroacetamide (Figure 1b).^11a,13^ Bull and Luisi developed conditions for the Rh-catalyzed transfer of carbamates to sulfoxides including Boc and Cbz carbamates.^14^ Recent developments include metal-free methods for NH-sulfoximine synthesis from sulfoxides and sulfides using hypervalent iodine(III) reagents and ammonium carbamate as the N-source.^15−17^ Willis has developed sulfinylamine (RNSO) reagents, suitable for reaction with nucleophiles to form sulfoximines.^18^ We have recently developed sulfonimidoyl fluorides for the preparation of enantioenriched sulfoximines with Grignard reagents.^19,20^ The formation of sulfilimines by N-transfer to sulfides has seen similar developments including enantioselective N-transfer,^21^ and these can be oxidized to sulfoximines.^10a,12^

Aiming to expand access to sulfoximine derivatives bearing pendant N-functionality we revisited our previous work on carbamate N-transfer to sulfoxides (Figure 1c). The previous conditions were effective for alkyl carbamates (R^2^ = Me, *t*Bu), but much lower yields were obtained with unsaturated carbamate substituents. Allyl carbamate for example gave a 40% yield with methyl *p*-tolyl sulfoxide.^14^ In particular, we envisaged that the direct incorporation of an alkyne as a click handle at the same time as constructing the sulfoximine motif would provide a useful process. Given the prevalence of sulfoxides and sulfones in biologically active compounds,^22^ as well as sulfoximines themselves,^1^ we envisaged that the additional vector afforded by a sulfoximine derived from these other S(IV) and S(VI) derivatives could be of value in labeling and provide a handle for further conjugation, for example attachment of a fluorophore or other derivatization of the alkyne. On this basis we investigated the use of propargyl carbamate as a substrate for the preparation of sulfoximines to install an alkyne motif suitable for click chemistry (Figure 1d).

Here we report improved conditions for the carbamate transfer to sulfoxides for the preparation of sulfoximines. In particular this allows the preparation of propargyl carbamate derivatives. Notably improved yields for the preparation of other carbamate derivatives containing π-electrons were also achieved under the modified conditions broadening the potential to form sulfoximine carbamates and sulfilimine carbamates more generally without requiring preformed activated carbamate reagents.

We initially investigated the transfer of 2-propynyl carbamate to methyl phenylsulfoxide **1a**. Applying directly our previously developed reaction conditions using Rh_2_(OAc)_4_ in CH_2_Cl_2_ afforded the *N*-propargylcarbamate sulfoximine with a low 24% yield along with an oxidized sulfone side product (Table 1, entry 1). This was in keeping with our previously reported observations that π-functionality in the carbamate was detrimental, and prompted a program of optimization on this reaction to increase yield. Increasing the reaction concentration and catalyst loading gave a slight improvement, but the undesired sulfone continued to be generated in significant quantities, which was also difficult to separate from the desired sulfoximine product (entry 3). Switching the solvent from CH_2_Cl_2_ to toluene provided a further improved yield of **2a** and reduced the formation of side product **3a** (entry 4), while also providing a more attractive solvent for development. Changing the rhodium catalyst to Rh_2_(O_2_CCF_3_)_4_ was unsuccessful, whereas using Rh_2_(Oct)_4_ afforded higher yields with Rh_2_(esp)_2_ achieving an 87% yield (entries 5–7).^23^

**Table 1:**
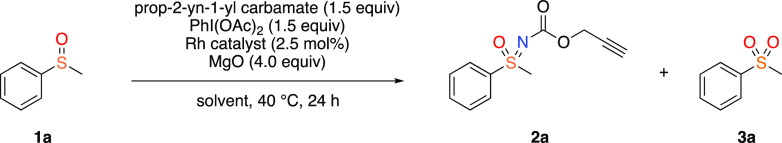
Selected Optimization for Rhodium Catalyzed Propargylic Carbamate Transfer to Sulfoxide **1a**

			Yield (%)^b^		
entry^a^	Rh catalyst	solvent (concn)	**2a**	**3a**	**1a**
1	Rh_2_(OAc)_4_	CH_2_Cl_2_ (0.3 M)	24	12	61
2	Rh_2_(OAc)_4_	CH_2_Cl_2_ (0.5 M)	30	15	57
3	Rh_2_(OAc)_4_^c^	CH_2_Cl_2_ (0.5 M)	43	22	27
4	Rh_2_(OAc)_4_	toluene (0.5 M)	53	4	38
5	Rh_2_(O_2_CCF_3_)_4_	toluene (0.5 M)	trace	7	93
**6**	Rh_2_(Oct)_4_	toluene (0.5 M)	73	5	12
**7**	**Rh**_**2**_**(esp)**_**2**_	**toluene (0.5 M)**	**87**	**4**	**6**

a Reactions on 0.3 mmol scale.

b Yields determined by the analysis of ^1^H NMR using 1,3,5-trimethoxybenzene as an internal standard.

c 5 mol % Rh_2_(OAc)_4_ used.

Next a design-of-experiments (DoE) optimization was undertaken to further optimize and improve the reaction conditions accounting for the interplay of conditions. The equivalents of the oxidant and carbamate, the catalyst loading and the reaction temperature were identified as major factors to influence yield. Identical equivalents of oxidant and carbamate were used to group these as a single variable so as to reduce the number of variables in the DOE study and improve model accuracy. Other factors such as the concentration (0.5 M) and the reaction time (24 h) were set to fixed values. Additionally, the DoE custom design examined second-order interactions of these parameters. The correlation between the predicted yield and the parameters was visualized by a three-dimensional response surface of the predicted yield against two major factors (the equivalents of oxidant/carbamate and the catalyst loading), and the dome-shaped surface showed a saddle point at 87% (Figure 2). These conditions gave excellent *in situ* (90%) and isolated yields (85%) of **2a** which were well correlated with the prediction. No difference was observed by comparing the *in situ* yield after 16 and 24 h, which indicated that the reaction time could be reduced to 16 h for efficiency purposes. Overall, the optimal yield was achieved by increasing the equivalents of the oxidant and carbamate and importantly reducing the catalyst loading, as well as the reaction time and temperature.

**Figure 2:**
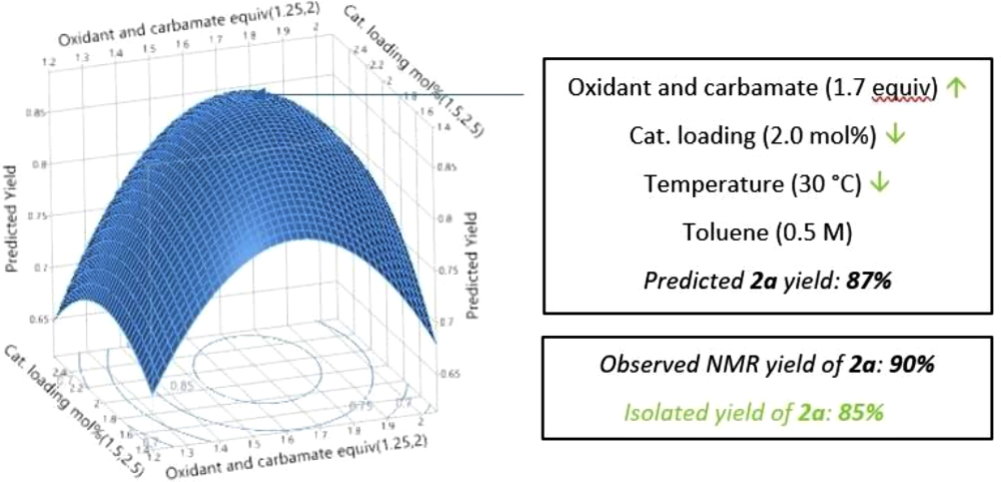
Plot of predicted yield of *N*-propargylic sulfoximine **2a** vs the equivalents of oxidant/carbamate and catalyst loading visualized at fixed concentration (0.5 M) and temperature (30 °C). DoE analysis carried out with JMP Pro 14 with a custom design screen.

With the optimized reaction conditions the scope of the reaction was then investigated on a slightly larger 0.5 mmol scale (Scheme 1). Good to excellent yields were obtained for *para*- and *meta*-substituted arylmethylsulfoxide derivatives bearing electron-donating and electron-withdrawing substituents (**2a**–**2g**), including halogen and ketone functionality. Enantioenriched (*S*)-**2b** was obtained with excellent *ee*, showing the complete retention of stereochemical information with enantioenriched substrates. Scaling the reaction to 3 mmol scale did not affect the yield significantly (**2e**). It was noticeable that low yields were observed for *ortho*-substituted examples **2h** and **2i**; the corresponding *ortho*-chloro-derivative (not shown) gave only trace amounts of the sulfoximine product and recovered starting material highlighting the steric and electronic demands on the nucleophilicity at sulfur. In these lower yielding examples, the mass balance was recovered starting material. Good yields were witnessed for alkyl-aryl substrates and their derivatives **2j**–**m**, including the cyclopropyl derivative (**2n**), a structural feature that has been observed in recent clinical candidates. The reduced yield with isopropyl (**2o**) was again indicative of steric demands which was also apparent with diphenyl and dibenzyl substrates (**2p** and **2q**). Other dialkyl substrates (**2r** and **2s**) were investigated, and each was successful including *t*Bu derivative **2s**. Cyclic sulfoxide **2t** gave an excellent yield. A moderate yield was achieved with the 2-pyridyl substrate (**2u**). This method also showed tolerance of other interesting functional groups, including terminal alcohol (**2v**) and terminal alkene (**2w**) where vinyl sulfoximine could be applied as a possible probe structure in chemical biology.^17^ Using protected methionine sulfoxide gave the propargylic carbamate derivative of methionine sulfoximine (MSO, **2x**), MSO itself being an inhibitor for the biosynthesis of glutathione.

**Scheme 1:**
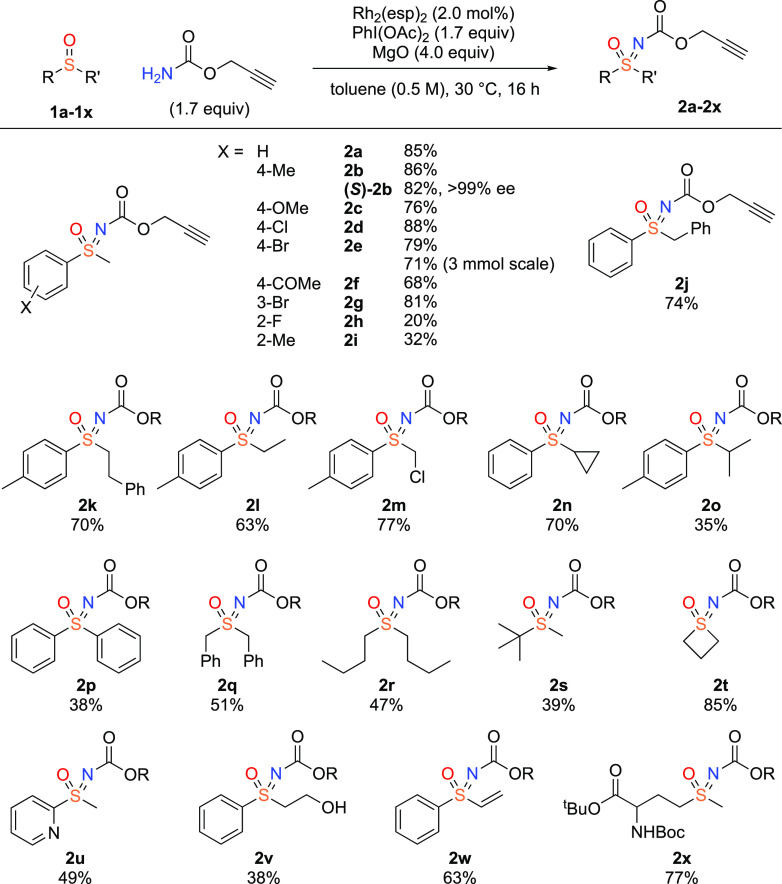
Substrate Scope of Propargylic Carbamate Transfer Reactions on 0.5 mmol scale. All yields correspond to isolated products. R = CH_2_CCH.

To make a direct comparison with our previous conditions (see Figure 1),^14^ we compared these across a range of carbamate types (Scheme 2). For carbamates with alkyl substituents, such as *tert*-butyl and methyl carbamate, both methods showed excellent yields on methyl *p*-tolylsulfoxide (98–99%, **4**, **5**). In our previous report, the yields dropped significantly to 60% and 54% for benzyl (Cbz) and phenyl carbamates.^14^ However, the yields remained at 95% for benzyl carbamate **6** and 90% for phenyl carbamate **7** using the new conditions. In contrast to the disappointing results obtained with allyl and alkynyl carbamates under the previous conditions, allyl **8** and TMS protected propargyl **9** were obtained in 82% and 93% yields.

**Scheme 2:**
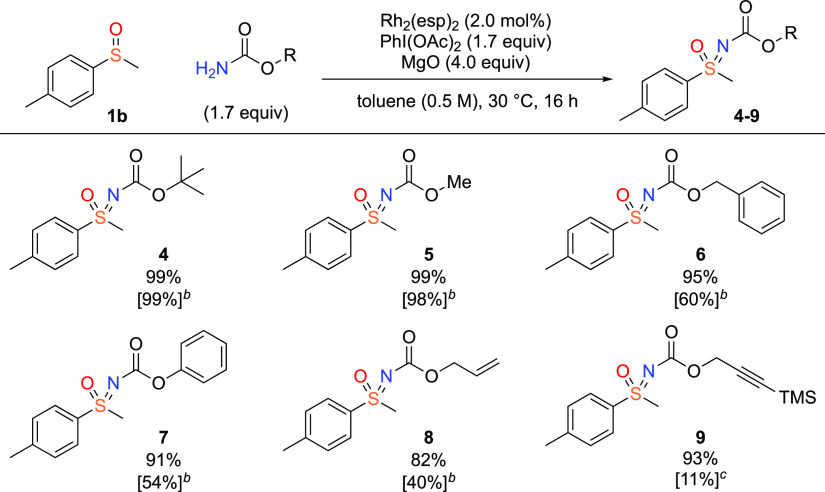
Effect of Variation of the Carbamate Reactions on 0.5 mmol scale. All yields correspond to isolated products. Yield in square brackets corresponds to the yields under previously reported conditions. Result as reported in ref (14). Yield using conditions reported in ref (14).

The developed conditions for carbamate transfer were also applied to sulfide substrates to afford sulfilimines (Scheme 3). Previous examples of sulfilimine carbamates have all applied preformed activated carbamates, bearing N–O or N–X groups.^10,21^ Applying our propargyl carbamate transfer to methyl *p*-tolyl sulfides gave sulfilimine **12** with a 32% yield, where direct oxidation to the corresponding sulfoxide represented the major side product. Moderate to good yields were observed when transferring *tert*-butyl carbamate to sulfides, affording the products **13**, **14**, and **15** with different aryl-substituents. Interestingly, switching to Cbz carbamate gave sulfilimine **16** in excellent yield. Additionally, the transfer of *tert*-butyl carbamate to a sulfenamide was successful, affording the unusual sulfinamidine product **17** with a 38% yield.^24^

**Scheme 3:**
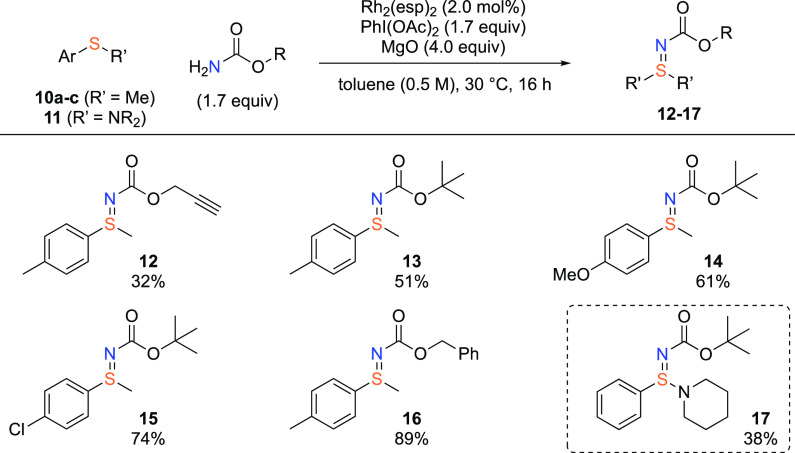
Sulfilimine Carbamate Synthesis

Finally, the potential utility of propargyl carbamate-sulfoximine was demonstrated in CuAAC click reactions with alkyl azides (Scheme 4). Using **2a** and benzylazide cleanly formed triazole **18**. Similarly, biotin azide was suitable to prepare the corresponding triazole (**19**) as may be valuable in chemical biology.

**Scheme 4:**
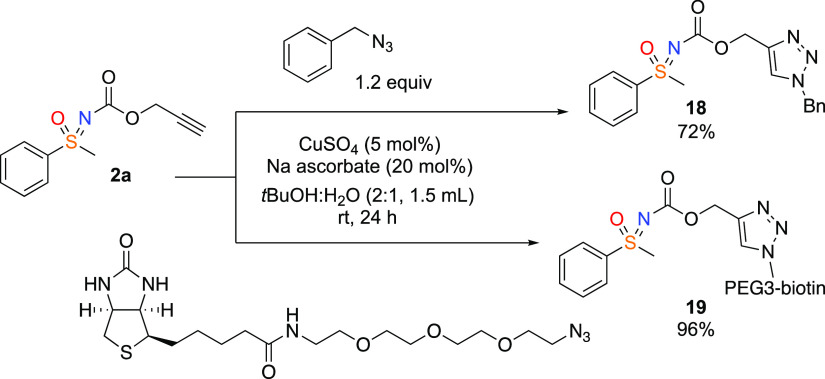
Cycloaddition Reactions of Sulfoximine Containing Alkynes with Azides

In conclusion, improved conditions for the preparation of sulfoximine carbamates are reported where the use of more reactive Rh_2_(esp)_2_ catalyst and toluene solvent gave improved tolerance of carbamates with unsaturated functionality. This method may further enhance the possibilities for the use of sulfoximines, to exploit that additional vector and directly install a broader range of carbamates with improved yields. The cycloaddition chemistry from the alkynes may provide an alternative way to incorporate small sulfoximine motifs, or allow labeling via other sulfur derivatives. It also provides the first example of the formation of sulfilimine carbamates without the requirement for a preactivated nitrogen source.

## Experimental Section

### General Experimental Considerations

All nonaqueous reactions were run under an inert atmosphere (argon) with flame-dried or oven-dried glassware using standard techniques. Anhydrous solvents were obtained by filtration through drying columns (CH_2_Cl_2_) or used directly from commercial sources (^*t*^BuOH, EtOAc, toluene) without drying. Rh_2_(esp)_2_ (96%) was purchased from Sigma-Aldrich and PhI(OAc)_2_ (98%) was purchased from Fluorochem, and used directly without further treatment. Flash column chromatography was performed using 230–400 mesh silica with the indicated solvent system according to standard techniques. Analytical thin-layer chromatography (TLC) was performed on precoated, glassbacked silica gel plates. Visualization of the developed chromatogram was performed by UV absorbance (254 nm) or aqueous potassium permanganate stains. Infrared spectra (ν_max_, FTIR ATR) were recorded in reciprocal centimeters (cm^–1^). Nuclear magnetic resonance spectra were recorded on 400 MHz spectrometers. Chemical shifts for ^1^H NMR spectra are recorded in parts per million from tetramethylsilane with the residual protic solvent resonance as the internal standard (chloroform: δ = 7.27 ppm, DMSO-*d*_*6*_: δ = 2.50 ppm, MeOD-*d*_*4*_: δ = 3.31 ppm). Data are reported as follows: chemical shift [multiplicity (s = singlet, d = doublet, t = triplet, quartet = q, pentet = p, m = multiplet and br = broad), coupling constant in Hz, integration, assignment]. ^13^C NMR spectra were recorded with complete proton decoupling. Chemical shifts are reported in parts per million from tetramethylsilane with the solvent resonance as the internal standard (^13^CDCl_3_: δ = 77.0 ppm, (^13^CD_3_)_2_SO: δ = 39.5 ppm, ^13^CD_3_OD: δ = 49.0 ppm). *J* values are reported in Hz. Assignments of ^1^H/^13^C spectra were made by the analysis of δ/*J* values, and COSY, HSQC, and HMBC experiments as appropriate. Melting points are uncorrected. Heating blocks were used for reactions above room temperature.

Reagents: Commercial reagents were used as supplied or purified by standard techniques where necessary. Commercially available sulfoxides (**1a**, **1b**, **(*****S*****)**-**1b**, **1n**, **1p**–**s**, and **1w**), carbamates (Boc, Cbz, CO_2_Me, and CO_2_Ph), and sulfides (**10a**–**c**) were used as supplied, and others were prepared from the corresponding commercially available sulfides or thiols as previously reported.^25^ Sulfenamide **11** was prepared as previously reported.^26^ Benzyl azide was prepared as previously reported.^27^ Azide-PEG3-biotin conjugate was purchased from Sigma-Aldrich.

### Carbamate Preparation

#### Prop-2-yn-1-yl Carbamate^28^

CF_3_CO_2_H (7.7 mL, 100 mmol, 2.0 equiv) was added dropwise to a stirred solution of propargyl alcohol (2.90 mL, 50 mmol, 1.0 equiv) and NaOCN (6.8 g, 100 mmol, 2.0 equiv) in anhydrous Et_2_O (100 mL) at 30 °C. The reaction mixture was stirred at 30 °C overnight. The resulting mixture was diluted with Et_2_O (100 mL) and filtered. The filtrate was concentrated under reduced pressure. Purification by flash column chromatography (SiO_2_, 30% Et_2_O in pentane) afforded prop-2-yn-1-yl carbamate as a white solid (2.13 g, 43%). R_*f*_ = 0.12 (30% Et_2_O in pentane); mp = 48–51 °C; IR (film)/cm^–1^ 3425, 3307, 3280, 3211, 2952, 1675, 1647, 1607, 1405, 1328, 1062, 865, 702, 634, 563; ^1^H NMR (400 MHz, CDCl_3_) δ 4.93 (br s, 2H, NH_2_), 4.69 (d, *J* = 2.5 Hz, 2H, CH_2_), 2.50 (t, *J* = 2.5 Hz, 1H, CCH); ^13^C NMR (101 MHz, CDCl_3_) δ 155.8 (C=O), 77.9 (*C*CH), 74.8 (C*C*H), 52.7 (CH_2_). Analytical data (NMR and IR) in agreement with those reported in the literature.^29^

#### 3-(Trimethylsilyl)prop-2-yn-1-yl Carbamate

CF_3_CO_2_H (3.1 mL, 40.4 mmol, 2.0 equiv) was added dropwise to a stirred solution of 3-trimethylsilyl-2-propyn-1-ol (3.0 mL, 20.2 mmol, 1.0 equiv) and NaOCN (2.63 g, 40.4 mmol, 2.0 equiv) in anhydrous Et_2_O (40 mL) at 30 °C. The reaction mixture was stirred at 30 °C overnight. The resulting mixture was diluted with Et_2_O (40 mL) and filtered. The filtrate was concentrated under reduced pressure. Purification by flash column chromatography (SiO_2_, 30% EtOAc in pentane) afforded 3-(trimethylsilyl)prop-2-yn-1-yl carbamate as a white solid (1.86 g, 54%). R_*f*_ = 0.33 (30% EtOAc in pentane); mp = 53–57 °C; IR (film)/cm^–1^ 3451, 3343, 3292, 3185, 2964, 2181, 1705 (C=O), 1606, 1388, 1320, 1067, 1011, 838, 764, 703; ^1^H NMR (400 MHz, CDCl_3_) δ 4.84 (br s, 2H, NH_2_), 4.69 (s, 2H, CH_2_), 0.19 (s, 9H, (CH_3_)_3_); ^13^C NMR (101 MHz, CDCl_3_) δ 155.8 (C=O), 99.2 (*C*CSi), 92.1 (C*C*Si), 53.5 (*C*(CH_3_)_3_), −0.3 (C(*C*H_3_)_3_); HRMS (ESI-TOF) *m*/*z*: Calcd. for C_7_H_14_NO_2_Si [M + H]^+^: 172.0788, found: 172.0789.

### General Procedure for Rhodium-Catalyzed Carbamate Transfer to Sulfoxides and Sulfides (Schemes 1–3)

PhI(OAc)_2_ (274 mg, 0.85 mmol, 1.7 equiv) was added to a suspension of the sulfoxide or sulfide (0.5 mmol, 1.0 equiv), carbamate (0.85 mmol, 1.7 equiv), MgO (81 mg, 2.0 mmol, 4.0 equiv), and Rh_2_(esp)_2_ (7.6 mg, 2.0 mol %) in toluene (1.0 mL) at rt. The resulting mixture was heated to 30 °C and stirred for 16 h. The reaction mixture was diluted with CH_2_Cl_2_ (1 mL), filtered through Celite, and concentrated under reduced pressure. The resulting residue was purified by flash column chromatography (SiO_2_) to afford the corresponding sulfoximine or sulfilimine carbamate.

#### Prop-2-yn-1-yl (Methyl(oxo)(phenyl)-λ^6^-sulfaneylidene)carbamate (**2a**)

Prepared using the General Procedure using sulfoxide **1a** (69.6 mg, 0.50 mmol) and prop-2-yn-1-yl carbamate (84.2 mg, 0.85 mmol, 1.7 equiv). Purification by flash column chromatography (SiO_2_, 40% EtOAc in hexane) afforded sulfoximine propargyl carbamate **2a** (100.2 mg, 85%) as a pale-yellow gum. R_*f*_ = 0.17 (40% EtOAc in hexane); IR (film)/cm^–1^ 3261, 3014, 2925, 2120, 1668 (C=O), 1444, 1368, 1223, 1084, 977, 867, 783, 742, 683, 559, 504; ^1^H NMR (400 MHz, CDCl_3_) δ 8.02–7.99 (m, 2H, 2 × Ar–H), 7.71–7.69 (m, 1H, Ar–H), 7.64–7.60 (m, 2H, 2 × Ar–H), 4.70–4.65 (m, 1H, OC*H*H), 4.65–4.60 (m, 1H, OCH*H*), 3.34 (s, 3H, SCH_3_), 2.43 (t, *J* = 2.5 Hz, 1H, CCH); ^13^C{^1^H} NMR (101 MHz, CDCl_3_) δ 158.0 (C=O), 138.0 (Ar–C_q_), 134.1 (Ar–C), 129.7 (2 × Ar–C), 127.4 (2 × Ar–C), 77.9 (*C*CH), 74.6 (C*C*H), 53.4 (CH_2_), 44.5 (SCH_3_); HRMS (ESI-TOF) *m*/*z*: Calcd. for C_11_H_12_NO_3_S [M + H]^+^: 238.0538, found: 238.0533.

#### Prop-2-yn-1-yl (Methyl(oxo)(p-tolyl)-λ^6^-sulfaneylidene)carbamate (**2b**)

Prepared using the General Procedure using sulfoxide **1b** (76.6 mg, 0.50 mmol) and prop-2-yn-1-yl carbamate (84.2 mg, 0.85 mmol, 1.7 equiv). Purification by flash column chromatography (SiO_2_, 40% EtOAc in hexane) afforded sulfoximine propargyl carbamate **2b** (108.1 mg, 86%) as a pale-yellow gum. R_*f*_ = 0.22 (40% EtOAc in hexane); IR (film)/cm^–1^ 3260, 3016, 2924, 2120, 1668 (C=O), 1593, 1368, 1224, 1086, 978, 866, 812, 631, 512, 493; ^1^H NMR (400 MHz, CDCl_3_) δ 7.88 (d, *J* = 8.4 Hz, 2H, 2 × Ar–H), 7.42–7.40 (m, 2H, 2 × Ar–H), 4.70–4.66 (m, 1H, OC*H*H), 4.65–4.61 (m, 1H, OCH*H*), 3.32 (s, 3H, SCH_3_), 2.47 (s, 3H, Ar–CH_3_), 2.43 (t, *J* = 2.5 Hz, 1H, CCH); ^13^C{^1^H} NMR (101 MHz, CDCl_3_) δ 158.1 (C=O), 145.3 (Ar–C_q_), 134.8 (Ar–C_q_), 130.4 (2 × Ar–C), 127.4 (2 × Ar–C), 78.0 (*C*CH), 74.6 (C*C*H), 53.4 (CH_2_), 44.6 (SCH_3_), 21.6 (Ar–CH_3_); HRMS (ESI-TOF) *m*/*z*: Calcd. for C_12_H_14_NO_3_S [M + H]^+^: 252.0694, found: 252.0682.

#### Prop-2-yn-1-yl (S)-(Methyl(oxo)(p-tolyl)-λ^6^-sulfaneylidene)carbamate (**(S)-2b**)

Prepared using General Procedure using sulfoxide **(*****S*****)-1b** (77.2 mg, 0.50 mmol) and prop-2-yn-1-yl carbamate (84.2 mg, 0.85 mmol, 1.7 equiv). Purification by flash column chromatography (SiO_2_, 40% EtOAc in hexane) afforded sulfoximine propargyl carbamate **(*****S*****)-2b** (102.5 mg, 82%, > 99% ee) as a pale-yellow gum. R_*f*_ = 0.22 (40% EtOAc in hexane); Spectroscopic data same as those for **2b** above. [α]^21^_D_ = +52 (*c* 1.0, CHCl_3_). HPLC conditions: Chiralpak IB column, 90:10 *n*-hexane:*i*PrOH, flow rate: 1 mL min^–1^, 35 °C, UV detection wavelength: 250 nm, (**(***S***)-2b**) retention time: 38 min. (**(*****rac*****)-2b**) retention times: 38 and 42 min.

#### Prop-2-yn-1-yl ((4-Methoxyphenyl)(methyl)(oxo)-λ^6^-sulfaneylidene)carbamate (**2c**)

Prepared using General Procedure using sulfoxide **1c** (84.7 mg, 0.50 mmol) and prop-2-yn-1-yl carbamate (84.2 mg, 0.85 mmol, 1.7 equiv). Purification by flash column chromatography (SiO_2_, 30% EtOAc in hexane) afforded sulfoximine propargyl carbamate **2c** (101.9 mg, 76%) as a white solid. R_*f*_ = 0.08 (30% EtOAc in hexane); mp = 84–85 °C; IR (film)/cm^–1^ 3265, 3019, 2930, 2848, 2125, 1670 (C=O), 1595, 1498, 1312, 1238, 1088, 984, 867; ^1^H NMR (400 MHz, CDCl_3_) δ 7.93–7.90 (m, 2H, 2 × Ar–H), 7.08–7.04 (m, 2H, 2 × Ar–H), 4.70–4.66 (m, 1H, OC*H*H), 4.65–4.60 (m, 1H, OCH*H*), 3.90 (s, 3H, OCH_3_), 3.32 (s, 3H, SCH_3_), 2.43 (t, *J* = 2.5 Hz, 1H, CCH); ^13^C{^1^H} NMR (101 MHz, CDCl_3_) δ 164.1 (Ar–C_q_), 158.1 (C=O), 129.6 (2 × Ar–C), 128.8(Ar–C_q_), 115.0 (2 × Ar–C), 78.0 (*C*CH), 74.6 (C*C*H), 55.8 (OCH_3_), 53.3 (CH_2_), 44.9 (SCH_3_); HRMS (ESI-TOF) *m*/*z*: Calcd. for C_12_H_14_NO_4_S [M + H]^+^: 268.0644, found: 268.0640.

#### Prop-2-yn-1-yl ((4-Chlorophenyl)(methyl)(oxo)-λ^6^-sulfaneylidene)carbamate (**2d**)

Prepared using General Procedure using sulfoxide **1d** (87.2 mg, 0.50 mmol) and prop-2-yn-1-yl carbamate (84.2 mg, 0.85 mmol, 1.7 equiv). Purification by flash column chromatography (SiO_2_, 40% Et_2_O in pentane) afforded sulfoximine propargyl carbamate **2d** (118.9 mg, 88%) as a white gum. R_*f*_ = 0.10 (40% Et_2_O in pentane); IR (film)/cm^–1^ 3283, 3083, 3016, 2120, 1672 (C=O), 1573, 1366, 1217, 1081, 977, 866, 783, 684, 462; ^1^H NMR (400 MHz, CDCl_3_) δ 7.95–7.93 (m, 2H, 2 × Ar–H), 7.61–7.58 (m, 2H, 2 × Ar–H), 4.70–4.66 (m, 1H, OC*H*H), 4.65–4.60 (m, 1H, OCH*H*), 3.34 (s, 3H, SCH_3_), 2.44 (t, *J* = 2.5 Hz, 1H, CCH); ^13^C{^1^H} NMR (101 MHz, CDCl_3_) δ 157.8 (C=O), 141.1 (Ar–C_q_), 136.4 (Ar–C_q_), 130.1 (2 × Ar–C), 128.9 (2 × Ar–C), 77.8 (*C*CH), 74.8 (C*C*H), 53.5 (CH_2_), 44.5 (SCH_3_); HRMS (ESI-TOF) *m*/*z*: Calcd. for C_11_H_11_NO_3_S^35^Cl [M + H]^+^: 272.0148, found: 272.0151.

#### Prop-2-yn-1-yl ((4-Bromophenyl)(methyl)(oxo)-λ^6^-sulfaneylidene)carbamate (**2e**)

Prepared using General Procedure using sulfoxide **1e** (110.4 mg, 0.50 mmol) and prop-2-yn-1-yl carbamate (84.2 mg, 0.85 mmol, 1.7 equiv). Purification by flash column chromatography (SiO_2_, 40% Et_2_O in pentane) afforded sulfoximine propargyl carbamate **2e** (125.4 mg, 79%) as a colorless gum. R_*f*_ = 0.12 (40% Et_2_O in pentane); IR (film)/cm^–1^ 3263, 3083, 3012, 2923, 2120, 1668 (C=O), 1567, 1367, 1224, 1085, 1065, 978, 867, 780, 676, 565, 508; ^1^H NMR (400 MHz, CDCl_3_) δ 7.85–7.82 (m, 2H, 2 × Ar–H), 7.75–7.72 (m, 2H, 2 × Ar–H), 4.65–4.60 (m, 1H, OC*H*H), 4.60–4.55 (m, 1H, OCH*H*), 3.31 (s, 3H, SCH_3_), 2.42 (t, *J* = 2.5 Hz, 1H, CCH); ^13^C{^1^H} NMR (101 MHz, CDCl_3_) δ 157.7 (C=O), 136.8 (Ar–C_q_), 132.9 (2 × Ar–C), 129.4 (Ar–C_q_), 128.8 (2 × Ar–C), 77.7 (*C*CH), 74.7 (C*C*H), 53.4 (CH_2_), 44.3 (SCH_3_); HRMS (ESI-TOF) *m*/*z*: Calcd. for C_11_H_11_NO_3_S^79^Br [M + H]^+^: 315.9643, found: 315.9650. Three mmol scale synthesis of *2e*: PhI(OAc)_2_ (1.64 g, 5.1 mmol, 1.7 equiv) was added in three-portions over 5 min to a stirring suspension of sulfoxide **1e** (657 mg, 3.0 mmol), prop-2-yn-1-yl carbamate (505 mg, 5.1 mmol, 1.7 equiv), MgO (484 mg, 12.0 mmol, 4.0 equiv) and Rh_2_(esp)_2_ (45.5 mg, 0.06 mmol, 2.0 mol %) in toluene (6 mL) at rt. The resulting mixture was heated to 30 °C and stirred for 16 h. The reaction mixture was diluted with CH_2_Cl_2_ (5 mL), filtered through Celite and concentrated under reduced pressure. Purification by flash column chromatography (SiO_2_, 40% Et_2_O in pentane) afforded **2e** as a colorless gum (669 mg, 71%).

#### Prop-2-yn-1-yl ((4-Acetylphenyl)(methyl)(oxo)-λ^6^-sulfaneylidene)carbamate (**2f**)

Prepared using General Procedure using sulfoxide **1f** (90.6 mg, 0.50 mmol) and prop-2-yn-1-yl carbamate (84.2 mg, 0.85 mmol, 1.7 equiv). Purification by flash column chromatography (SiO_2_, 40% EtOAc in hexane) afforded sulfoximine propargyl carbamate **2f** (94.6 mg, 68%) as a colorless gum. R_*f*_ = 0.10 (40% EtOAc in hexane); IR (film)/cm^–1^ 3272, 3032, 2926, 1682 (C=O), 1663 (C=O), 1367, 1251, 1229, 968, 920, 863, 789, 621, 500; ^1^H NMR (400 MHz, CDCl_3_) δ 8.17–8.15 (m, 2H, 2 × Ar–H), 8.12–8.10 (m, 2H, 2 × Ar–H), 4.69–4.64 (m, 1H, OC*H*H), 4.64–4.60 (m, 1H, OCH*H*), 3.35 (s, 3H, SCH_3_), 2.68 (s, 3H, COCH_3_), 2.43 (t, *J* = 2.5 Hz, 1H, CCH); ^13^C{^1^H} NMR (101 MHz, CDCl_3_) δ 196.4 (C=O), 157.8 (C=O), 141.9 (Ar–C_q_), 141.1 (Ar–C_q_), 129.4 (2 × Ar–C), 127.9 (2 × Ar–C), 77.7 (*C*CH), 74.8 (C*C*H), 53.6 (CH_2_), 44.2 (SCH_3_), 26.9 (CO*C*H_3_); HRMS (ESI-TOF) *m*/*z*: Calcd. for C_13_H_14_NO_4_S [M + H]^+^: 280.0644, found: 280.0638.

#### Prop-2-yn-1-yl ((3-Bromophenyl)(methyl)(oxo)-λ^6^-sulfaneylidene)carbamate (**2g**)

Prepared using General Procedure using sulfoxide **1g** (108.5 mg, 0.50 mmol) and prop-2-yn-1-yl carbamate (84.2 mg, 0.85 mmol, 1.7 equiv). Purification by flash column chromatography (SiO_2_, 50% Et_2_O in hexane) afforded sulfoximine propargyl carbamate **2g** (127.6 mg, 81%) as a colorless gum. R_*f*_ = 0.13 (50% Et_2_O in hexane); IR (film)/cm^–1^ 3273, 3079, 3019, 2930, 2125, 1670 (C=O), 1365, 1230, 1103, 977, 872, 775, 671; ^1^H NMR (400 MHz, CDCl_3_) δ 8.12–8.09 (m, 1H, Ar–H), 7.91–7.88 (m, 1H, Ar–H), 7.80–7.77 (m, 1H, Ar–H), 7.49–7.45 (m, 1H, Ar–H), 4.65–4.61 (m, 1H, OC*H*H), 4.60–4.55 (m, 1H, OCH*H*), 3.32 (s, 3H, SCH_3_), 2.42 (t, *J* = 2.5 Hz, 1H, CCH); ^13^C{^1^H} NMR (101 MHz, CDCl_3_) δ 157.6 (C=O), 139.7 (Ar–C_q_), 137.1 (Ar–C), 131.1 (Ar–C), 130.2 (Ar–C), 125.8 (Ar–C), 123.5 (Ar–C_q_), 77.7 (*C*CH), 74.8 (C*C*H), 53.4 (CH_2_), 44.3 (SCH_3_); HRMS (ESI-TOF) *m*/*z*: Calcd. for C_11_H_11_NO_3_S^79^Br [M + H]^+^: 315.9643, found: 315.9649.

#### Prop-2-yn-1-yl ((2-Fluorophenyl)(methyl)(oxo)-λ^6^-sulfaneylidene)carbamate (**2h**)

Prepared using General Procedure using sulfoxide **1h** (80.0 mg, 0.51 mmol) and prop-2-yn-1-yl carbamate (84.2 mg, 0.85 mmol, 1.7 equiv). Purification by flash column chromatography (SiO_2_, 40% EtOAc in hexane) afforded sulfoximine propargyl carbamate **2h** (25.4 mg, 20%) as a colorless gum. R_*f*_ = 0.22 (40% EtOAc in hexane); IR (film)/cm^–1^ 3273, 3019, 2937, 2125, 1670 (C=O), 1476, 1230, 1126, 1074, 977, 865, 760; ^1^H NMR (400 MHz, CDCl_3_) δ 8.09 (ddd, *J* = 8.0, 7.1, 1.8 Hz, 1H, Ar–H), 7.71 (dddd, *J* = 8.3, 7.5, 5.0, 1.8 Hz, 1H, Ar–H), 7.46–7.38 (m, 1H, Ar–H), 7.31–7.26 (m, 1H, Ar–H), 4.66 (dd, *J* = 15.6, 2.5 Hz, 1H, OC*H*H), 4.59 (dd, *J* = 15.6, 2.5 Hz, 1H, OCH*H*), 3.47 (d, *J* = 0.6 Hz, 3H, SCH_3_), 2.42 (t, *J* = 2.5 Hz, 1H, CCH). ^13^C{^1^H} NMR (101 MHz, CDCl_3_) δ 159.7 (C=O), 157.3 (Ar–C_q_), 136.6 (Ar–C), 131.0 (Ar–C), 125.5 (Ar–C_q_), 125.1 (Ar–C), 117.4 (Ar–C), 77.8 (*C*CH), 74.7 (C*C*H), 53.5 (OCH_2_), 43.5 (SCH_3_). HRMS (ESI-TOF) *m*/*z*: Calcd. for C_11_H_11_NO_3_SF [M + H]^+^: 256.0444, found: 256.0452.

#### Prop-2-yn-1-yl (Methyl(oxo)(o-tolyl)-λ^6^-sulfaneylidene)carbamate (**2i**)

Prepared using General Procedure using sulfoxide **1i** (77.0 mg, 0.50 mmol) and prop-2-yn-1-yl carbamate (84.2 mg, 0.85 mmol, 1.7 equiv). Purification by flash column chromatography (SiO_2_, 40% EtOAc in hexane) afforded sulfoximine propargyl carbamate **2i** (39.9 mg, 32%) as a colorless gum. R_*f*_ = 0.27 (40% EtOAc in hexane); IR (film)/cm^–1^ 3265, 3019, 2937, 2125, 1670 (C=O), 1446, 1223, 1118, 977, 865, 753; ^1^H NMR (400 MHz, CDCl_3_) δ 8.11–8.09 (m, 1H, Ar–H), 7.58–7.54 (m, 1H, Ar–H), 7.46–7.42 (m, 1H, Ar–H), 7.39–7.37 (m, 1H, Ar–H), 4.68–4.64 (m, 1H, OC*H*H), 4.64–4.59 (m, 1H, OCH*H*), 3.34 (s, 3H, SCH_3_), 2.71 (s, 3H, Ar–CH_3_), 2.41 (t, *J* = 2.5 Hz, 1H, CCH); ^13^C{^1^H} NMR (101 MHz, CDCl_3_) δ 157.8 (C=O), 137.2 (Ar–C_q_), 136.0 (Ar–C_q_), 134.0 (Ar–C), 133.3 (Ar–C), 129.6 (Ar–C), 127.1 (Ar–C), 78.0 (*C*CH), 74.6 (C*C*H), 53.4 (OCH_2_), 43.3 (SCH_3_), 20.5 (Ar–CH_3_); HRMS (ESI-TOF) *m*/*z*: Calcd. for C_12_H_14_NO_3_S [M + H]^+^: 252.0694, found: 252.0699.

#### Prop-2-yn-1-yl (Benzyl(oxo)(phenyl)-λ^6^-sulfaneylidene)carbamate (**2j**)

Prepared using General Procedure using sulfoxide **1j** (109.0 mg, 0.50 mmol) and prop-2-yn-1-yl carbamate (84.2 mg, 0.85 mmol, 1.7 equiv). Purification by flash column chromatography (SiO_2_, CH_2_Cl_2_:Et_2_O:hexane = 20:30:50) afforded sulfoximine propargyl carbamate **2j** (117.4 mg, 74%) as a white solid. R_*f*_ = 0.25 (CH_2_Cl_2_:Et_2_O:hexane = 20:30:50); mp = 100–102 °C; IR (film)/cm^–1^ 3250, 3064, 2132, 1655 (C=O), 1520, 1446, 1267, 1074, 992, 880, 790, 738; ^1^H NMR (400 MHz, CDCl_3_) δ 7.64–7.59 (m, 3H, 3 × Ar–H), 7.46–7.42 (m, 2H, 2 × Ar–H), 7.32–7.27 (m, 1H, Ar–H), 7.22–7.18 (m, 2H, 2 × Ar–H), 6.96–6.94 (m, 2H, 2 × Ar–H), 4.77 (d, *J* = 15.9 Hz, 1H, SC*H*H), 4.73 (d, *J* = 15.9 Hz, 1H, SCH*H*), 4.72 (dd, *J* = 15.6, 2.5 Hz, 1H, OC*H*H), 4.65 (dd, *J* = 15.6, 2.5 Hz, 1H, OCH*H*), 2.45 (t, *J* = 2.5 Hz, 1H, CCH); ^13^C{^1^H} NMR (101 MHz, CDCl_3_) δ 158.3 (C=O), 134.4 (Ar–C_q_), 134.0 (Ar–C_q_), 131.1 (2 × Ar–C), 129.2 (Ar–C), 129.0 (2 × Ar–C), 128.6 (2 × Ar–C), 128.5 (2 × Ar–C), 126.6 (Ar–C), 78.0 (*C*CH), 74.6 (C*C*H), 62.0 (SCH_2_), 53.4 (OCH_2_); HRMS (ESI-TOF) *m*/*z*: Calcd. for C_17_H_16_NO_3_S [M + H]^+^: 314.0851, found: 314.0851.

#### Prop-2-yn-1-yl (Oxo(phenethyl)(p-tolyl)-λ^6^-sulfaneylidene)carbamate (**2k**)

Prepared using General Procedure using sulfoxide **1k** (123.4 mg, 0.50 mmol) and prop-2-yn-1-yl carbamate (84.2 mg, 0.85 mmol, 1.7 equiv). Purification by flash column chromatography (SiO_2_, CH_2_Cl_2_:Et_2_O:hexane = 20:15:65) afforded sulfoximine propargyl carbamate **2k** (118.9 mg, 70%) as a white solid. R_*f*_ = 0.29 (CH_2_Cl_2_:Et_2_O:hexane = 20:15:65); mp = 79–81 °C; IR (film)/cm^–1^ 3310, 3034, 2982, 2182, 1670 (C=O), 1595, 1498, 1372, 1215, 1118, 984, 775, 626, 492; ^1^H NMR (400 MHz, CDCl_3_) δ 7.86 (d, *J* = 8.4 Hz, 2H, 2 × Ar–H), 7.42–7.39 (m, 2H, 2 × Ar–H), 7.28–7.21 (m, 3H, 3 × Ar–H), 7.11–7.09 (m, 2H, 2 × Ar–H), 4.69–4.65 (m, 1H, OC*H*H), 4.64–4.60 (m, 1H, OCH*H*), 3.71 (ddd, *J* = 14.0, 11.9, 5.1 Hz, 1H, SC*H*H), 3.59 (ddd, *J* = 13.9, 11.7, 5.0 Hz, 1H, SCH*H*), 3.10 (ddd, *J* = 13.8, 11.7, 5.0 Hz, 1H, PhC*H*H), 2.97 (ddd, *J* = 13.8, 11.9, 5.0 Hz, 1H, PhCH*H*), 2.43 (t, *J* = 2.4 Hz, 1H, CCH); ^13^C{^1^H} NMR (101 MHz, CDCl_3_) δ 158.1 (C=O), 145.3 (Ar–C_q_), 136.6 (Ar–C_q_), 133.0 (Ar–C_q_), 130.3 (2 × Ar–C), 128.8 (2 × Ar–C), 128.3 (2 × Ar–C), 128.0 (2 × Ar–C), 127.0 (Ar–C_q_), 78.0 (*C*CH), 74.6 (C*C*H), 57.2 (SCH_2_), 53.4 (OCH_2_), 28.3 (PhCH_2_), 21.6 (Ar–CH_3_); HRMS (ESI-TOF) *m*/*z*: Calcd. for C_19_H_20_NO_3_S [M + H]^+^: 342.1164, found: 342.1168.

#### Prop-2-yn-1-yl (Benzyl(oxo)(phenyl)-λ^6^-sulfaneylidene)carbamate (**2l**)

Prepared using General Procedure using sulfoxide **1l** (85.0 mg, 0.51 mmol) and prop-2-yn-1-yl carbamate (84.2 mg, 0.85 mmol, 1.7 equiv). Purification by flash column chromatography (SiO_2_, CH_2_Cl_2_:Et_2_O:hexane = 30:20:50) afforded sulfoximine propargyl carbamate **2l** (85.5 mg, 63%) as a colorless gum. R_*f*_ = 0.3 (CH_2_Cl_2_:Et_2_O:hexane = 30:20:50); IR (film)/cm^–1^ 3340, 3263, 2937, 2125, 1677 (C=O), 1521, 1431, 1208, 1074, 969, 738. ^1^H NMR (400 MHz, CDCl_3_) δ 7.80 (d, *J* = 8.3 Hz, 2H, 2 × Ar–H), 7.39 (d, *J* = 8.1 Hz, 2H, 2 × Ar–H), 4.66–4.61 (m, 1H, OC*H*H), 4.60–4.56 (m, 1H, OCH*H*), 3.49–3.42 (m, 1H, SC*H*H), 3.41–3.34 (m, 1H, SCH*H*) 2.45 (s, 3H, Ar–CH_3_), 2.40 (t, *J* = 2.5 Hz, 1H, CCH), 1.25 (t, *J* = 7.4 Hz, 3H, CH_3_); ^13^C{^1^H} NMR (101 MHz, CDCl_3_) δ 158.0 (C=O), 145.1 (Ar–C_q_), 132.2 (Ar–C_q_), 130.2 (2 × Ar–C), 128.0 (2 × Ar–C), 77.9 (*C*CH), 74.4 (C*C*H), 53.1 (OCH_2_), 50.6 (SCH_2_), 21.5 (Ar–CH_3_), 6.8 (CH_3_); HRMS (ESI-TOF) *m*/*z*: Calcd. for C_13_H_16_NO_3_S [M + H]^+^: 266.0851, found: 266.0848.

#### Prop-2-yn-1-yl ((Chloromethyl)(oxo)(p-tolyl)-λ^6^-sulfaneylidene)carbamate (**2m**)

Prepared using General Procedure using sulfoxide **1m** (94.0 mg, 0.50 mmol) and prop-2-yn-1-yl carbamate (84.2 mg, 0.85 mmol, 1.7 equiv). Purification by flash column chromatography (SiO_2_, 20% EtOAc in hexane) afforded sulfoximine propargyl carbamate **2m** (115.4 mg, 77%) as a white solid. R_*f*_ = 0.22 (20% EtOAc in hexane); mp = 83–84 °C; IR (film)/cm^–1^ 3271, 3014, 2944, 2122, 1671 (C=O), 1592, 1432, 1368, 1234, 1084, 975, 923, 881, 780, 633, 522; ^1^H NMR (400 MHz, CDCl_3_) δ 7.93–7.91 (m, 2H, 2 × Ar–H), 7.45–7.42 (m, 2H, 2 × Ar–H), 5.20 (d, *J* = 11.9 Hz, 1H, SC*H*H), 4.87 (d, *J* = 12.0 Hz, 1H, SCH*H*), 4.76–4.72 (m, 1H, OC*H*H), 4.72–4.68 (m, 1H, OCH*H*), 2.49 (s, 3H, Ar–CH_3_), 2.48 (t, *J* = 2.5 Hz, 1H, CCH); ^13^C{^1^H} NMR (101 MHz, CDCl_3_) δ 157.7 (C=O), 146.6 (Ar–C_q_), 130.3 (2 × Ar–C), 129.5 (Ar–C_q_), 129.4 (2 × Ar–C), 77.7 (*C*CH), 75.0 (C*C*H), 58.5 (SCH_2_), 53.8 (OCH_2_), 21.7 (Ar–CH_3_); HRMS (ESI-TOF) *m*/*z*: Calcd. for C_12_H_13_NO_3_S^35^Cl [M + H]^+^: 286.0305, found: 286.0298.

#### Prop-2-yn-1-yl (Cyclopropyl(oxo)(phenyl)-λ^6^-sulfaneylidene)carbamate (**2n**)

Prepared using General Procedure using sulfoxide **1n** (83.4 mg, 0.50 mmol) and prop-2-yn-1-yl carbamate (84.2 mg, 0.85 mmol, 1.7 equiv). Purification by flash column chromatography (SiO_2_, 50% Et_2_O in hexane) afforded sulfoximine propargyl carbamate **2n** (91.3 mg, 70%) as a colorless gum. R_*f*_ = 0.12 (50% Et_2_O in hexane); IR (film)/cm^–1^ 3265, 3056, 2945, 2117, 1677 (C=O), 1446, 1245, 1088, 977, 880, 783; ^1^H NMR (400 MHz, CDCl_3_) δ 7.93–7.91 (m, 2H, 2 × Ar–H), 7.68–7.64 (m, 1H, Ar–H), 7.61–7.56 (m, 2H, 2 × Ar–H), 4.63–4.58 (m, 1H, OC*H*H), 4.58–4.53 (m, 1H, OCH*H*), 2.68–2.62 (m, 1H, SCH), 2.39 (d, *J* = 2.5 Hz, 1H, CCH), 1.68–1.59 (m, 1H, CH), 1.28–1.19 (m, 2H, 2 × CH), 1.05–0.94 (m, 1H, CH); ^13^C{^1^H} NMR (101 MHz, CDCl_3_) δ 157.7 (C=O), 138.3 (Ar–C_q_), 133.6 (Ar–C), 129.5 (2 × Ar–C), 127.4 (2 × Ar–C), 77.9 (*C*CH), 74.5 (C*C*H), 53.2 (OCH_2_), 33.4 (SCH), 6.8 (CH_2_), 5.2 (CH_2_); HRMS (ESI-TOF) *m*/*z*: Calcd. for C_13_H_14_NO_3_S [M + H]^+^: 264.0694, found: 264.0695.

#### Prop-2-yn-1-yl (Isopropyl(oxo)(p-tolyl)-λ^6^-sulfaneylidene)carbamate (**2o**)

Prepared using General Procedure using sulfoxide **1o** (90.0 mg, 0.50 mmol) and prop-2-yn-1-yl carbamate (84.2 mg, 0.85 mmol, 1.7 equiv). Purification by flash column chromatography (SiO_2_, CH_2_Cl_2_:Et_2_O:hexane = 30:30:40) afforded sulfoximine propargyl carbamate **2o** (48.5 mg, 35%) as a pale-yellow gum; R_*f*_ = 0.43 (CH_2_Cl_2_:Et_2_O:hexane = 30:30:40); IR (film)/cm^–1^ 3265, 2937, 2125, 1677 (C=O), 1431, 1372, 1238, 1088, 977, 872, 723, 641; ^1^H NMR (400 MHz, CDCl_3_) δ 7.77 (d, *J* = 8.4 Hz, 2H, 2 × Ar–H), 7.40–7.38 (m, 2H, 2 × Ar–H), 4.66–4.62 (m, 1H, OC*H*H), 4.61–4.57 (m, 1H, OCH*H*), 3.54 (hept, *J* = 6.8 Hz, 1H, SCH), 2.46 (s, 3H, Ar–CH_3_), 2.40 (t, *J* = 2.5 Hz, 1H, CCH), 1.41 (d, *J* = 6.8 Hz, 3H, CHC*H*_3_), 1.24 (d, *J* = 6.8 Hz, 3H, CHC*H*_3_); ^13^C{^1^H} NMR (101 MHz, CDCl_3_) δ 158.3 (C=O), 145.1 (Ar–C_q_), 130.8 (Ar–C_q_), 130.2 (2 × Ar–C), 129.0 (2 × Ar–C), 78.2 (*C*CH), 74.4 (C*C*H), 56.4 (SCH), 53.3 (CH_2_), 21.6 (Ar–CH_3_), 15.6 (CH*C*H_3_), 15.0 (CH*C*H_3_); HRMS (ESI-TOF) *m*/*z*: Calcd. for C_14_H_18_NO_3_S [M + H]^+^: 280.1007, found: 280.0999.

#### Prop-2-yn-1-yl (Oxodiphenyl-λ^6^-sulfaneylidene)carbamate (**2p**)

Prepared using General Procedure using sulfoxide **1p** (101.0 mg, 0.50 mmol) and prop-2-yn-1-yl carbamate (84.2 mg, 0.85 mmol, 1.7 equiv). Purification by flash column chromatography (SiO_2_, 40% Et_2_O in pentane) afforded sulfoximine propargyl carbamate **2p** (57.6 mg, 38%) as a colorless gum. R_*f*_ = 0.13 (40% Et_2_O in pentane). IR (film)/cm^–1^ 3271, 3084, 3056, 2124, 1667 (C=O), 1443, 1368, 1226, 1135, 1074, 982, 962, 877, 679, 575, 554. ^1^H NMR (400 MHz, CDCl_3_) δ 8.03–8.00 (m, 4H, 4 × Ar–H), 7.62–7.58 (m, 2H, 2 × Ar–H), 7.55–7.51 (m, 4H, 4 × Ar–H), 4.65 (d, *J* = 2.4 Hz, 2H, OCH_2_), 2.41 (t, *J* = 2.5 Hz, 1H, CCH). ^13^C{^1^H} NMR (101 MHz, CDCl_3_) δ 157.8 (C=O), 139.1 (2 × Ar–C_q_), 133.5 (2 × Ar–C), 129.5 (4 × Ar–C), 127.7 (4 × Ar–C), 77.9 (*C*CH), 74.6 (C*C*H), 53.5 (CH_2_). HRMS (ESI-TOF) *m*/*z*: Calcd. for C_16_H_14_NO_3_S [M + H]^+^: 300.0694; found: 300.0700.

#### Prop-2-yn-1-yl (Dibenzyl(oxo)-λ^6^-sulfaneylidene)carbamate (**2q**)

Prepared using General Procedure using sulfoxide **1q** (115.2 mg, 0.50 mmol) and prop-2-yn-1-yl carbamate (84.2 mg, 0.85 mmol, 1.7 equiv). Purification by flash column chromatography (SiO_2_, CH_2_Cl_2_:Et_2_O:hexane = 20:30:50) afforded sulfoximine propargyl carbamate **2q** (84.1 mg, 51%) as a white solid. R_*f*_ = 0.28 (CH_2_Cl_2_:Et_2_O:hexane = 20:30:50); mp = 124–126 °C; IR (film)/cm^–1^ 3289, 3058, 2983, 2933, 2118, 1651 (C=O), 1491, 1428, 1377, 1271, 1230, 1152, 1096, 1069, 964, 918, 873, 694, 600, 480; ^1^H NMR (400 MHz, CDCl_3_) δ 7.44–7.38 (m, 10H, 10 × Ar–H), 4.71 (d, *J* = 2.4 Hz, 2H, OCH_2_), 4.59 (d, *J* = 13.9 Hz, 2H, 2 × SC*H*H), 4.53 (d, *J* = 13.9 Hz, 2H, 2 × SCH*H*), 2.49 (t, *J* = 2.5 Hz, 1H, CCH); ^13^C{^1^H} NMR (101 MHz, CDCl_3_) δ 158.4 (C=O), 131.3 (4 × Ar–C), 129.6 (2 × Ar–C), 129.1 (4 × Ar–C), 126.2 (2 × Ar–C_q_), 78.3 (*C*CH), 74.5 (C*C*H), 56.5 (2 × SCH_2_), 53.5 (OCH_2_); HRMS (ESI-TOF) *m*/*z*: Calcd. for C_18_H_18_NO_3_S [M + H]^+^: 328.1007, found: 328.1012.

#### Prop-2-yn-1-yl (Dibutyl(oxo)-λ^6^-sulfaneylidene)carbamate (**2r**)

Prepared using General Procedure using sulfoxide **1r** (81.4 mg, 0.50 mmol) and prop-2-yn-1-yl carbamate (84.2 mg, 0.85 mmol, 1.7 equiv). Purification by flash column chromatography (SiO_2_, CH_2_Cl_2_:Et_2_O:hexane = 20:30:50) afforded sulfoximine propargyl carbamate **2r** (60.9 mg, 47%) as a colorless gum. R_*f*_ = 0.24 (CH_2_Cl_2_:Et_2_O:hexane = 20:30:50); IR (film)/cm^–1^ 3250, 2960, 2878, 2125, 1662 (C=O), 1461, 1230, 1074, 977, 865, 783, 671; ^1^H NMR (400 MHz, CDCl_3_) δ 4.67 (d, *J* = 2.5 Hz, 2H, OCH_2_), 3.42–3.26 (m, 4H, 2 × SCH_2_), 2.44 (t, *J* = 2.5 Hz, 1H, CCH), 1.90–1.74 (m, 4H, 2 × SCH_2_C*H*_2_), 1.53–1.44 (m, 4H, CH_3_C*H*_2_), 0.97 (d, *J* = 7.3 Hz, 6H, 2 × CH_3_); ^13^C{^1^H} NMR (101 MHz, CDCl_3_) δ 158.3 (C=O), 78.1 (*C*CH), 74.5 (C*C*H), 53.3 (OCH_2_), 51.1 (2 × SCH_2_), 24.0 (2 × SCH_2_*C*H_2_), 21.6 (2 × *C*H_2_CH_3_), 13.5 (2 × CH_3_); HRMS (ESI-TOF) *m*/*z*: Calcd. for C_12_H_22_NO_3_S [M + H]^+^: 260.1320, found: 260.1317.

#### Prop-2-yn-1-yl (tert-Butyl(methyl)(oxo)-λ^6^-sulfaneylidene)carbamate (**2s**)

Prepared using General Procedure using sulfoxide **1s** (57.8 mg, 0.48 mmol) and prop-2-yn-1-yl carbamate (84.2 mg, 0.85 mmol, 1.7 equiv). Purification by flash column chromatography (SiO_2_, 40% EtOAc in hexane) afforded sulfoximine propargyl carbamate **2s** (41.0 mg, 39%) as a colorless oil. R_*f*_ = 0.15 (40% EtOAc in hexane); IR (film)/cm^–1^ 3265, 2915, 2125, 1670 (C=O), 1461, 1260, 992, 872, 783, 723; ^1^H NMR (400 MHz, CDCl_3_) δ 4.73–4.69 (m, 1H, OC*H*H), 4.86–4.64 (m, 1H, OCH*H*), 3.28 (s, 3H, SCH_3_), 2.45 (t, *J* = 2.5 Hz, CCH), 1.51 (s, 9H, C(CH_3_)_3_); ^13^C{^1^H} NMR (101 MHz, CDCl_3_) δ 159.2 (C=O), 78.3 (*C*CH), 74.5 (C*C*H), 60.5 (*C*(CH_3_)_3_), 53.3 (OCH_2_), 32.1 (SCH_3_), 22.9 (C(*C*H_3_)_3_); HRMS (ESI-TOF) *m*/*z*: Calcd. for C_9_H_16_NO_3_S [M + H]^+^: 218.0851, found: 218.0854.

#### Prop-2-yn-1-yl (1-Oxido-1λ^6^-thietan-1-ylidene)carbamate (**2t**)

Prepared using General Procedure using sulfoxide **1t** (45.6 mg, 0.50 mmol) and prop-2-yn-1-yl carbamate (84.2 mg, 0.85 mmol, 1.7 equiv). Purification by flash column chromatography (SiO_2_, 50% EtOAc in hexane) afforded sulfoximine propargyl carbamate **2t** (79.2 mg, 85%) as a pale-red gum. R_*f*_ = 0.16 (50% EtOAc in hexane). IR (film)/cm^–1^ 3264, 3029, 2957, 2121, 1654 (C=O), 1369, 1225, 1115, 992, 871, 782, 689, 553. ^1^H NMR (400 MHz, CDCl_3_) δ 4.69–4.67 (m, 2H, OCH_2_), 4.42–4.33 (m, 2H, 2 × SC*H*H), 4.30–4.22 (m, 2H, 2 × SCH*H*), 2.47–2.46 (m, 1H, CCH), 2.44–2.34 (m, 2H, CH_2_). ^13^C{^1^H} NMR (101 MHz, CDCl_3_) δ 158.4 (C=O), 77.8 (*C*CH), 74.8 (C*C*H), 62.9 (2 × SCH_2_), 53.5 (OCH_2_), 9.4 (CH_2_). HRMS (ESI-TOF) *m*/*z*: Calcd. for C_7_H_10_NO_3_S [M + H]^+^: 188.0381, found: 188.0375.

#### Prop-2-yn-1-yl (Methyl(oxo)(pyridin-2-yl)-λ^6^-sulfaneylidene)carbamate (**2u**)

Prepared using General Procedure using sulfoxide **1u** (69.7 mg, 0.49 mmol) and prop-2-yn-1-yl carbamate (84.2 mg, 0.85 mmol, 1.7 equiv). Purification by flash column chromatography (SiO_2_, 60% EtOAc in hexane) afforded sulfoximine propargyl carbamate **2u** (57.2 mg, 49%) as a pale-red gum. R_*f*_ = 0.21 (60% EtOAc in hexane); IR (film)/cm^–1^ 3265, 3019, 2930, 2125, 1670 (C=O), 1431, 1223, 1111, 984, 872, 760; ^1^H NMR (400 MHz, CDCl_3_) δ 8.74 (ddd, *J* = 4.7, 1.7, 0.9 Hz, 1H, Ar–H), 8.29 (dt, *J* = 7.9, 1.0 Hz, 1H, Ar–H), 8.02 (td, *J* = 7.8, 1.7 Hz, 1H, Ar–H), 7.59 (ddd, *J* = 7.7, 4.7, 1.1 Hz, 1H, Ar–H), 4.63 (dd, *J* = 15.6, 2.5 Hz, 1H, OC*H*H), 4.56 (dd, *J* = 15.6, 2.5 Hz, 1H, OCH*H*), 3.47 (s, 3H, SCH_3_), 2.40 (t, *J* = 2.5 Hz, 1H, CCH); ^13^C{^1^H} NMR (101 MHz, CDCl_3_) δ 157.9 (C=O), 156.0 (Ar–C_q_), 150.1 (Ar–C), 138.3 (Ar–C), 127.6 (Ar–C), 123.5 (Ar–C), 77.8 (*C*CH), 74.7 (C*C*H), 53.4 (OCH_2_), 39.8 (SCH_3_); HRMS (ESI-TOF) *m*/*z*: Calcd. for C_10_H_11_N_2_O_3_S [M + H]^+^: 239.0490, found: 239.0498.

#### Prop-2-yn-1-yl ((2-Hydroxyethyl)(oxo)(phenyl)-λ^6^-sulfaneylidene)carbamate (**2v**)

Prepared using General Procedure using sulfoxide **1v** (86.8 mg, 0.51 mmol) and prop-2-yn-1-yl carbamate (84.2 mg, 0.85 mmol, 1.7 equiv). Purification by flash column chromatography (SiO_2_, 60% EtOAc in hexane) afforded sulfoximine propargyl carbamate **2v** (52.0 mg, 38%) as a colorless gum. R_*f*_ = 0.24 (60% EtOAc in hexane); IR (film)/cm^–1^ 3429, 3273, 3064, 2937, 2125, 1670 (C=O), 1446, 1372, 1223, 1081, 969, 872, 783, 686; ^1^H NMR (400 MHz, CDCl_3_) δ 7.99–7.97 (m, 2H, 2 × Ar–H), 7.75–7.71 (m, 1H, Ar–H), 7.66–7.62 (m, 2H, 2 × Ar–H), 4.65 (dd, *J* = 15.6, 2.5 Hz, 1H, OC*H*H), 4.60 (dd, *J* = 15.6, 2.5 Hz, 1H, OCH*H*), 4.19–4.11 (m, 1H, C*H*HOH), 4.08–4.00 (m, 1H, CH*H*OH), 3.68–3.62 (m, 1H, SC*H*H), 3.52–3.46 (m, 1H, SCH*H*), 3.41–3.37 (m, 1H, OH); ^13^C{^1^H} NMR (101 MHz, CDCl_3_) δ 157.7 (C=O), 136.7 (Ar–C_q_), 134.4 (Ar–C), 129.9 (2 × Ar–C), 127.9 (2 × Ar–C), 77.7 (*C*CH), 74.8 (d, C*C*H), 58.9 (SCH_2_), 55.8 (SCH_2_*C*H_2_), 53.6 (OCH_2_); HRMS (ESI-TOF) *m*/*z*: Calcd. for C_12_H_13_NO_4_NaS [M + Na]^+^: 290.0463, found: 290.0451.

#### Prop-2-yn-1-yl (Oxo(phenyl)(vinyl)-λ^6^-sulfaneylidene)carbamate (**2w**)

Prepared using General Procedure using sulfoxide **1w** (78.8 mg, 0.52 mmol) and prop-2-yn-1-yl carbamate (84.2 mg, 0.85 mmol, 1.7 equiv). Purification by flash column chromatography (SiO_2_, 50% Et_2_O in hexane) afforded sulfoximine propargyl carbamate **2w** (79.0 mg, 63%) as a colorless gum. R_*f*_ = 0.12 (50% Et_2_O in hexane). IR (film)/cm^–1^ 3263, 3055, 2121, 1673 (C=O), 1443, 1368, 1230, 1082, 972, 873, 744, 686, 635, 536, 494. ^1^H NMR (400 MHz, CDCl_3_) δ 7.98–7.96 (m, 2H, 2 × Ar–H), 7.68–7.65 (m, 1H, Ar–H), 7.61–7.57 (m, 2H, 2 × Ar–H), 6.75 (dd, *J* = 16.3, 9.6 Hz, 1H, SCH), 6.54 (dt, *J* = 16.3, 1.1 Hz, 1H, SCHC*H*H), 6.21 (dd, *J* = 9.6 Hz, 1.1 Hz, 1H, SCHCH*H*), 4.70–4.65 (m, 1H, OC*H*H), 4.65–4.61 (m, 1H, OCH*H*), 2.43 (t, *J* = 2.5 Hz, 1H, CCH). ^13^C{^1^H} NMR (101 MHz, CDCl_3_) δ 157.7 (C=O), 137.0 (Ar–C_q_), 136.5 (SCH), 134.0 (Ar–C), 129.6 (2 × Ar–C), 129.5 (HC*C*H_2_), 127.9 (2 × Ar–C), 77.9 (*C*CH), 74.7 (C*C*H), 53.5 (OCH_2_). HRMS (ESI-TOF) *m*/*z*: Calcd. for C_12_H_12_NO_3_S [M + H]^+^: 250.0538, found: 250.0533.

#### tert-Butyl 2-((tert-Butoxycarbonyl)amino)-4-(S-methyl-N-((prop-2-yn-1-yloxy)carbonyl)sulfonimidoyl)-butanoate (**2x**)

Prepared using General Procedure using sulfoxide **1x** (161.4 mg, 0.50 mmol) and prop-2-yn-1-yl carbamate (84.2 mg, 0.85 mmol, 1.7 equiv). Purification by flash column chromatography (SiO_2_, 35% EtOAc in hexane) afforded sulfoximine propargyl carbamate **2x** (160.8 mg, 77%) as a colorless gum. R_*f*_ = 0.22 (35% EtOAc in hexane); IR (film)/cm^–1^ 3355, 3280, 2982, 2125, 1707 (C=O), 1670 (C=O), 1513, 1372, 1238, 1148, 1044, 992, 872, 783, 634, 497; ^1^H NMR (400 MHz, CDCl_3_) δ 5.30–5.28 (m, 1H, NH), 4.65–4.64 (m, 2H, OCH_2_), 4.24–4.21 (m, 1H, HCCO_2_), 3.55–3.31 (m, 2H, SCH_2_), 3.26 (d, *J* = 7.3 Hz, 3H, SCH_3_), 2.45–2.44 (m, 1H, CCH), 2.43–2.35 (m, 1H, SCH_2_C*H*H), 2.21–2.08 (m, 1H, SCH_2_CH*H*), 1.45 (s, 9H, CHCO_2_(C*H*_3_)_3_), 1.41 (s, 9H, NHCO_2_(C*H*_3_)_3_); ^13^C{^1^H} NMR (101 MHz, CDCl_3_) δ 169.9 (C=O), 158.1 (C=O), 155.4 (C=O), 83.2 (*C*(CH_3_)_3_), 80.3 (*C*(CH_3_)_3_), 77.9 (*C*CH), 74.7 (C*C*H), 53.3 (OCH_2_), 52.3 (*C*HCO_2_), 50.6 (SCH_2_), 39.3 (d, SCH_3_), 28.2 (C(*C*H_3_)_3_), 27.8 (C(*C*H_3_)_3_), 25.7 (d, SCH_2_*C*H_2_); HRMS (ESI-TOF) *m*/*z*: Calcd. for C_18_H_30_N_2_O_7_SNa [M + Na]^+^: 441.1671, found: 441.1668.

#### tert-Butyl (Methyl(oxo)(p-tolyl)-λ^6^-sulfaneylidene)carbamate (**4**)

Prepared using General Procedure using sulfoxide **1b** (76.8 mg, 0.50 mmol) and *tert*-butylcarbamate (99.6 mg, 0.85 mmol, 1.7 equiv). Purification by flash column chromatography (SiO_2_, 50% EtOAc in hexane) afforded *N*-Boc sulfoximine **4** (132.9 mg, 99%) as a white solid. R_*f*_ = 0.5 (50% EtOAc in hexane); mp = 108–112 °C; IR (film)/cm^–1^ 3027, 2974, 2109, 1662 (C=O), 1364, 1267, 1148, 984, 865, 790; ^1^H NMR (400 MHz, CDCl_3_) δ 7.84 (d, *J* = 8.4 Hz, 2H, 2 × Ar–H), 7.37 (d, *J* = 8.4 Hz, 2H, 2 × Ar–H), 3.21 (s, 3H, SCH_3_), 2.44 (s, 3H, Ar–CH_3_), 1.37 (s, 9H, C(CH_3_)_3_); ^13^C{^1^H} NMR (101 MHz, CDCl_3_) δ 157.6 (C=O), 144.7 (Ar–C_q_), 135.6 (Ar–C_q_), 130.2 (2 × Ar–C), 127.3 (2 × Ar–C), 80.4 (*C*(CH_3_)_3_), 44.8 (SCH_3_), 27.9 (C(*C*H_3_)_3_), 21.5 (Ar–CH_3_). Analytical data (NMR and IR) in agreement with those reported in the literature.^14^

#### Methyl (Methyl(oxo)(p-tolyl)-λ^6^-sulfaneylidene)carbamate (**5**)

Prepared using General Procedure using sulfoxide **1b** (75.9 mg, 0.49 mmol) and methyl carbamate (63.8 mg, 0.85 mmol, 1.7 equiv). Purification by flash column chromatography (SiO_2_, 50% EtOAc in hexane) afforded *N*-CO_2_Me sulfoximine **5** (111.5 mg, 99%) as a colorless oil. R_*f*_ = 0.26 (50% EtOAc in hexane); mp = 92–96 °C; IR (film)/cm^–1^ 3019, 2930, 2117, 1670 (C=O), 1431, 1223, 1088, 984, 872, 790; ^1^H NMR (400 MHz, CDCl_3_) δ 7.87 (d, *J* = 8.4 Hz, 2H, 2 × Ar–H), 7.40 (d, *J* = 8.2 Hz, 2H, 2 × Ar–H), 3.67 (s, 3H, CO_2_CH_3_), 3.30 (s, 3H, SCH_3_), 2.46 (s, 3H, Ar–CH_3_); ^13^C{^1^H} NMR (101 MHz, CDCl_3_) δ 159.4 (C=O), 145.1 (Ar–C_q_), 135.1 (Ar–C_q_), 130.3 (2 × Ar–C), 127.4 (2 × Ar–C), 53.1 (CO_2_*C*H_3_), 44.6 (SCH_3_), 21.6 (Ar–CH_3_). Analytical data (NMR and IR) in agreement with those reported in the literature.^14^

#### Benzyl (Methyl(oxo)(p-tolyl)-λ^6^-sulfaneylidene)carbamate (**6**)

Prepared using General Procedure using sulfoxide **1b** (76.8 mg, 0.50 mmol) and benzyl carbamate (128.5 mg, 0.85 mmol, 1.7 equiv). Purification by flash column chromatography (SiO_2_, 50% EtOAc in hexane) afforded *N*-Cbz sulfoximine **6** (144.8 mg, 95%) as a white solid. R_*f*_ = 0.4 (50% EtOAc in hexane); mp = 90–94 °C; IR (film)/cm^–1^ 3027, 2922, 2110, 1655 (C=O), 1379, 1215, 1088, 969, 895 775; ^1^H NMR (400 MHz, CDCl_3_) δ 7.82 (d, *J* = 8.4 Hz, 2H, 2 × Ar–H), 7.35 (d, *J* = 8.4 Hz, 2H, 2 × Ar–H), 7.29–7.22 (m, 5H, 5 × Ar–H), 5.09 (d, *J* = 12.3 Hz, 1H, OC*H*H), 5.01 (d, *J* = 12.3 Hz, 1H, OCH*H*), 3.25 (s, 3H, SCH_3_), 2.43 (s, 3H, Ar–CH_3_); ^13^C{^1^H} NMR (101 MHz, CDCl_3_) δ 158.4 (C=O), 144.9 (Ar–C_q_), 136.1 (Ar–C_q_), 134.9 (Ar–C_q_), 130.1 (2 × Ar–C), 128.1 (2 × Ar–C), 128.0 (2 × Ar–C), 127.7 (Ar–C), 127.2 (2 × Ar–C), 67.5 (OCH_2_), 44.5 (SCH_3_), 21.4 (Ar–CH_3_). Analytical data (NMR and IR) in agreement with those reported in the literature.^14^

#### Phenyl (Methyl(oxo)(p-tolyl)-λ^6^-sulfaneylidene)carbamate (**7**)

Prepared using General Procedure using sulfoxide **1b** (76.0 mg, 0.49 mmol) and phenyl carbamate (116.6 mg, 0.85 mmol, 1.7 equiv). Purification by flash column chromatography (SiO_2_, 50% EtOAc in hexane) afforded *N*-CO_2_Ph sulfoximine **7** (128.7 mg, 91%) as a white solid. R_*f*_ = 0.5 (50% EtOAc in hexane); mp = 110–116 °C; IR (film)/cm^–1^ 3042, 2072, 1670 (C=O), 1491, 1260, 1185, 977, 880, 716; ^1^H NMR (400 MHz, CDCl_3_) δ 7.92 (d, *J* = 8.4 Hz, 2H, 2 × Ar–H), 7.43–7.41 (m, 2H, 2 × Ar–H), 7.34–7.30 (m, 2H, 2 × Ar–H), 7.18–7.11 (m, 3H, 3 × Ar–H), 3.38 (s, 3H, SCH_3_), 2.47 (s, 3H, Ar–CH_3_); ^13^C{^1^H} NMR (101 MHz, CDCl_3_) δ 157.2 (C=O), 151.3 (Ar–C_q_), 145.2 (Ar–C_q_), 134.6 (Ar–C_q_), 130.3 (2 × Ar–C), 129.0 (2 × Ar–C), 127.2 (2 × Ar–C), 125.1 (Ar–C), 121.5 (2 × Ar–C), 44.3 (SCH_3_), 21.5 (Ar–CH_3_). Analytical data (NMR and IR) in agreement with those reported in the literature.^14^

#### Allyl (Methyl(oxo)(p-tolyl)-λ^6^-sulfaneylidene)carbamate (**8**)

Prepared using General Procedure using sulfoxide **1b** (77.0 mg, 0.50 mmol) and allyl carbamate (85.9 mg, 0.85 mmol, 1.7 equiv). Purification by flash column chromatography (SiO_2_, 30% EtOAc in hexane) afforded sulfoximine allyl carbamate **8** (104.4 mg, 82%) as a colorless oil. R_*f*_ = 0.13 (30% EtOAc in hexane); IR (film)/cm^–1^ 3019, 2930, 2110, 1670 (C=O), 1446, 1357, 1223, 1088, 977, 872, 790; ^1^H NMR (400 MHz, CDCl_3_) δ 7.84 (d, *J* = 8.2 Hz, 2H, 2 × Ar–H), 7.38 (d, *J* = 8.2 Hz, 2H, 2 × Ar–H), 5.92–5.82 (m, 1H, C*H*CH_2_), 5.28–5.22 (m, 1H, CHC*H*H), 5.17–5.13 (m, 1H, CHCH*H*), 4.56–4.51 (m, 1H, OC*H*H), 4.51–4.46 (m, 1H, OCH*H*), 3.27 (s, 3H, SCH_3_), 2.43 (s, 3H, Ar–CH_3_); ^13^C{^1^H} NMR (101 MHz, CDCl_3_) δ 158.5 (C=O), 145.0 (Ar–C_q_), 135.0 (Ar–C_q_), 132.4 (*C*HCH_2_), 130.2 (2 × Ar–C), 127.3 (2 × Ar–C), 117.7 (CH*C*H_2_), 66.5 (OCH_2_), 44.5 (SCH_3_), 21.5 (Ar–CH_3_). Analytical data (NMR and IR) in agreement with those reported in the literature.^14^

#### 3-(Trimethylsilyl)prop-2-yn-1-yl (Methyl(oxo)(p-tolyl)-λ^6^-sulfaneylidene)carbamate (**9**)

Prepared using General Procedure using sulfoxide **1b** (76.4 mg, 0.50 mmol) and TMS-protected propargyl carbamate (145.6 mg, 0.85 mmol, 1.7 equiv). Purification by flash column chromatography (SiO_2_, 30% EtOAc in hexane) afforded sulfoximine propargyl carbamate **9** (150.0 mg, 93%) as a colorless gum. R_*f*_ = 0.38 (30% EtOAc in hexane); IR (film)/cm^–1^ 3027, 2960, 2117, 1670 (C=O), 1372, 1230, 1088, 1029, 977, 842, 760;^1^H NMR (400 MHz, CDCl_3_) δ 7.84–7.82 (m, 2H, 2 × Ar–H), 7.38–7.35 (m, 2H, 2 × Ar–H), 4.64–4.59 (m, 1H, OC*H*H), 4.58–4.54 (m, 1H, OCH*H*), 3.27 (s, 3H, SCH_3_), 2.42 (s, 3H, Ar–CH_3_), 0.11 (s, 9H, (CH_3_)_3_); ^13^C{^1^H} NMR (101 MHz, CDCl_3_) δ 158.0 (C=O), 145.1 (Ar–C_q_), 134.6 (Ar–C_q_), 130.2 (2 × Ar–C), 127.2 (2 × Ar–C), 99.3 (*C*CSi), 91.5 (C*C*Si), 54.1 (OCH_2_), 44.4 (SCH_3_), 21.5 (Ar–CH_3_), −0.5 ((CH_3_)_3_). HRMS (ESI-TOF) *m*/*z*: Calcd. for C_15_H_22_NO_3_SSi [M + H]^+^: 324.1090, found: 324.1081.

#### Prop-2-yn-1-yl (Z)-(Methyl(p-tolyl)-λ^4^-sulfaneylidene)carbamate (**12**)

Prepared using General Procedure using sulfide **10a** (69.1 mg, 0.50 mmol) and 2-propynyl carbamate (84.2 mg, 0.85 mmol, 1.7 equiv). Purification by flash column chromatography (SiO_2_, EtOAc) afforded sulfilimine propargyl carbamate **12** (38.1 mg, 32%) as a colorless gum. R_*f*_ = 0.36 (EtOAc); IR (film)/cm^–1^ 3235, 3019, 2922, 2117, 1759, 1625 (C=O), 1245, 1074, 954, 813, 671; ^1^H NMR (400 MHz, CDCl_3_) δ 7.68 (d, *J* = 8.3 Hz, 2H, 2 × Ar–H), 7.34 (d, *J* = 8.2 Hz, 2H, 2 × Ar–H), 4.67 (dd, *J* = 15.6, 2.4 Hz, 1H, OC*H*H), 4.63 (dd, *J* = 15.7, 2.4 Hz, 1H, OCH*H*), 2.82 (s, 3H, SCH_3_), 2.42 (s, 3H, Ar–CH_3_), 2.40 (t, *J* = 2.5 Hz, 1H, CCH); ^13^C{^1^H} NMR (101 MHz, CDCl_3_) δ 163.6 (C=O), 143.3 (Ar–C_q_), 132.7 (Ar–C_q_), 130.6 (2 × Ar–C), 126.4 (2 × Ar–C), 79.1 (*C*CH), 73.9 (C*C*H), 53.2 (OCH_2_), 36.1 (SCH_3_), 21.4 (Ar–CH_3_). HRMS (ESI-TOF) *m*/*z*: Calcd. for C_12_H_14_NO_2_S [M + H]^+^: 236.0745, found: 236.0755.

#### tert-Butyl [(Z)-Methyl(p-tolyl)-λ^4^-sulfanylidene]carbamate (**13**)

Prepared using General Procedure using sulfide **10a** (69.1 mg, 0.50 mmol) and *tert*-butyl carbamate (99.6 mg, 0.85 mmol, 1.7 equiv). Purification by flash column chromatography (SiO_2_, EtOAc) afforded *N*-Boc sulfilimine **13** (64.0 mg, 51%) as a white solid. R_*f*_ = 0.3 (EtOAc); mp = 152–155 °C; IR (film)/cm^–1^ 3011, 2975, 2925, 1625, 1361, 1275, 1163, 1079, 985, 835, 817, 786, 753; ^1^H NMR (400 MHz, CDCl_3_) δ 7.64 (d, *J* = 8.1 Hz, 2H, 2 × Ar–H), 7.31 (d, *J* = 8.2 Hz, 2H, 2 × Ar–H), 2.76 (s, 3H, SCH_3_), 2.39 (s, 3H, Ar–CH_3_), 1.44 (s, 9H, (CH_3_)_3_); ^13^C{^1^H} NMR (101 MHz, CDCl_3_) δ 164.4 (C=O), 142.8 (Ar–C_q_), 133.6 (Ar–C_q_), 130.5 (2 × Ar–C), 126.1 (2 × Ar–C), 78.9 (*C*(CH_3_)_3_), 35.8 (SCH_3_), 28.4 (C(*C*H_3_)_3_), 21.4 (Ar–CH_3_). Analytical data (NMR and IR) in agreement with those reported in the literature.^14^

#### tert-Butyl (Z)-((4-Methoxyphenyl)(methyl)-λ^4^-sulfaneylidene)carbamate (**14**)

Prepared using General Procedure using sulfide **10b** (77.5 mg, 0.50 mmol) and *tert*-butyl carbamate (99.6 mg, 0.85 mmol, 1.7 equiv). Purification by flash column chromatography (SiO_2_, EtOAc) afforded *N*-Boc sulfilimine **14** (77.3 mg, 61%) as a white solid. R_*f*_ = 0.15 (EtOAc); mp = 146–149 °C; IR (film)/cm^–1^ 3086, 3012, 1625 (C=O), 1498, 1260, 1156, 984, 835; ^1^H NMR (400 MHz, CDCl_3_) δ 7.72 (d, *J* = 9.0 Hz, 2H, 2 × Ar–H), 7.02 (d, *J* = 8.8 Hz, 2H, 2 × Ar–H), 3.86 (s, 3H, OCH_3_), 2.78 (s, 3H, SCH_3_), 1.46 (s, 9H, (CH_3_)_3_); ^13^C{^1^H} NMR (101 MHz, CDCl_3_) δ 164.5 (C=O), 162.7 (Ar–C_q_), 128.2 (2 × Ar–C), 127.5 (Ar–C_q_), 115.3 (2 × Ar–C), 78.8 (*C*(CH_3_)_3_), 55.6 (OCH_3_), 35.9 (SCH_3_), 28.4 (C(*C*H_3_)_3_). HRMS (ESI-TOF) *m*/*z*: Calcd. for C_13_H_20_NO_3_S [M + H]^+^: 270.1164, found: 270.1162.

#### tert-Butyl (Z)-((4-Chlorophenyl)(methyl)-λ^4^-sulfaneylidene)carbamate (**15**)

Prepared using General Procedure using sulfide **10c** (79.9 mg, 0.50 mmol) and *tert*-butyl carbamate (99.6 mg, 0.85 mmol, 1.7 equiv). Purification by flash column chromatography (SiO_2_, EtOAc) afforded *N*-Boc sulfilimine **15** (103.6 mg, 74%) as a white solid. R_*f*_ = 0.18 (EtOAc); mp = 142–144 °C; IR (film)/cm^–1^ 3012, 1625 (C=O), 1476, 1282, 1163, 835; ^1^H NMR (400 MHz, CDCl_3_) δ 7.72 (d, *J* = 8.6 Hz, 2H, 2 × Ar–H), 7.52 (d, *J* = 8.6 Hz, 2H, 2 × Ar–H), 2.80 (s, 3H, SCH_3_), 1.46 (s, 9H, (CH_3_)_3_); ^13^C{^1^H} NMR (101 MHz, CDCl_3_) δ 164.4 (C=O), 138.5 (Ar–C_q_), 135.6 (Ar–C_q_), 130.2 (2 × Ar–C), 127.4 (2 × Ar–C), 79.3 (*C*(CH_3_)_3_), 35.8 (SCH_3_), 28.3 (C(*C*H_3_)_3_). HRMS (ESI-TOF) *m*/*z*: Calcd. for C_12_H_17_NO_2_S^35^Cl [M + H]^+^: 274.0669, found: 274.0662.

#### Benzyl (Z)-(Methyl(p-tolyl)-λ^4^-sulfaneylidene)carbamate (**16**)

Prepared using General Procedure using sulfide **10a** (70.9 mg, 0.51 mmol) and benzyl carbamate (128.5 mg, 0.85 mmol, 1.7 equiv). Purification by flash column chromatography (SiO_2_, EtOAc) afforded *N*-Cbz sulfilimine **16** (131.1 mg, 89%) as a colorless oil. R_*f*_ = 0.19 (EtOAc); IR (film)/cm^–1^ 3027, 2922, 2095, 1625 (C=O), 1446, 1252, 1074, 969, 813, 746; ^1^H NMR (400 MHz, CDCl_3_) δ 7.67 (d, *J* = 8.1 Hz, 2H, 2 × Ar–H), 7.40–7.38 (m, 2H, 2 × Ar–H), 7.34–7.24 (m, 5H, 5 × Ar–H), 5.13 (d, *J* = 12.4 Hz, 1H, OC*H*H), 5.07 (d, *J* = 12.4 Hz, 1H, OCH*H*), 2.80 (s, 3H, SCH_3_), 2.41 (s, 3H, Ar–CH_3_); ^13^C{^1^H} NMR (101 MHz, CDCl_3_) δ 164.5 (C=O), 143.1 (Ar–C_q_), 137.3 (Ar–C_q_), 133.1 (Ar–C_q_), 130.6 (2 × Ar–C), 128.2 (2 × Ar–C), 128.1 (2 × Ar–C), 127.6 (Ar–C), 126.3 (2 × Ar–C), 67.5 (OCH_2_), 36.1 (SCH_3_), 21.4 (Ar–CH_3_). HRMS (ESI-TOF) *m*/*z*: Calcd. for C_16_H_18_NO_2_S [M + H]^+^: 288.1058, found: 288.1059.

#### tert-Butyl (Z)-(Phenyl(piperidin-1-yl)-λ^4^-sulfaneylidene)carbamate (**17**)

Prepared using General Procedure using 1-(phenylsulphenyl)piperidylamide **11** (58.0 mg, 0.3 mmol) and *tert*-butyl *carbamate* (59.7 mg, 0.51 mmol, 1.7 equiv). Purification by flash column chromatography (SiO_2_, 30% EtOAc in pentane) afforded sulfinamidine **17** (35.2 mg, 38%) as a colorless oil. R_*f*_ = 0.26 (30% EtOAc in pentane); IR (film)/cm^–1^ 2972, 2936, 2853, 2116, 1630 (C=O), 1273, 1247, 1161, 1029, 848, 751, 688, 548; ^1^H NMR (400 MHz, CDCl_3_) δ 7.91–7.83 (m, 2H, 2 × Ar–H), 7.53–7.47 (m, 3H, 3 × Ar–H), 3.18 (ddd, *J* = 11.6, 6.6, 4.1 Hz, 2H, NCH_2_), 2.95 (ddd, *J* = 11.7, 6.7, 3.9 Hz, 2H, NCH_2_), 1.77–1.55 (m, 4H, 2 × CH_2_), 1.53 (s, 9H, C(CH_3_)_3_), 1.53–1.50 (m, 2H, CH_2_); ^13^C{^1^H} NMR (101 MHz, CDCl_3_) δ 165.0 (C=O), 134.6 (Ar–C_q_), 131.2 (Ar–C), 128.9 (2 × Ar–C), 128.1 (2 × Ar–C), 78.7 (*C*(CH_3_)_3_), 48.2 (2 × NCH_2_), 28.4 (C(*C*H_3_)_3_), 26.1 (2 × NCH_2_*C*H_2_), 23.5 (NCH_2_CH_2_*C*H_2_); HRMS (ESI-TOF) *m*/*z*: Calcd. for C_16_H_25_N_2_O_2_S [M + H]^+^: 309.1637, found: 309.1630.

#### (1-Benzyl-1H-1,2,3-triazol-4-yl)methyl (Methyl(oxo)(phenyl)-λ^6^-sulfaneylidene)carbamate (**18**)

CuSO_4_ anhydrous (2.4 mg, 0.015 mol, 5 mol %) was added to sulfoximine **2a** (71.2 mg, 0.3 mmol, 1.0 equiv), benzyl azide (47.9 mg, 0.36 mmol, 1.2 equiv), and sodium ascorbate (11.9 mg, 0.06 mmol, 20 mol %) in *tert*-butyl alcohol (1.0 mL) and H_2_O (0.5 mL) at rt. The resulting mixture was stirred at rt for 24 h, then quenched with saturated aqueous NH_4_Cl solution (1.0 mL), and extracted with CH_2_Cl_2_ (3 × 5 mL). The combined organic layer was dried over Na_2_SO_4_, and the solvent was removed under reduced pressure. Purification by flash column chromatography (SiO_2_, 5% EtOH in EtOAc) afforded triazole **18** (77.1 mg, 72%) as a white solid. R_*f*_ = 0.52 (5% EtOH in EtOAc); mp = 131–134 °C; IR (film)/cm^–1^ 3129, 3089, 3062, 2957, 2923, 2106, 1660 (C=O), 1443, 1338, 1249, 1224, 1084, 945, 859, 720, 683; ^1^H NMR (400 MHz, CDCl_3_) δ 7.92–7.89 (m, 2H, 2 × Ar–H), 7.65–7.61 (m, 1H, Ar–H), 7.54–7.50 (m, 2H, 2 × Ar–H), 7.48 (s, 1H, CCH), 7.35–7.30 (m, 3H, 3 × Ar–H), 7.23–7.20 (m, 2H, 2 × Ar–H), 5.47 (d, *J* = 14.9 Hz, 1H, NC*H*H), 5.43 (d, *J* = 14.9 Hz, 1H, NCH*H*), 5.14 (d, *J* = 12.8 Hz, 1H, OC*H*H), 5.09 (d, *J* = 12.8 Hz, 1H, OCH*H*), 3.25 (s, 3H, SCH_3_); ^13^C{^1^H} NMR (101 MHz, CDCl_3_) δ 158.3 (C=O), 143.3 (Ar–C_q_), 137.8 (*C*CH), 134.4 (Ar–C_q_), 133.9 (Ar–C), 129.5 (2 × Ar–C), 128.9 (2 × Ar–C), 128.5 (Ar–C), 127.9 (2 × Ar–C), 127.1 (2 × Ar–C), 123.4 (C*C*H), 59.2 (OCH_2_), 53.9 (NCH_2_), 44.3 (SCH_3_); HRMS (ESI-TOF) *m*/*z*: Calcd. for C_18_H_19_N_4_O_3_S [M + H]^+^: 371.1178, found: 371.1183.

#### (1-(13-Oxo-17-((3aR,4R,6aS)-2-oxohexahydro-1H-thieno[3,4-*d*]imidazol-4-yl)-3,6,9-trioxa-12-azaheptadecyl)-1H-1,2,3-triazol-4-yl)methyl (Methyl(oxo)(phenyl)-λ^6^-sulfaneylidene)carbamate (**19**)

Sulfoximine **2a** (5.3 mg, 0.0225 mmol, 1.0 equiv), biotin-PEG3-azide (12.0 mg, 0.027 mmol, 1.2 equiv), and sodium ascorbate (1.1 mg, 0.0054 mmol, 20 mol %) were added to a microwave vial and sealed. The vial was degassed and backfilled with argon three times. CuSO_4_ anhydrous (2.1 mg, 0.0135 mmol) was dissolved in a mixture of *t*BuOH (750 μL) and H_2_O (375 μL), degassed, and backfilled with argon three times. CuSO_4_ (0.11 mL in *t*BuOH and H_2_O, 0.00135 mmol, 5 mol %) was added to the reaction vial and stirred at rt for 24 h. Purification by flash column chromatography (SiO_2_, 5% MeOH in CH_2_Cl_2_) afforded the biotin-PEG3-triazole **19** as a white gum (14.7 mg, 96%). R_*f*_ = 0.18 (5% MeOH in CH_2_Cl_2_); IR (film)/cm^–1^ 3295, 2922, 2867, 1698 (C=O), 1669 (C=O), 1541, 1449, 1253, 1119, 1090, 977, 893, 740, 687, 546, 512; ^1^H NMR (400 MHz, CDCl_3_) δ 7.99 (d, *J* = 7.6 Hz, 2H, 2 × Ar–H), 7.70 (t, *J* = 7.3 Hz, 1H, Ar–H), 7.62 (t, *J* = 7.5 Hz, 2H, 2 × Ar–H), 6.84 (s, 1H, NH), 6.40 (s, 1H, NH), 5.52 (s, 1H, NH), 5.19 (s, 2H, OCH_2_), 4.54 (s, 3H, 3 × CH), 4.35 (s, 1H, CH), 3.88 (s, 2H, CH_2_), 3.59–3.53 (m, 10H, 5 × CH_2_), 3.41 (s, 2H, CH_2_), 3.35 (s, 3H, SCH_3_), 2.21 (s, 2H, CH_2_), 1.67 (s, 4H, 2 × CH_2_), 1.43 (s, 2H, CH_2_); ^13^C{^1^H} NMR (101 MHz, CDCl_3_) δ 173.4 (C=O), 158.6 (C=O), 138.0 (Ar–C_q_), 134.1 (Ar–C), 129.8 (2 × Ar–C), 127.4 (2 × Ar–C), 77.2 (SCH), 70.5 (OCH_2_), 70.4 (OCH_2_), 70.3 (OCH_2_), 70.0 (OCH_2_), 69.9 (OCH_2_), 69.3 (OCH_2_), 61.8 (CONH*C*H), 60.3 (CONH*C*H), 59.3 (OCH_2_), 55.5 (NCH_2_), 50.5 (NCH_2_), 44.5 (SCH_3_), 39.1 (CH_2_), 35.9 (CH_2_), 28.2 (2 × CH_2_), 25.6 (CH_2_); HRMS (ESI-TOF) *m*/*z*: Calcd. for C_29_H_44_N_7_O_8_S_2_ [M + H]^+^: 682.2693, found: 682.2711. Note: Broadening of the ^1^H and ^13^C NMR signals of the triazole ring (C and CH signals) occurred to an extent that these were not visible. Similarly the ^13^C NMR signal for the biotin urea carbonyl (HN-CO-NH) is not observed.

## Notes

All characterization data for the synthesized compounds can be found at https://doi.org/10.14469/hpc/11861.

## Supporting Information Available

Details of DOE investigation. Copies of ^1^H and ^13^C NMR spectra. (PDF)
