# Supplementary material for: Synthesis of Sulfoximine Propargyl Carbamates under Improved Conditions for Rhodium Catalyzed Carbamate Transfer to Sulfoxides
Source: J Org Chem. 2022 Nov 15;87(23):16115–26. doi: 10.1021/acs.joc.2c02083 (PMC9724092; doi:10.1021/acs.joc.2c02083)
Supplement: Supplementary file 1 — jo2c02083_si_001.pdf [file jo2c02083_si_001.pdf]

SUPPORTING INFORMATION

Synthesis of Sulfoximine Propargyl Carbamates under Improved Conditions for Rhodium Catalyzed Carbamate Transfer to Sulfoxides

Zhenhao Zhong, Julian Chesti, Alan Armstrong,* James A. Bull*

Department of Chemistry, Imperial College London, Molecular Sciences Research Hub, White City Campus, Wood Lane, London W12 0BZ, UK.

*E-mail: j.bull@imperial.ac.uk; a.armstrong@imperial.ac.uk

DoE study for the optimisation of the Rh2(esp)2-catalysed propargyl carbamate transfer.....S2-S4

1H and 13C NMR spectra..S5-S46

H2N-Carbamates……………..S6-S7

Sulfoximine carbamates 2a–2x and 4–9…………...S8-S37

Sulfilimines 12−16 and sulfinamidine 17……...S38-S43

Triazoles 18 and 19 ....S39-S46

HPLC data of enantioenriched compound..S47

1H NMR spectra of other starting materials...S48-S57

DoE study for the optimisation of the $Rh_2(esp)_2$-catalysed propargyl carbamate transfer

A Design of Experiment (DoE) study was conducted to investigate how the yield was affected by multiple variables. This was achieved by carrying out a set of reactions with multiple variable parameters followed by establishing a predicted response surface as a function to demonstrate the correlation between the desired response (e.g. yield) and multiple variables simultaneously.

JMP Pro 14 was used to design experiments with multiple variable factors, analysing experimental results and predicting models.

Setting the yield of the propargyl carbamate sulfoximine **2a** as the desired response to be optimised, four factors including equivalents of oxidant, equivalents of carbamate, temperature and catalyst loading were considered as the main factors affecting the yield. To reduce the number of variables and improve the model accuracy, equivalents of oxidant and equivalents of carbamate were combined to a single factor (equivalents of oxidant and carbamate) in the factor model. Factor ranges were considered and set by either extending the value from the previous study (equivalents of oxidant and carbamate and temperature) or by reducing the cost of catalyst (catalyst loading). The factors and settings were summarised as shown in **Table S1**.

Table S1. Summary of factors and settings for DoE study

Factor	Factor Type	Factor Range	Difference in Value	Desired Response	Desired Response Value
Equivalents of oxidant and carbamate (equiv)	Continuous	1.25–2.0	0.25	Maximised Yield (%)	100%
Catalyst loading (mol%)	Continuous	1.5–2.5	0.5		
Temperature (°C)	Continuous	25–55	15		

According to the settings shown in **Table S1**, 26 default experiments with specific factor values were generated from JMP. The experimental outcomes were manually imported into the software followed by an analysis of the correlation between the yield of propargyl carbamate sulfoximine

2a and the multiple factors investigated. A fit between the experimental conditions and the actual yields was generated (**Figure S1**, left), where the black dots showed the actual yields generated under specific reaction conditions and the red line indicated the predictive model (R^2 = 0.85). Additionally, the correlation showing how significantly the predicted yield could be affected by factors were generated (**Figure S1**, right), where 2nd order interactions were taken into consideration to understand how multiple factors could affect the predicted yield jointly. According to the PValue shown, temperature was the most impactful factor to affect the predicted yield. Though the 1st order of the oxidant and carbamate equivalents showed weak influence on the predicted yield (LogWorth = 0.194), the 2nd order interaction of oxidant and carbamate equivalents showed significant influence on the predicted yield (LogWorth = 2.229). This indicated that changing the oxidant and carbamate equivalents from 1.25 equivalents to 2.0 equivalents would not affect the predicted yield as significantly as the other two factors, but maintaining high loading of the reagents would help to push the reaction to completion.

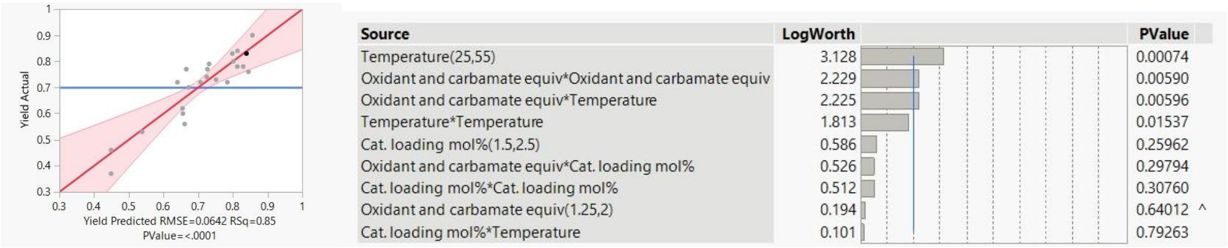

Figure S1. Fit of actual yields (black dots) to the predicted yield plot (red line).

A prediction profiler was then generated from JMP Pro 14, where the predicted yield has been modelled as a function against different factors (**Figure S2**). The example below showed the optimised reaction conditions where the modifier was positioned at the highest predicted yield by maximising the desirability. These values were then adjusted slightly and the test reaction was performed with 1.7 equivalents of oxidant and carbamate, 2.0 mol% catalyst at 30 °C, which generated excellent *in situ* yield (90%) and isolated yield (85%) of **2a**, which were highly correlated with the highest predicted yield (87%) achieved at the dome of the prediction profiler, showing excellent agreement between the predicted model and the reaction outcome. Considering that the saddle point was achieved in prediction profiler, any significant shift of the values would result in a decrease in yield. Therefore, the reaction conditions employed in the test reaction was maintained for further reactions.

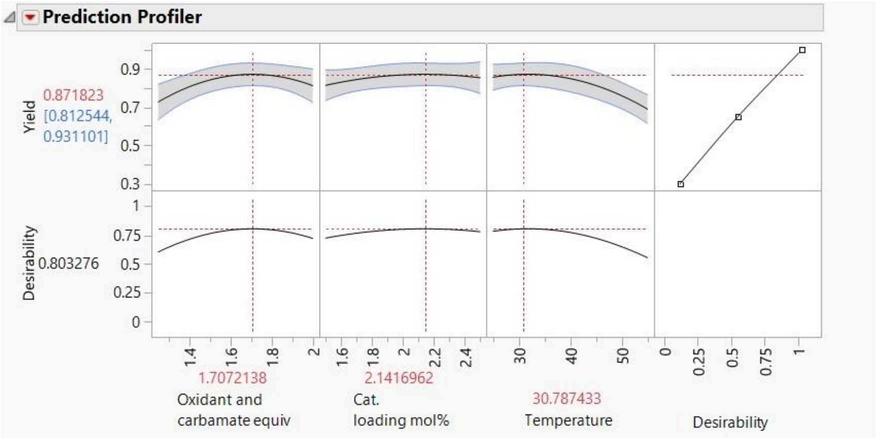

Figure S2. Prediction profiler showing the change of the predicted yield with single factors changing continuously. The cross point of the red dot lines is placed at the point where the desirability is maximized, showing the highest predicted yield was 87% approximately. The error of the predicted yield is shown as the grey area in the profiler.

^{1}H and ^{13}C NMR spectra

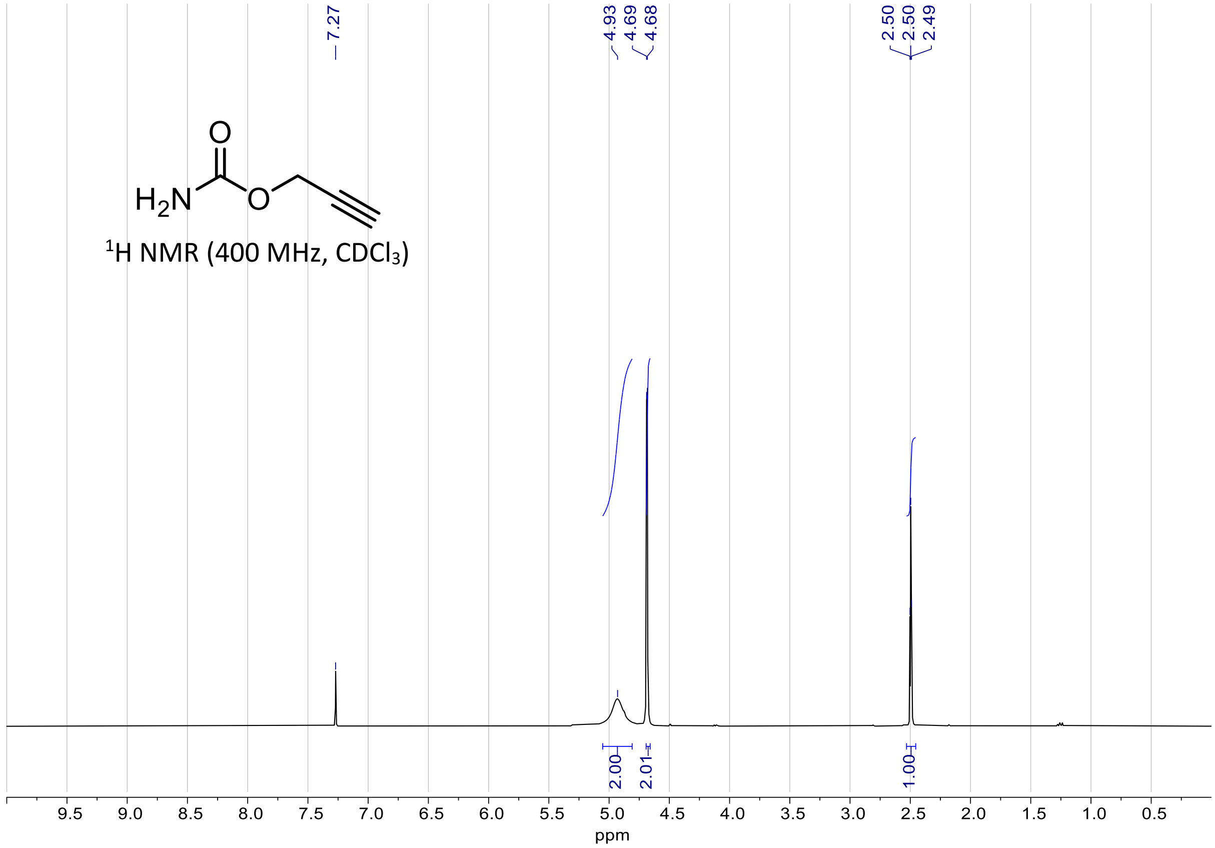
7.27
4.93
4.69
4.68
2.50
2.50
2.49
H2N
O
O
1H NMR (400 MHz, CDCl3)
2.00
2.01
1.00
9.5 9.0 8.5 8.0 7.5 7.0 6.5 6.0 5.5 5.0 4.5 4.0 3.5 3.0 2.5 2.0 1.5 1.0 0.5
ppm

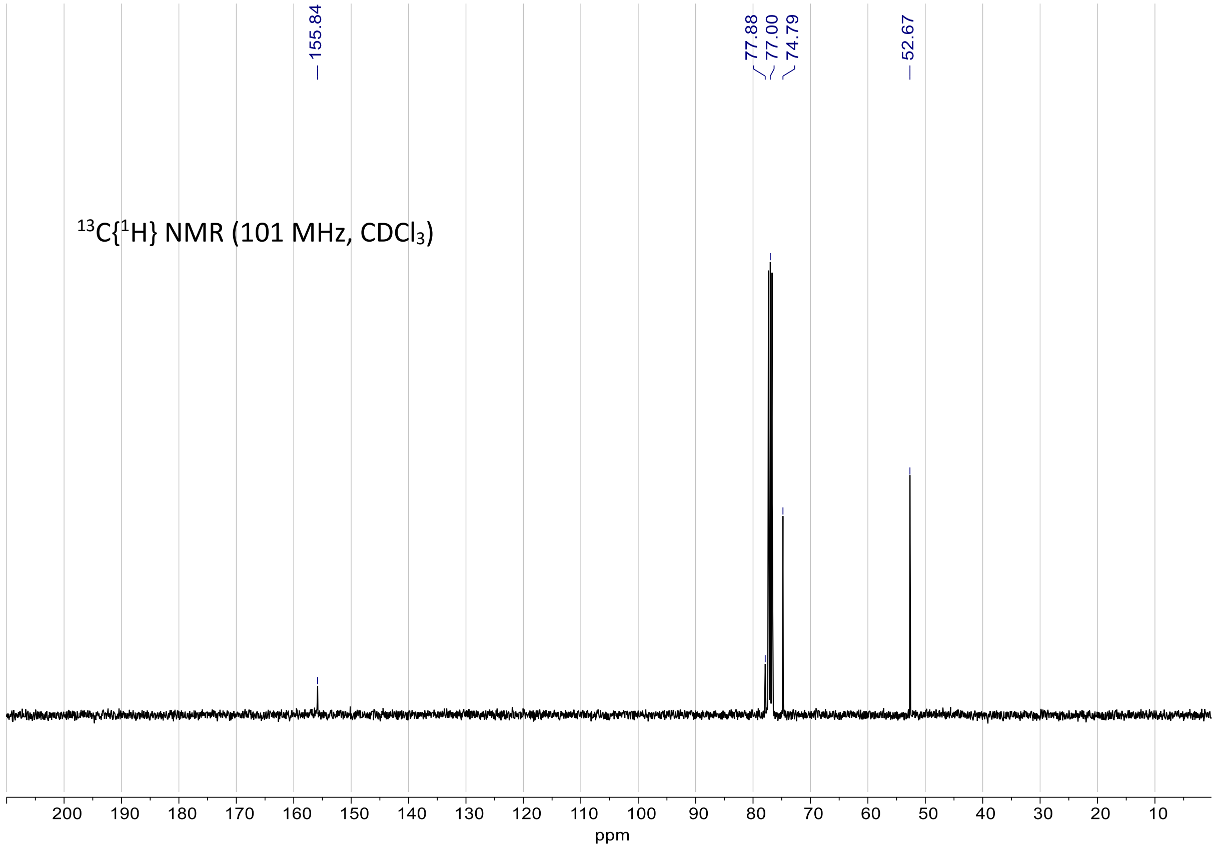
155.84
77.88
77.00
74.79
52.67
13C{1H} NMR (101 MHz, CDCl3)
200 190 180 170 160 150 140 130 120 110 100 90 80 70 60 50 40 30 20 10
ppm

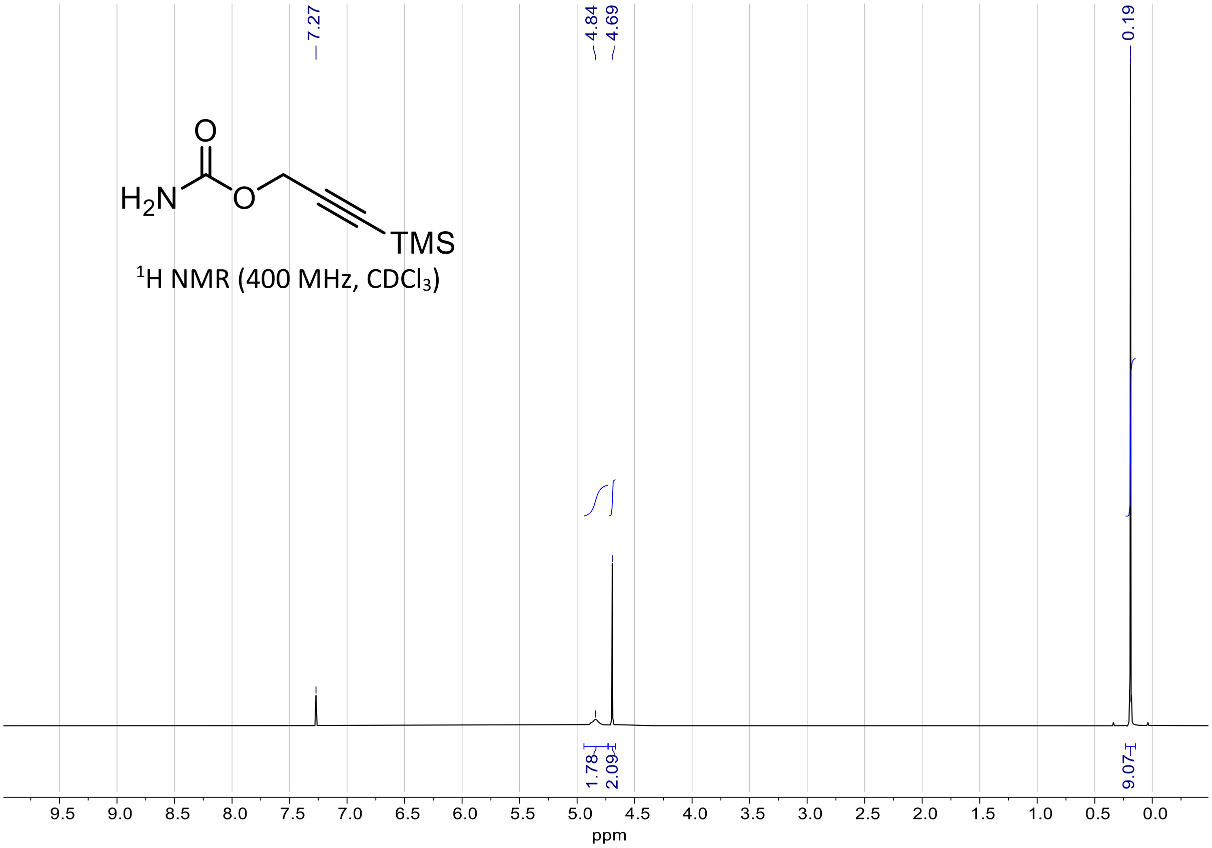

^1H NMR (400 MHz, $CDCl_3$)

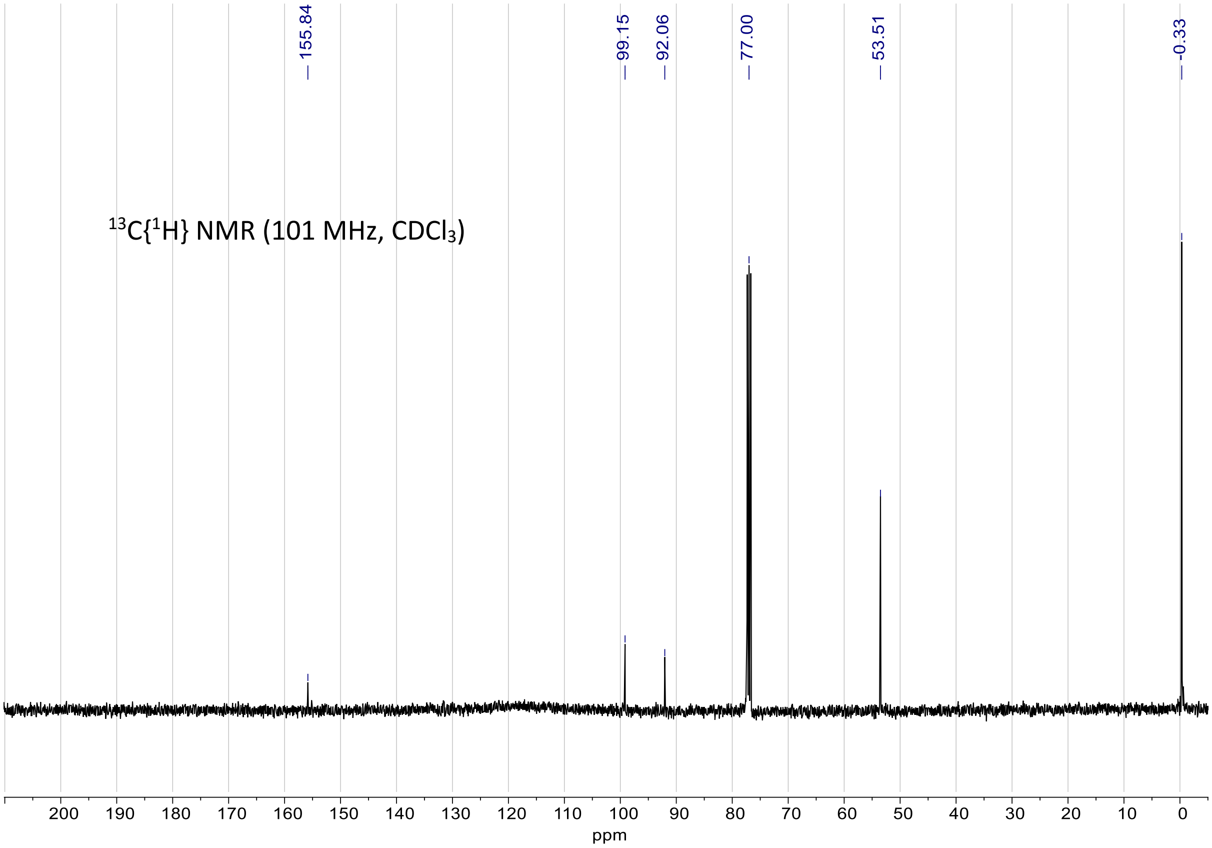

$^{13}C\{^1H\}$ NMR (101 MHz, $CDCl_3$)

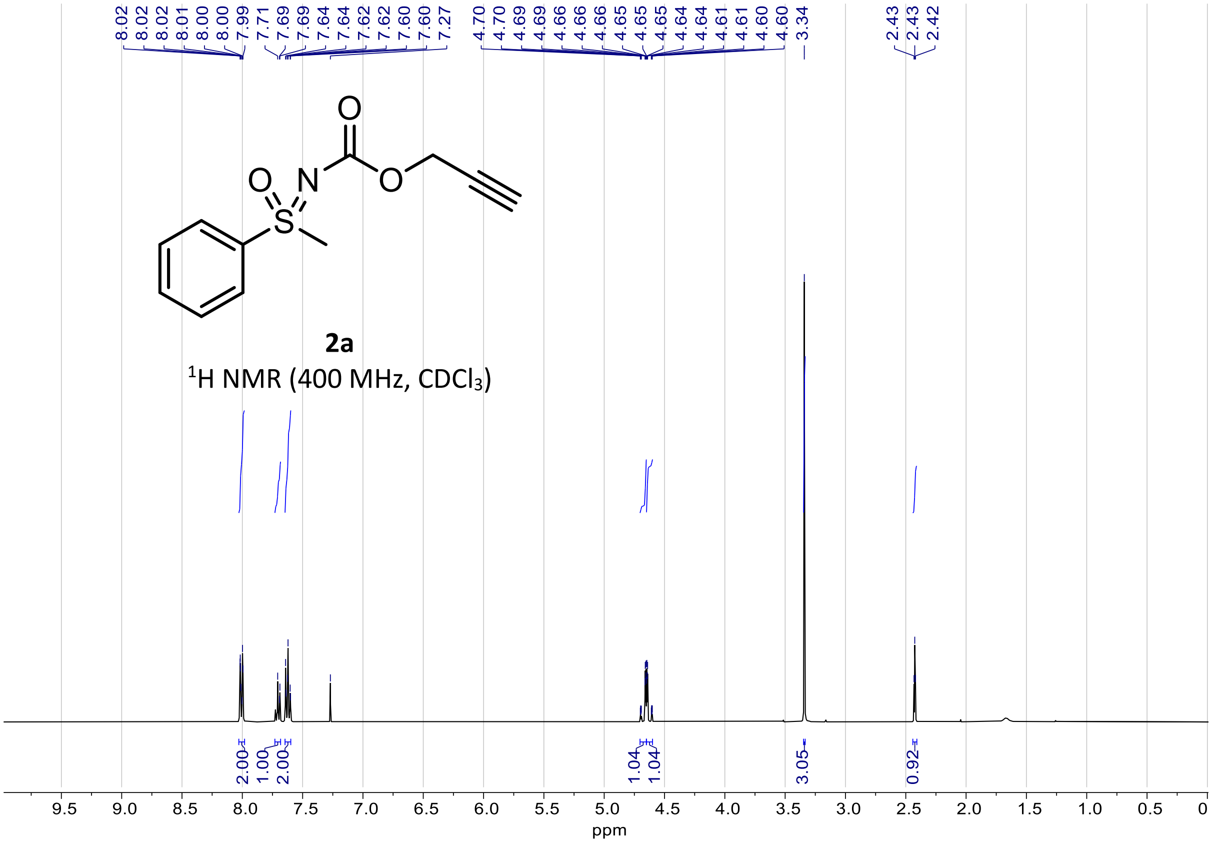

2a

^{1}H NMR (400 MHz, $CDCl_3$)

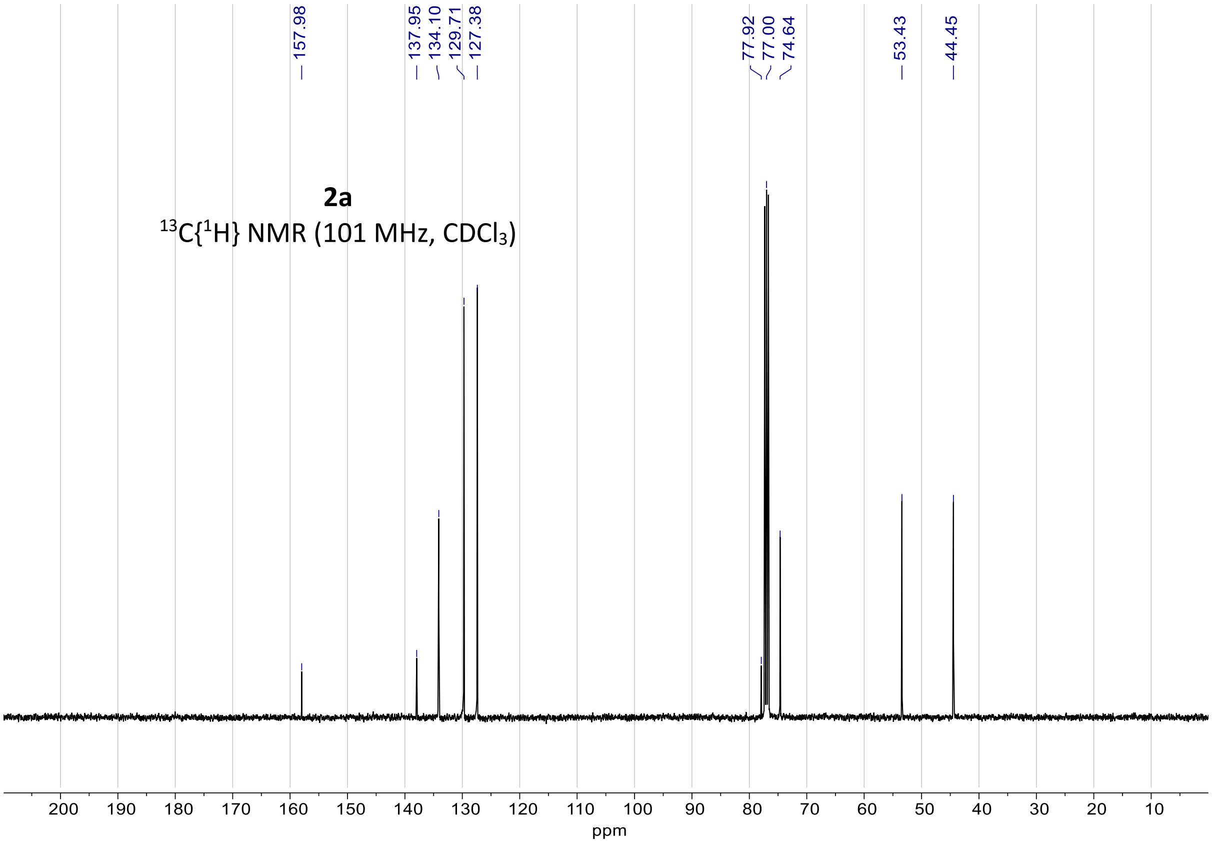

2a

$^{13}C\{^{1}H\}$ NMR (101 MHz, $CDCl_3$)

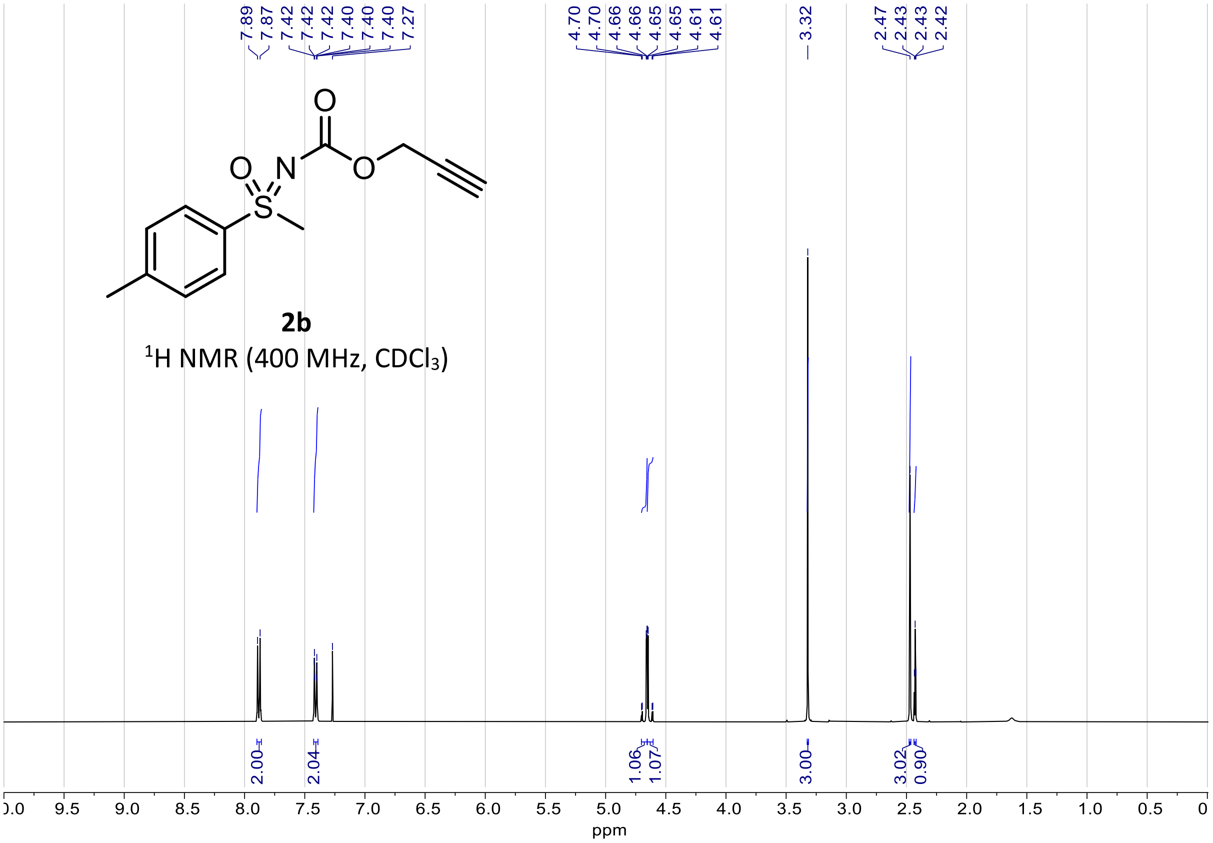

2b

^{1}H NMR (400 MHz, $CDCl_3$)

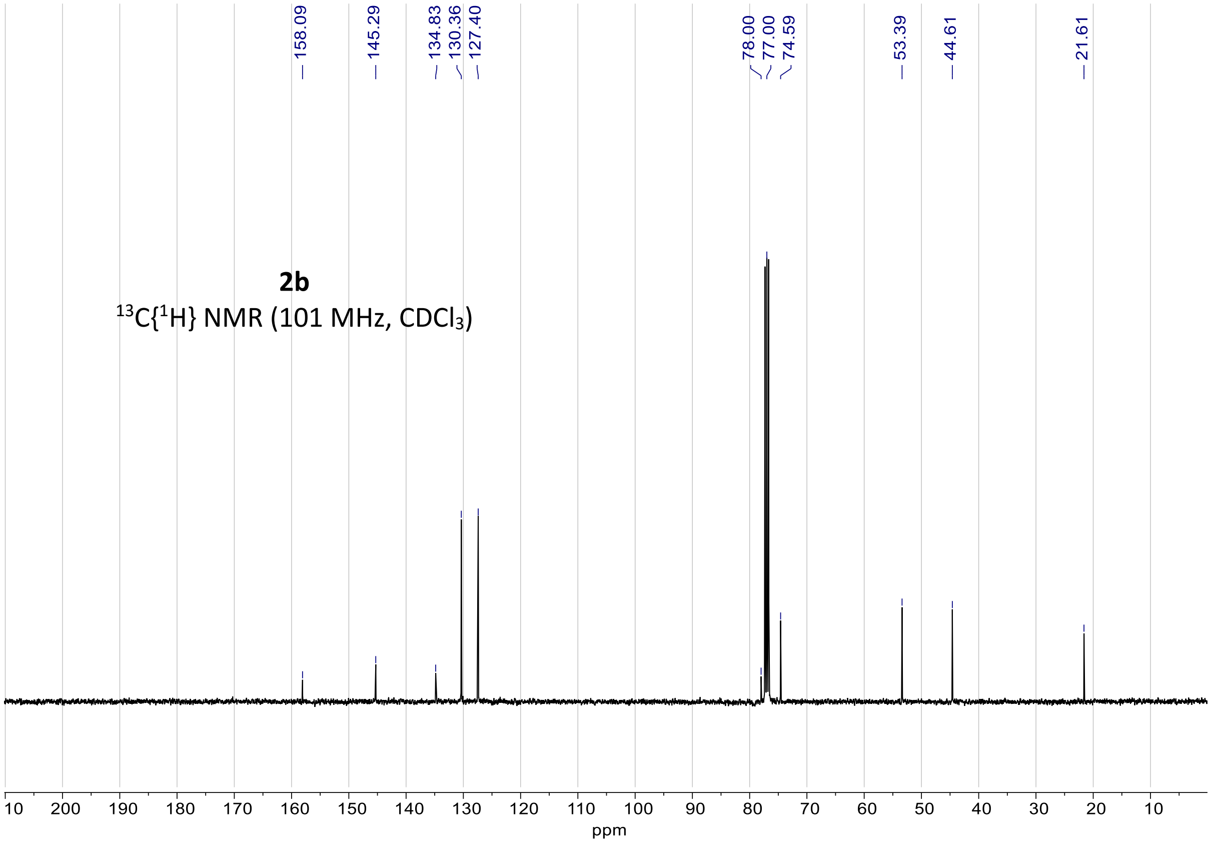

2b

$^{13}C\{^{1}H\}$ NMR (101 MHz, $CDCl_3$)

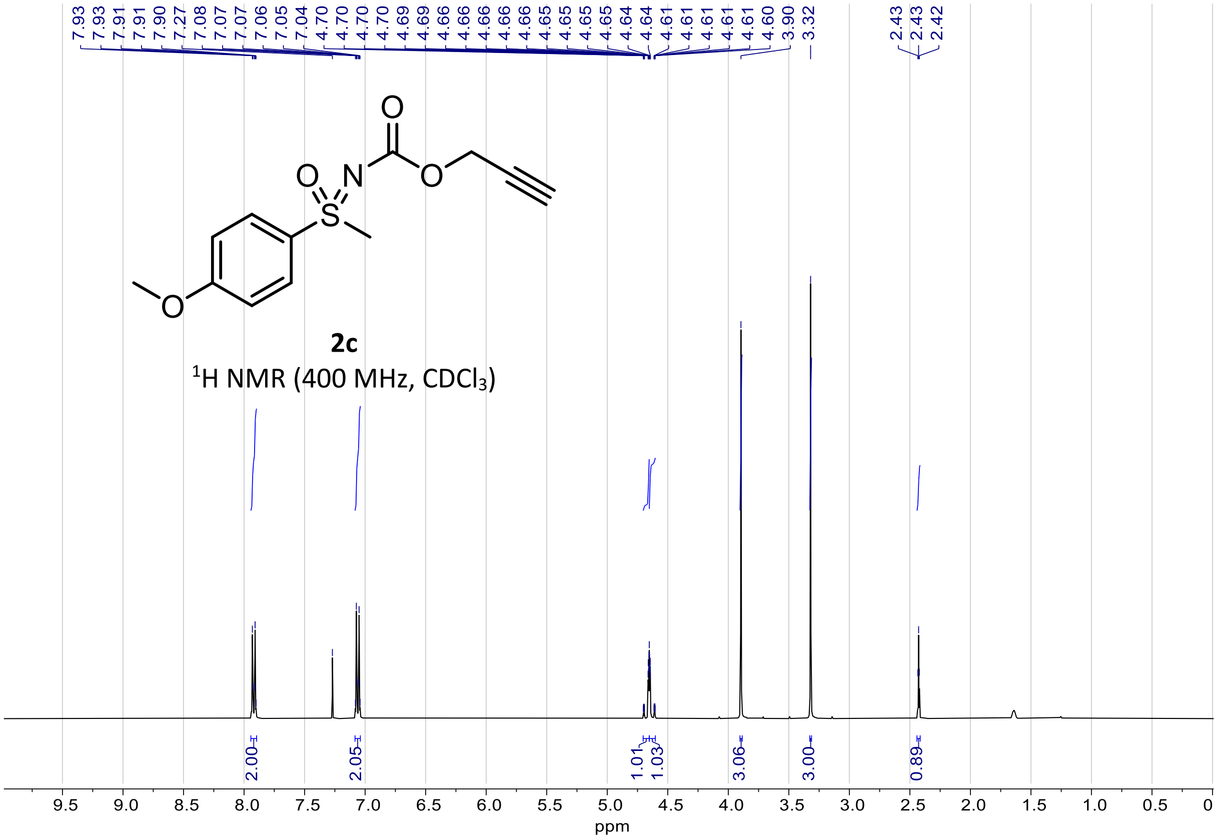
7.93 7.93 7.91 7.91 7.90 7.27 7.08 7.07 7.07 7.06 7.05 7.04 4.70 4.70 4.70 4.69 4.69 4.66 4.66 4.66 4.66 4.66 4.65 4.65 4.65 4.64 4.64 4.61 4.61 4.61 4.61 4.61 4.60 3.90 3.32
2.43 2.43 2.42
O
N
O
O
S
O
2c
^{1}H NMR (400 MHz, $CDCl_3$)
2.00 2.05 1.01 1.03 3.06 3.00 0.89
9.5 9.0 8.5 8.0 7.5 7.0 6.5 6.0 5.5 5.0 4.5 4.0 3.5 3.0 2.5 2.0 1.5 1.0 0.5 0
ppm

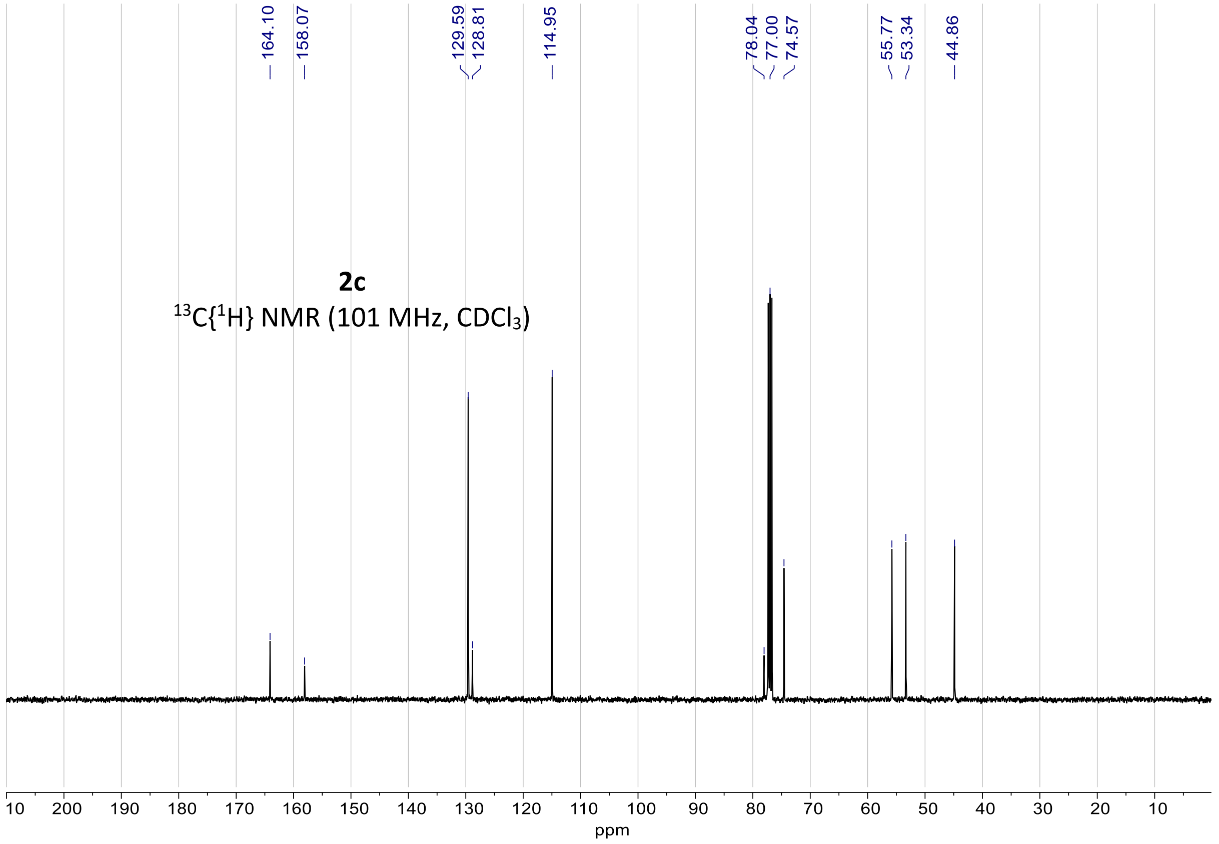
164.10 158.07 129.59 128.81 114.95 78.04 77.00 74.57 55.77 53.34 44.86
2c
$^{13}C\{^1H\}$ NMR (101 MHz, $CDCl_3$)
10 200 190 180 170 160 150 140 130 120 110 100 90 80 70 60 50 40 30 20 10
ppm

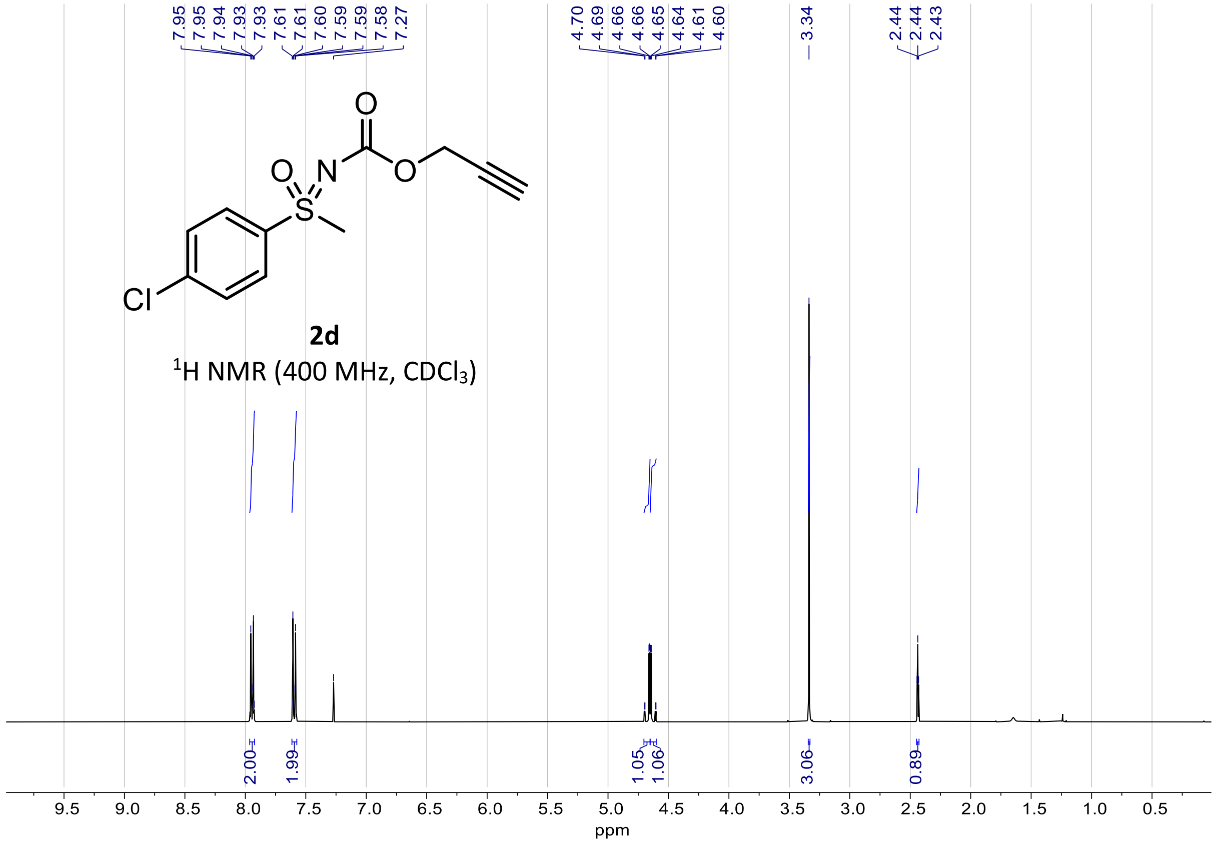
7.95 7.95 7.94 7.93 7.93 7.61 7.61 7.60 7.59 7.59 7.58 7.27
4.70 4.69 4.66 4.66 4.65 4.64 4.61 4.60
3.34
2.44 2.44 2.43
O
N
O
O
S
Cl
2d
^{1}H NMR (400 MHz, CDCl$_3$)
2.00
1.99
1.05
1.06
3.06
0.89
9.5 9.0 8.5 8.0 7.5 7.0 6.5 6.0 5.5 5.0 4.5 4.0 3.5 3.0 2.5 2.0 1.5 1.0 0.5
ppm

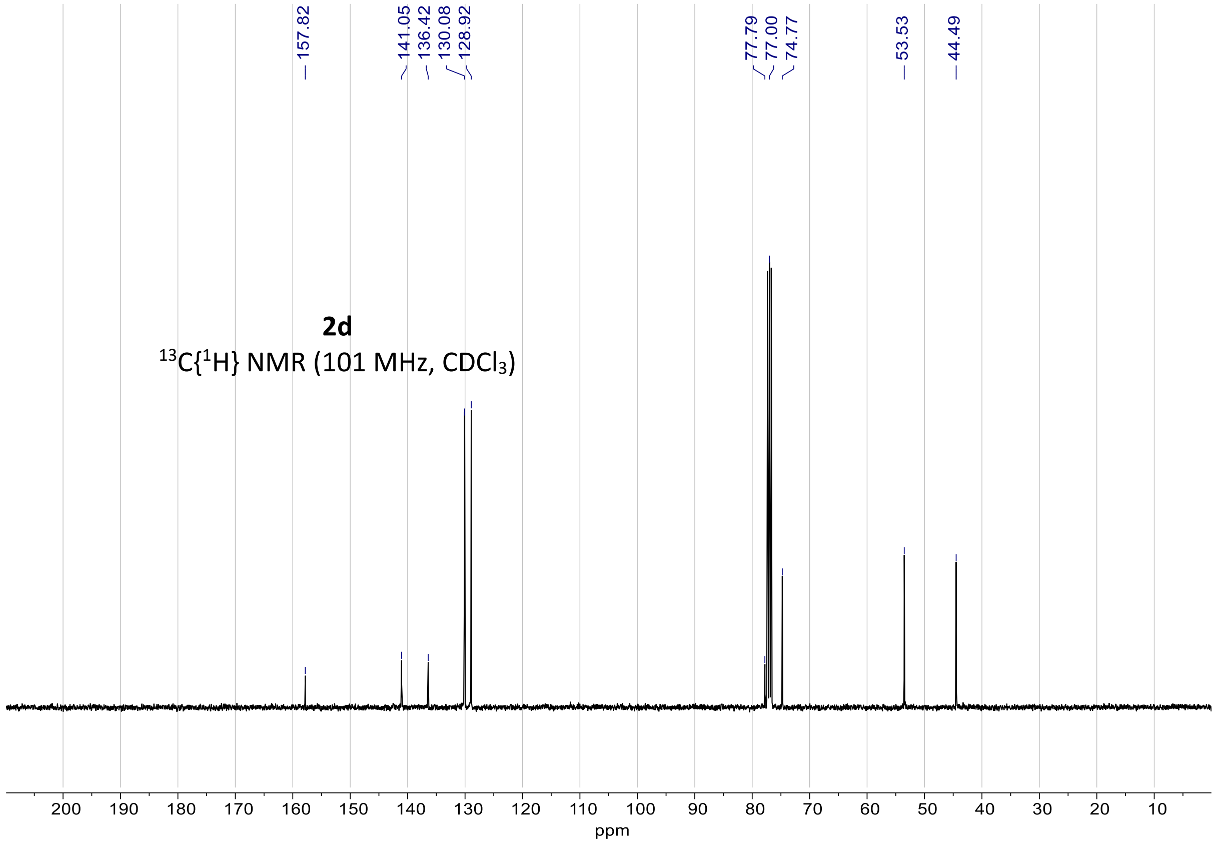
157.82
141.05 136.42 130.08 128.92
77.79 77.00 74.77
53.53
44.49
2d
^{13}C{^{1}H} NMR (101 MHz, CDCl$_3$)
200 190 180 170 160 150 140 130 120 110 100 90 80 70 60 50 40 30 20 10
ppm

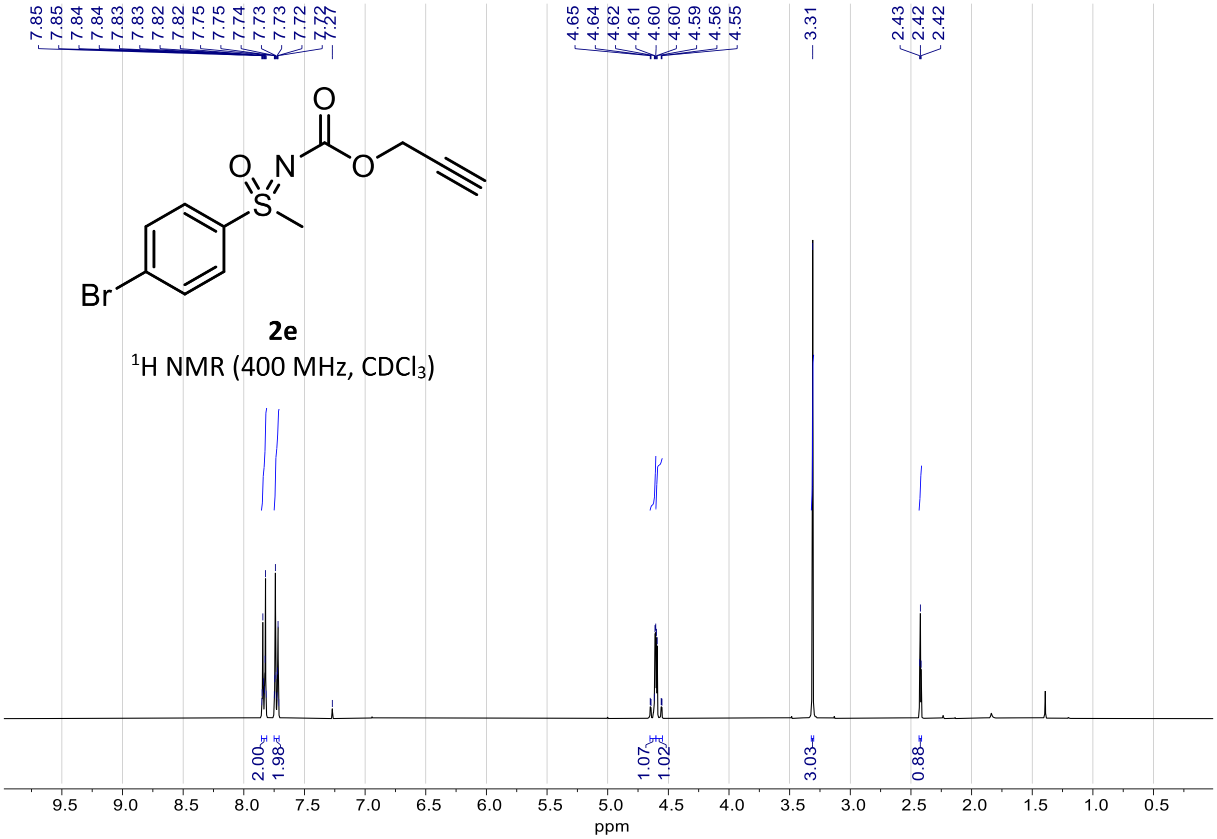
7.85
7.85
7.84
7.84
7.83
7.83
7.82
7.82
7.75
7.75
7.74
7.73
7.73
7.72
7.72
7.27
4.65
4.64
4.62
4.61
4.60
4.60
4.59
4.56
4.55
3.31
2.43
2.42
2.42
O
N
O
O
S
O
Br
2e
^{1}H NMR (400 MHz, CDCl$_3$)
2.00
1.98
1.07
1.02
3.03
0.88
9.5 9.0 8.5 8.0 7.5 7.0 6.5 6.0 5.5 5.0 4.5 4.0 3.5 3.0 2.5 2.0 1.5 1.0 0.5
ppm

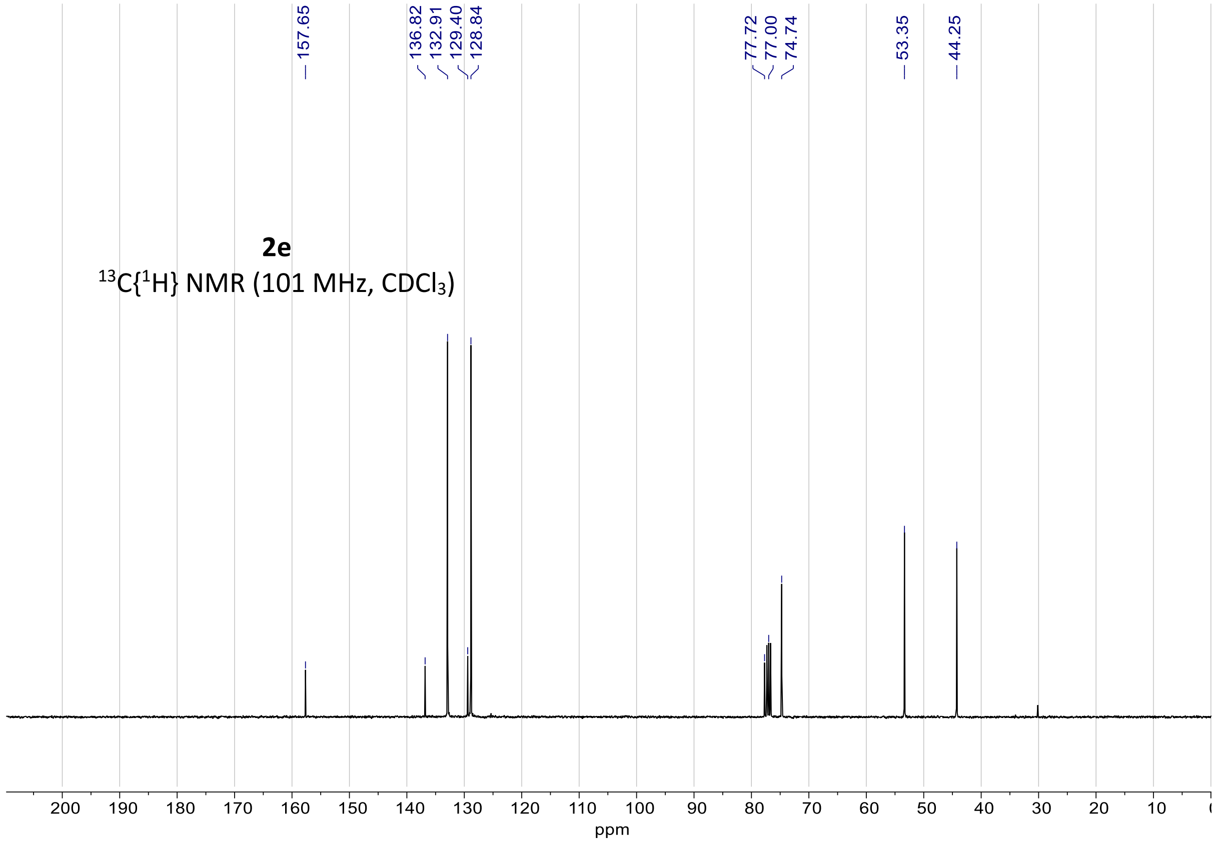
157.65
136.82
132.91
129.40
128.84
77.72
77.00
74.74
53.35
44.25
2e
^{13}C{^{1}H} NMR (101 MHz, CDCl$_3$)
200 190 180 170 160 150 140 130 120 110 100 90 80 70 60 50 40 30 20 10
ppm

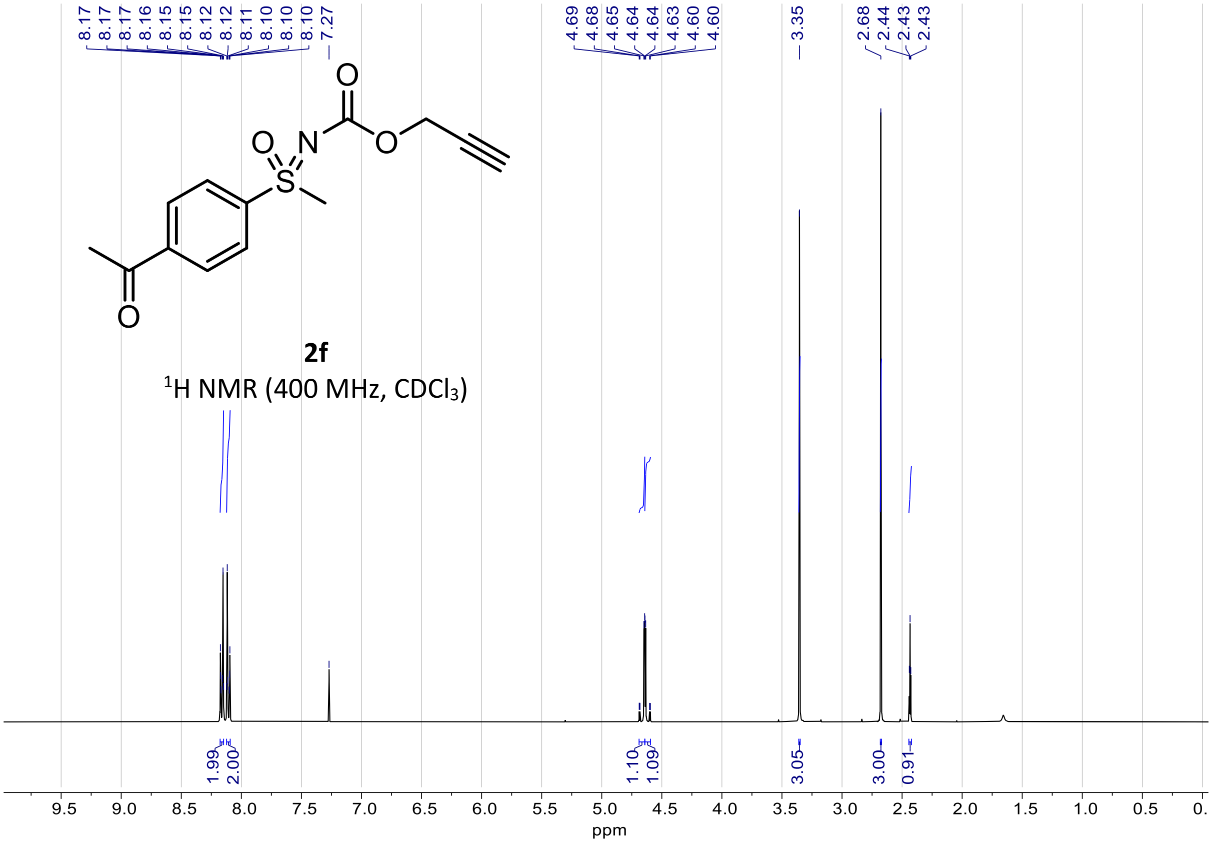

2f

^{1}H NMR (400 MHz, $CDCl_3$)

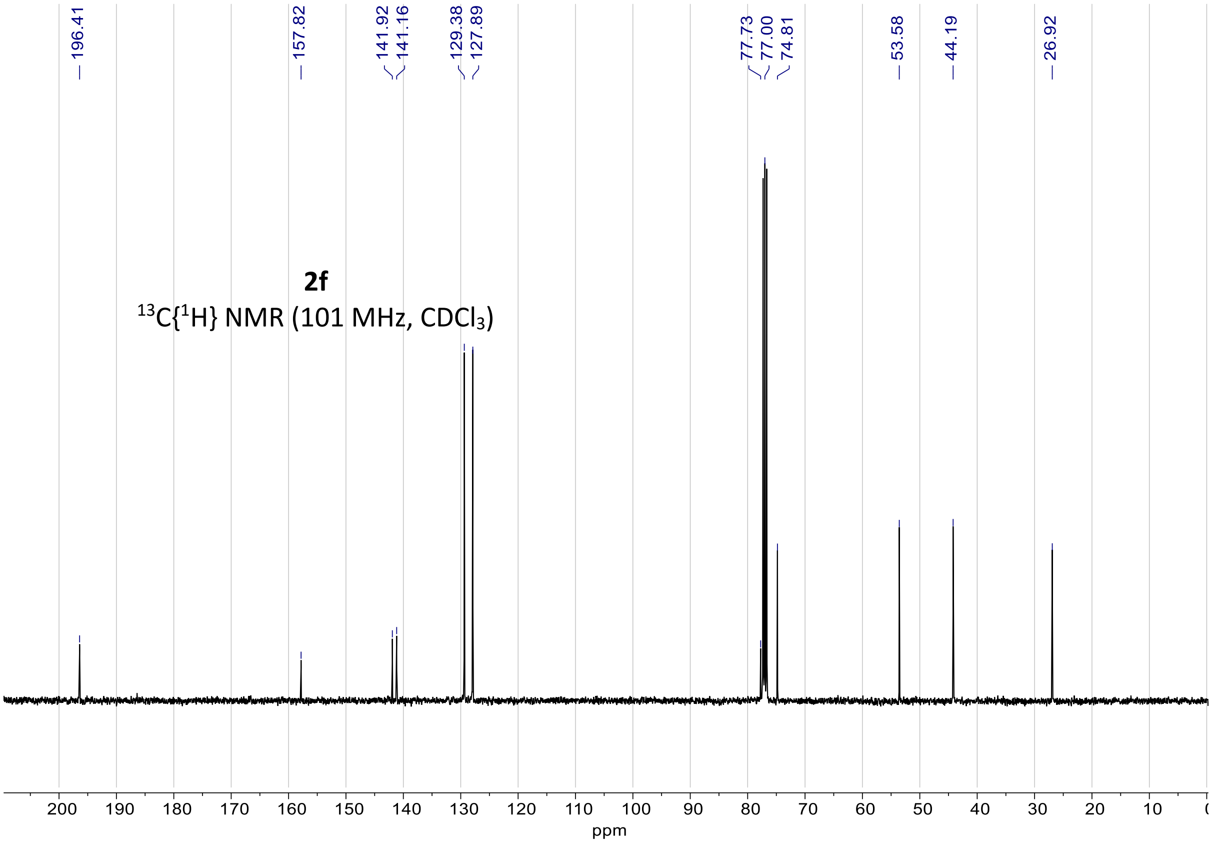

2f

$^{13}C\{^{1}H\}$ NMR (101 MHz, $CDCl_3$)

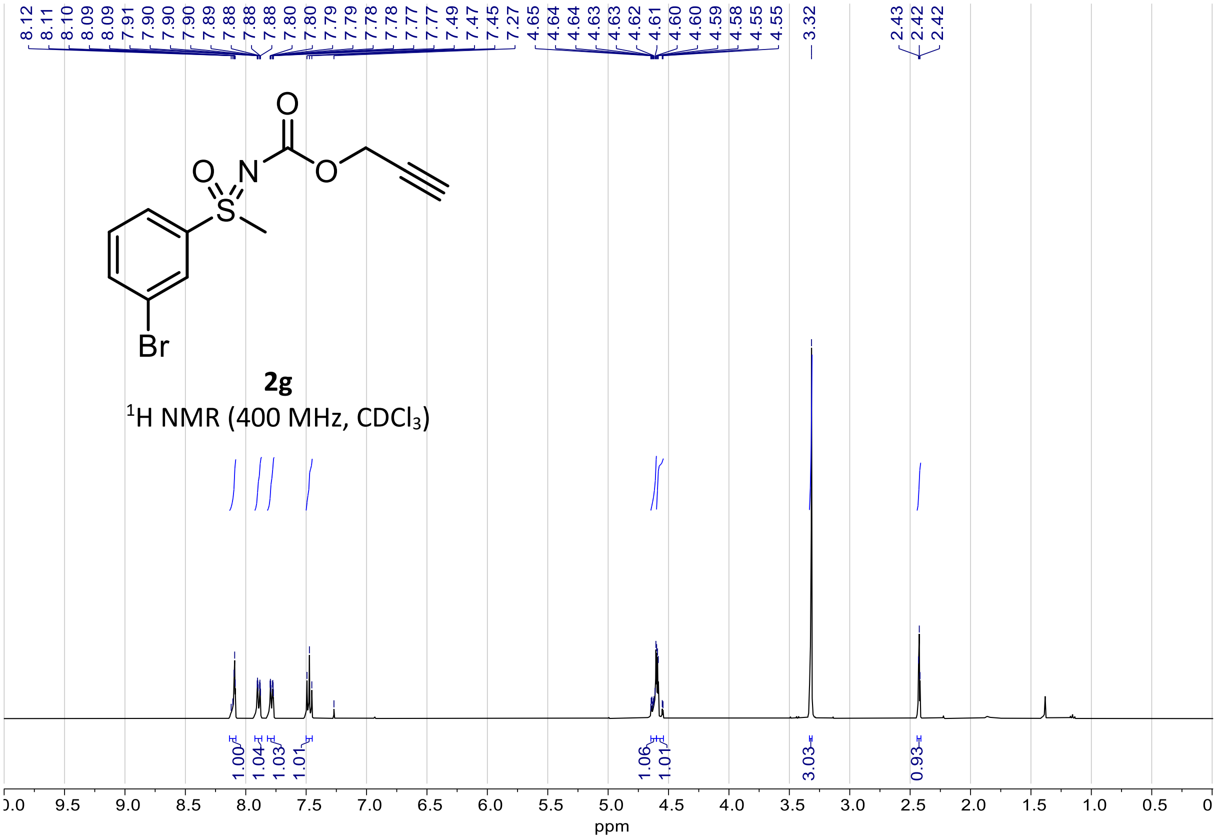

2g

^{1}H NMR (400 MHz, $CDCl_3$)

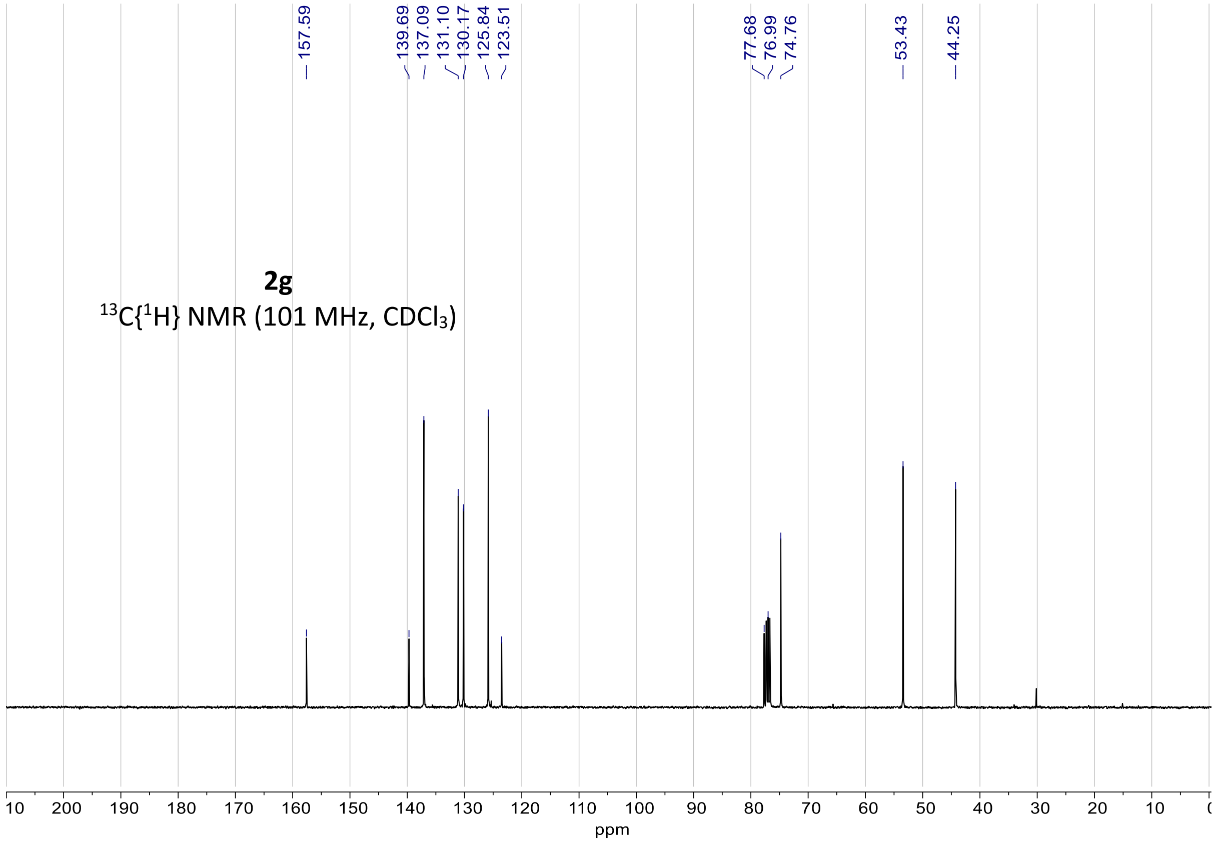

2g

^{13}C{^{1}H} NMR (101 MHz, $CDCl_3$)

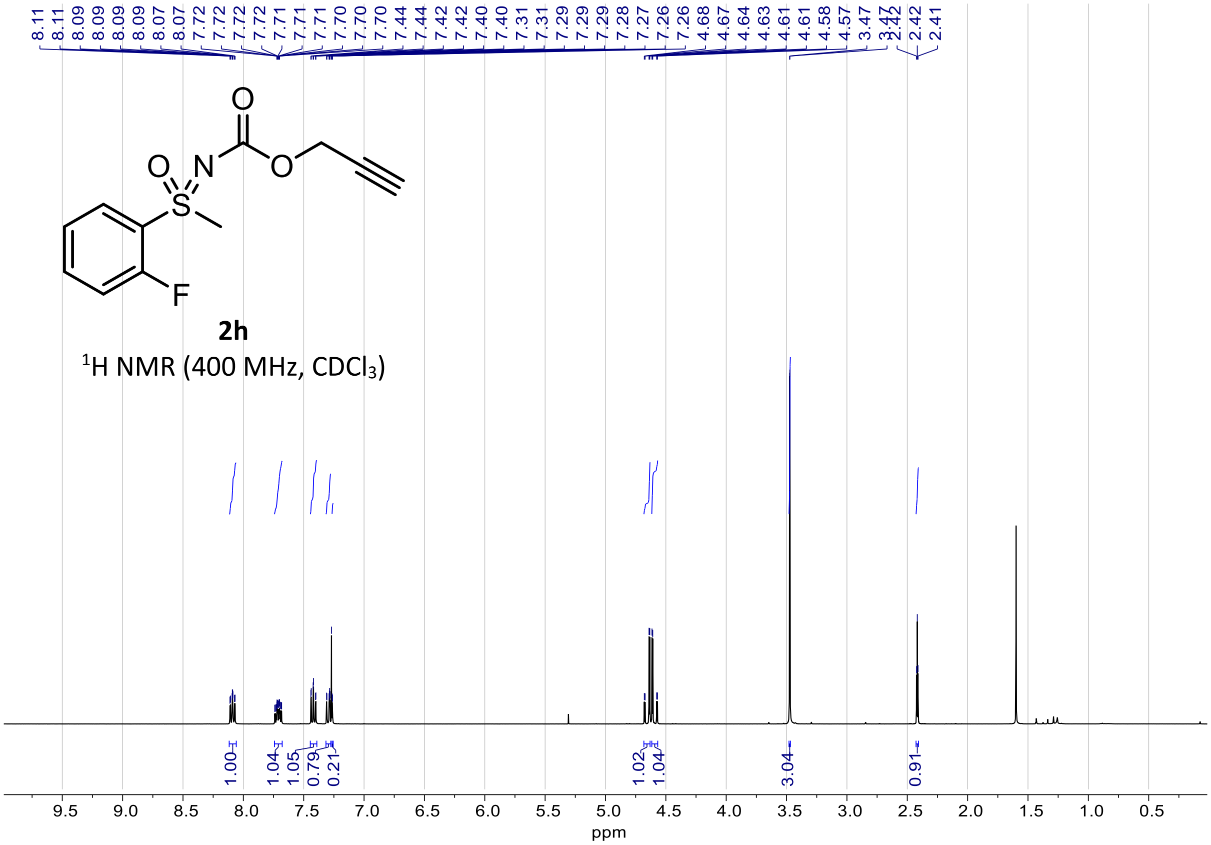

2h

^{1}H NMR (400 MHz, $CDCl_3$)

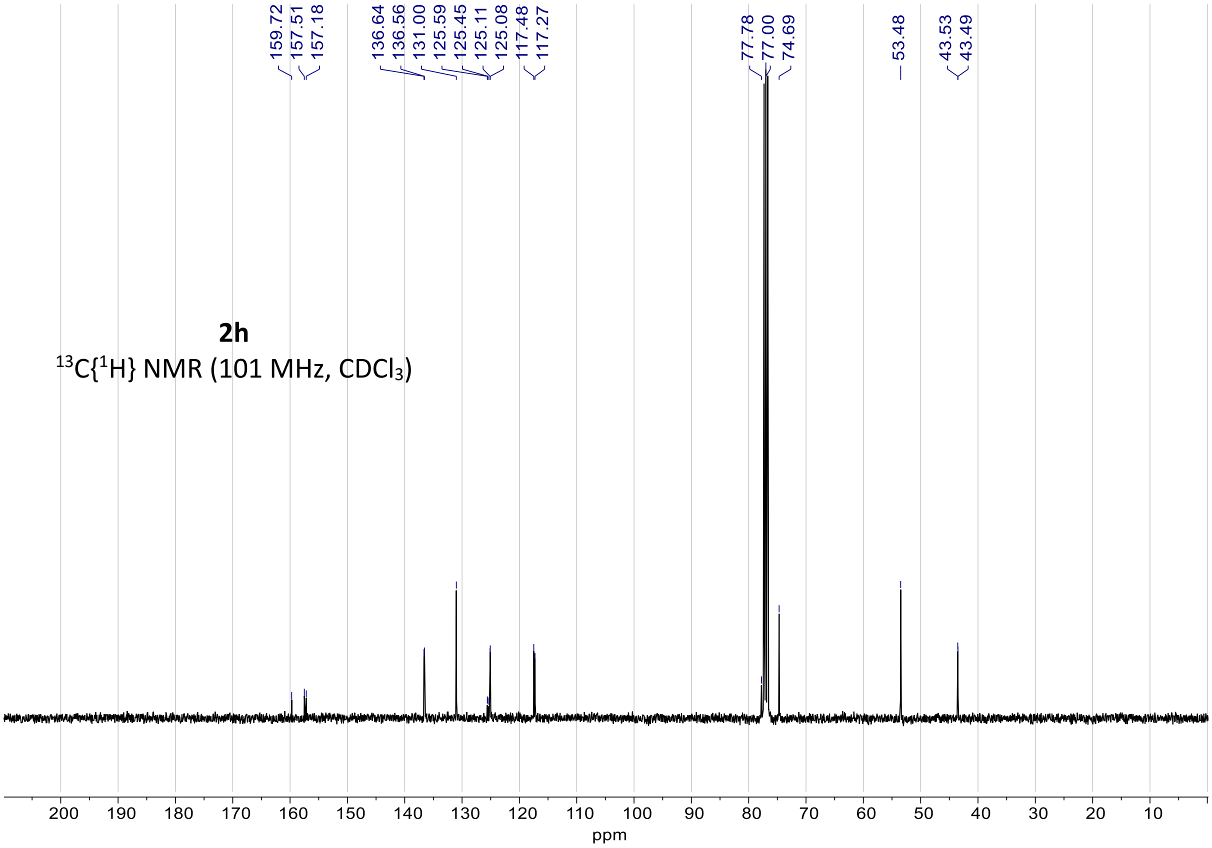

2h

$^{13}C\{^{1}H\}$ NMR (101 MHz, $CDCl_3$)

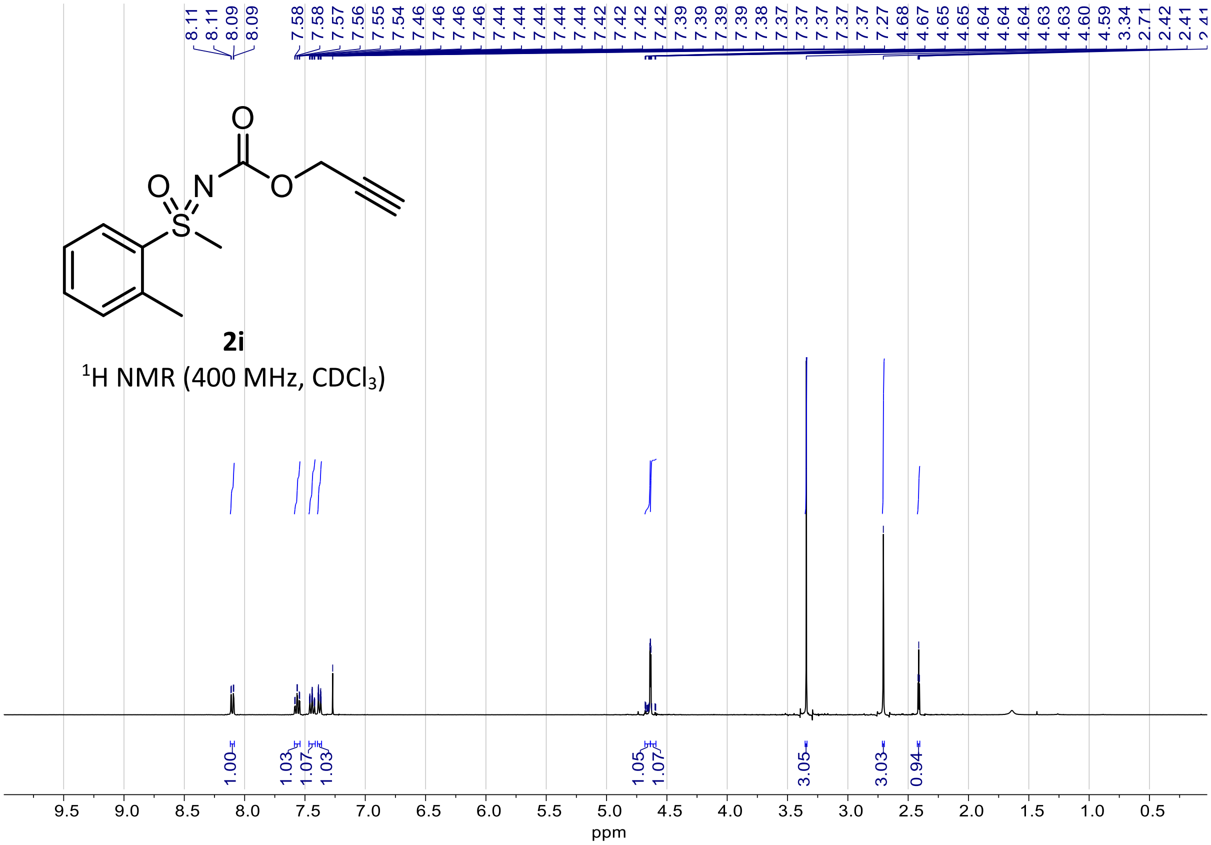

2i

^{1}H NMR (400 MHz, $CDCl_3$)

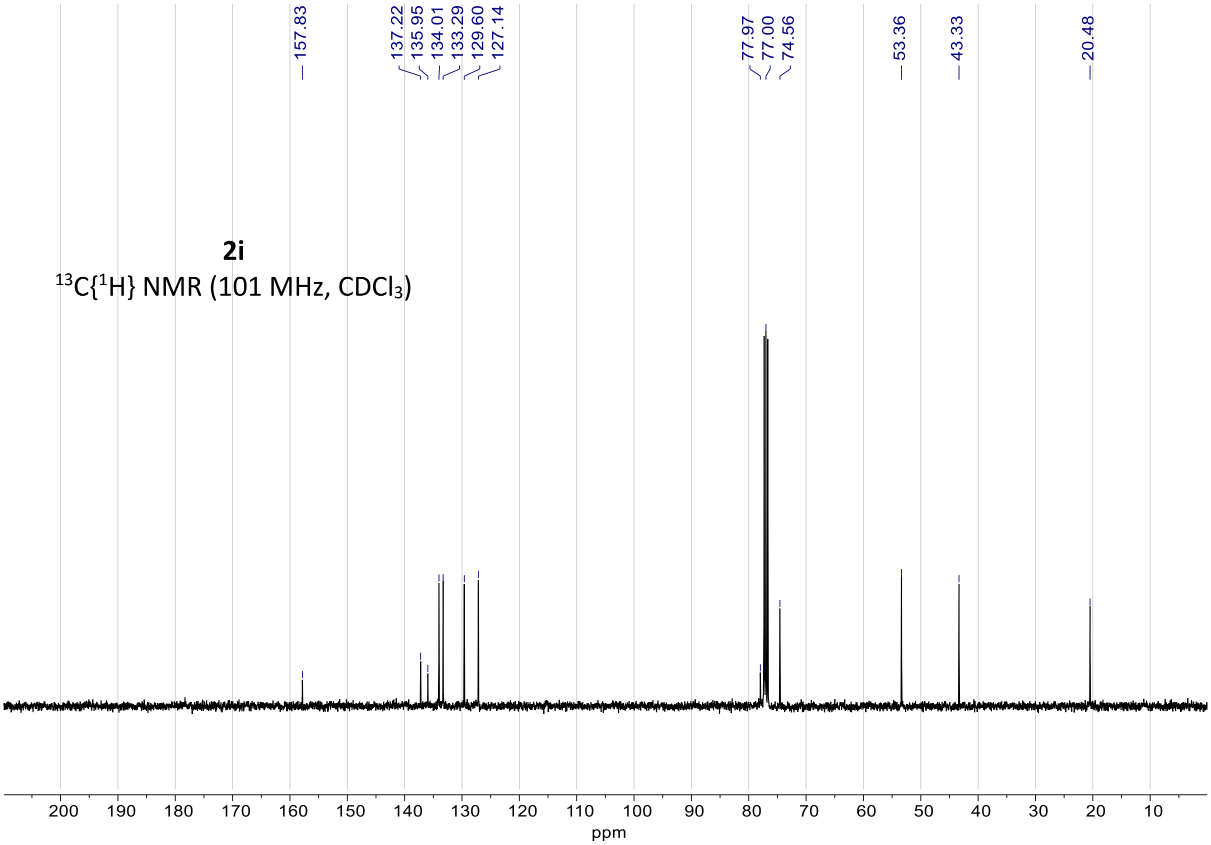

2i

$^{13}C\{^{1}H\}$ NMR (101 MHz, $CDCl_3$)

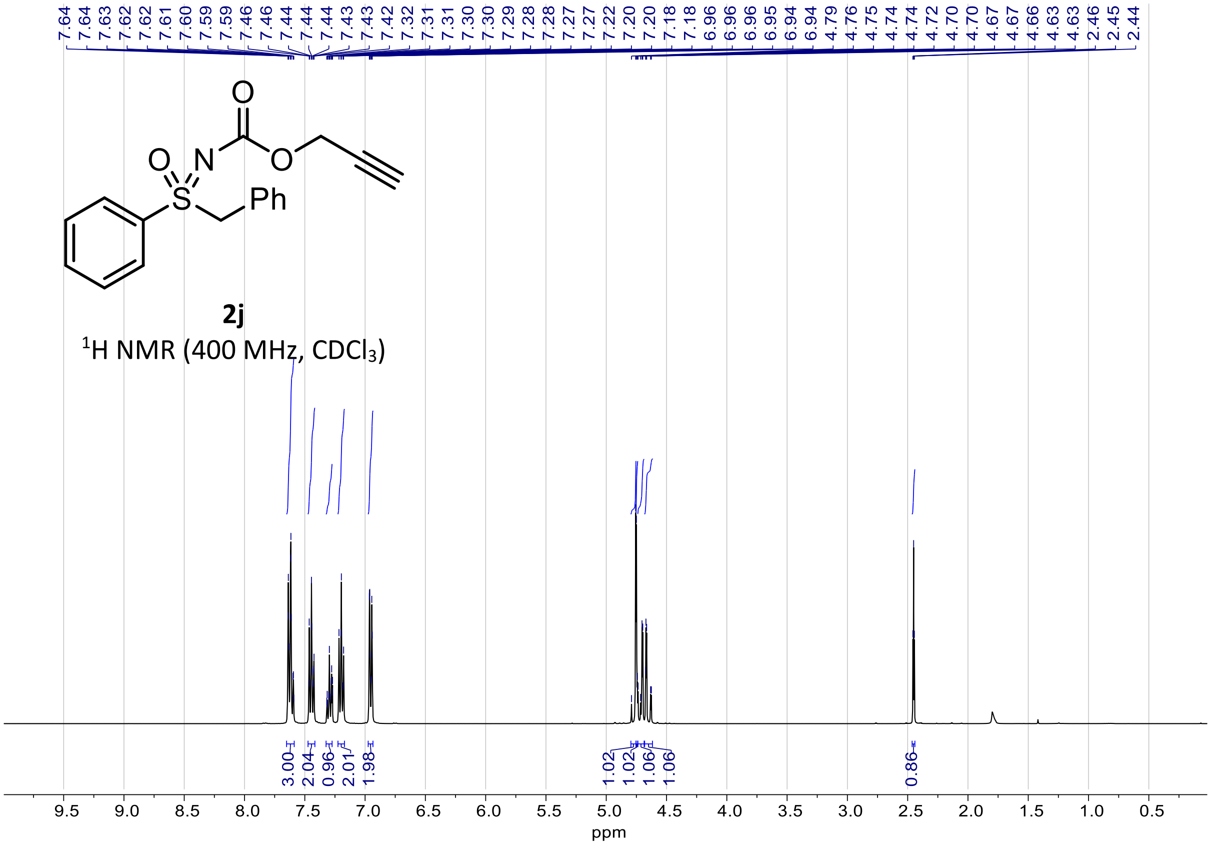

7.64 7.64 7.63 7.62 7.62 7.61 7.60 7.59 7.59 7.46 7.44 7.44 7.44 7.43 7.43 7.42 7.32 7.31 7.31 7.30 7.30 7.29 7.28 7.28 7.27 7.27 7.22 7.20 7.20 7.18 7.18 6.96 6.96 6.95 6.94 6.94 4.79 4.76 4.75 4.74 4.74 4.72 4.70 4.70 4.67 4.67 4.66 4.63 4.63 2.46 2.45 2.44
O
N
O
O
S
Ph
2j
1H NMR (400 MHz, CDCl3)
3.00
2.04
0.96
2.01
1.98
1.02
1.02
1.06
1.06
0.86
9.5 9.0 8.5 8.0 7.5 7.0 6.5 6.0 5.5 5.0 4.5 4.0 3.5 3.0 2.5 2.0 1.5 1.0 0.5
ppm

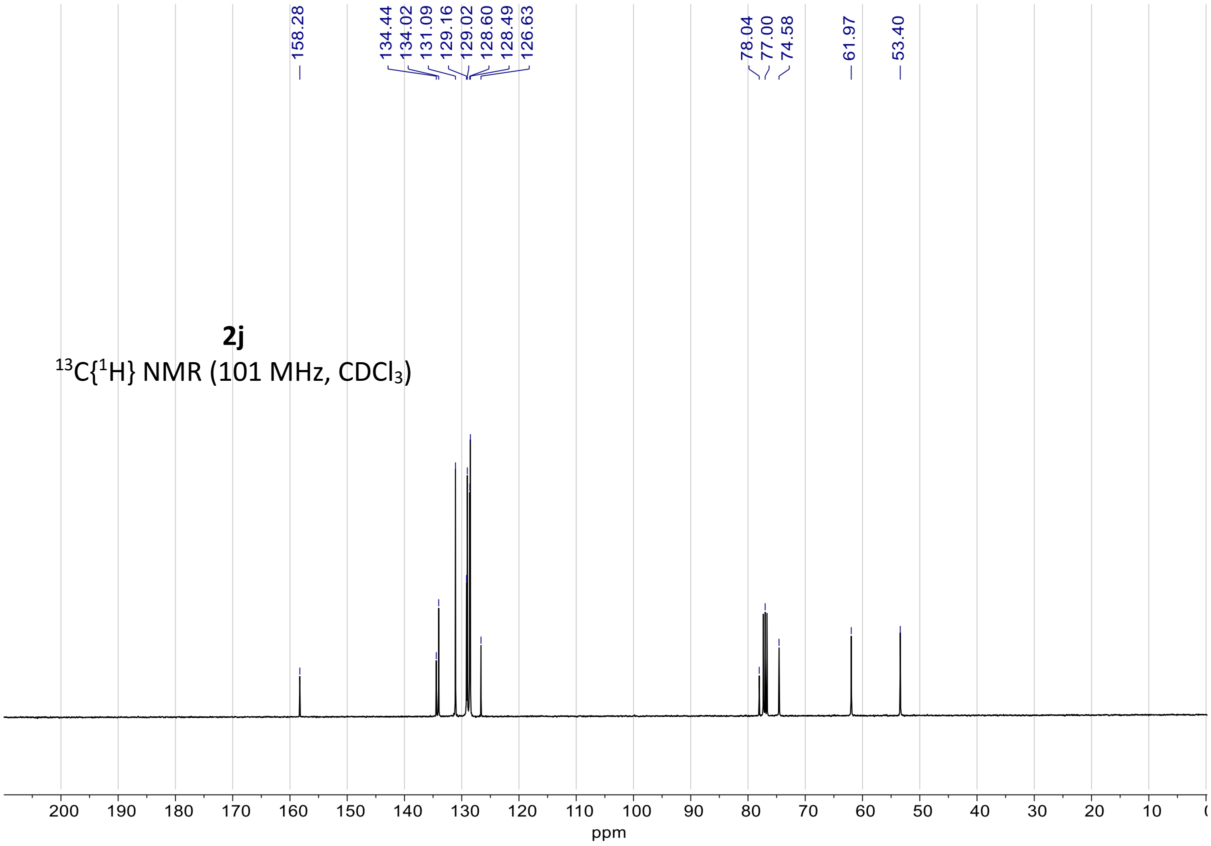

158.28
134.44 134.02 131.09 129.16 129.02 128.60 128.49 126.63
78.04 77.00 74.58
61.97
53.40
2j
13C{1H} NMR (101 MHz, CDCl3)
200 190 180 170 160 150 140 130 120 110 100 90 80 70 60 50 40 30 20 10
ppm

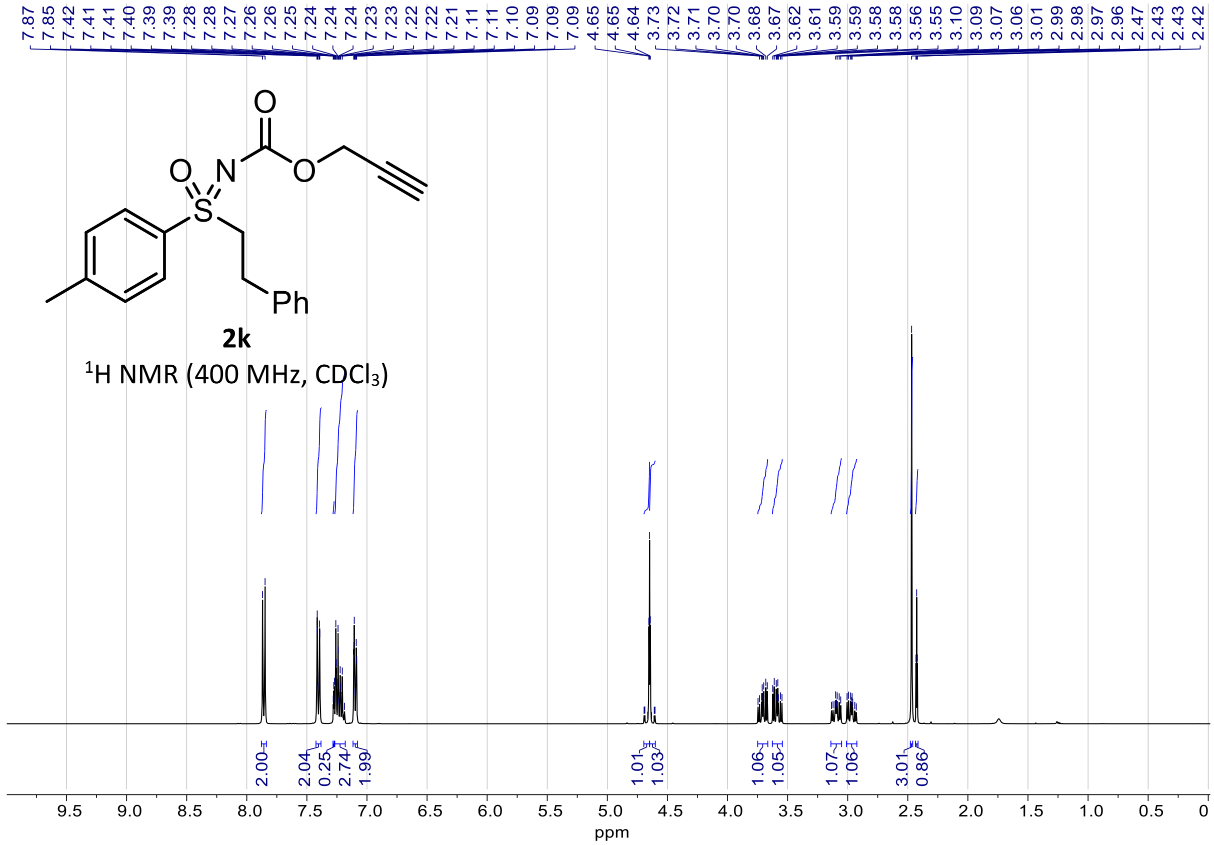
O
N
O
O
S
Ph
2k
1H NMR (400 MHz, CDCl3)
ppm

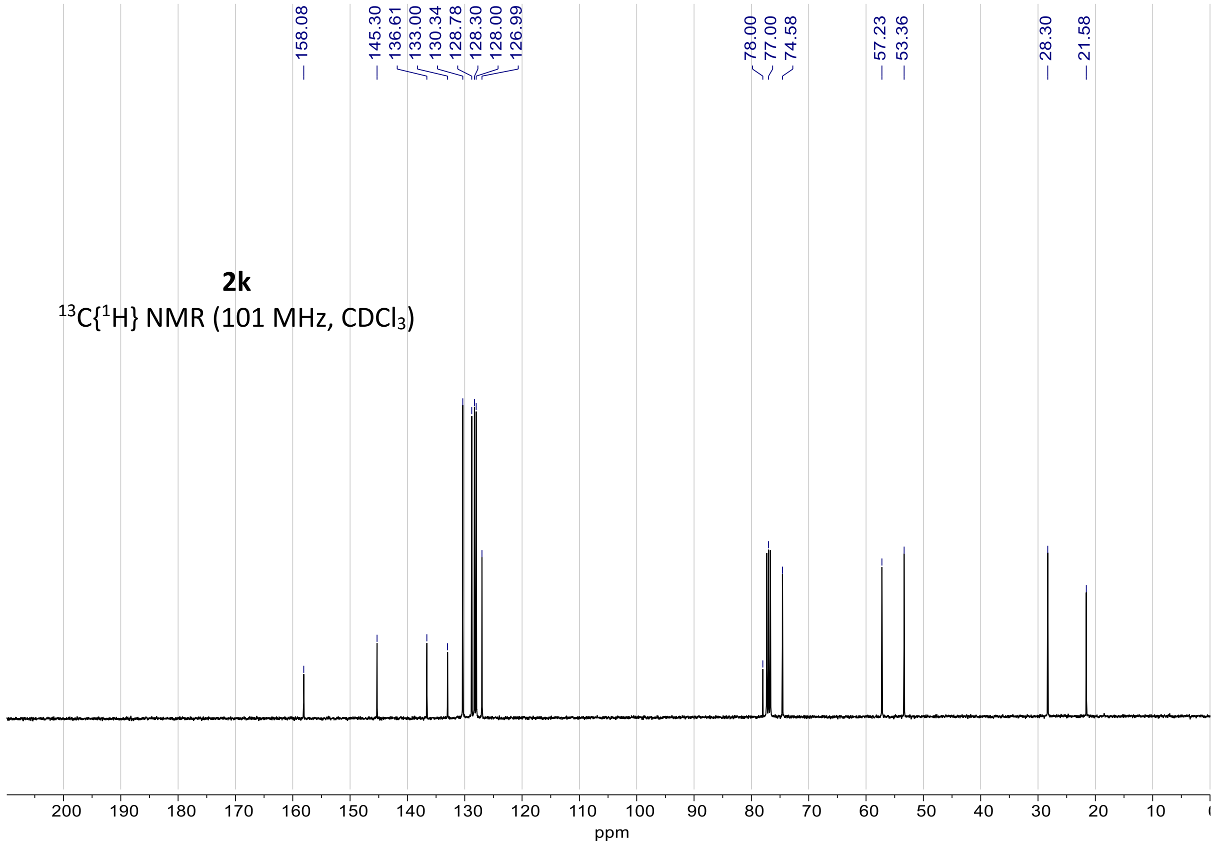
158.08
145.30
136.61
133.00
130.34
128.78
128.30
128.00
126.99
78.00
77.00
74.58
57.23
53.36
28.30
21.58
2k
13C{1H} NMR (101 MHz, CDCl3)
ppm

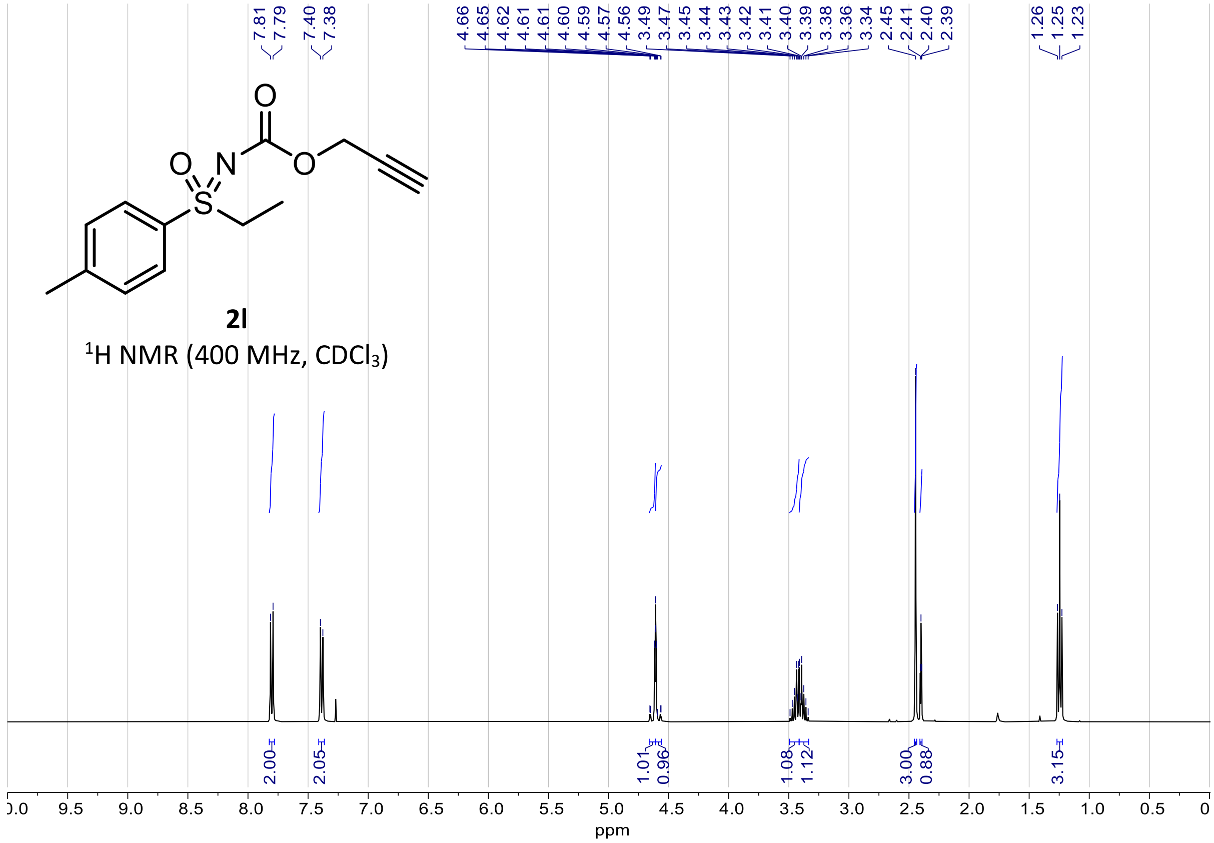

2l

^{1}H NMR (400 MHz, $CDCl_3$)

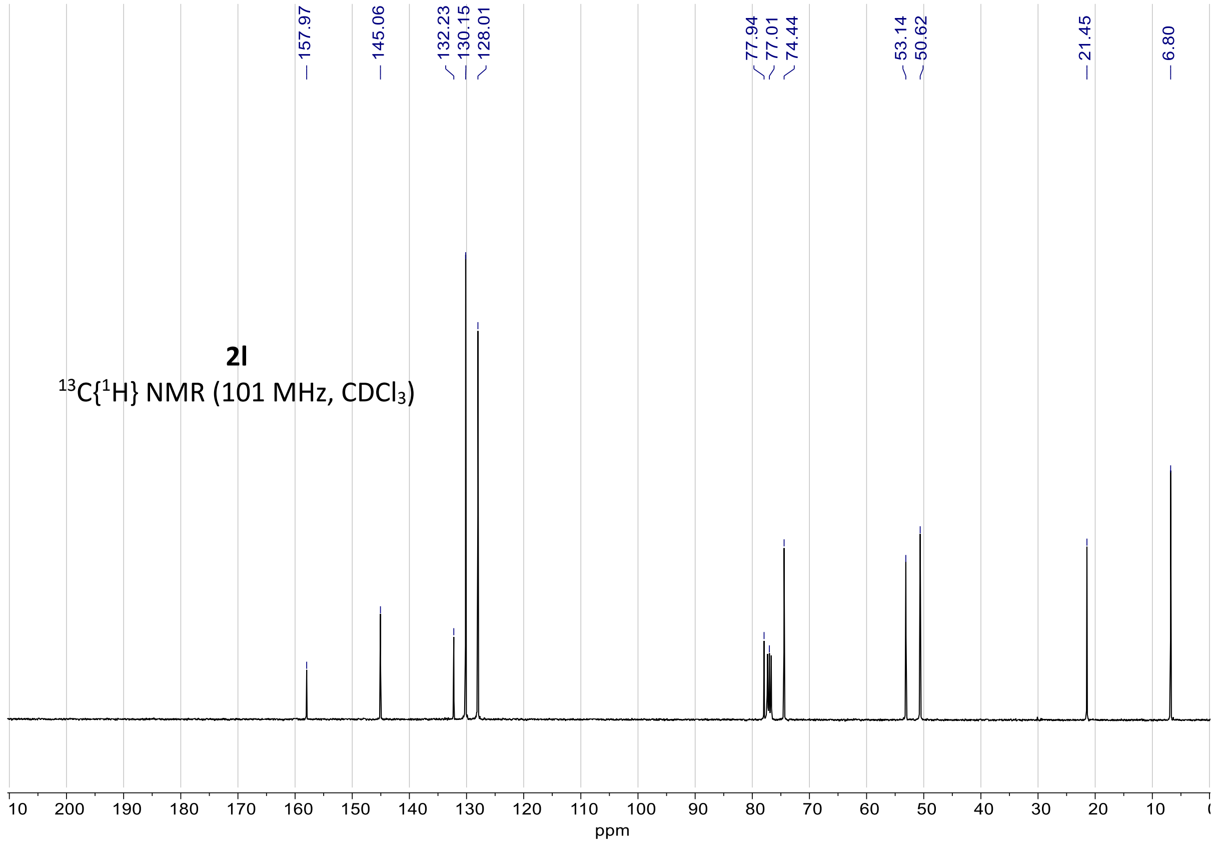

2l

$^{13}C\{^{1}H\}$ NMR (101 MHz, $CDCl_3$)

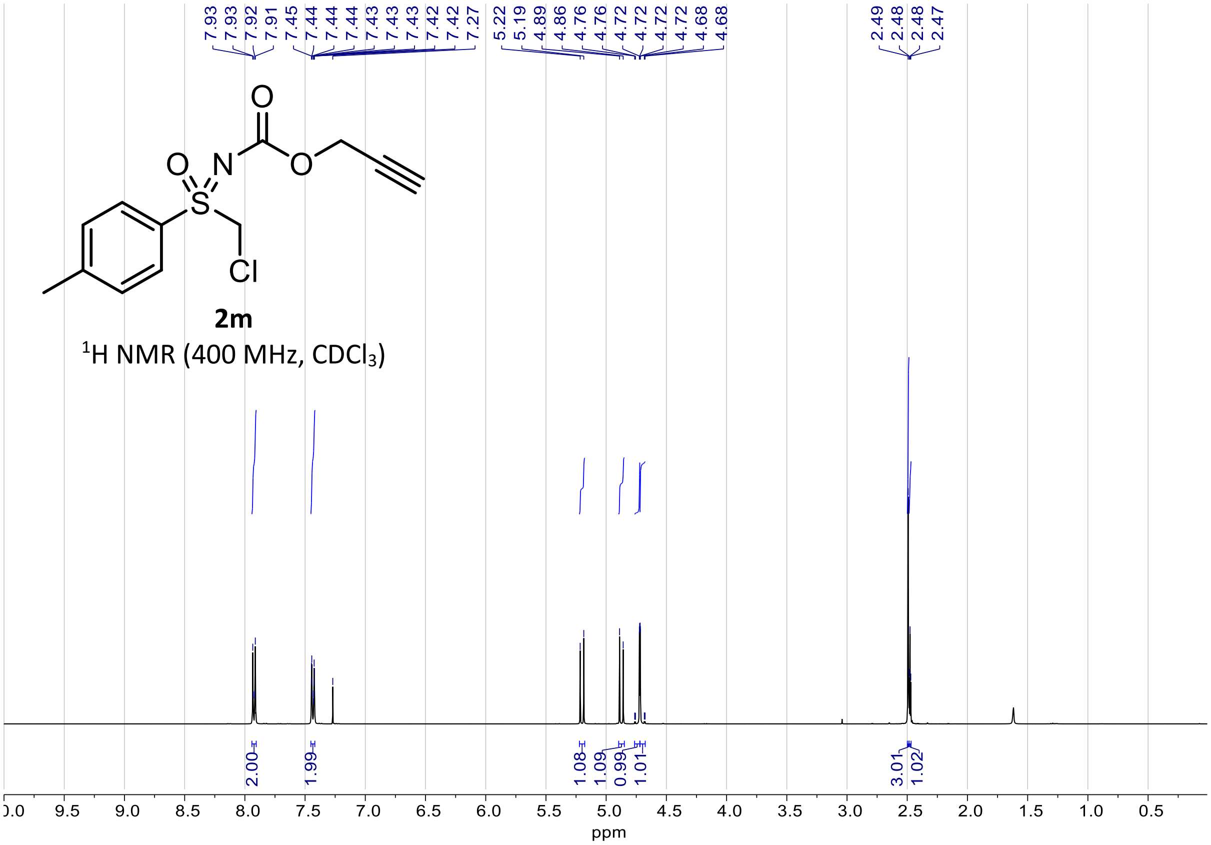

2m

^{1}H NMR (400 MHz, $CDCl_3$)

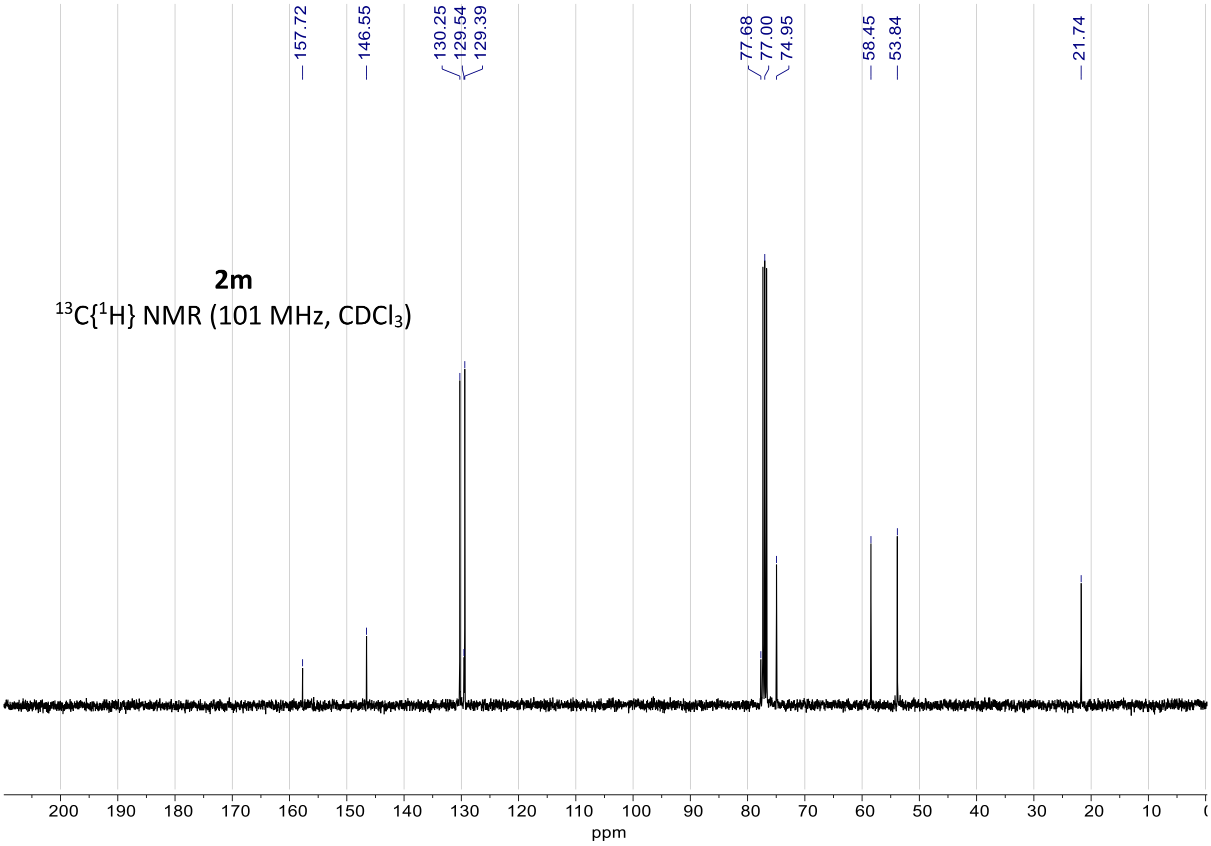

2m

$^{13}C\{^1H\}$ NMR (101 MHz, $CDCl_3$)

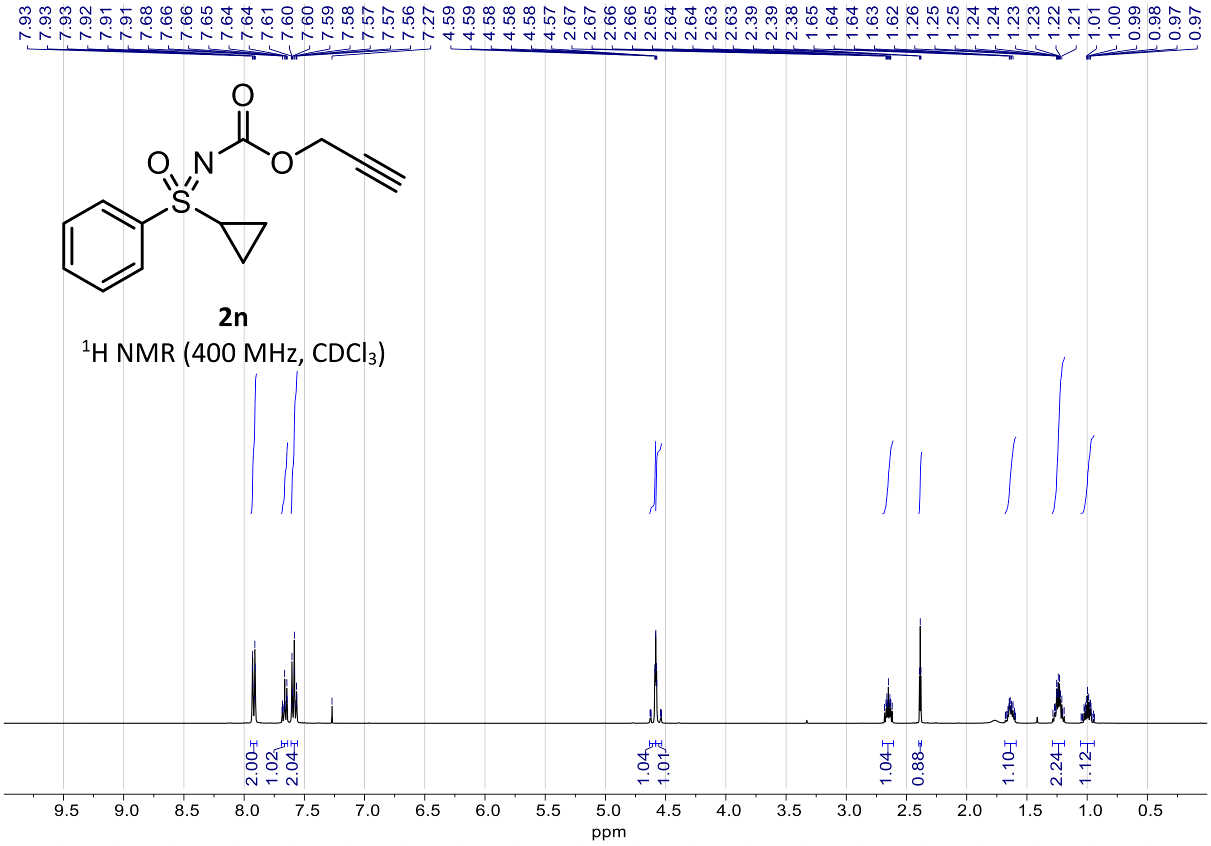
2n
1H NMR (400 MHz, CDCl3)
ppm

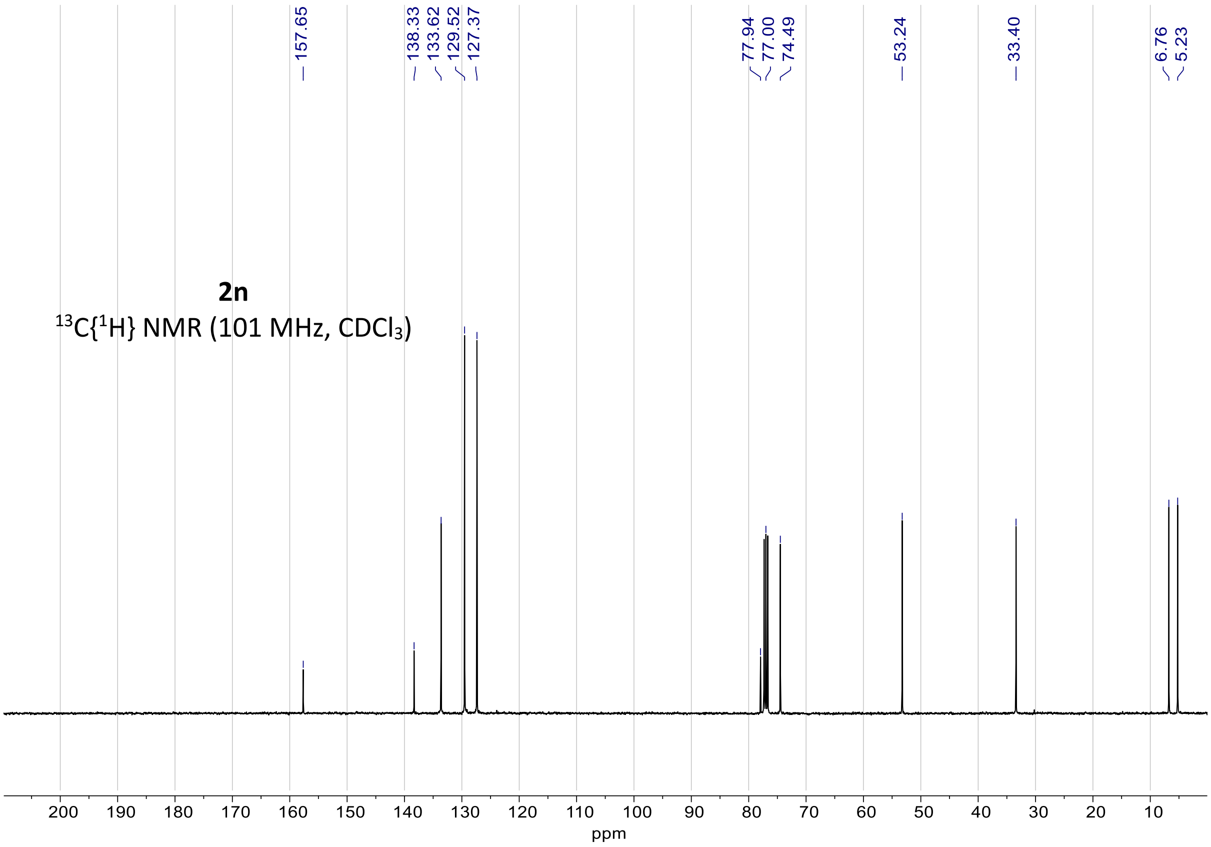
157.65
138.33
133.62
129.52
127.37
77.94
77.00
74.49
53.24
33.40
6.76
5.23
2n
13C{1H} NMR (101 MHz, CDCl3)
ppm

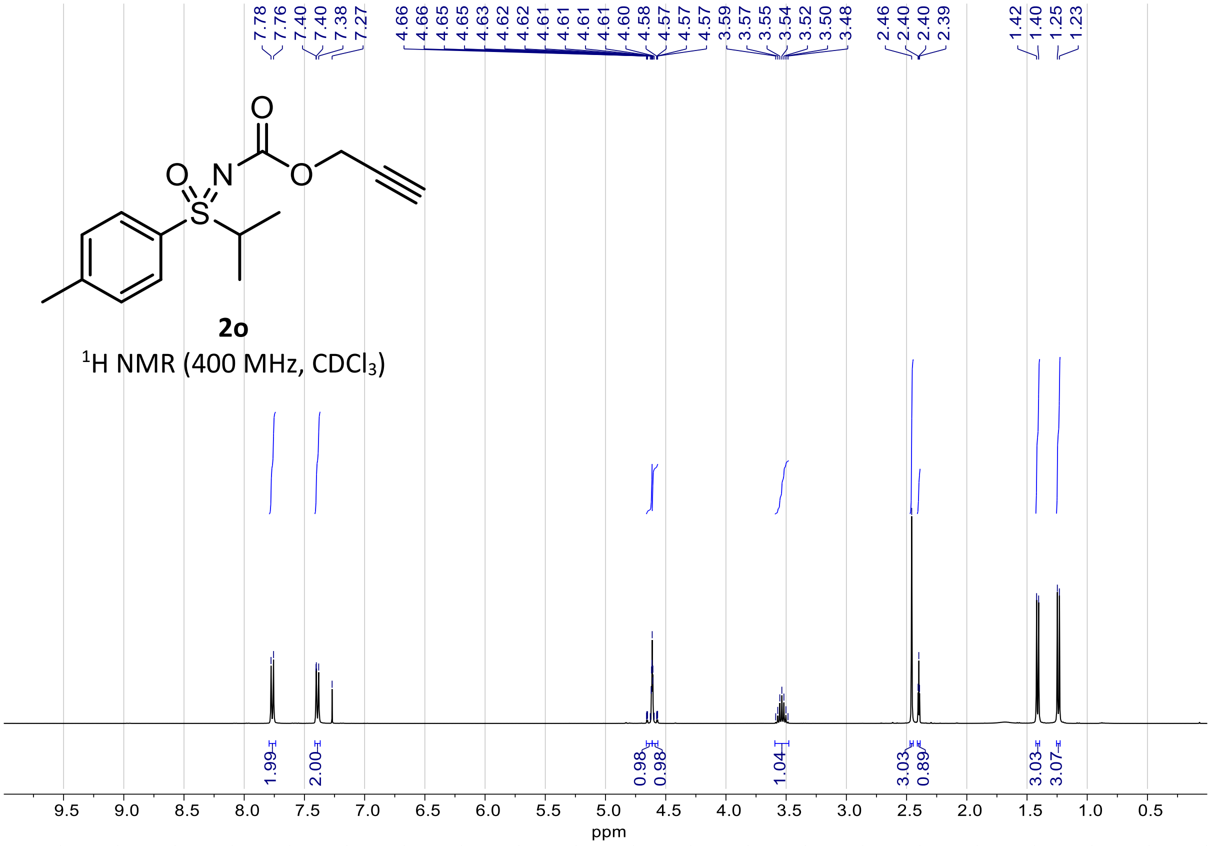
2o
^{1}H NMR (400 MHz, CDCl$_3$)
7.78
7.76
7.40
7.40
7.38
7.27
4.66
4.66
4.65
4.65
4.63
4.62
4.62
4.61
4.61
4.61
4.61
4.60
4.58
4.57
4.57
4.57
3.59
3.57
3.55
3.54
3.52
3.50
3.48
2.46
2.40
2.40
2.39
1.42
1.40
1.25
1.23
1.99
2.00
0.98
0.98
1.04
3.03
0.89
3.03
3.07
ppm

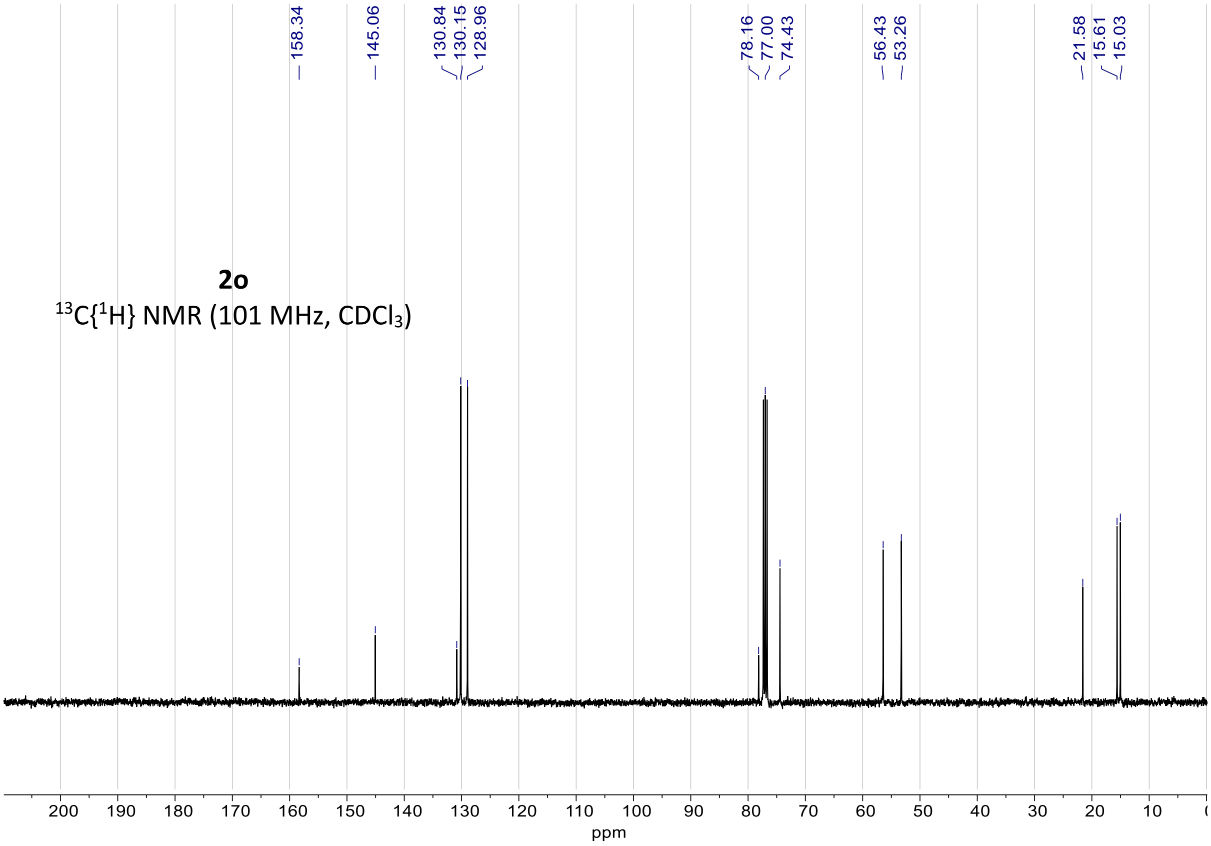
2o
^{13}C{^{1}H} NMR (101 MHz, CDCl$_3$)
158.34
145.06
130.84
130.15
128.96
78.16
77.00
74.43
56.43
53.26
21.58
15.61
15.03
ppm

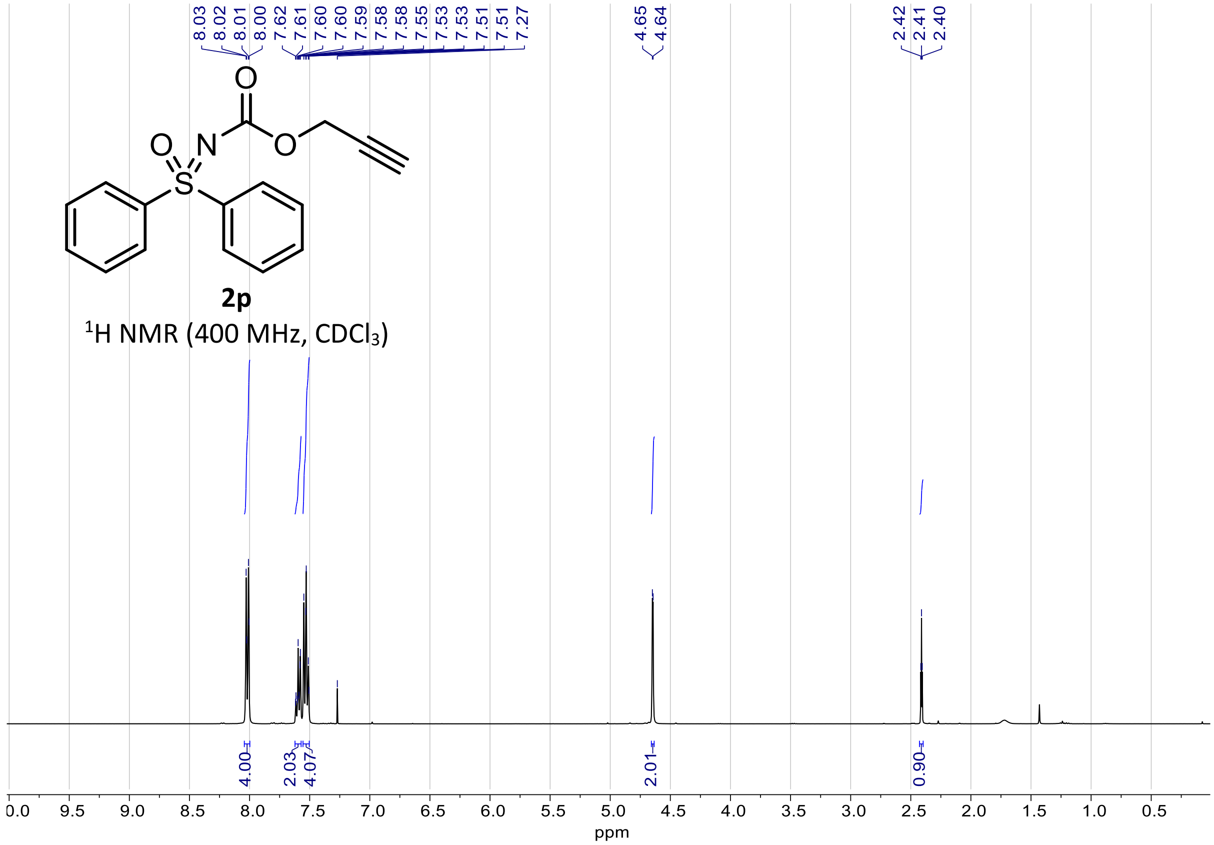

2p

^{1}H NMR (400 MHz, $CDCl_3$)

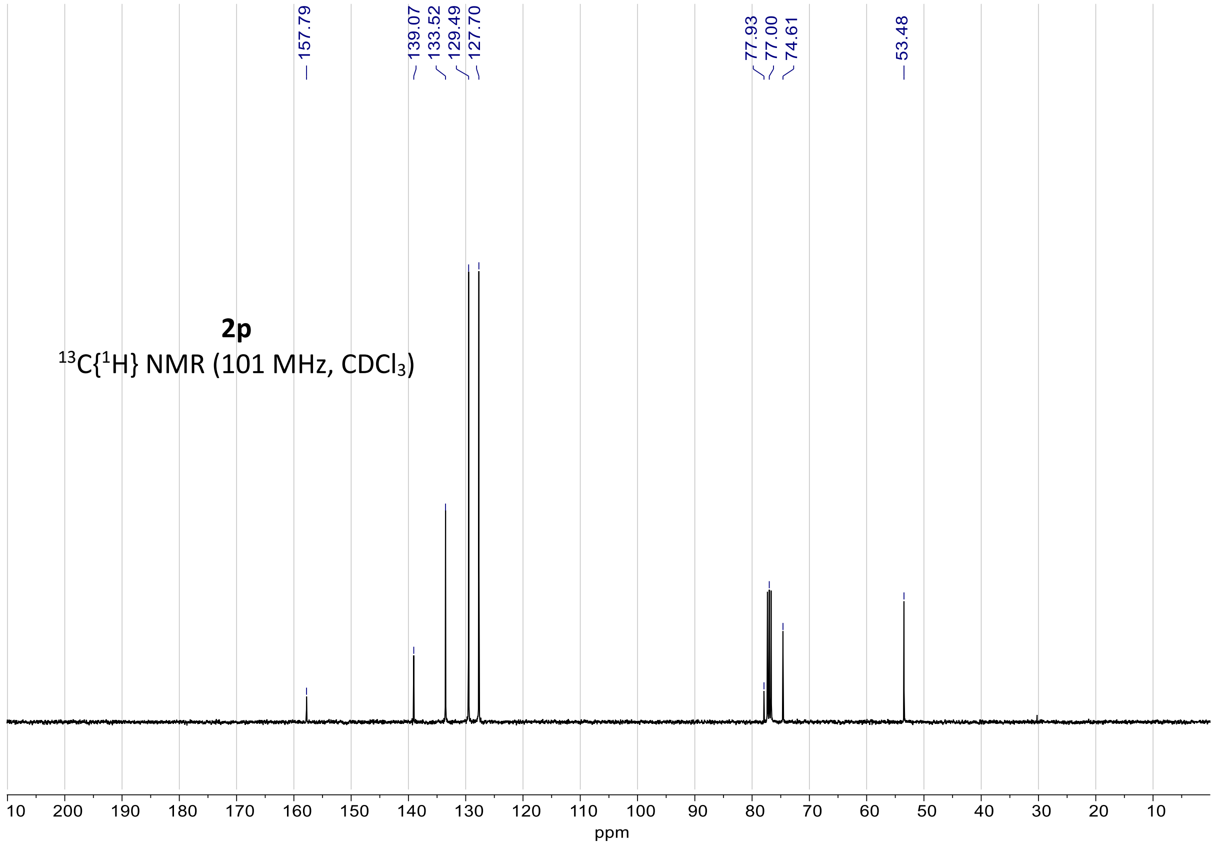

2p

$^{13}C\{^1H\}$ NMR (101 MHz, $CDCl_3$)

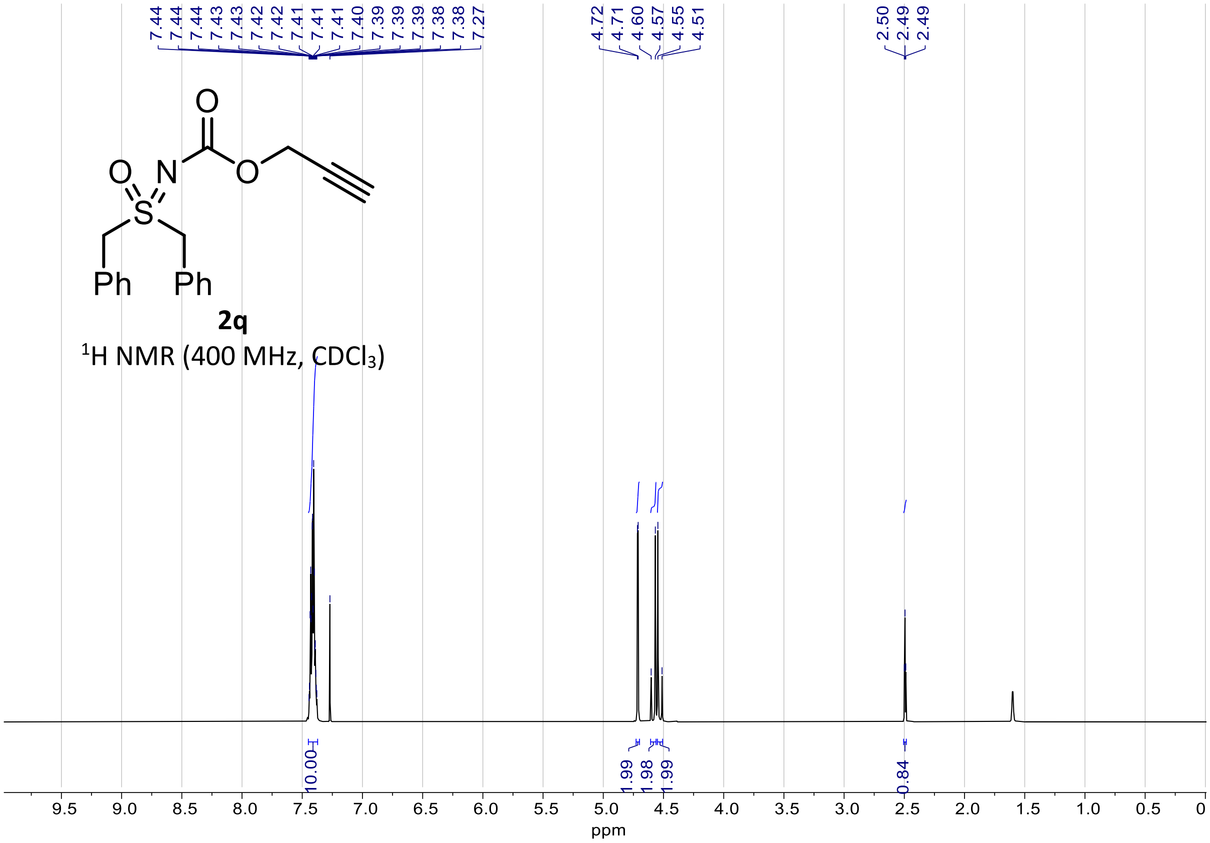

2q

^{1}H NMR (400 MHz, $CDCl_3$)

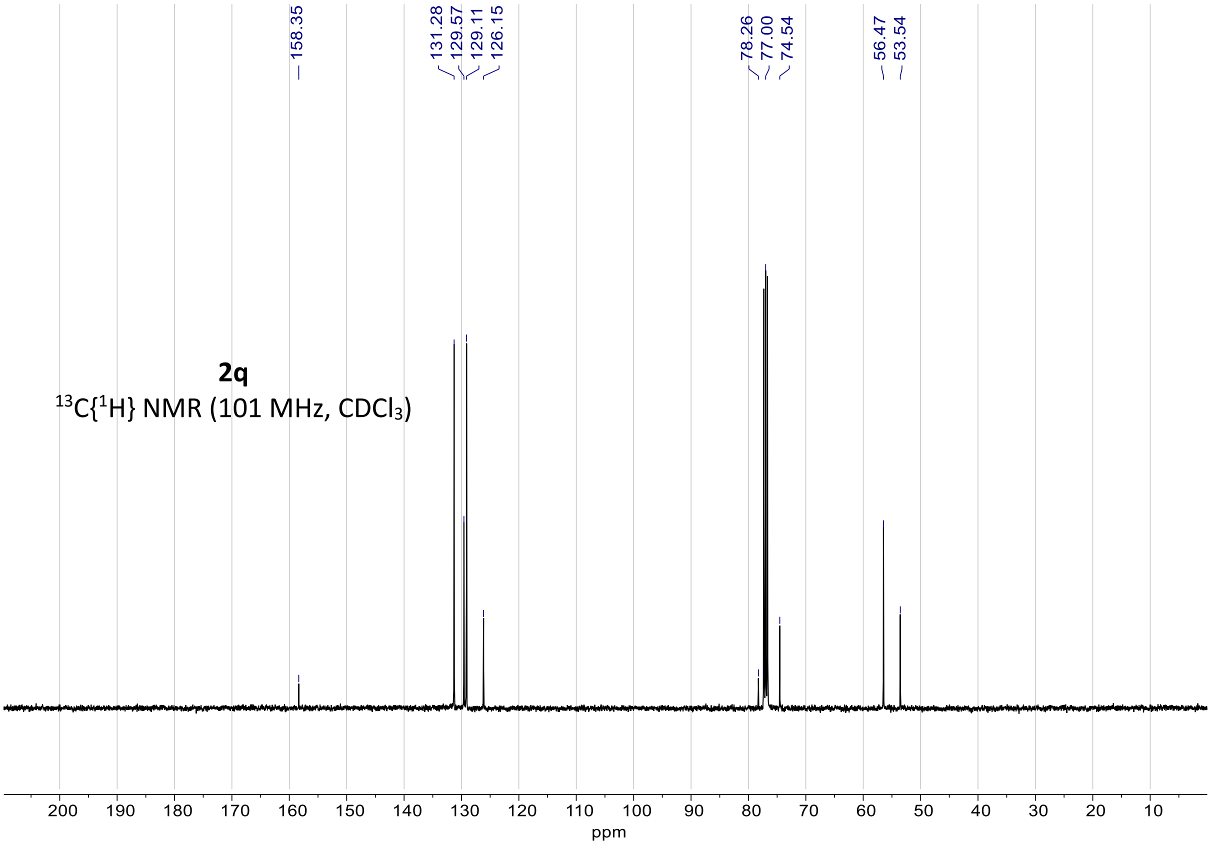

2q

$^{13}C\{^{1}H\}$ NMR (101 MHz, $CDCl_3$)

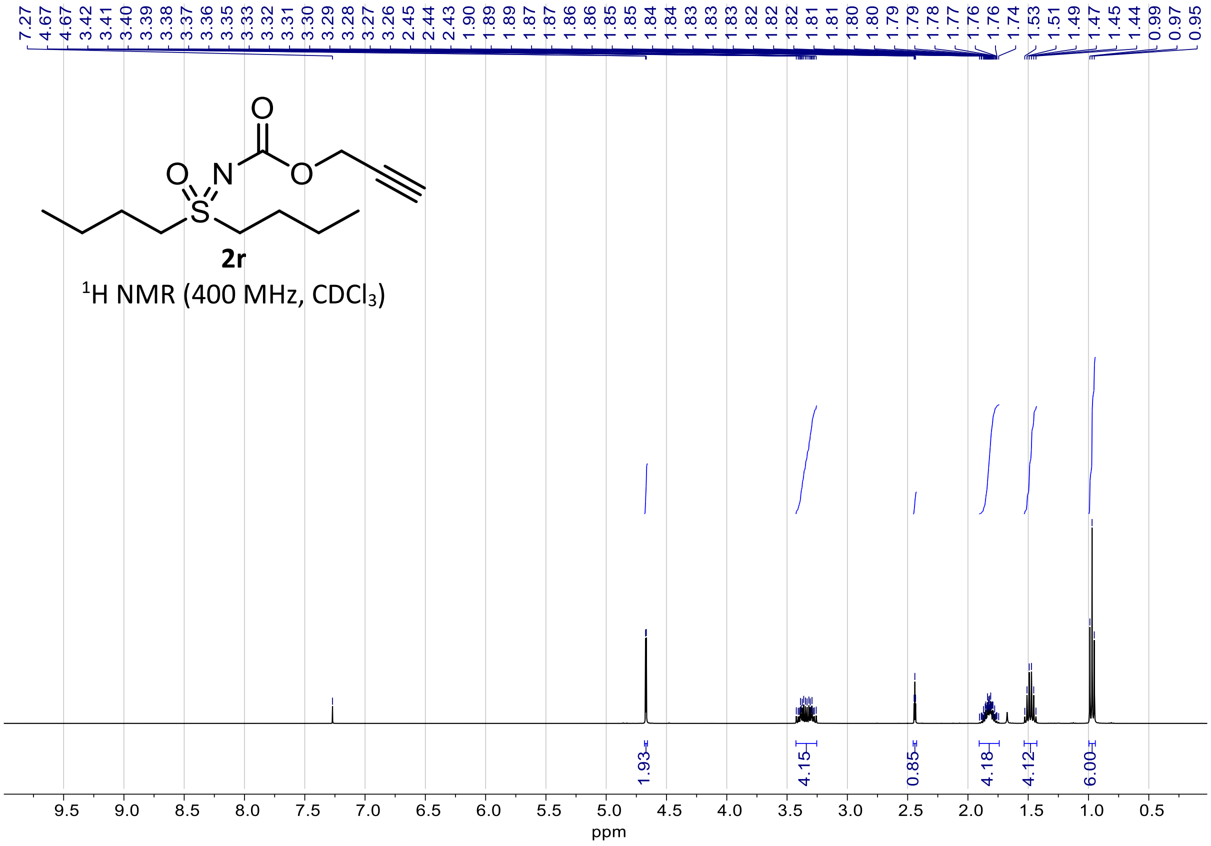
7.27 4.67 4.67 3.42 3.41 3.40 3.39 3.38 3.37 3.36 3.35 3.33 3.32 3.31 3.30 3.29 3.28 3.27 3.26 2.45 2.44 2.43 1.90 1.89 1.89 1.87 1.87 1.86 1.85 1.85 1.84 1.84 1.83 1.83 1.83 1.82 1.82 1.82 1.81 1.81 1.80 1.80 1.79 1.79 1.78 1.77 1.76 1.76 1.74 1.53 1.51 1.49 1.47 1.45 1.44 0.99 0.97 0.95
O
N
O
O
S
2r
^{1}H NMR (400 MHz, CDCl$_3$)
1.93
4.15
0.85
4.18
4.12
6.00
9.5 9.0 8.5 8.0 7.5 7.0 6.5 6.0 5.5 5.0 4.5 4.0 3.5 3.0 2.5 2.0 1.5 1.0 0.5
ppm

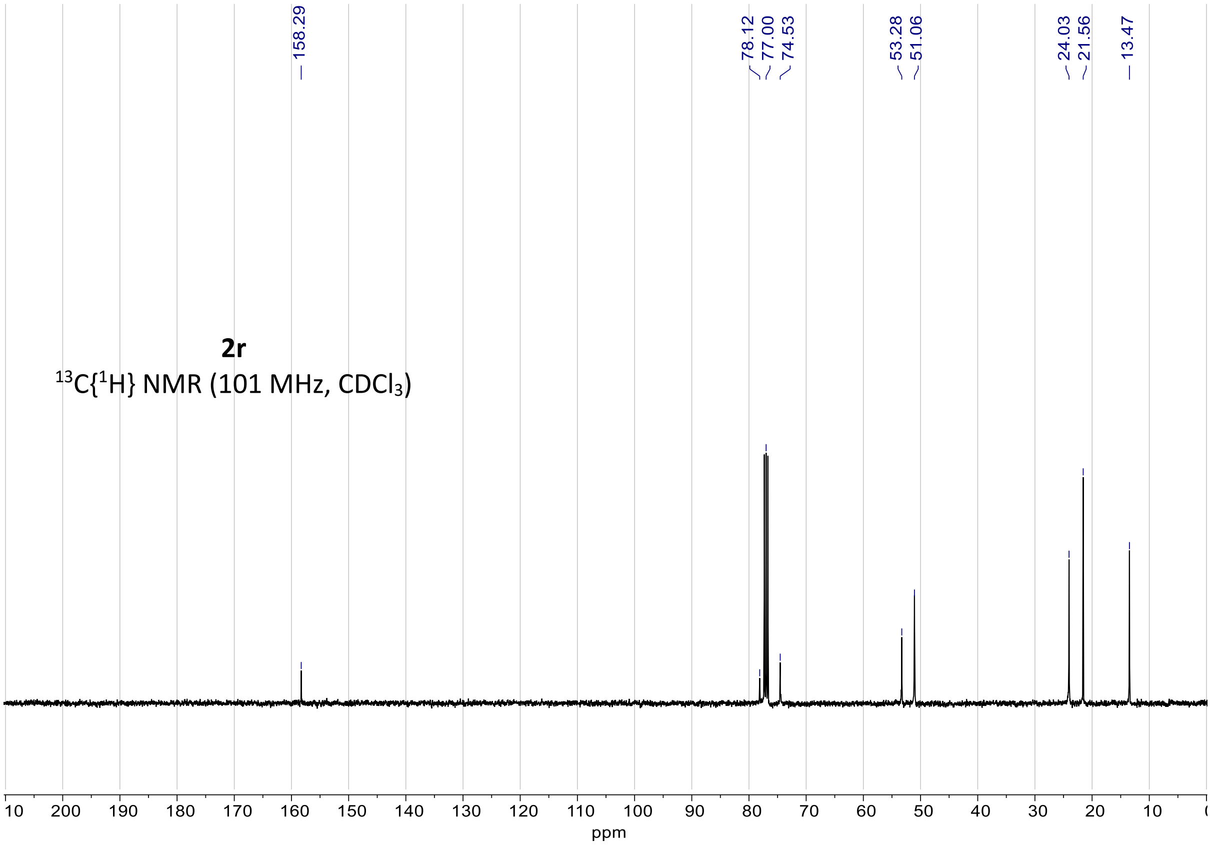
158.29
78.12 77.00 74.53
53.28 51.06
24.03 21.56
13.47
2r
^{13}C{^{1}H} NMR (101 MHz, CDCl$_3$)
10 200 190 180 170 160 150 140 130 120 110 100 90 80 70 60 50 40 30 20 10
ppm

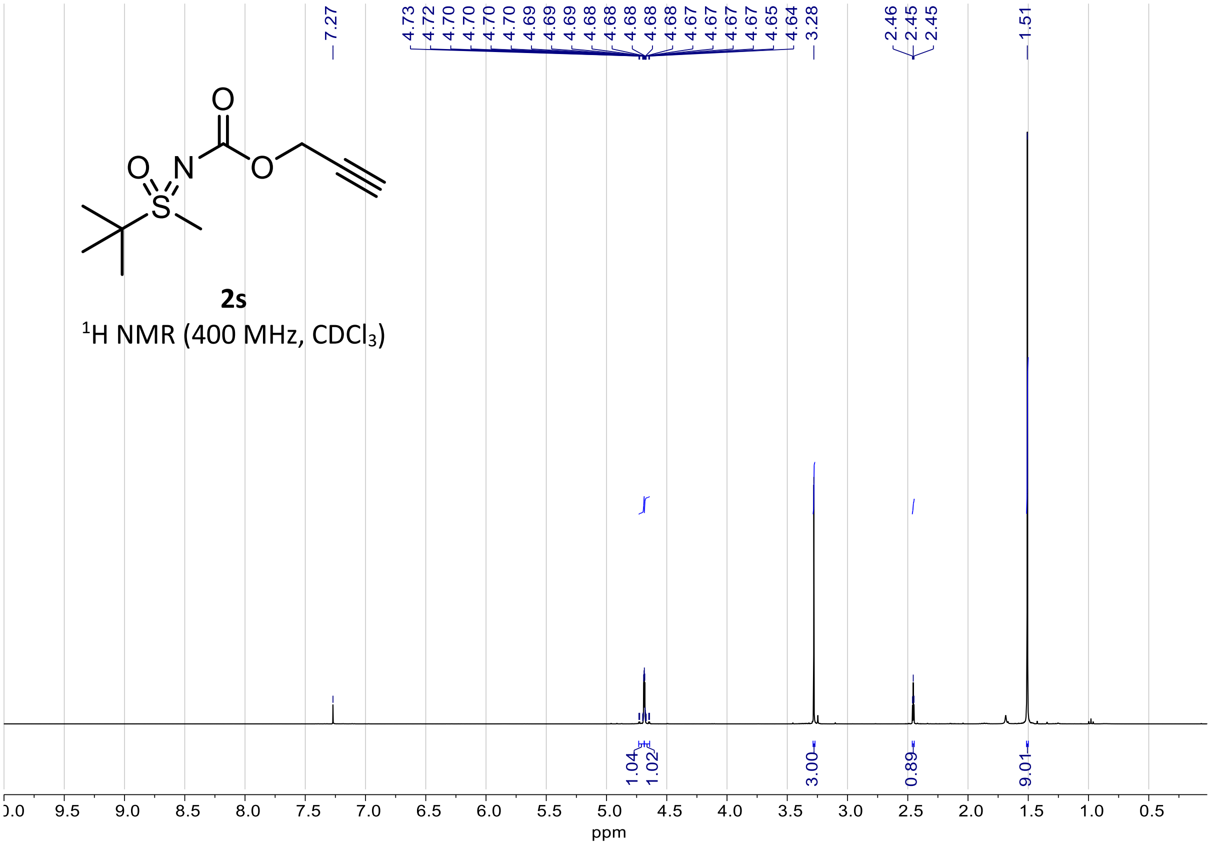

2s
^{1}H NMR (400 MHz, CDCl$_3$)
7.27
4.73 4.72 4.70 4.70 4.70 4.69 4.69 4.68 4.68 4.68 4.68 4.68 4.67 4.67 4.67 4.65 4.64
3.28
2.46 2.45 2.45
1.51
1.04 1.02 3.00 0.89 9.01
ppm

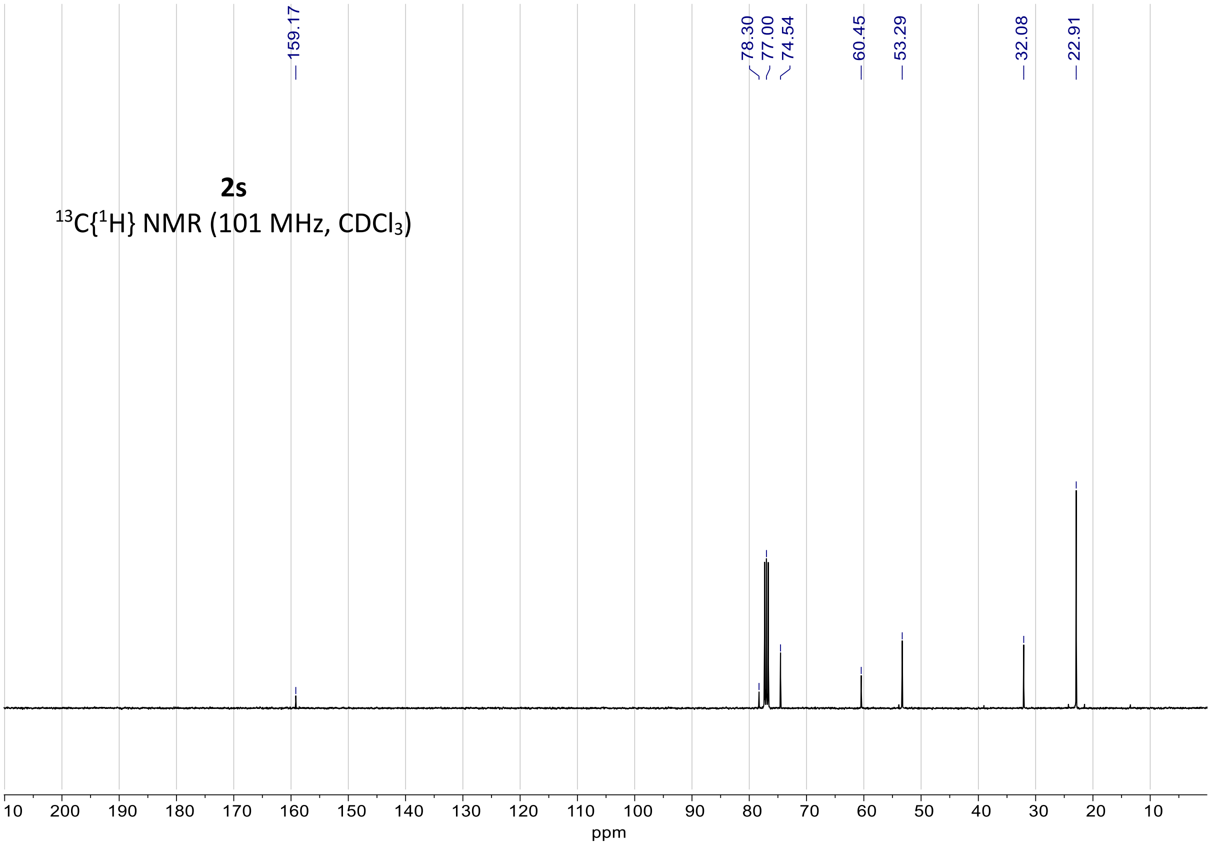

2s
^{13}C{^{1}H} NMR (101 MHz, CDCl$_3$)
159.17
78.30 77.00 74.54
60.45
53.29
32.08
22.91
ppm

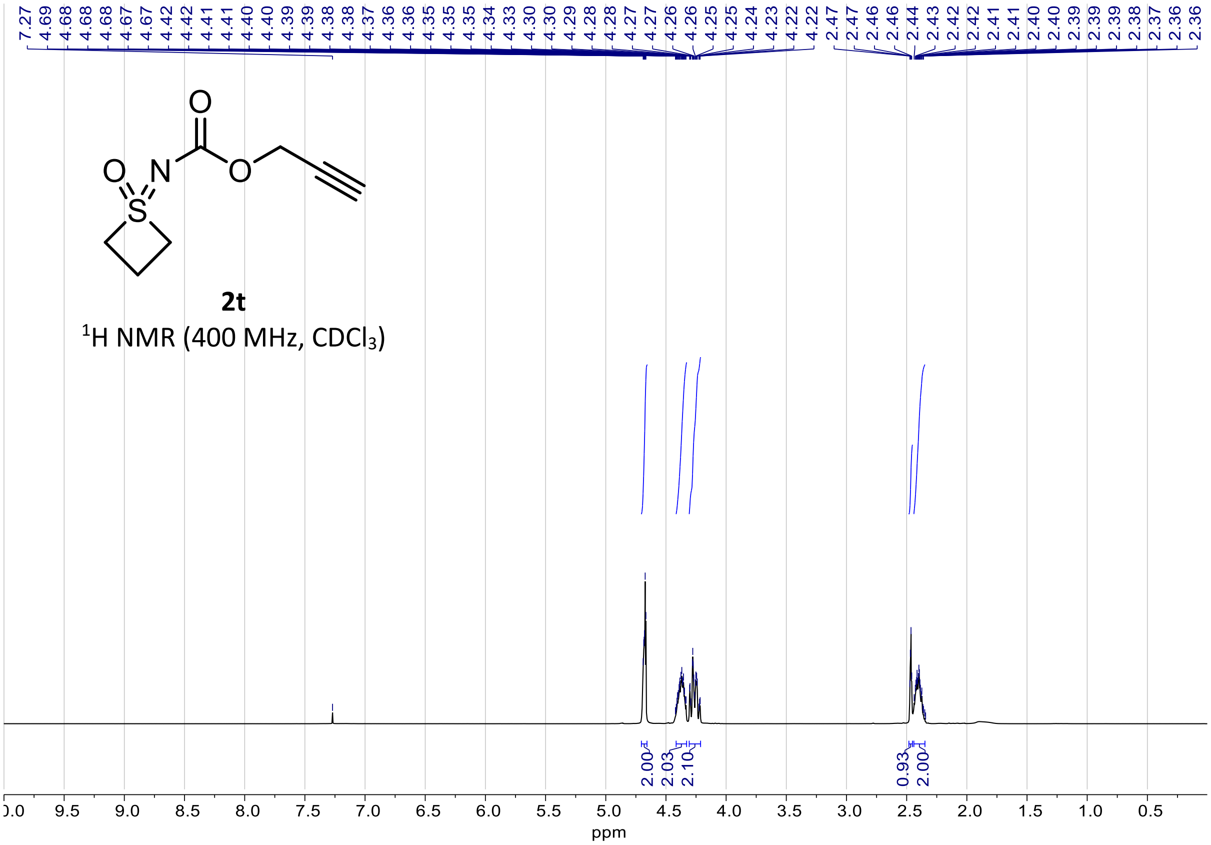

2t
^{1}H NMR (400 MHz, CDCl$_3$)

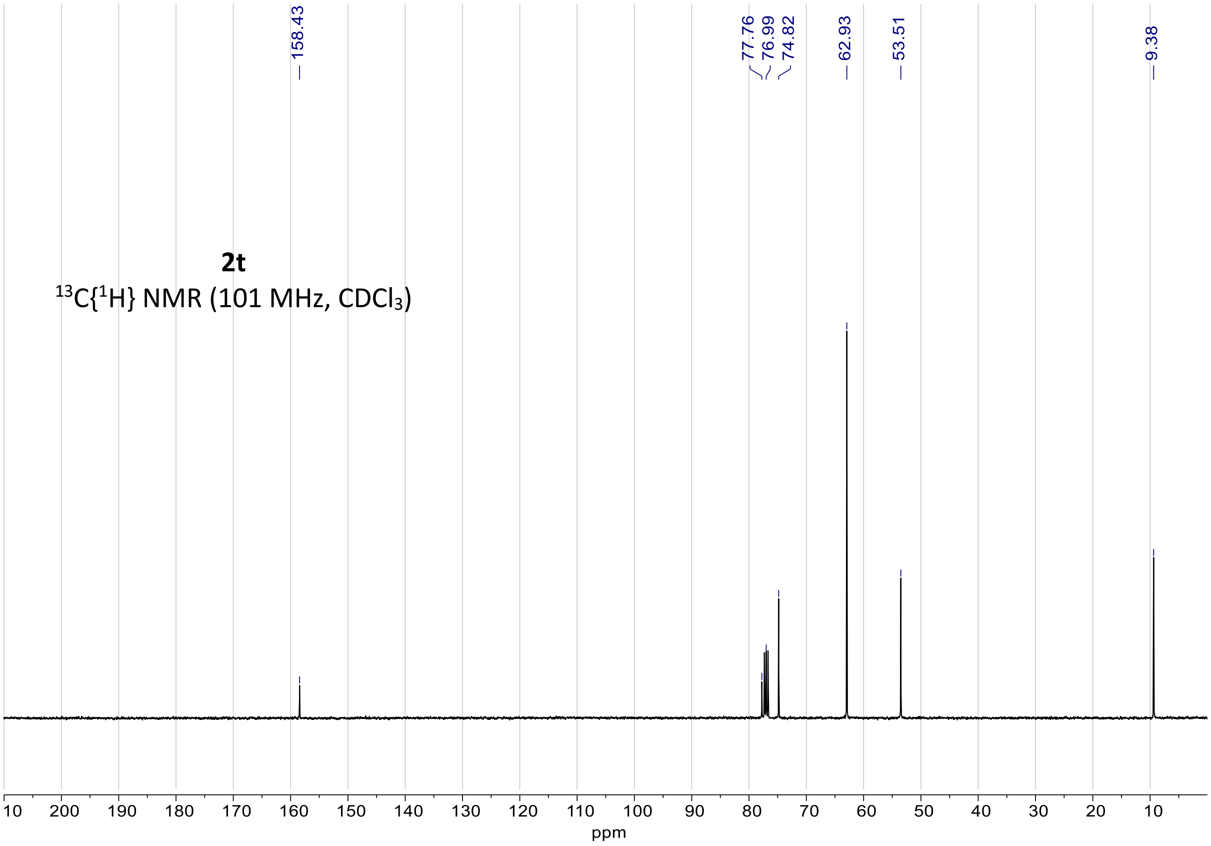

2t
^{13}C{^{1}H} NMR (101 MHz, CDCl$_3$)

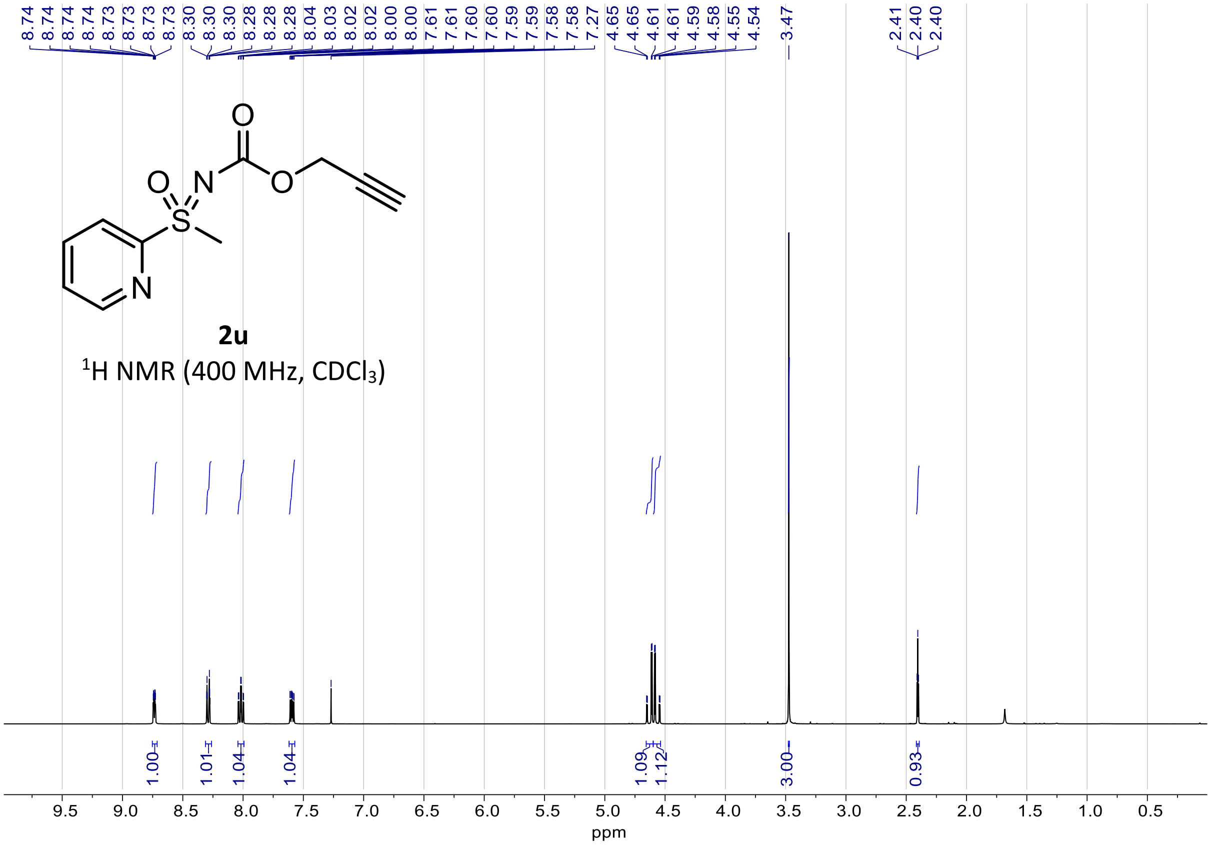

2u

^{1}H NMR (400 MHz, $CDCl_3$)

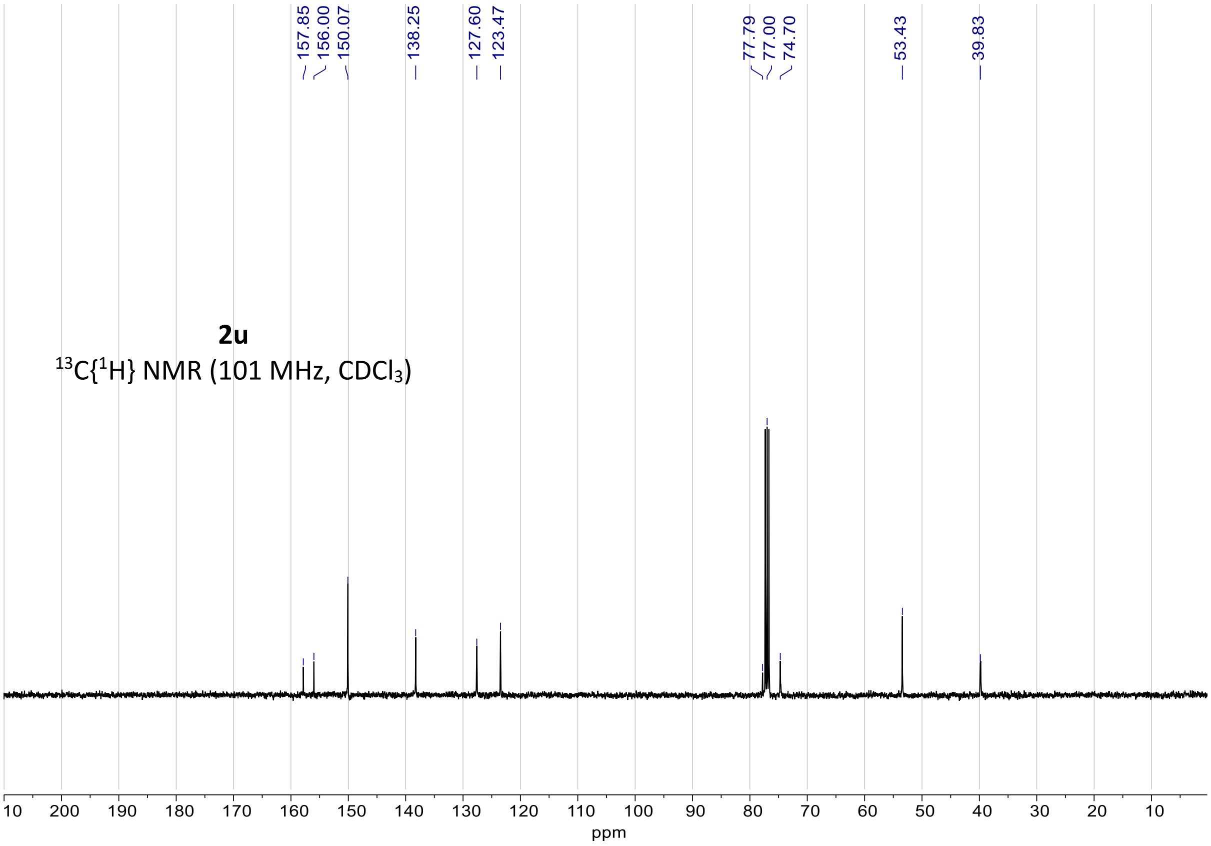

2u

^{13}C{^{1}H} NMR (101 MHz, $CDCl_3$)

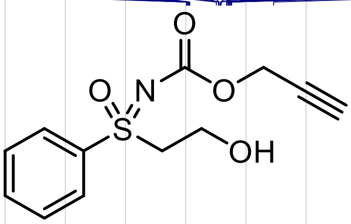

2v

^{1}H NMR (400 MHz, $CDCl_3$)

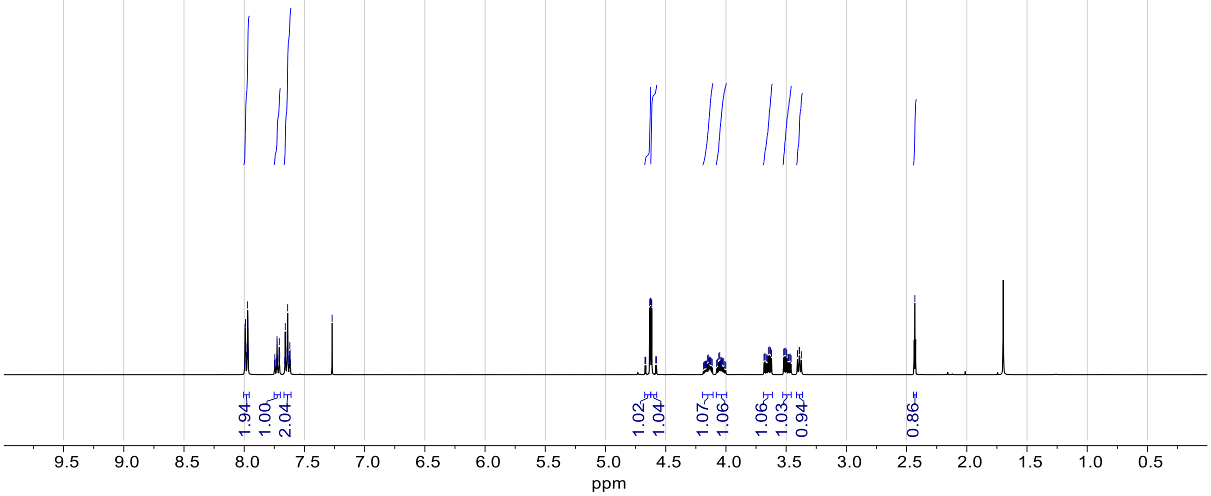

2v

^{13}C{^{1}H} NMR (101 MHz, $CDCl_3$)

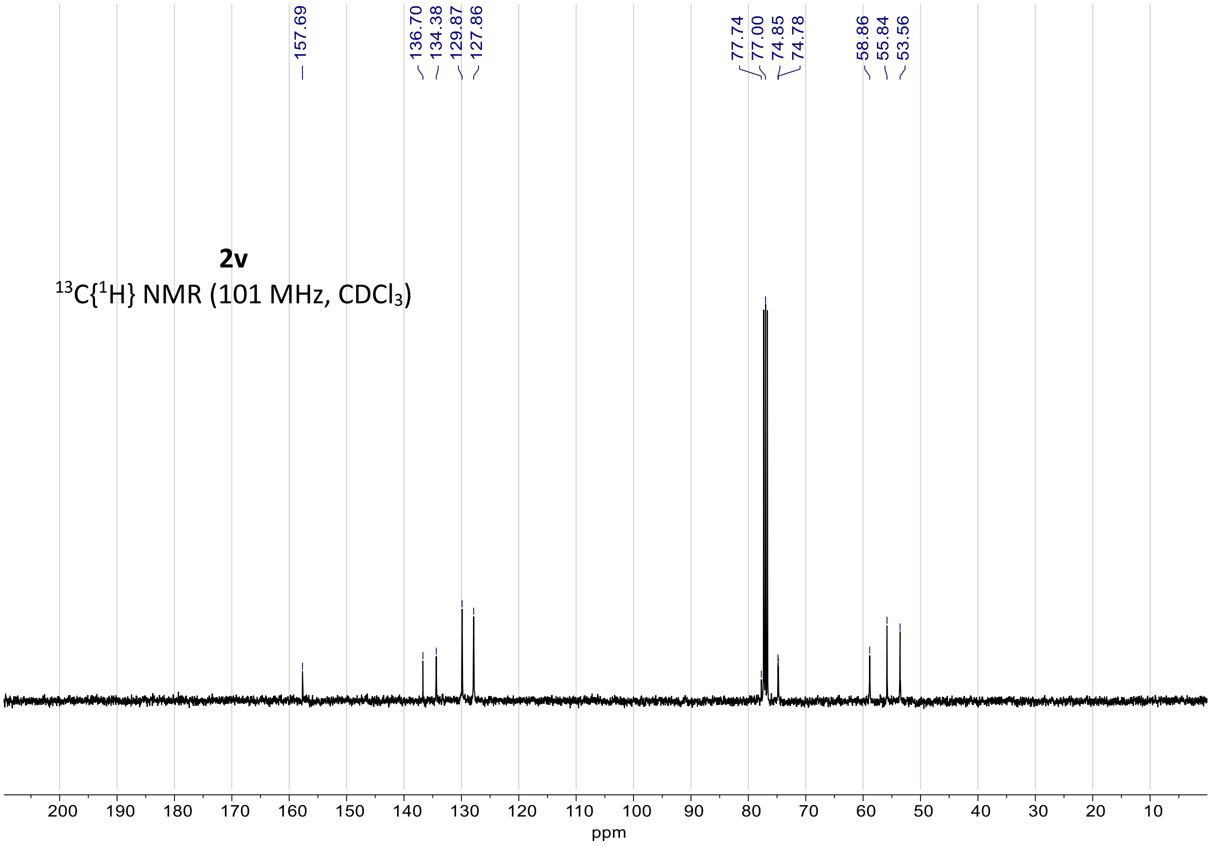

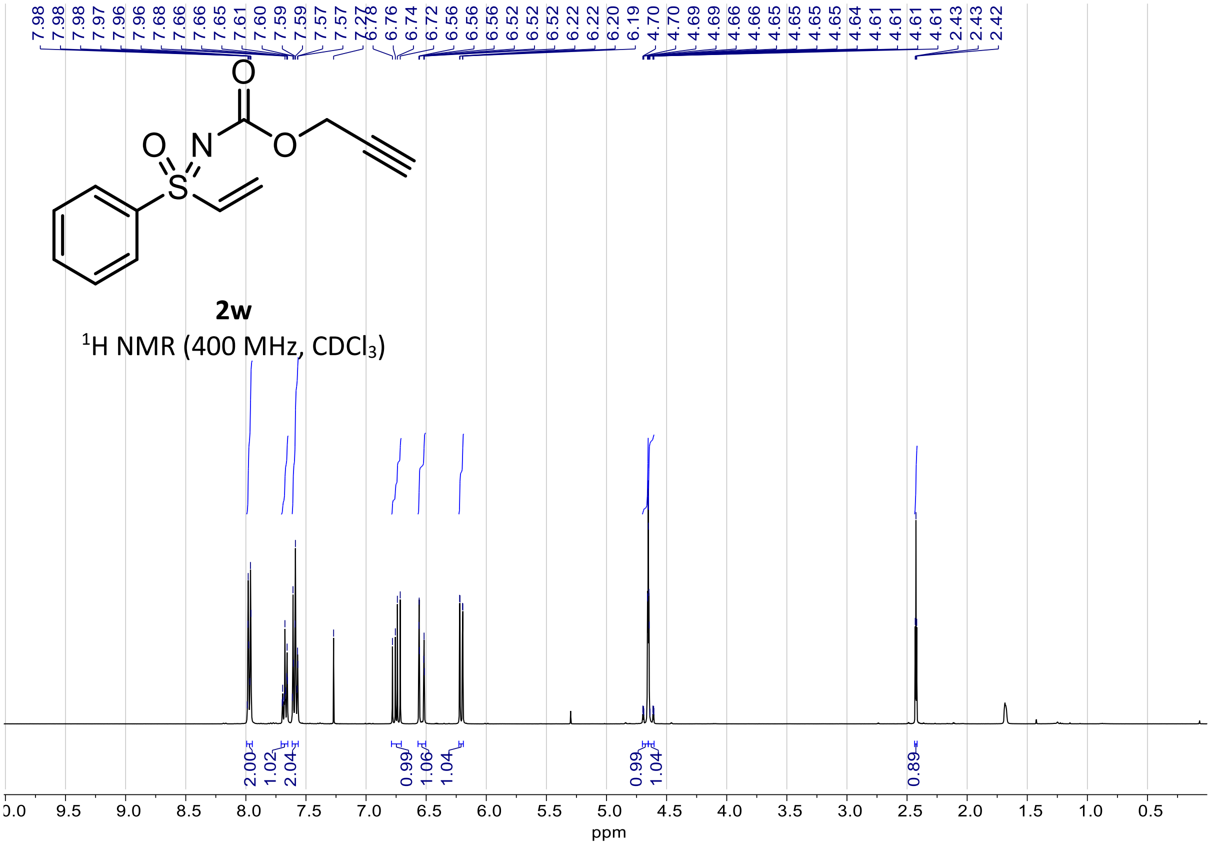

2w

^{1}H NMR (400 MHz, $CDCl_3$)

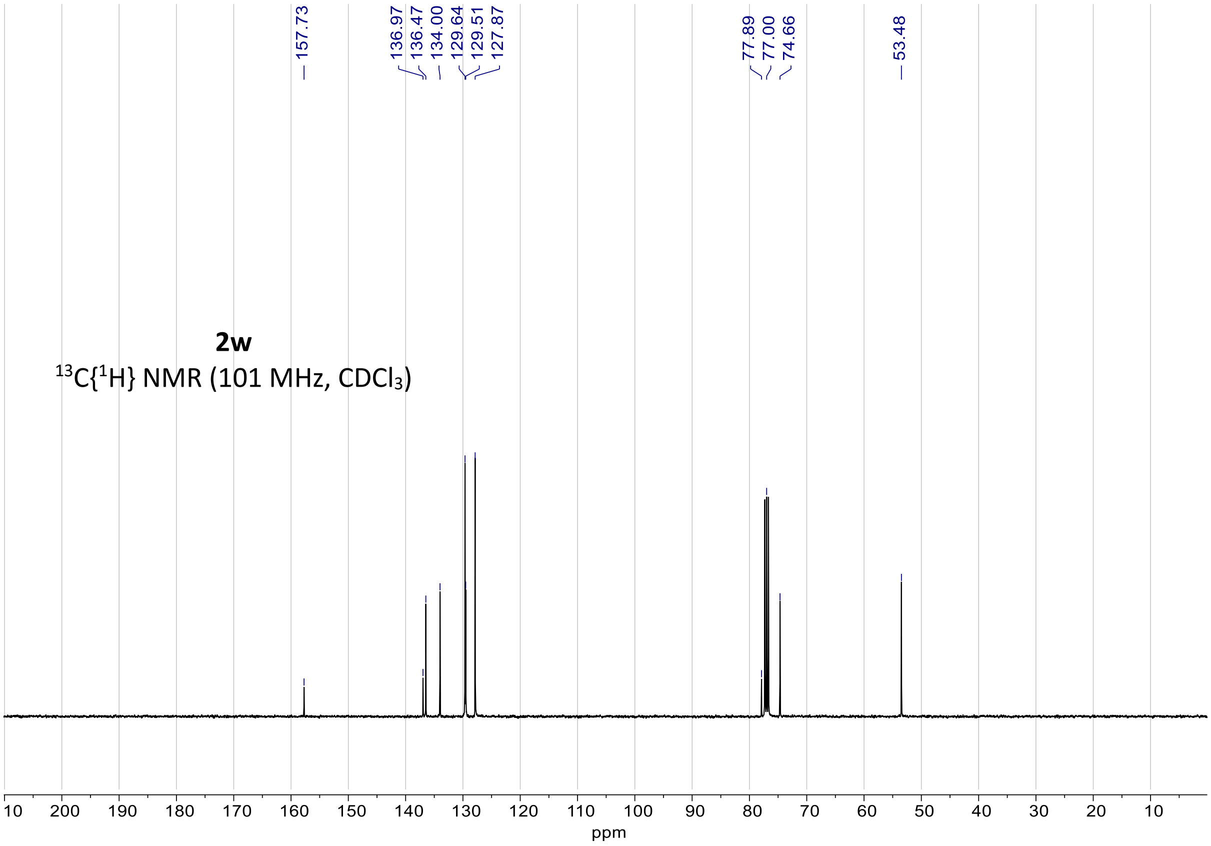

2w

$^{13}C\{^{1}H\}$ NMR (101 MHz, $CDCl_3$)

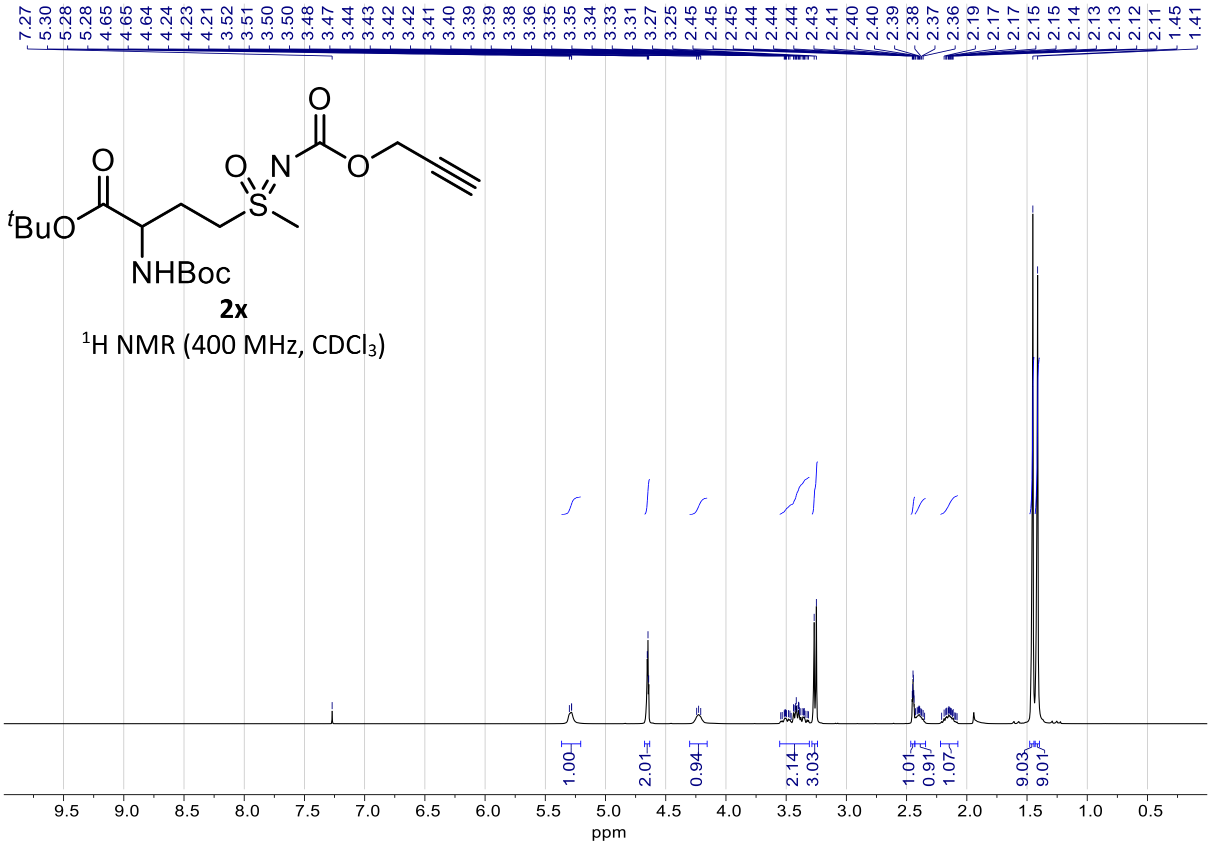
7.27 5.30 5.28 5.28 4.65 4.64 4.24 4.23 4.21 3.52 3.51 3.50 3.50 3.48 3.47 3.44 3.43 3.42 3.42 3.41 3.40 3.39 3.39 3.38 3.36 3.35 3.35 3.34 3.33 3.31 3.27 3.25 2.45 2.45 2.45 2.44 2.44 2.44 2.43 2.41 2.40 2.40 2.39 2.38 2.38 2.37 2.36 2.19 2.17 2.17 2.15 2.15 2.14 2.13 2.13 2.12 2.11 1.45 1.41
O
O
N
O
O
S
^{t}BuO
NHBoc
2x
^{1}H NMR (400 MHz, CDCl$_3$)
1.00 2.01 0.94 2.14 3.03 1.01 0.91 1.07 9.03 9.01
9.5 9.0 8.5 8.0 7.5 7.0 6.5 6.0 5.5 5.0 4.5 4.0 3.5 3.0 2.5 2.0 1.5 1.0 0.5
ppm

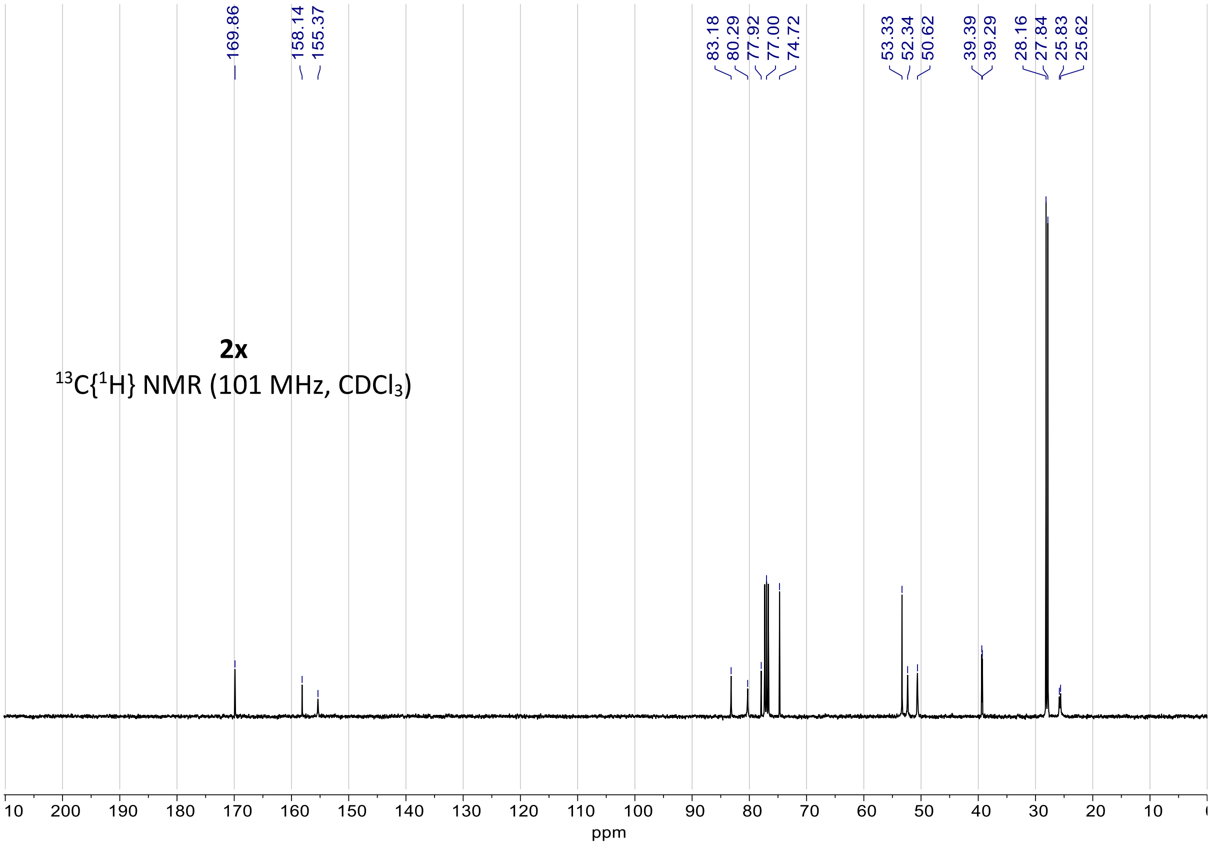
169.86
158.14 155.37
83.18 80.29 77.92 77.00 74.72
53.33 52.34 50.62
39.39 39.29
28.16 27.84 25.83 25.62
2x
^{13}C{^{1}H} NMR (101 MHz, CDCl$_3$)
10 200 190 180 170 160 150 140 130 120 110 100 90 80 70 60 50 40 30 20 10
ppm

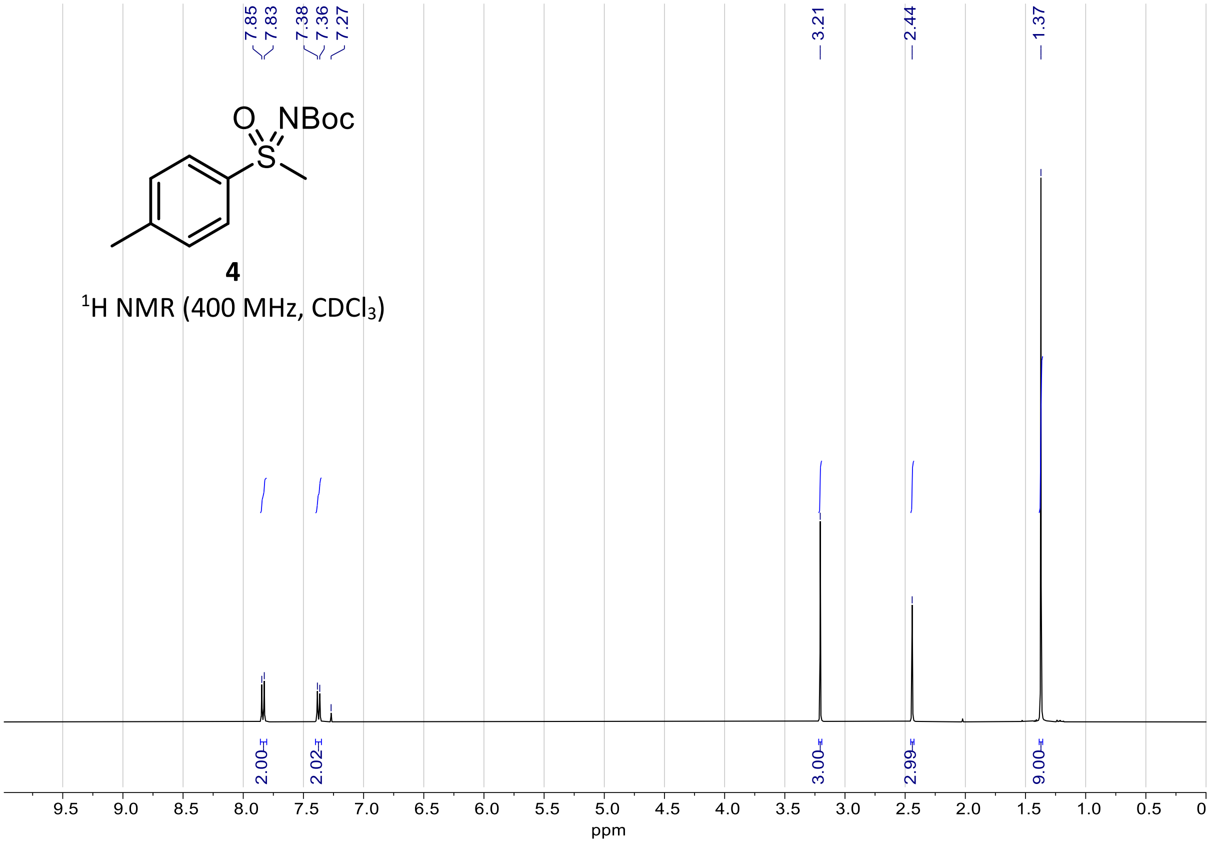
7.85
7.83
7.38
7.36
7.27
3.21
2.44
1.37
O
NBoc
S
4
1H NMR (400 MHz, CDCl3)
2.00
2.02
3.00
2.99
9.00
9.5 9.0 8.5 8.0 7.5 7.0 6.5 6.0 5.5 5.0 4.5 4.0 3.5 3.0 2.5 2.0 1.5 1.0 0.5 0
ppm

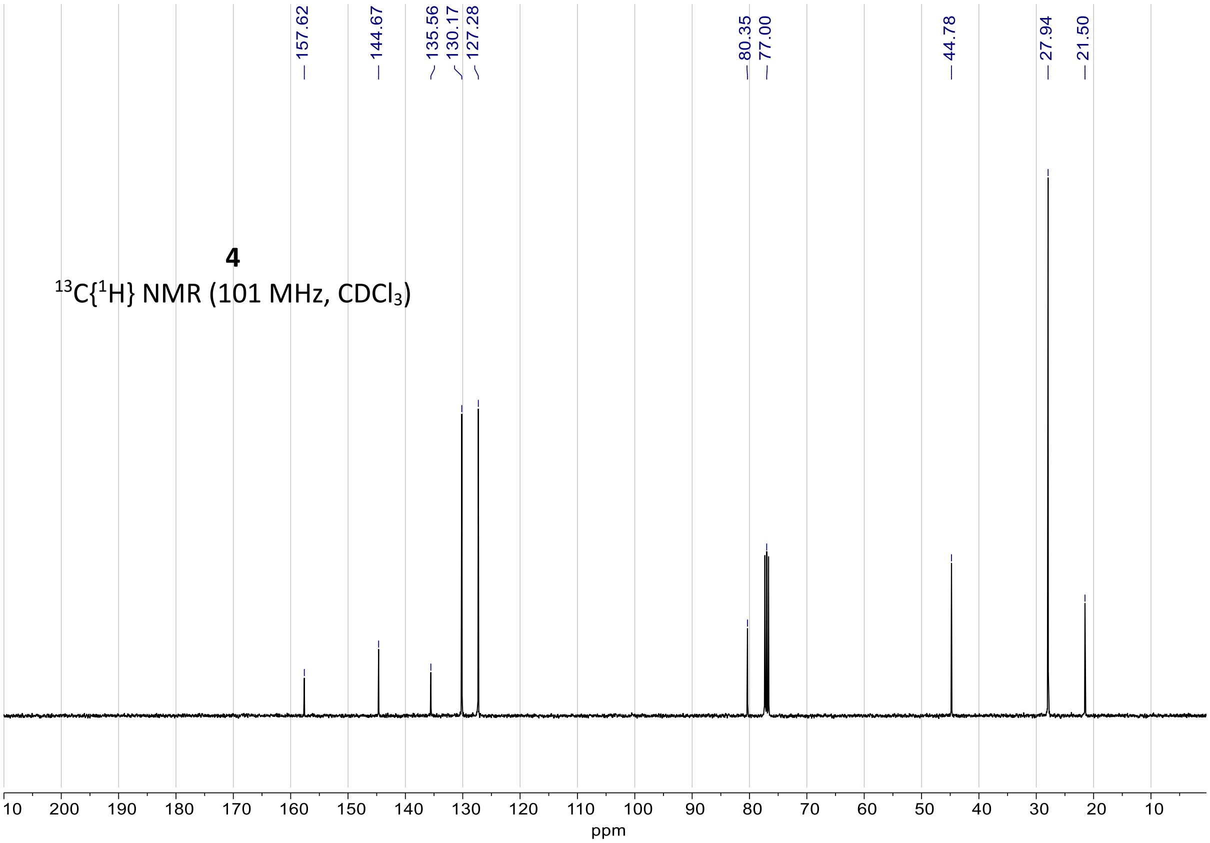
157.62
144.67
135.56
130.17
127.28
80.35
77.00
44.78
27.94
21.50
4
13C{1H} NMR (101 MHz, CDCl3)
10 200 190 180 170 160 150 140 130 120 110 100 90 80 70 60 50 40 30 20 10
ppm

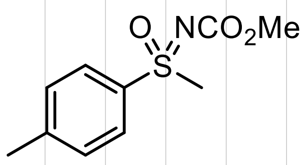

5

^{1}H NMR (400 MHz, $CDCl_3$)

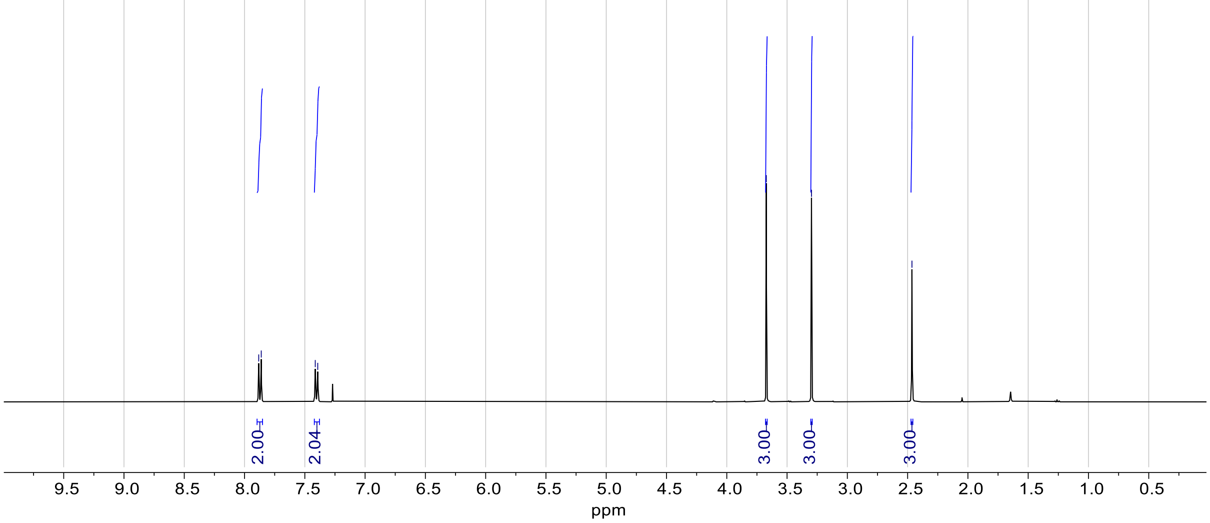

5

$^{13}C\{^1H\}$ NMR (101 MHz, $CDCl_3$)

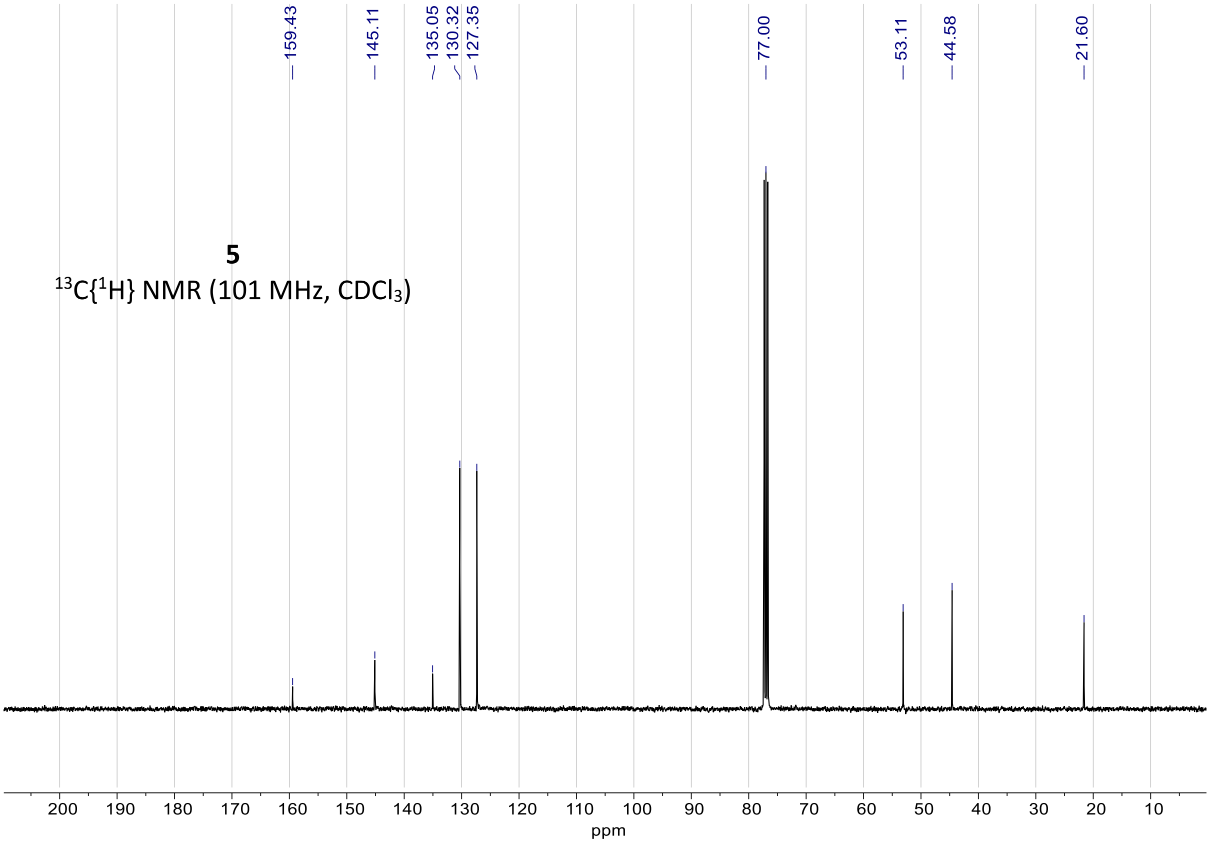

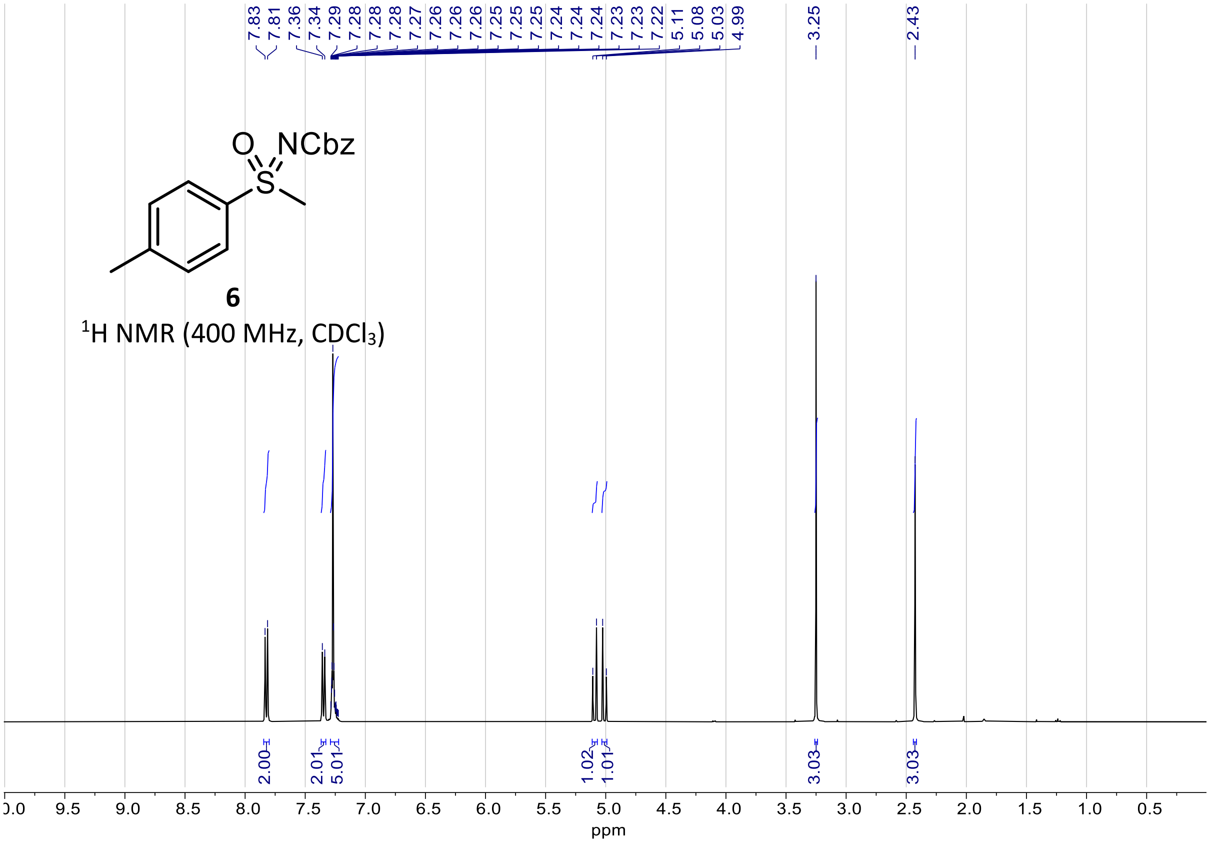

6

^{1}H NMR (400 MHz, $CDCl_3$)

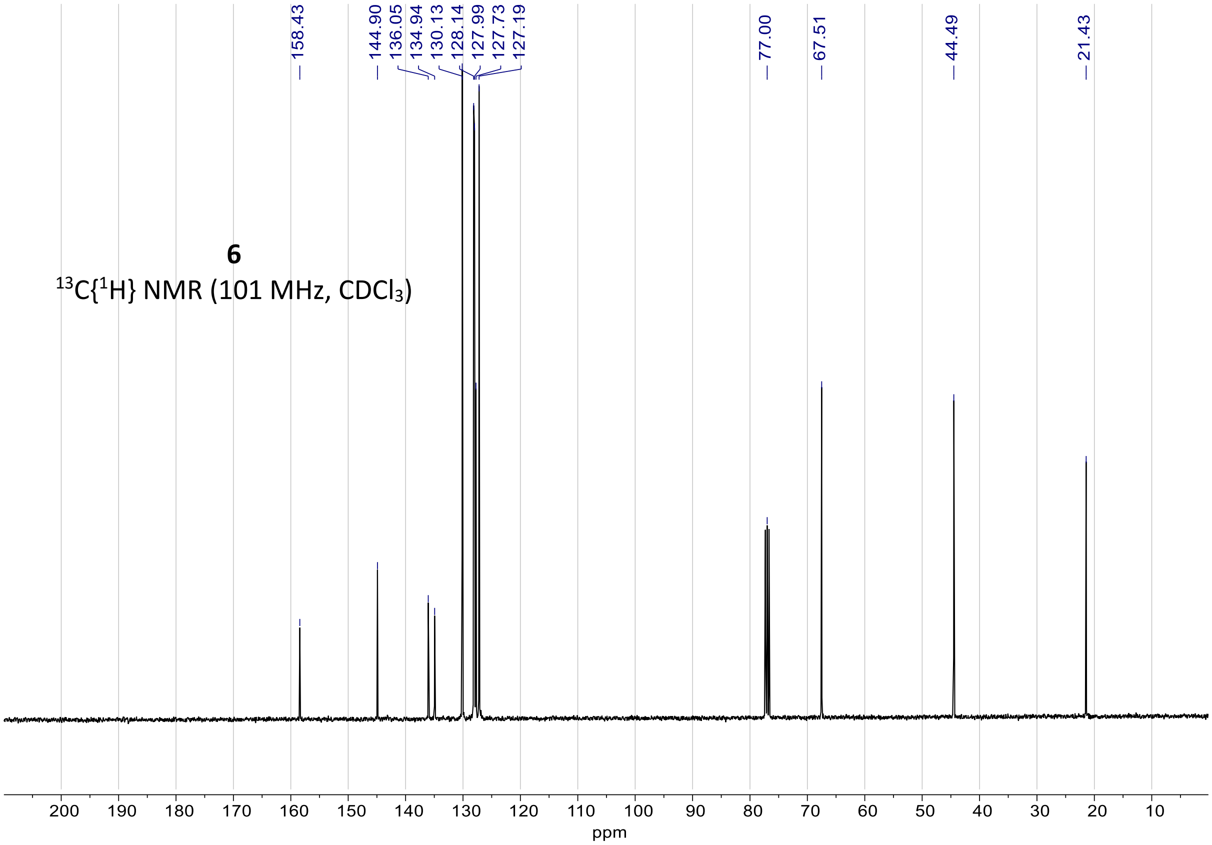

6

$^{13}C\{^{1}H\}$ NMR (101 MHz, $CDCl_3$)

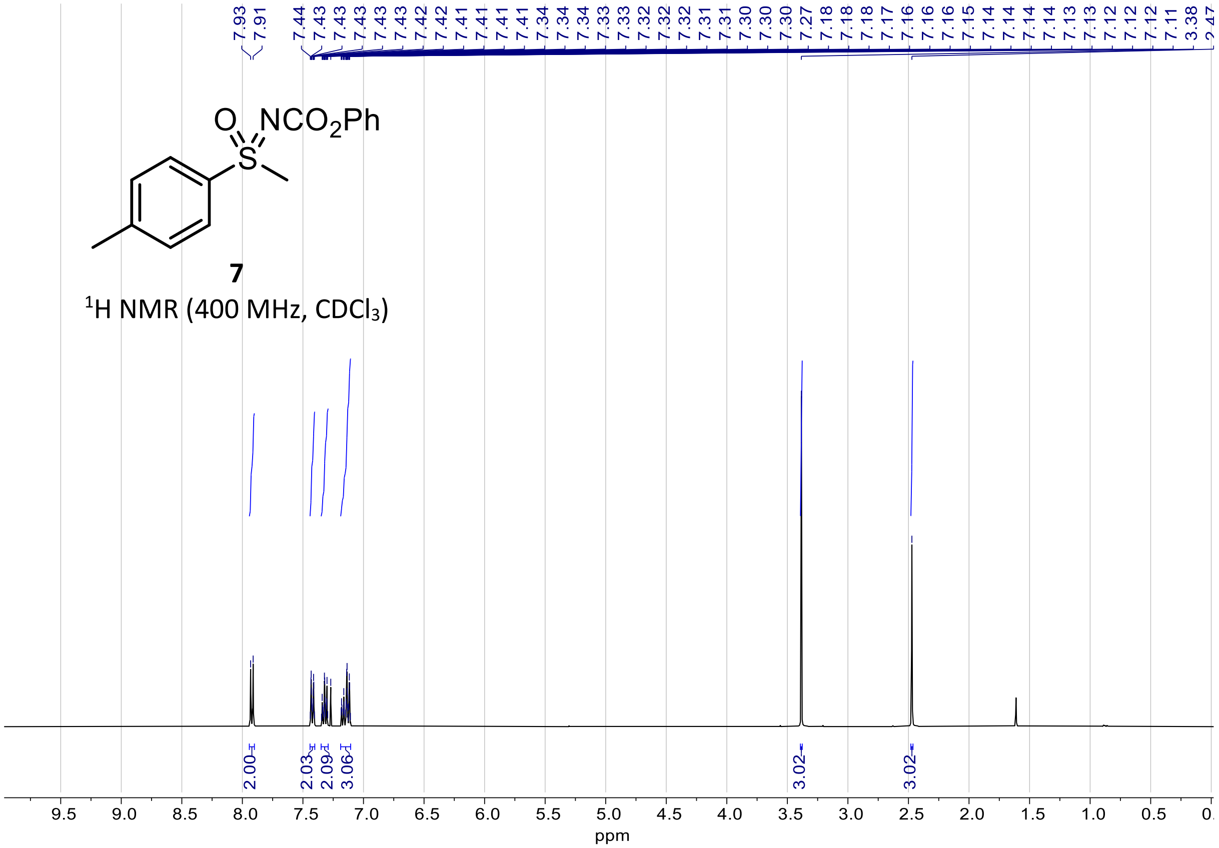
7.93
7.91
7.44
7.43
7.43
7.43
7.43
7.43
7.42
7.42
7.41
7.41
7.41
7.41
7.41
7.34
7.34
7.34
7.33
7.33
7.32
7.32
7.32
7.31
7.31
7.30
7.30
7.30
7.27
7.18
7.18
7.18
7.17
7.16
7.16
7.16
7.15
7.14
7.14
7.14
7.14
7.13
7.13
7.12
7.12
7.12
7.11
3.38
2.47
O
NCO2Ph
S
7
1H NMR (400 MHz, CDCl3)
2.00
2.03
2.09
3.06
3.02
3.02
ppm

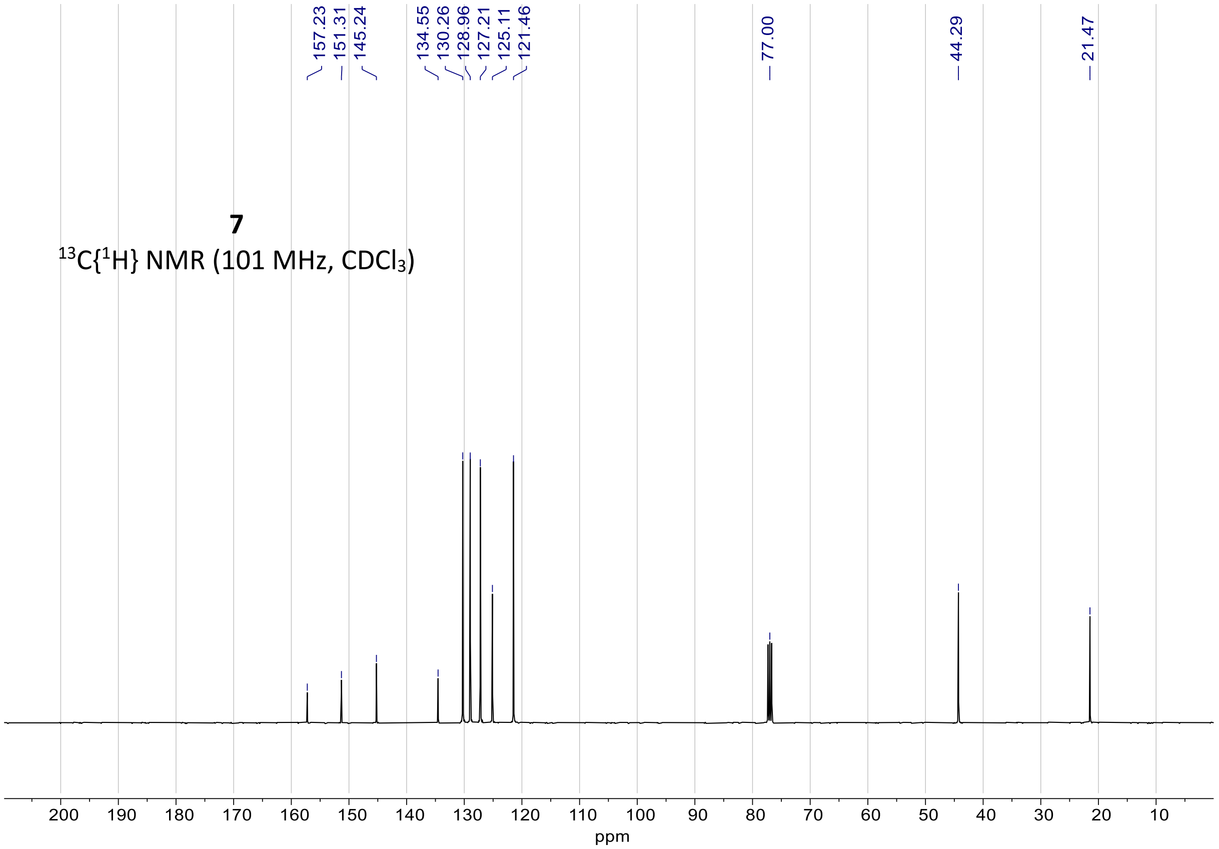
157.23
151.31
145.24
134.55
130.26
128.96
127.21
125.11
121.46
77.00
44.29
21.47
7
13C{1H} NMR (101 MHz, CDCl3)
ppm

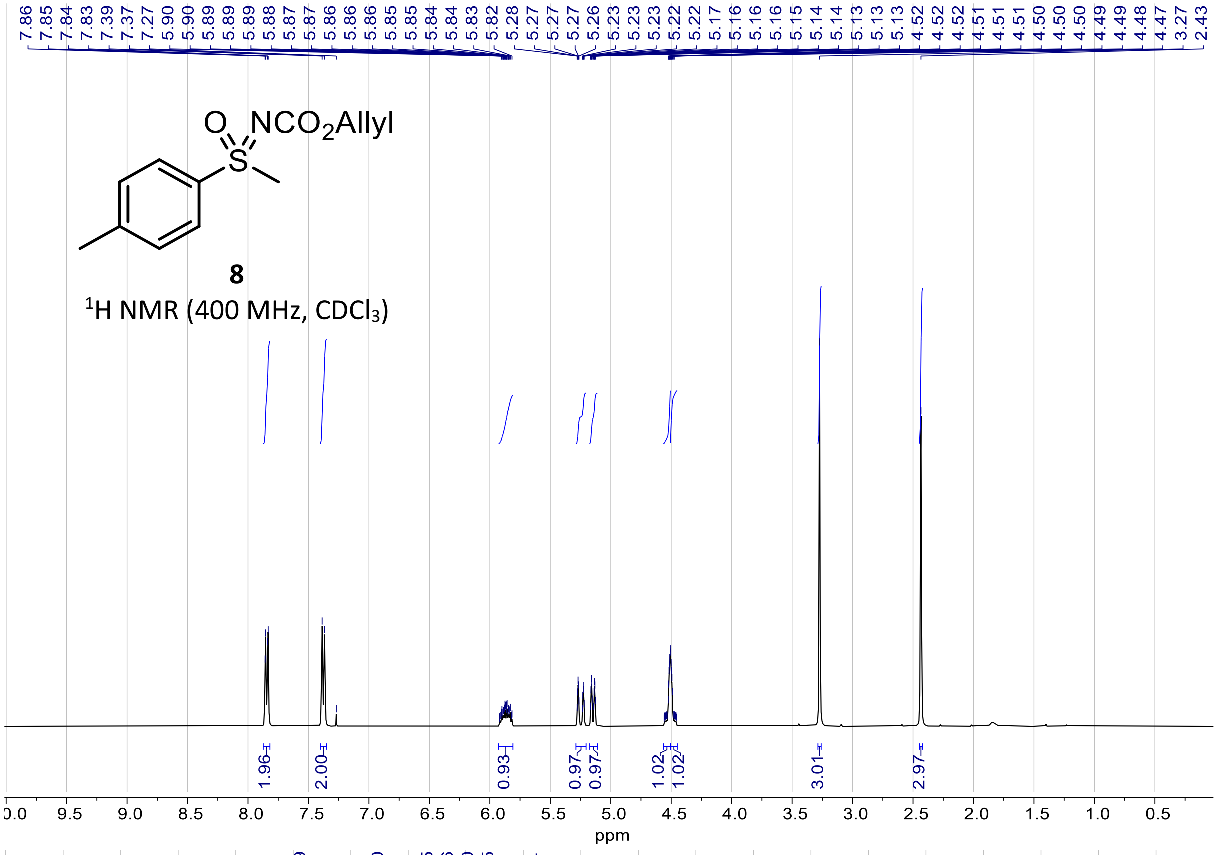
7.86 7.85 7.84 7.83 7.39 7.37 7.27 5.90 5.89 5.89 5.88 5.87 5.87 5.86 5.86 5.85 5.84 5.84 5.83 5.82 5.28 5.27 5.27 5.26 5.23 5.23 5.22 5.22 5.17 5.16 5.16 5.15 5.14 5.14 5.13 5.13 4.52 4.52 4.52 4.51 4.51 4.50 4.50 4.49 4.49 4.48 4.47 3.27 2.43
O NCO2Allyl
S
8
1H NMR (400 MHz, CDCl3)
1.96 2.00 0.93 0.97 0.97 1.02 1.02 3.01 2.97
0.0 9.5 9.0 8.5 8.0 7.5 7.0 6.5 6.0 5.5 5.0 4.5 4.0 3.5 3.0 2.5 2.0 1.5 1.0 0.5
ppm

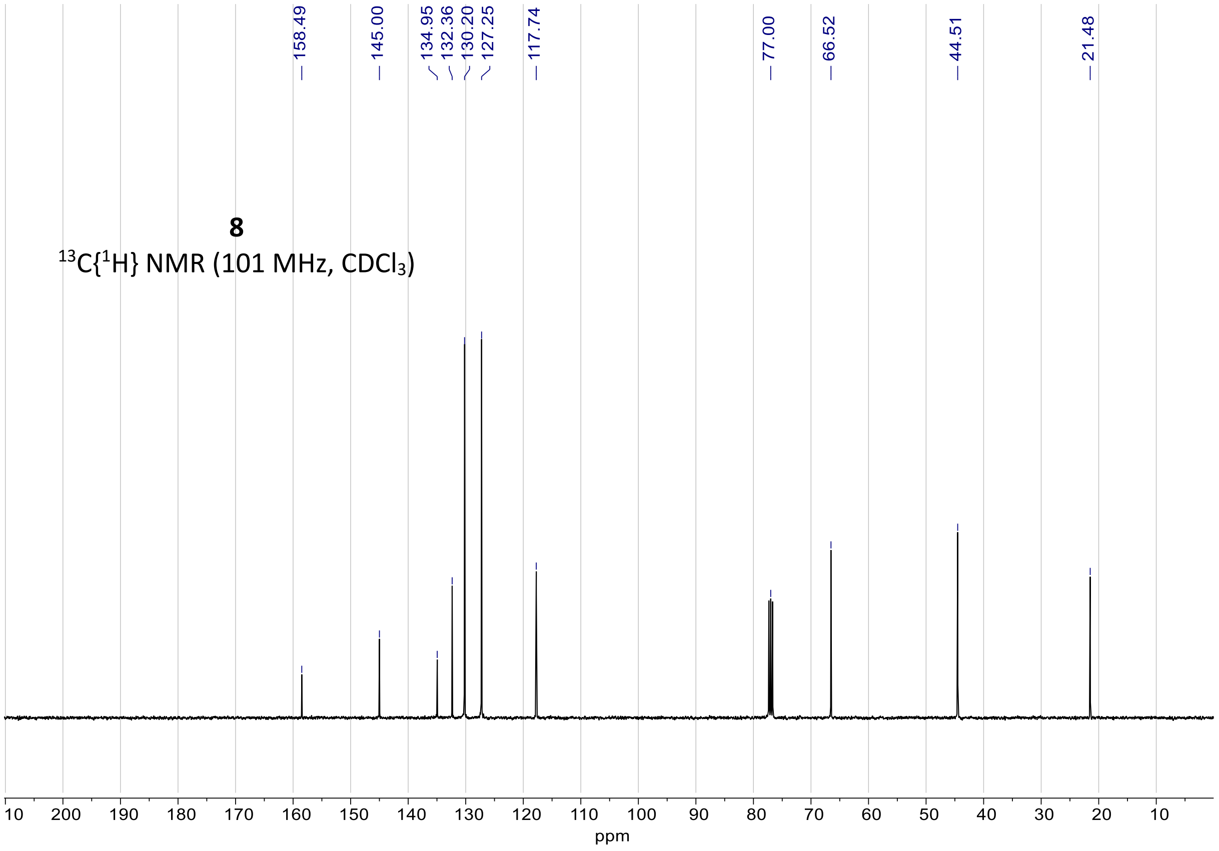
158.49
145.00
134.95 132.36 130.20 127.25
117.74
77.00
66.52
44.51
21.48
8
13C{1H} NMR (101 MHz, CDCl3)
10 200 190 180 170 160 150 140 130 120 110 100 90 80 70 60 50 40 30 20 10
ppm

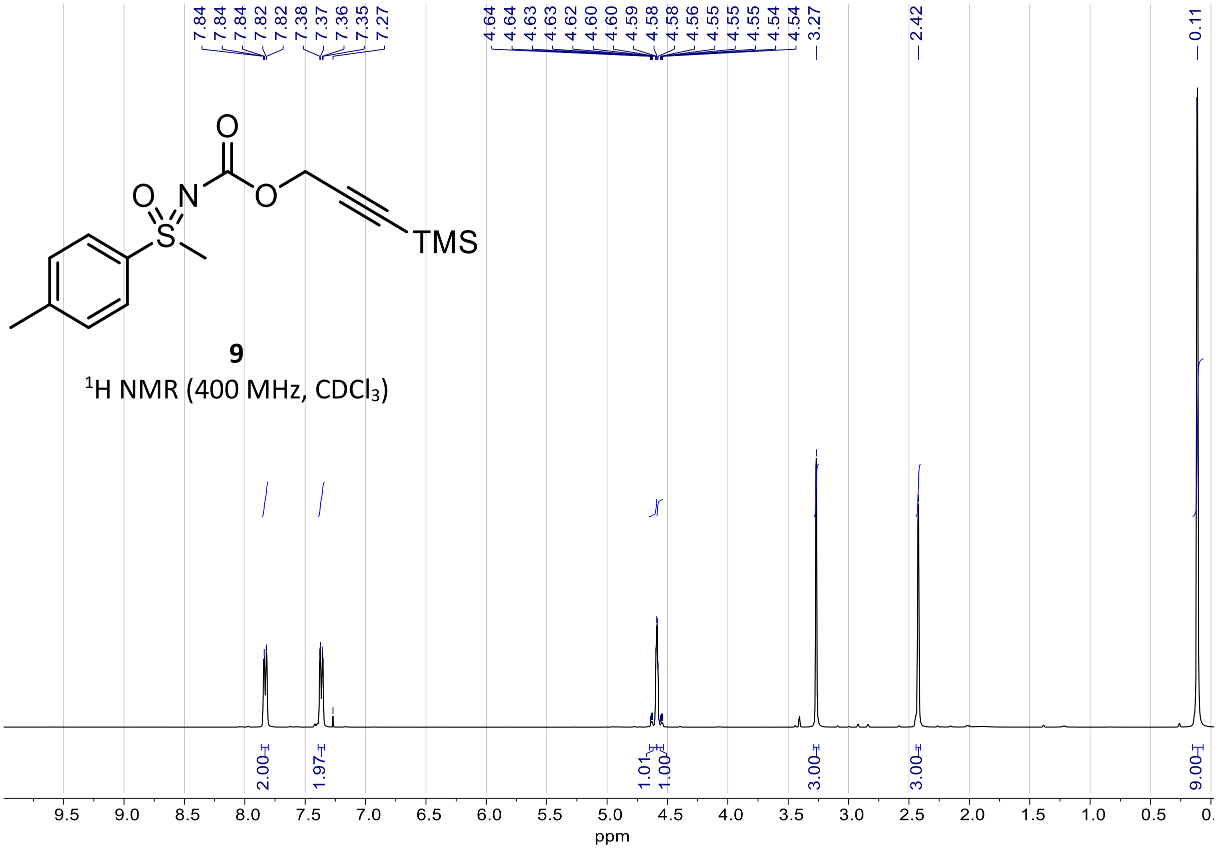
7.84 7.84 7.84 7.82 7.82 7.38 7.37 7.36 7.35 7.27
4.64 4.64 4.63 4.63 4.62 4.60 4.60 4.59 4.58 4.58 4.56 4.55 4.55 4.55 4.54 4.54
3.27
2.42
0.11
O
N
O
S
O
TMS
9
1H NMR (400 MHz, CDCl3)
2.00
1.97
1.01
1.00
3.00
3.00
9.00
ppm

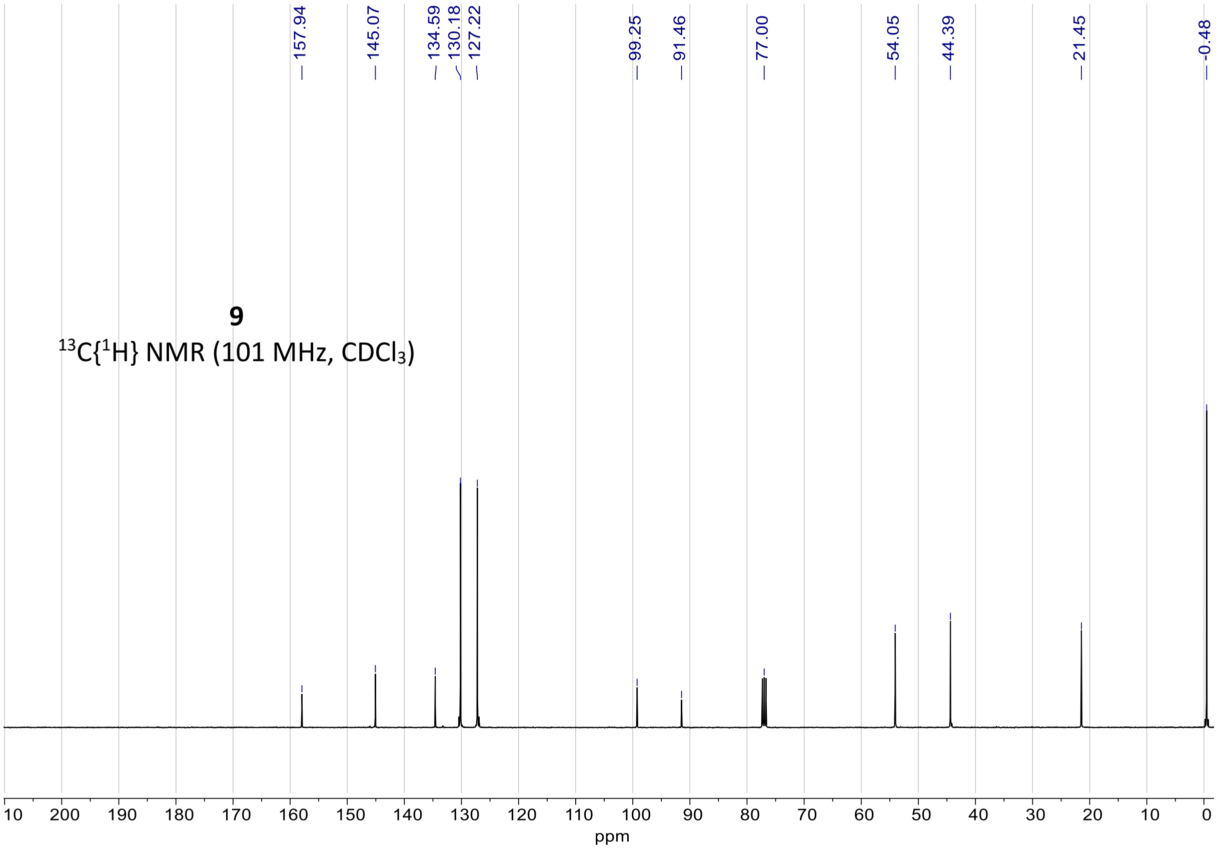
157.94
145.07
134.59
130.18
127.22
99.25
91.46
77.00
54.05
44.39
21.45
-0.48
9
13C{1H} NMR (101 MHz, CDCl3)
ppm

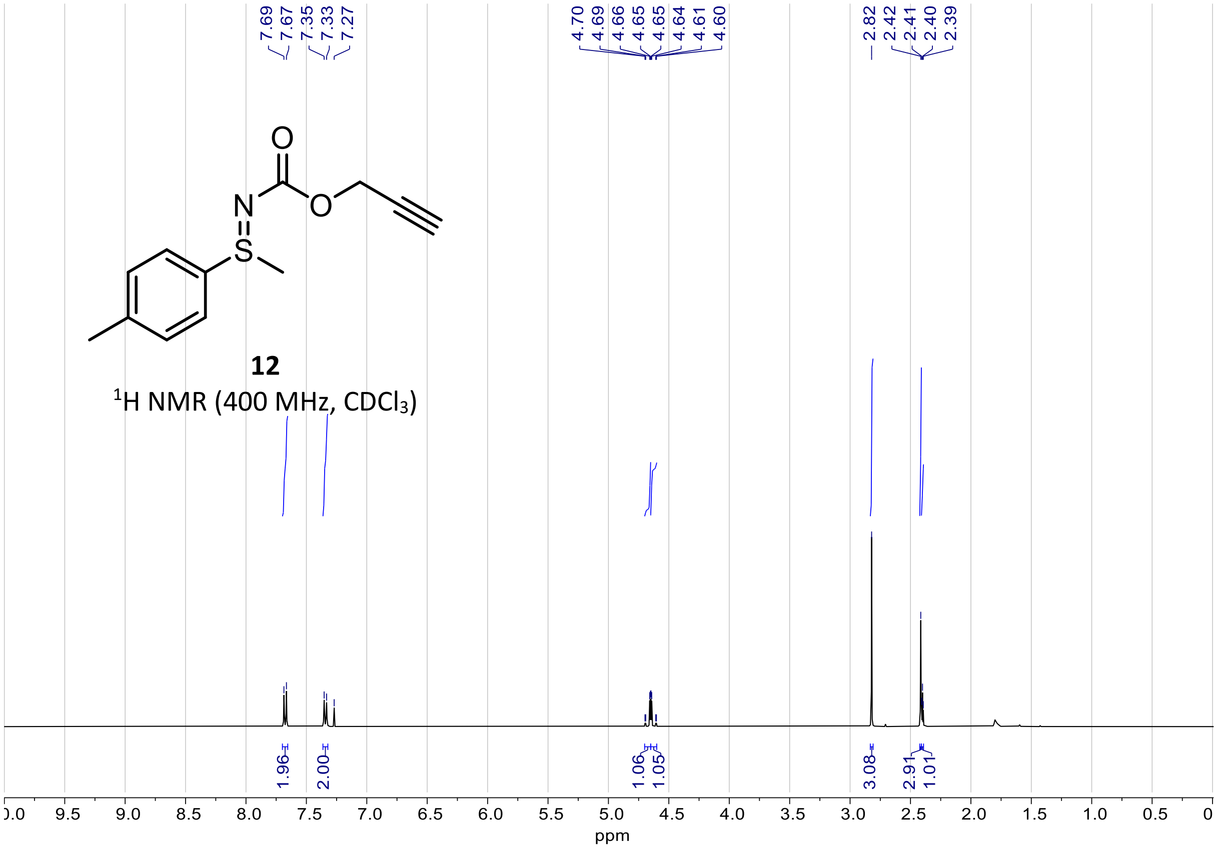
12
^{1}H NMR (400 MHz, $CDCl_3$)
7.69 7.67 7.35 7.33 7.27
4.70 4.69 4.66 4.65 4.65 4.64 4.61 4.60
2.82 2.42 2.41 2.40 2.39
1.96 2.00 1.06 1.05 3.08 2.91 1.01
ppm

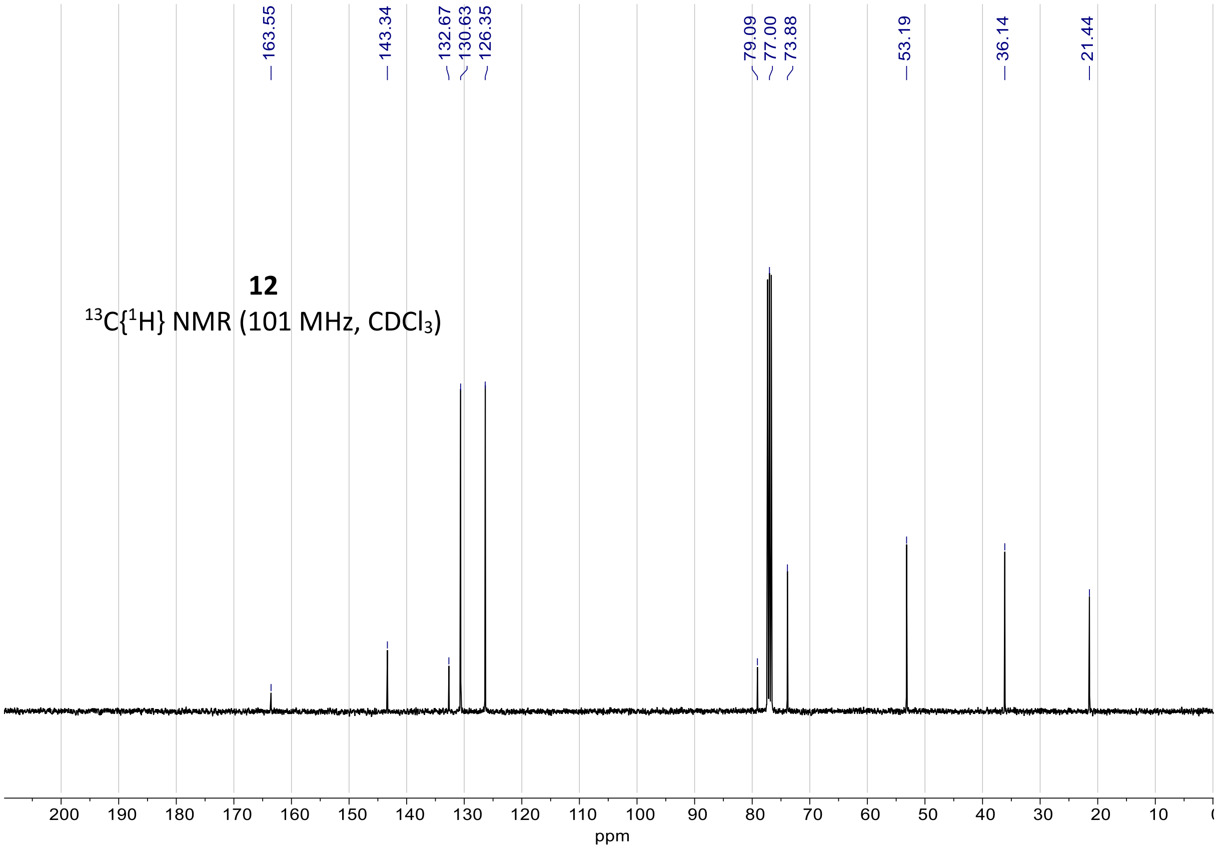
12
^{13}C{^{1}H} NMR (101 MHz, $CDCl_3$)
163.55 143.34 132.67 130.63 126.35 79.09 77.00 73.88 53.19 36.14 21.44
ppm

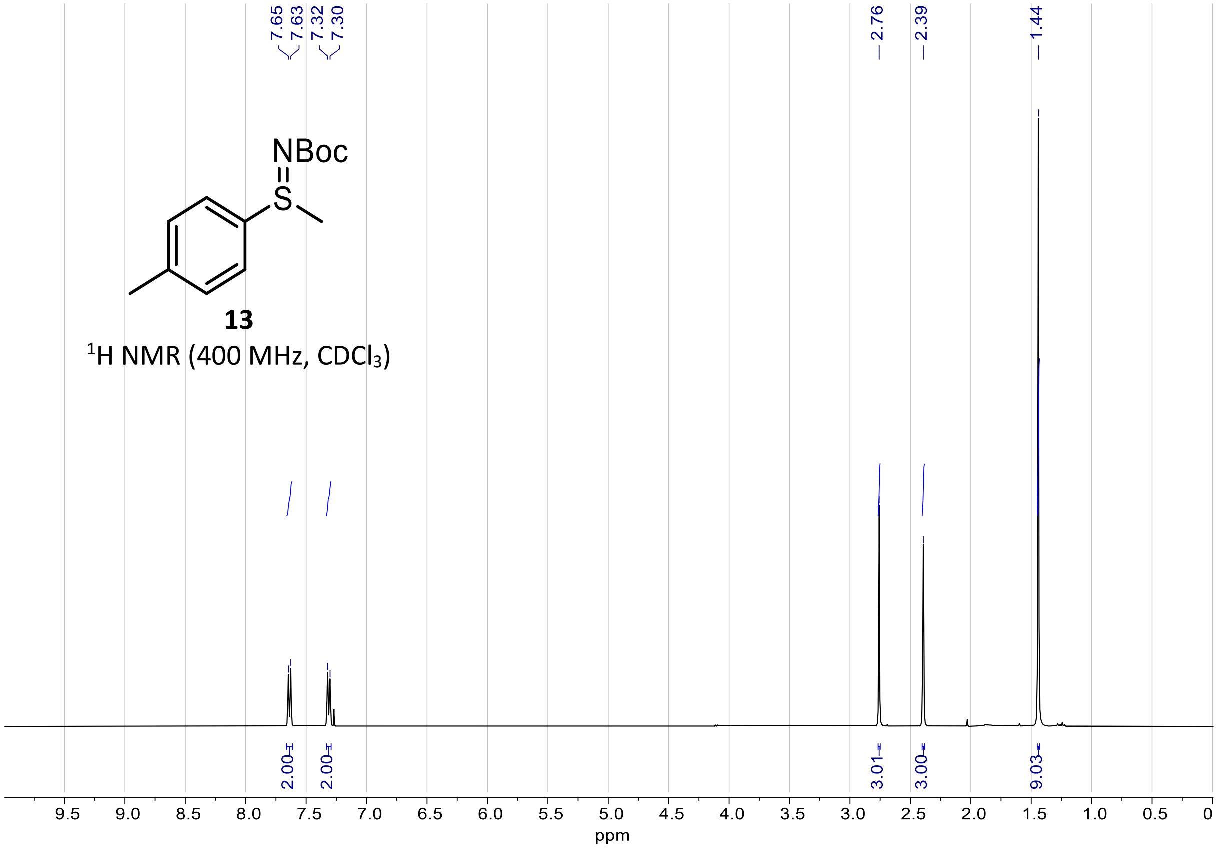
7.65
7.63
7.32
7.30
2.76
2.39
1.44
NBoc
S
13
^{1}H NMR (400 MHz, $CDCl_3$)
2.00
2.00
3.01
3.00
9.03
9.5 9.0 8.5 8.0 7.5 7.0 6.5 6.0 5.5 5.0 4.5 4.0 3.5 3.0 2.5 2.0 1.5 1.0 0.5 0
ppm

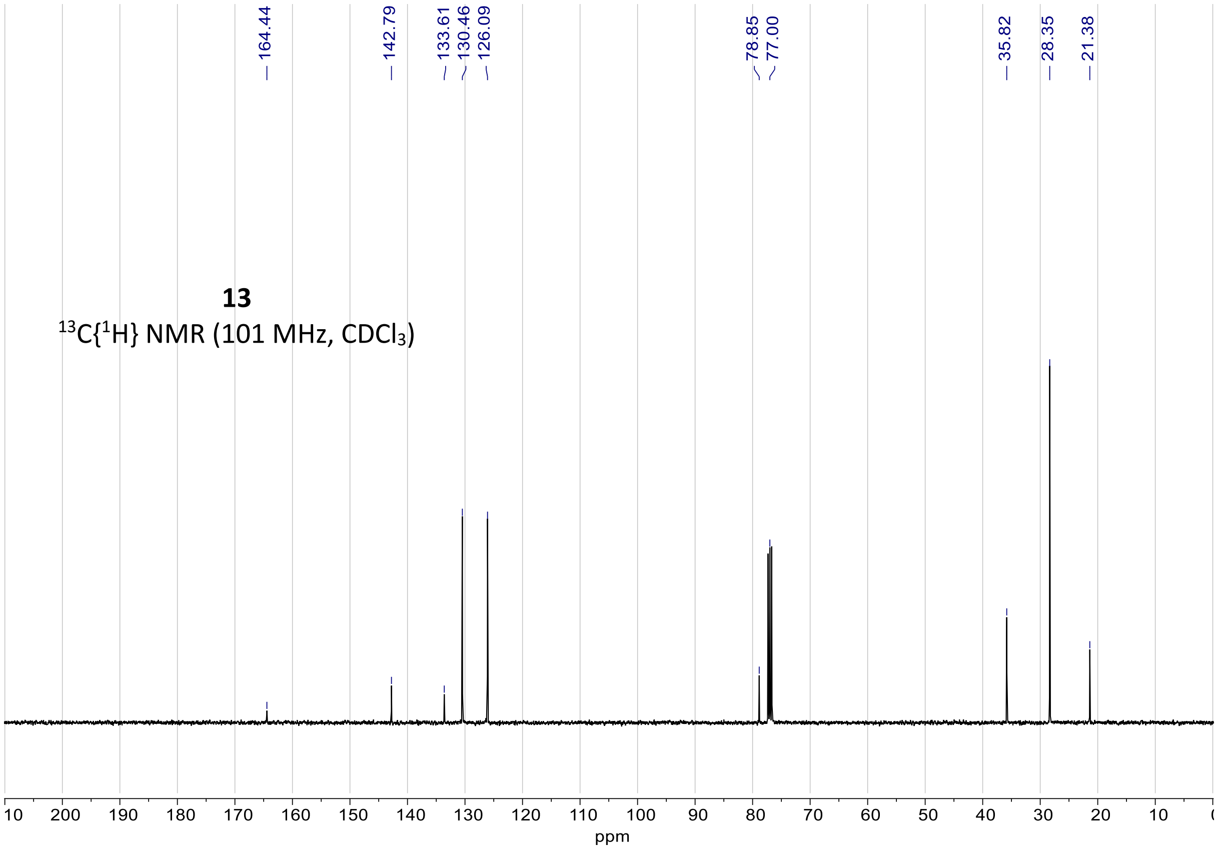
164.44
142.79
133.61
130.46
126.09
78.85
77.00
35.82
28.35
21.38
13
$^{13}C\{^{1}H\}$ NMR (101 MHz, $CDCl_3$)
10 200 190 180 170 160 150 140 130 120 110 100 90 80 70 60 50 40 30 20 10
ppm

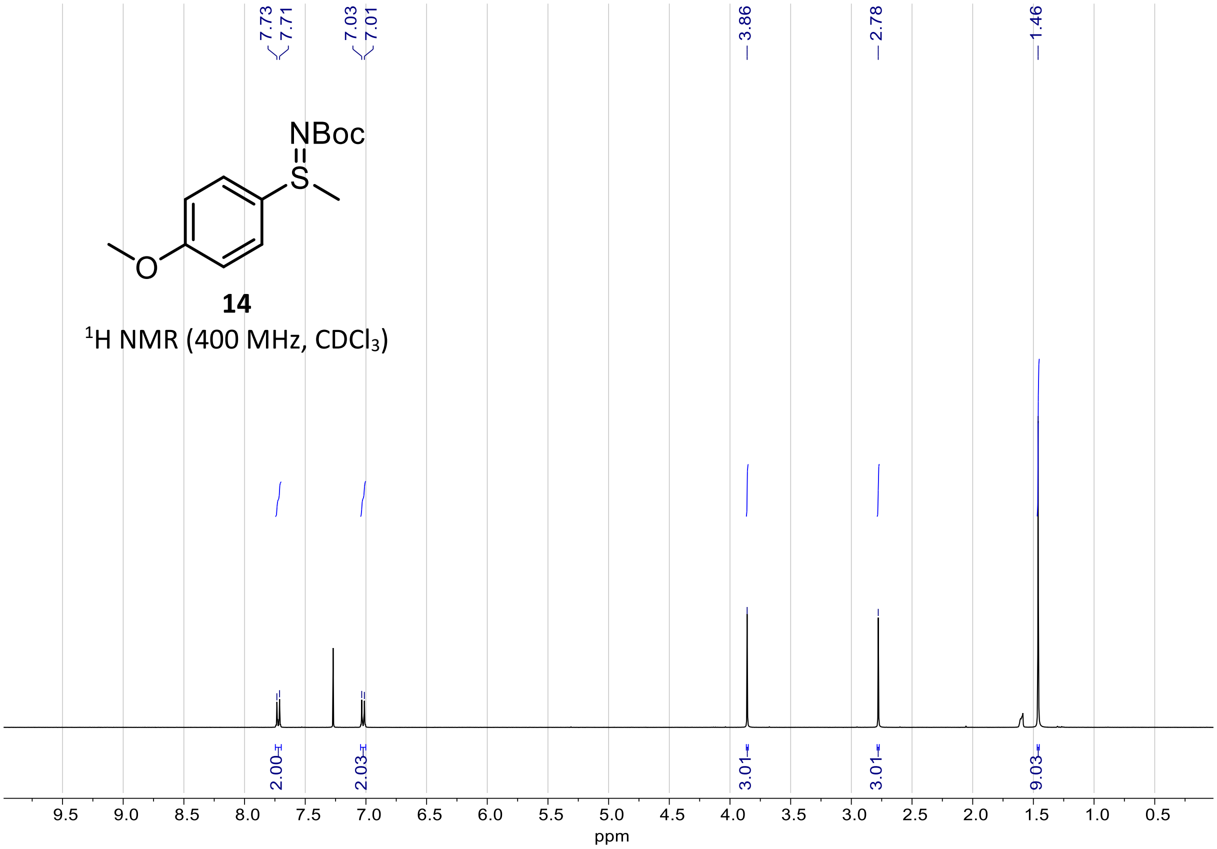
NBoc
S
O
14
^{1}H NMR (400 MHz, CDCl$_3$)
7.73
7.71
7.03
7.01
3.86
2.78
1.46
2.00
2.03
3.01
3.01
9.03
9.5 9.0 8.5 8.0 7.5 7.0 6.5 6.0 5.5 5.0 4.5 4.0 3.5 3.0 2.5 2.0 1.5 1.0 0.5
ppm

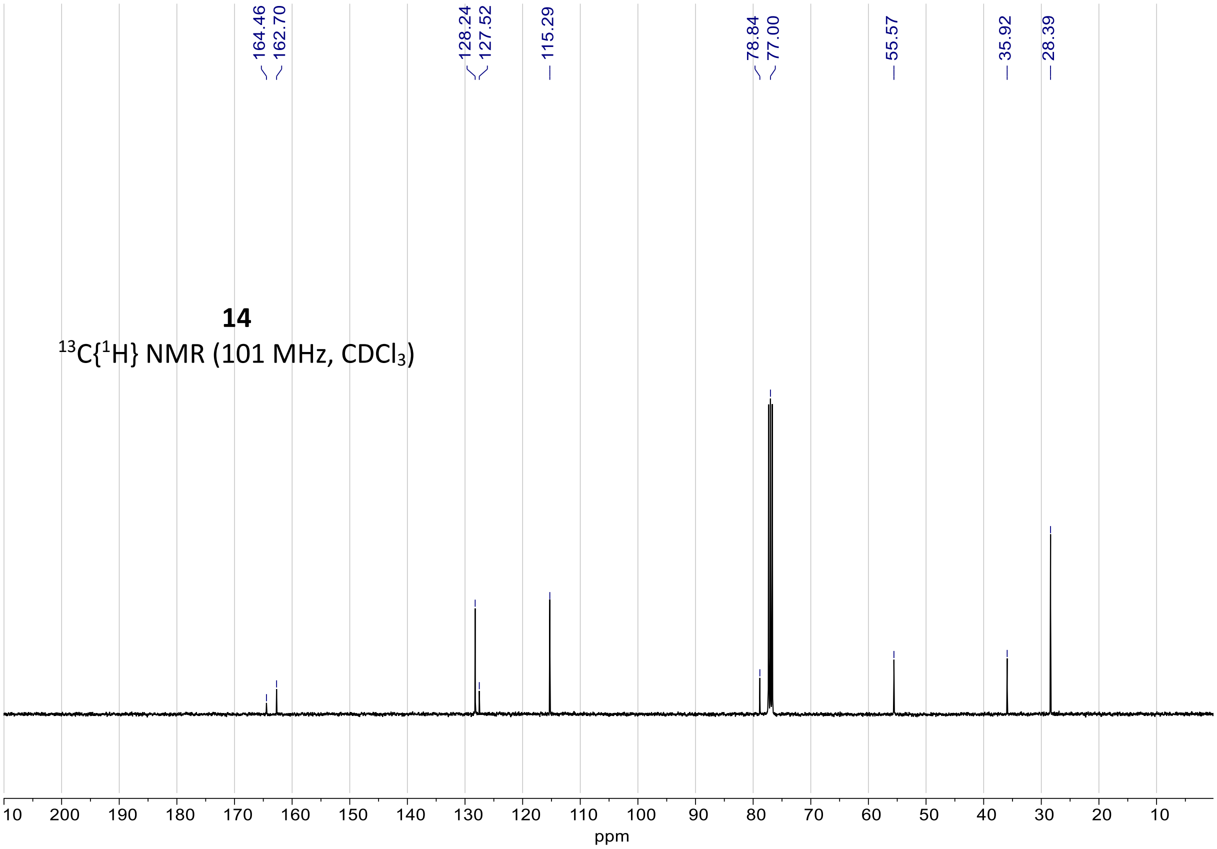
14
^{13}C{^{1}H} NMR (101 MHz, CDCl$_3$)
164.46
162.70
128.24
127.52
115.29
78.84
77.00
55.57
35.92
28.39
10 200 190 180 170 160 150 140 130 120 110 100 90 80 70 60 50 40 30 20 10
ppm

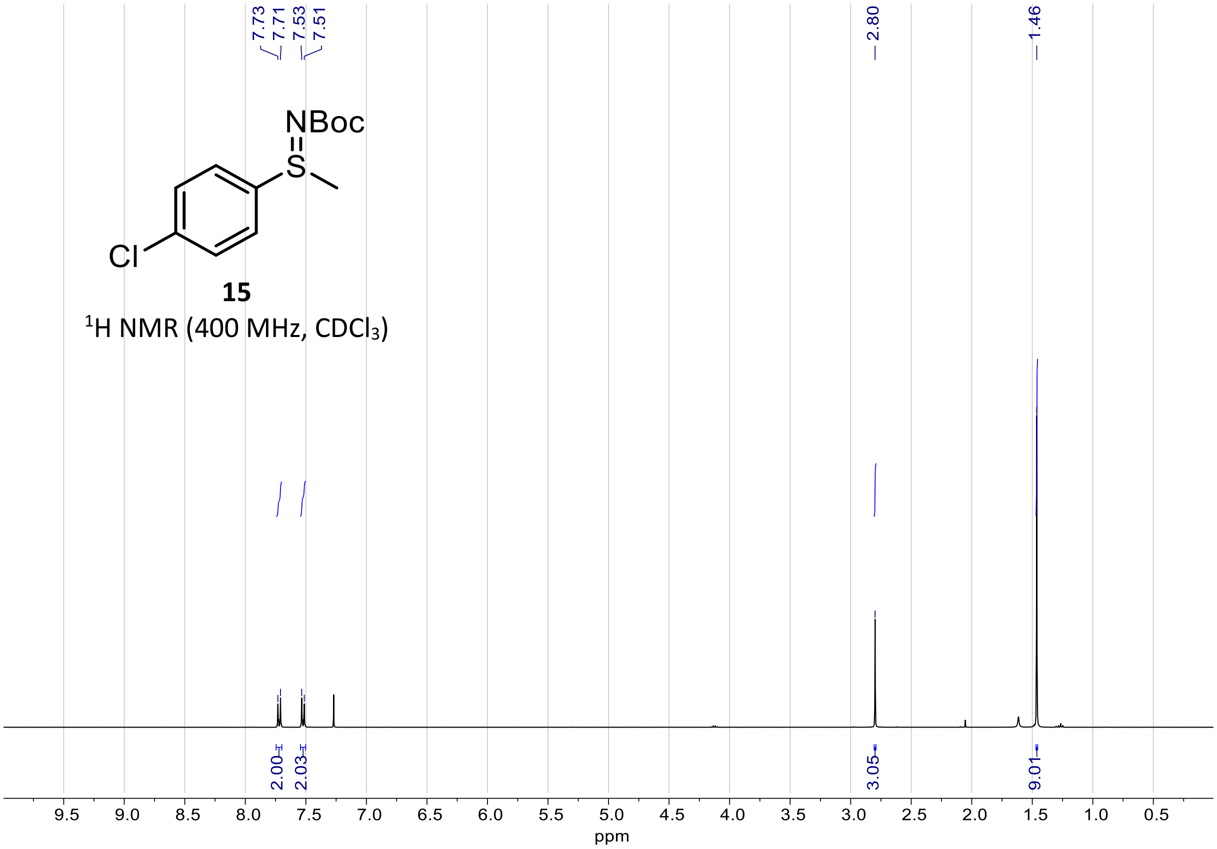

15

^{1}H NMR (400 MHz, $CDCl_3$)

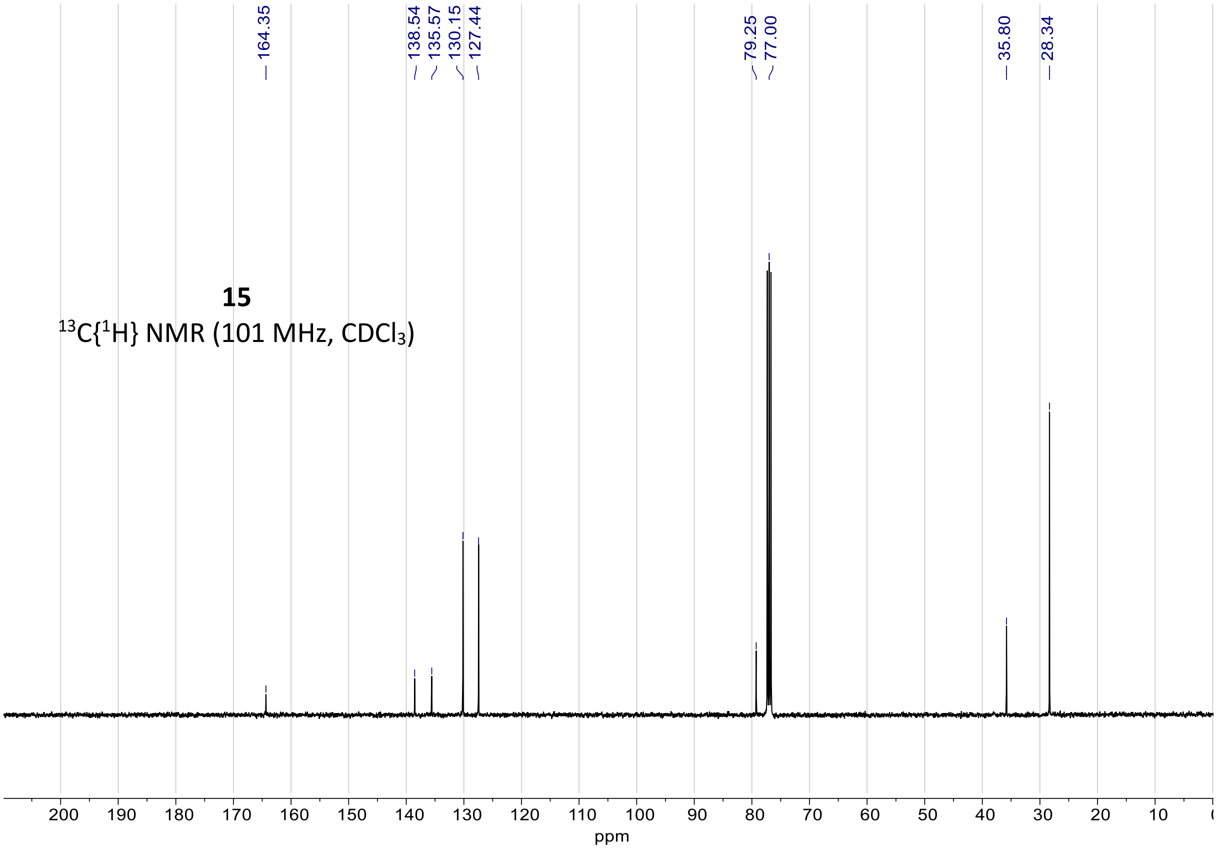

15

$^{13}C\{^1H\}$ NMR (101 MHz, $CDCl_3$)

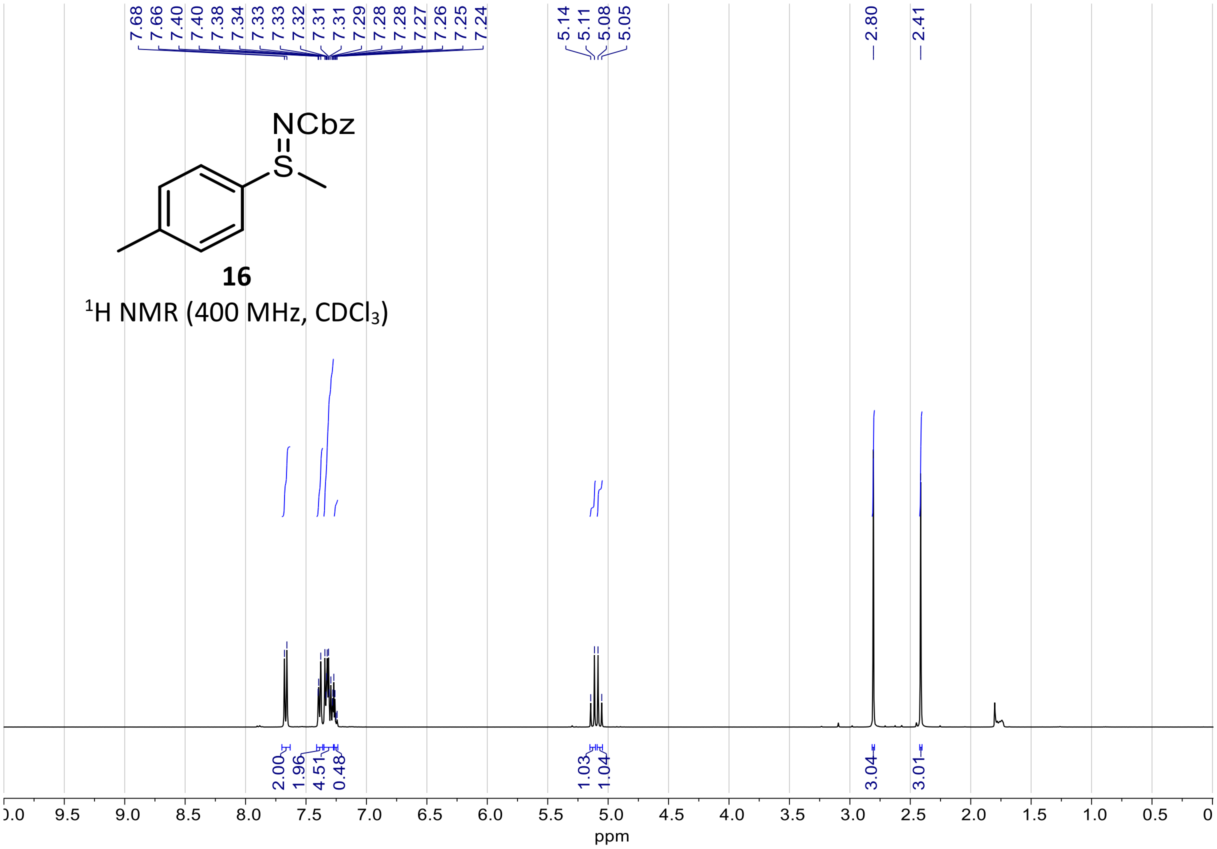
7.68 7.66 7.40 7.40 7.38 7.34 7.33 7.33 7.32 7.31 7.31 7.29 7.28 7.28 7.27 7.26 7.25 7.24
5.14 5.11 5.08 5.05
2.80
2.41
NCbz
S
16
1H NMR (400 MHz, CDCl3)
2.00 1.96 4.51 0.48
1.03 1.04
3.04
3.01
ppm

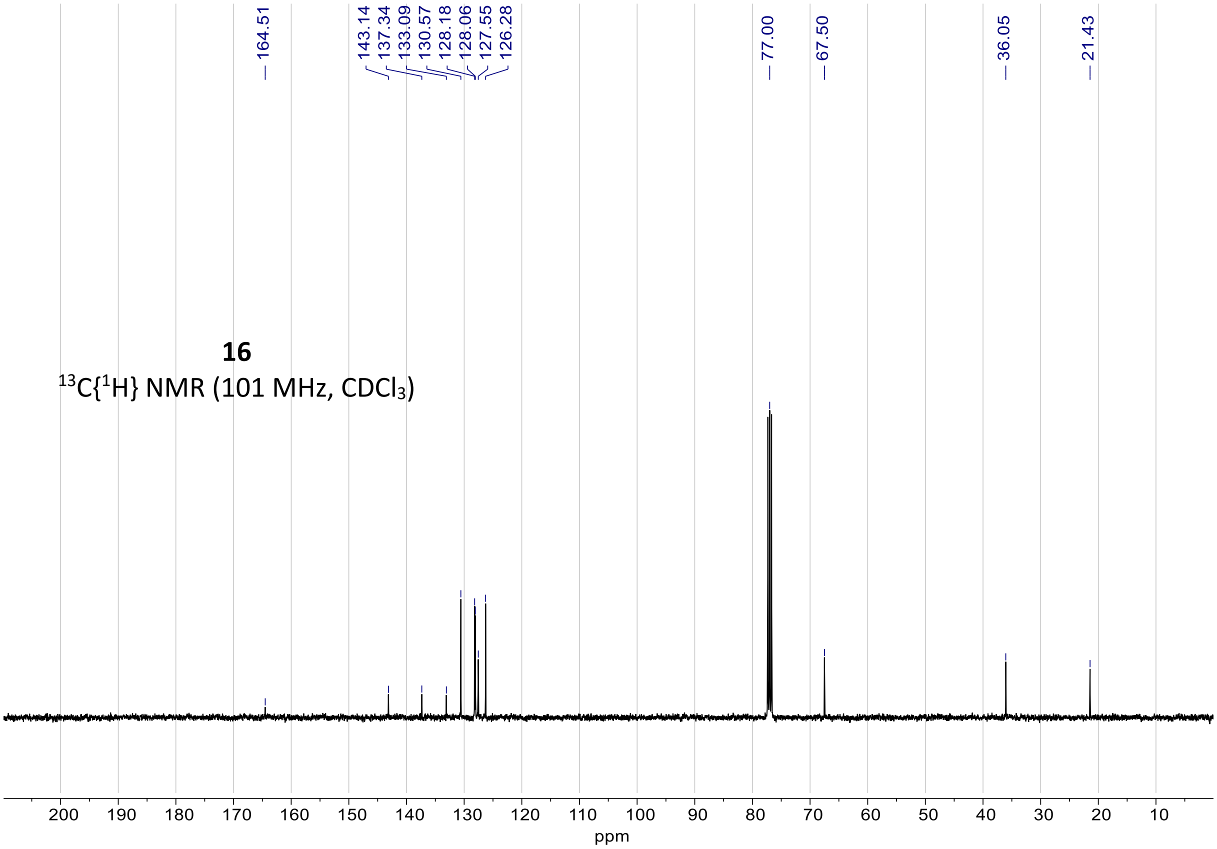
164.51
143.14 137.34 133.09 130.57 128.18 128.06 127.55 126.28
77.00
67.50
36.05
21.43
16
13C{1H} NMR (101 MHz, CDCl3)
ppm

17

^{1}H NMR (400 MHz, $CDCl_3$)

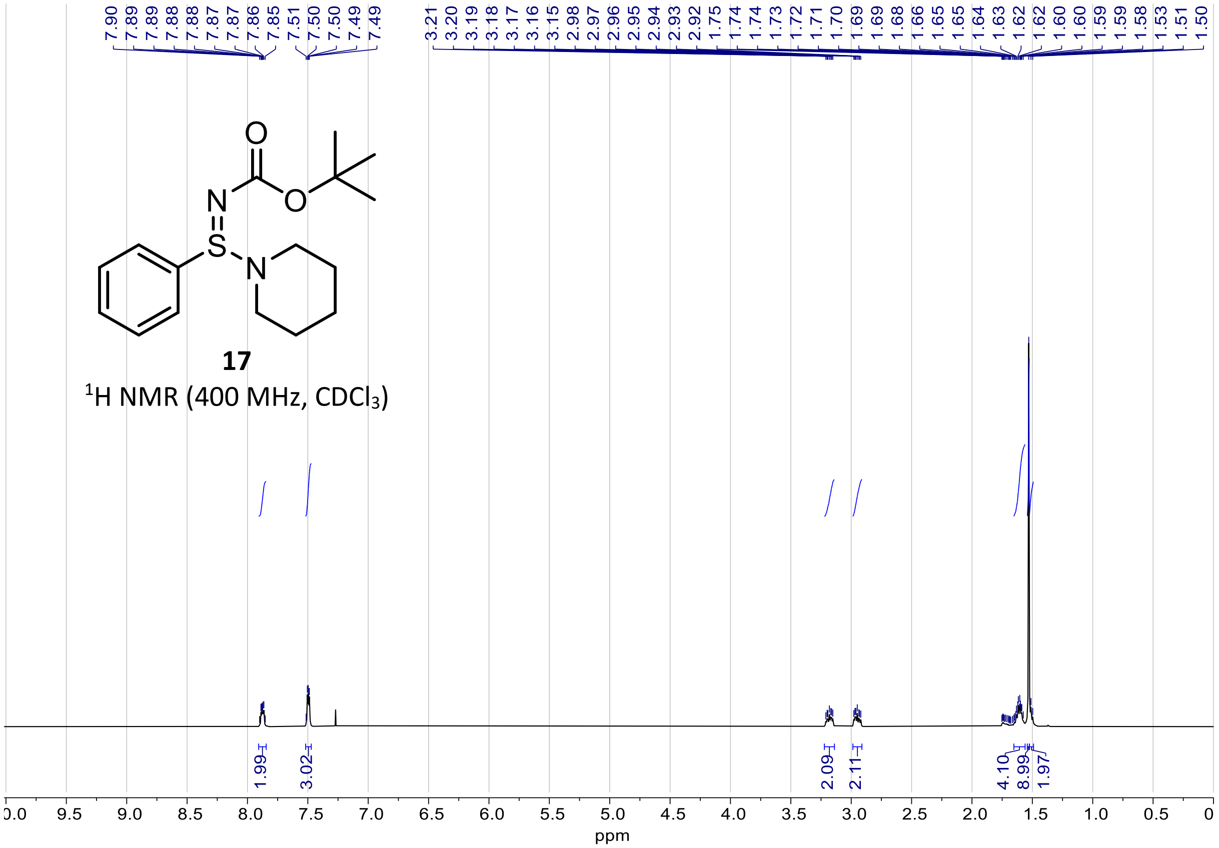

17

$^{13}C\{^{1}H\}$ NMR (101 MHz, $CDCl_3$)

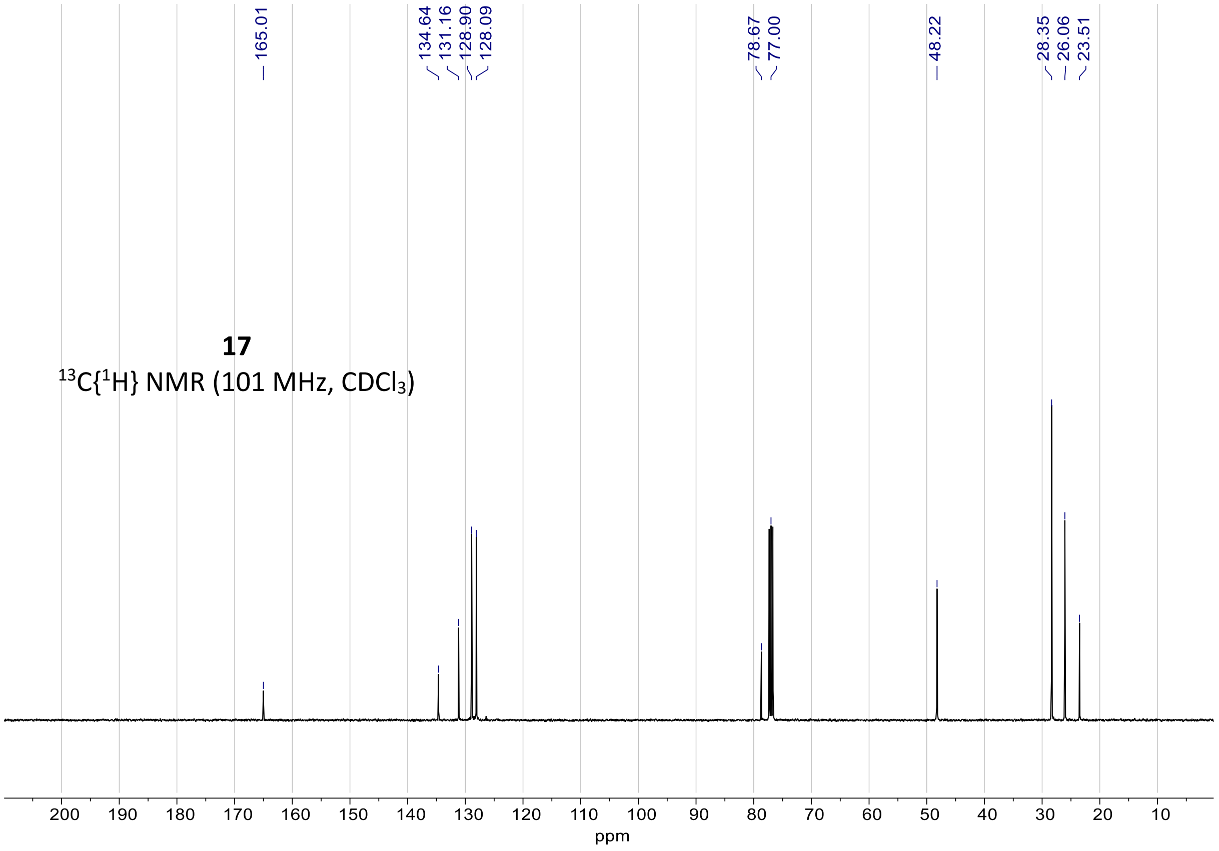

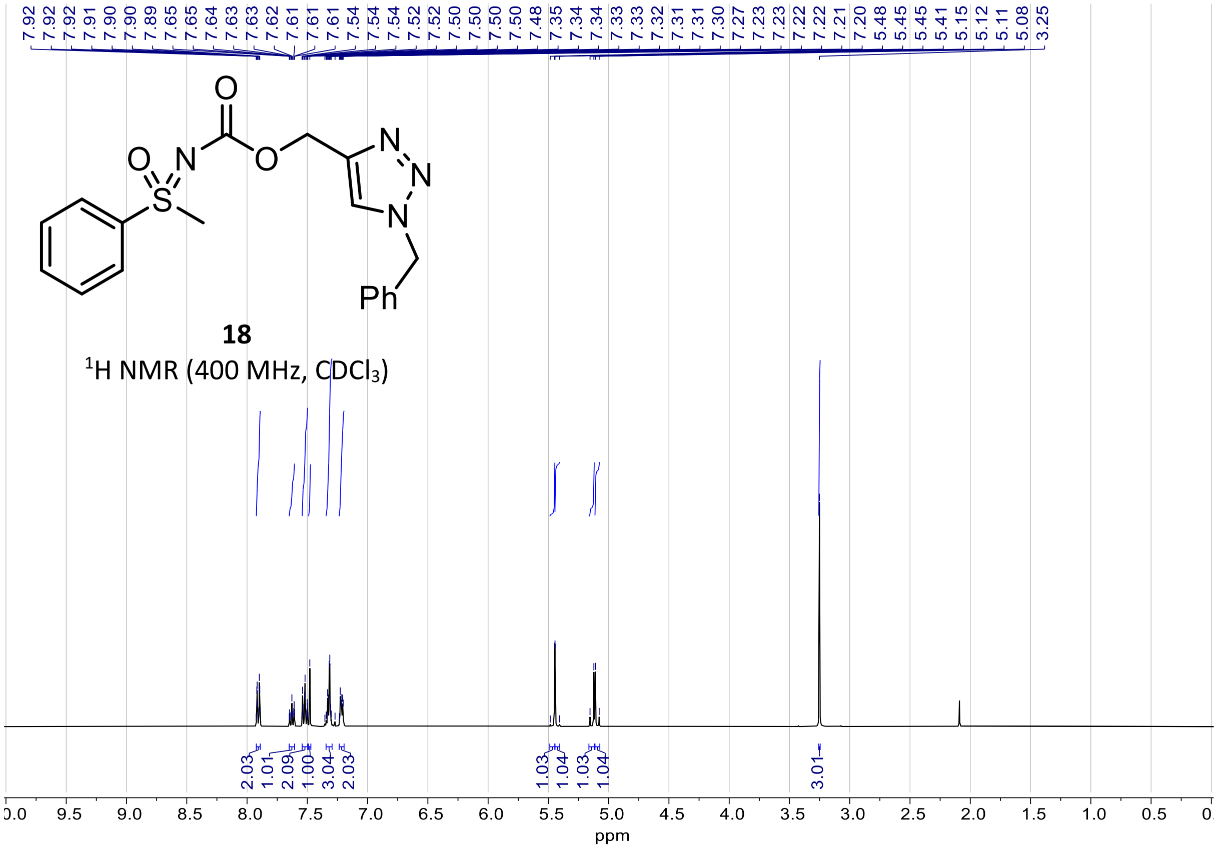
7.92 7.92 7.92 7.91 7.90 7.89 7.65 7.65 7.64 7.63 7.63 7.62 7.61 7.61 7.61 7.54 7.54 7.54 7.52 7.52 7.50 7.50 7.50 7.50 7.48 7.35 7.34 7.34 7.33 7.33 7.32 7.31 7.31 7.30 7.27 7.23 7.23 7.22 7.22 7.21 7.20 5.48 5.45 5.45 5.41 5.15 5.12 5.11 5.08 3.25
18
^{1}H NMR (400 MHz, CDCl$_3$)
ppm

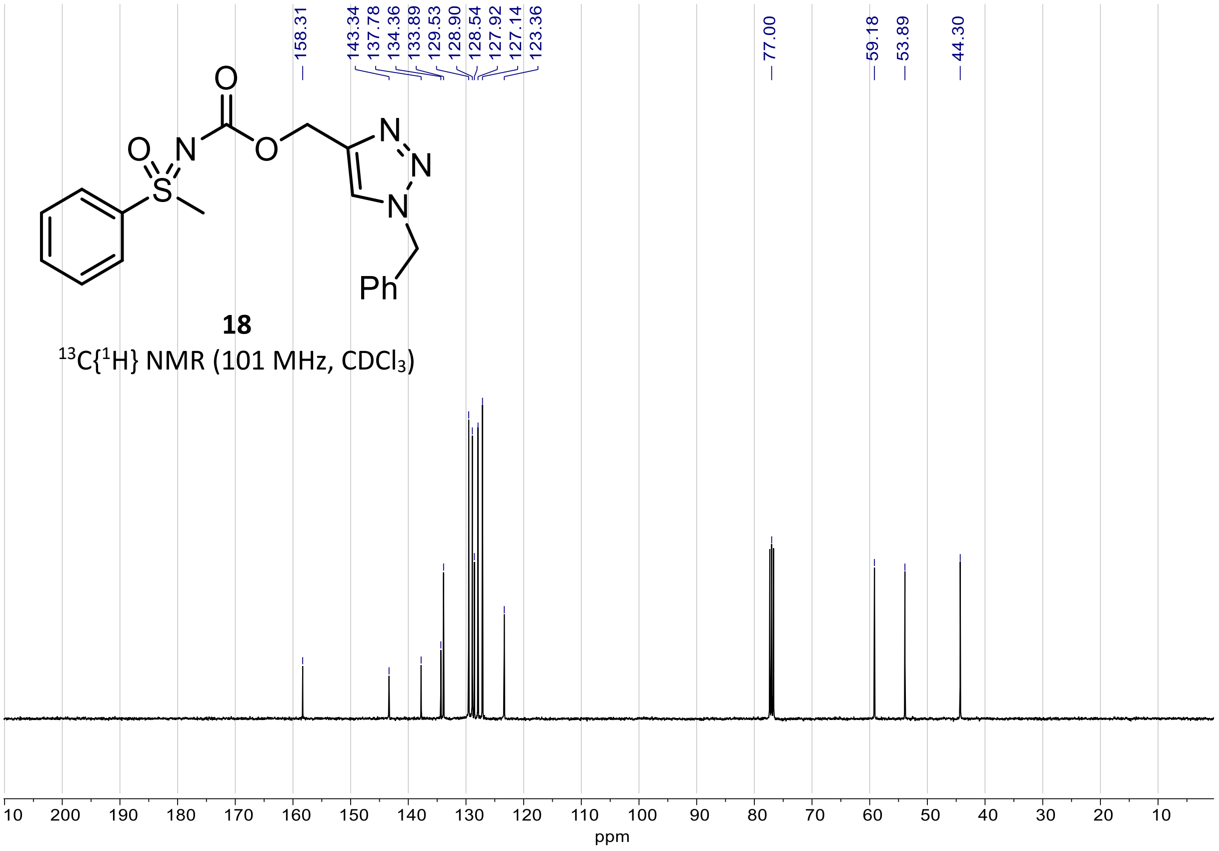
158.31 143.34 137.78 134.36 133.89 129.53 128.90 128.54 127.92 127.14 123.36 77.00 59.18 53.89 44.30
18
^{13}C{^{1}H} NMR (101 MHz, CDCl$_3$)
ppm

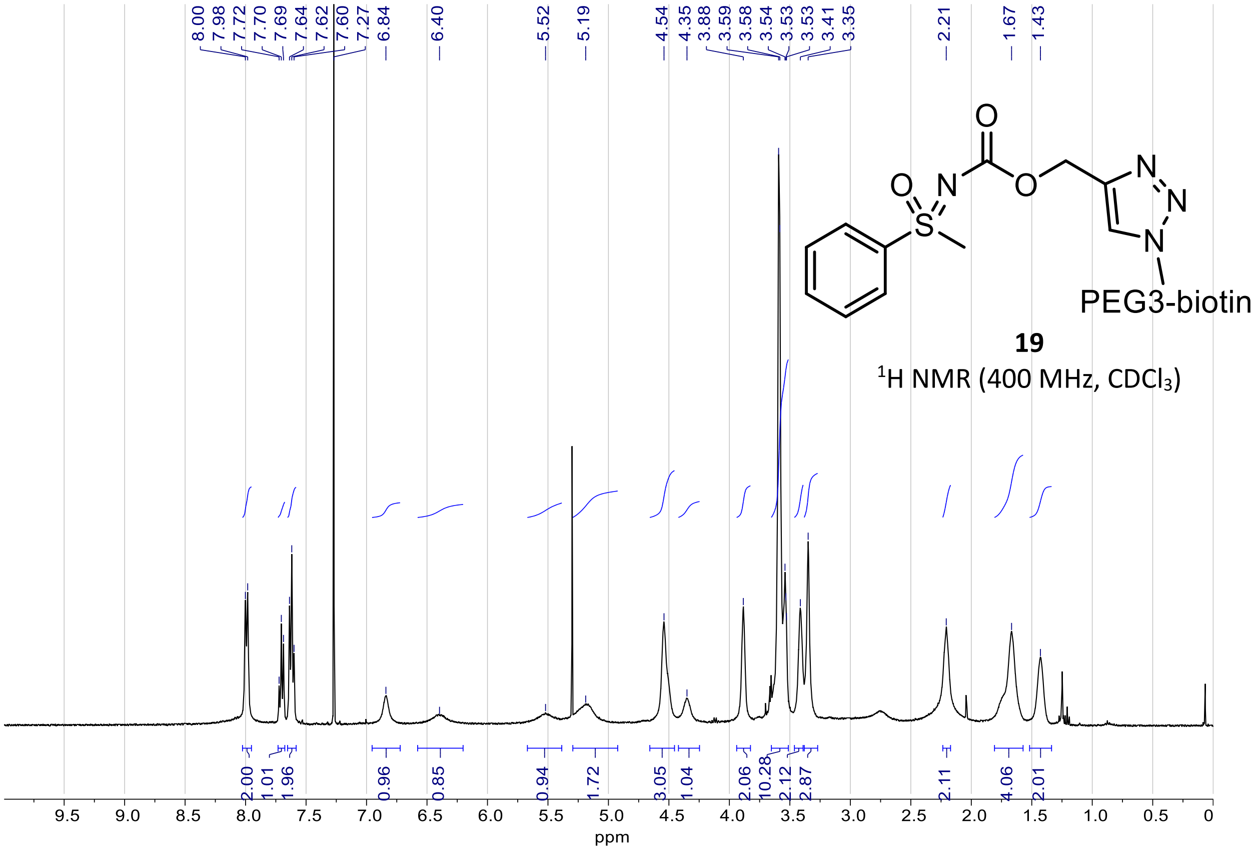

19

^{1}H NMR (400 MHz, $CDCl_3$)

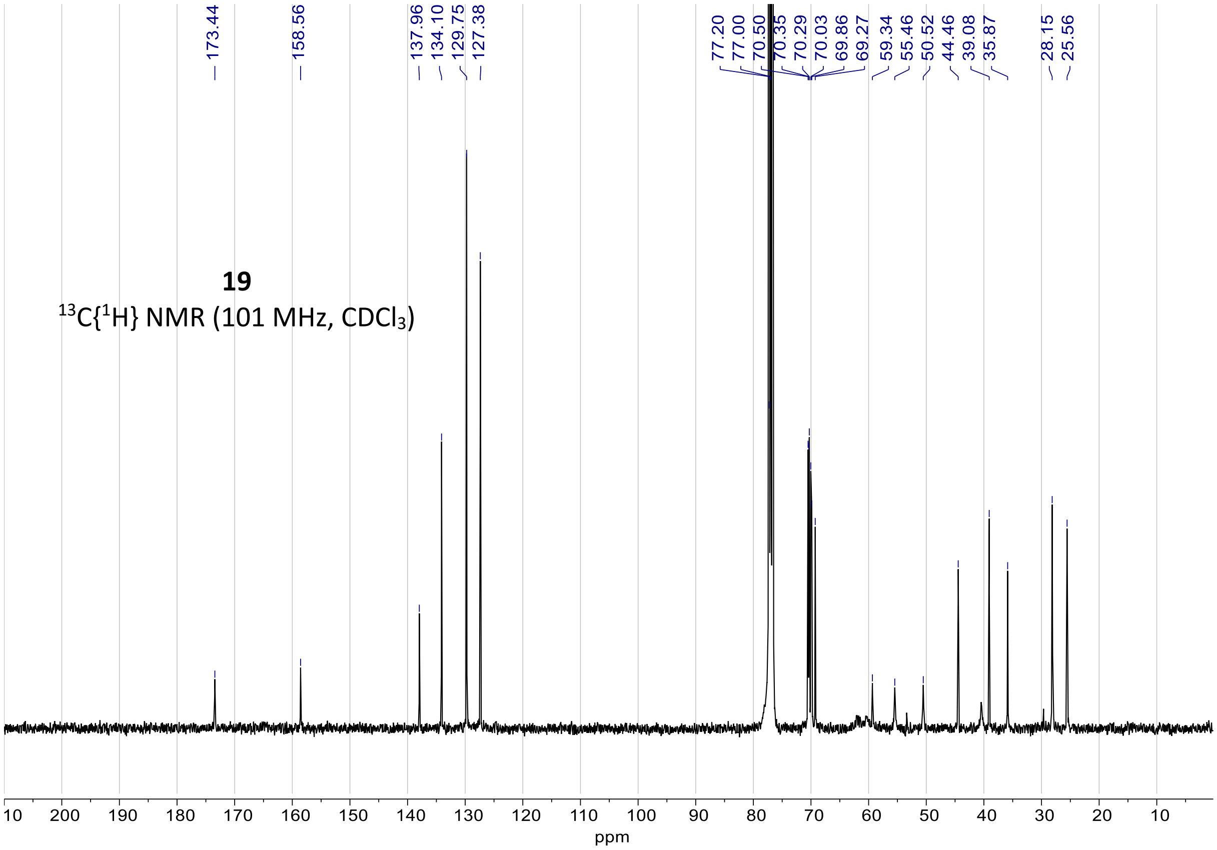

19

$^{13}C\{^{1}H\}$ NMR (101 MHz, $CDCl_3$)

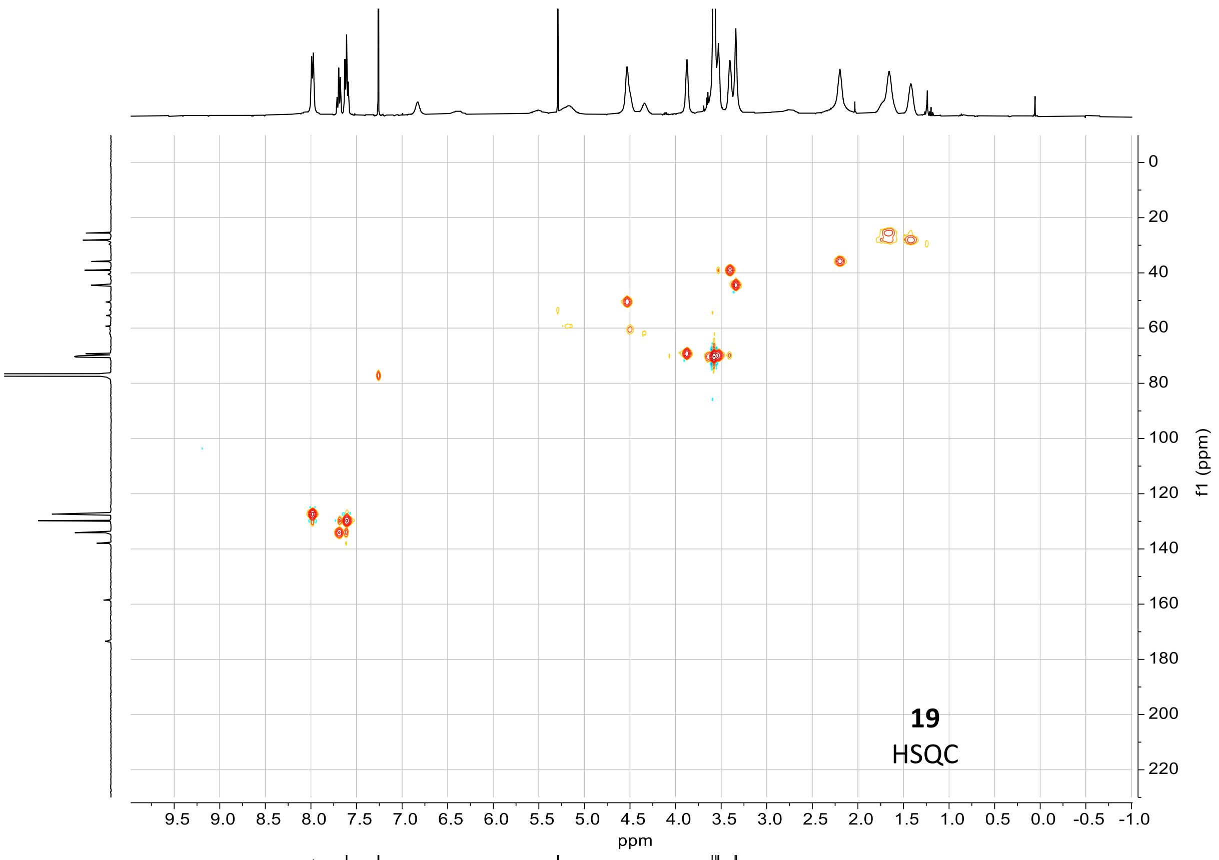
19
HSQC
ppm
f1 (ppm)

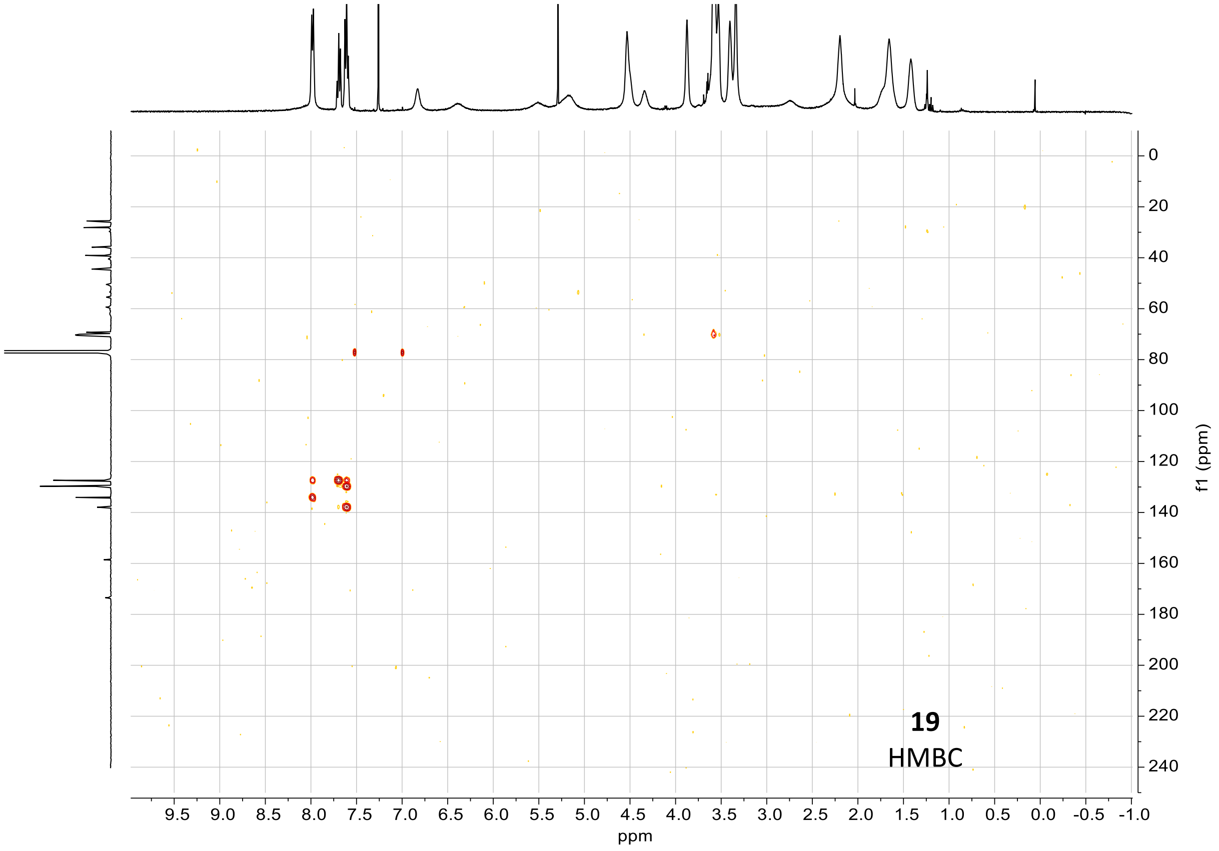
19
HMBC
ppm
f1 (ppm)

HPLC data of enantioenriched compound

Prop-2-yn-1-yl (S)-(methyl(oxo)(*p*-tolyl)-λ^6-sulfaneylidene)carbamate ((*S*)-2b)

Conditions: Chiralpak IB column, 90:10 *n*-hexane:*i*PrOH, flow rate: 1 mL min^{-1}, 35 °C, UV detection wavelength: 250 nm.

(*rac*)-2b

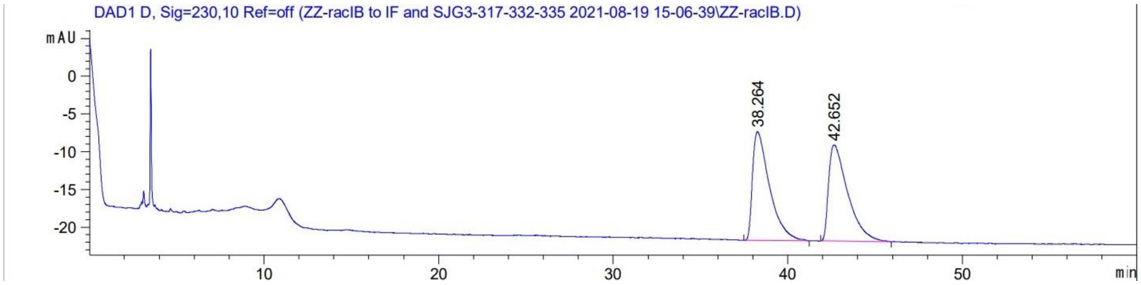

Signal 4: DAD1 D, Sig=230,10 Ref=off

Peak #	RetTime [min]	Type	Width [min]	Area [mAU*s]	Height [mAU]	Area %
1	38.264	BB	0.9377	977.59686	14.40676	50.1562
2	42.652	BB	1.0062	971.50824	12.76142	49.8438
Totals :				1949.10510	27.16817	

(*S*)-2b

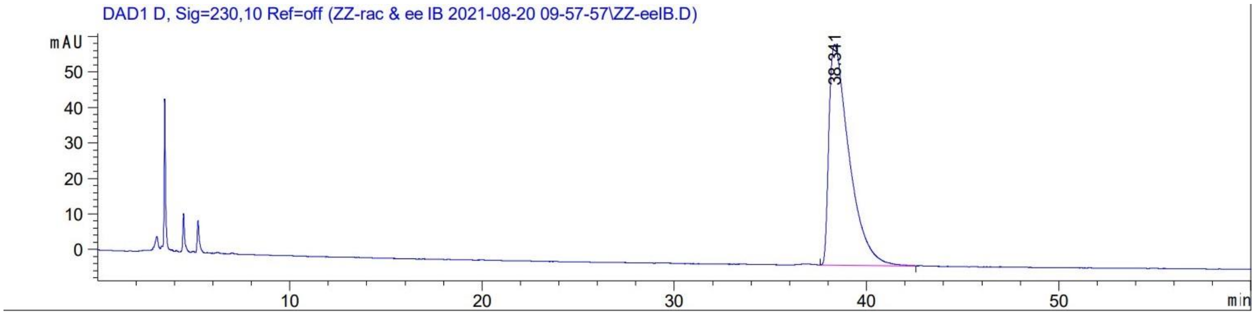

Signal 4: DAD1 D, Sig=230,10 Ref=off

Peak #	RetTime [min]	Type	Width [min]	Area [mAU*s]	Height [mAU]	Area %
1	38.341	BB	1.0550	4534.41064	62.16188	100.0000
Totals :				4534.41064	62.16188	

***ee* > 99%**

^{1}H NMR spectra of other starting materials

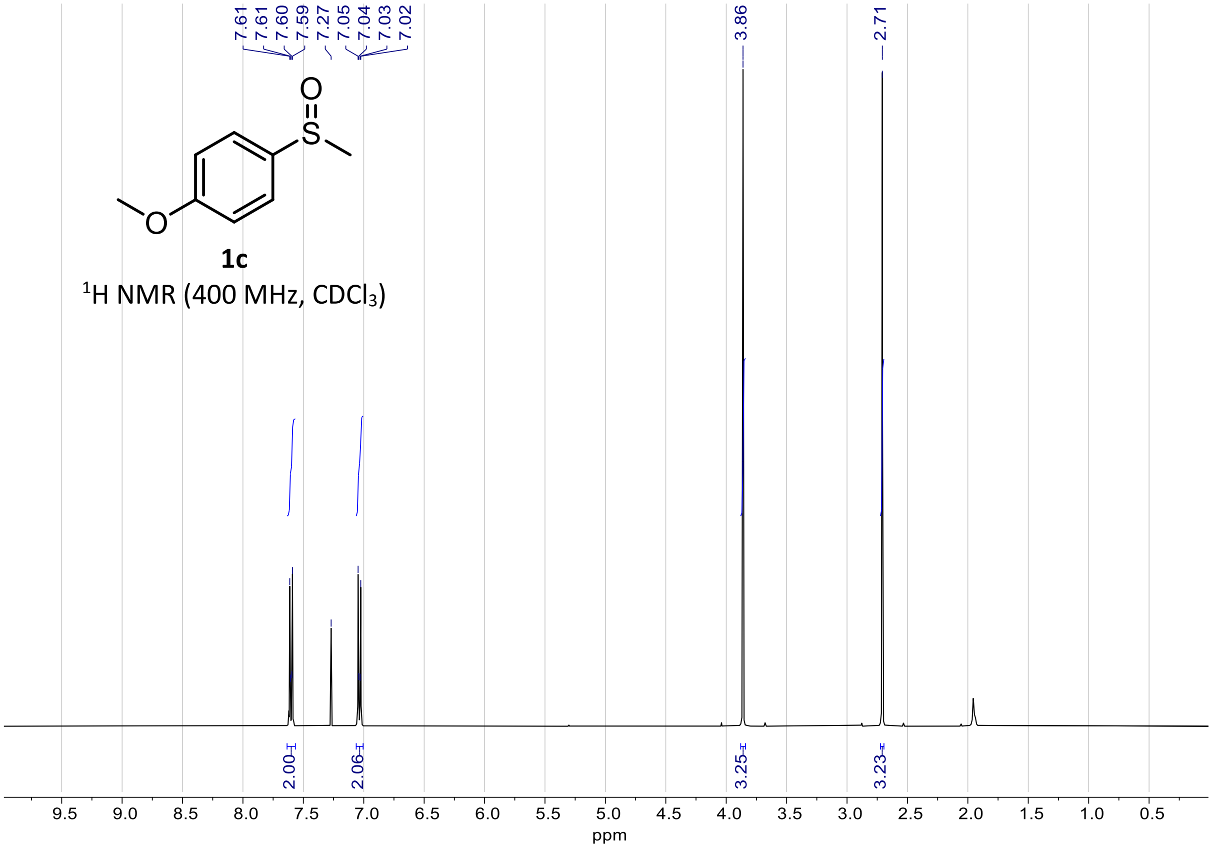
7.61
7.61
7.60
7.59
7.27
7.05
7.04
7.03
7.02
3.86
2.71
1c
^{1}H NMR (400 MHz, CDCl$_3$)
2.00
2.06
3.25
3.23
9.5
9.0
8.5
8.0
7.5
7.0
6.5
6.0
5.5
5.0
4.5
4.0
3.5
3.0
2.5
2.0
1.5
1.0
0.5
ppm

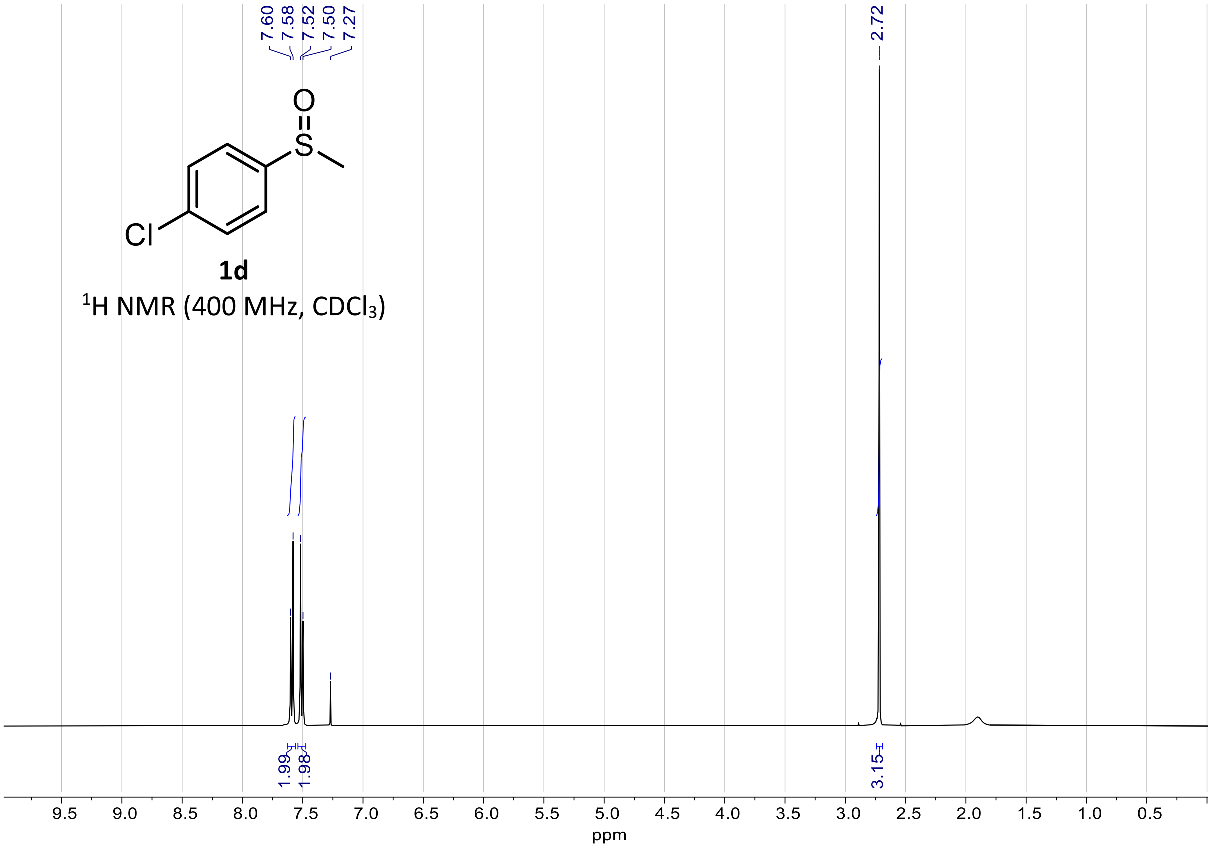
7.60
7.58
7.52
7.50
7.27
2.72
Cl
1d
^{1}H NMR (400 MHz, CDCl$_3$)
1.99
1.98
3.15
9.5
9.0
8.5
8.0
7.5
7.0
6.5
6.0
5.5
5.0
4.5
4.0
3.5
3.0
2.5
2.0
1.5
1.0
0.5
ppm

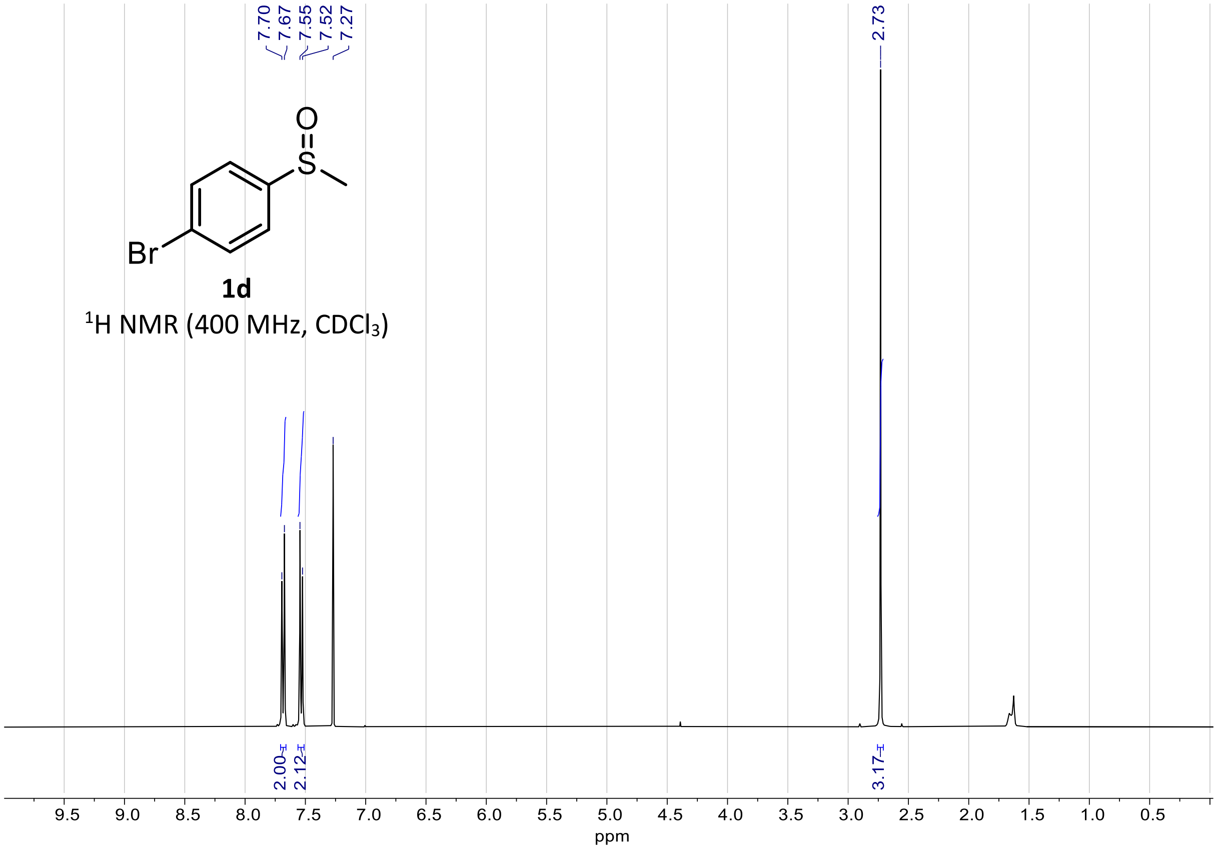

1d

^{1}H NMR (400 MHz, $CDCl_3$)

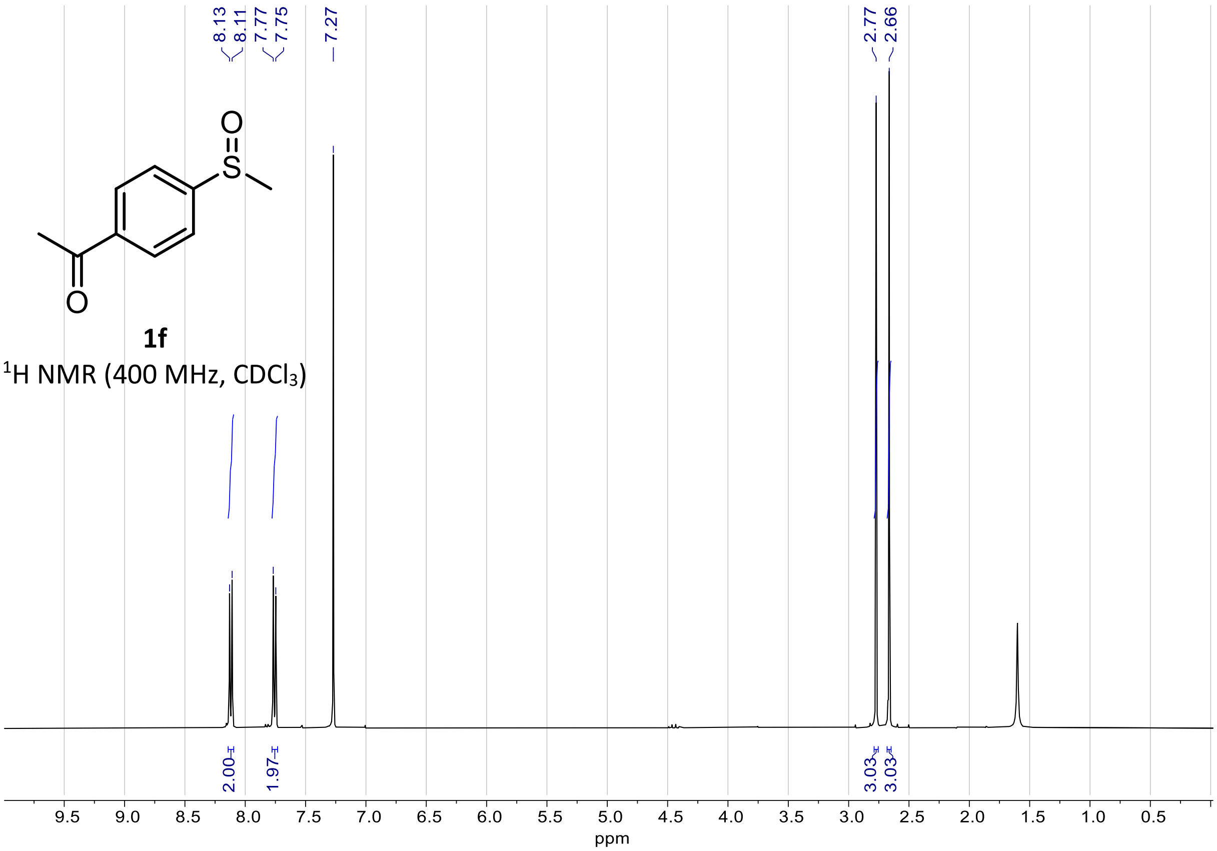

1f

^{1}H NMR (400 MHz, $CDCl_3$)

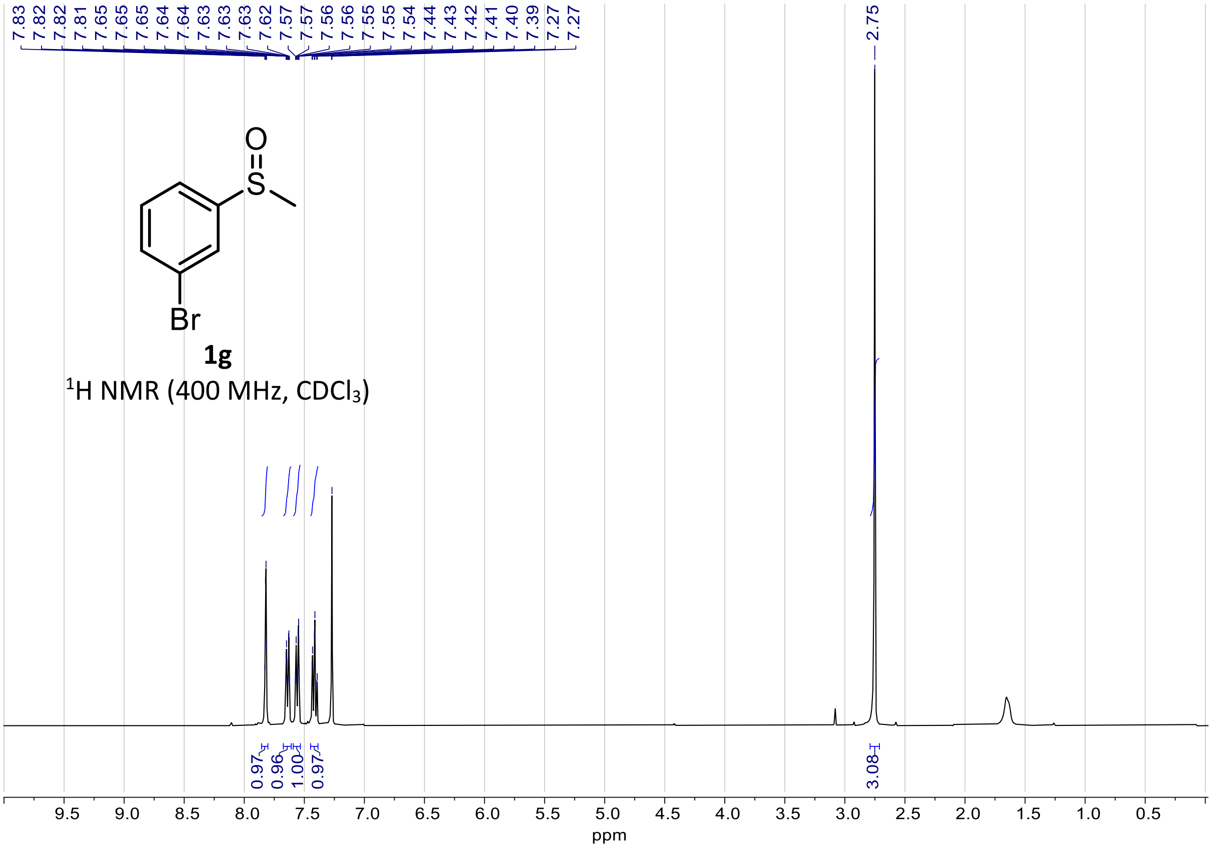

1g

^{1}H NMR (400 MHz, $CDCl_3$)

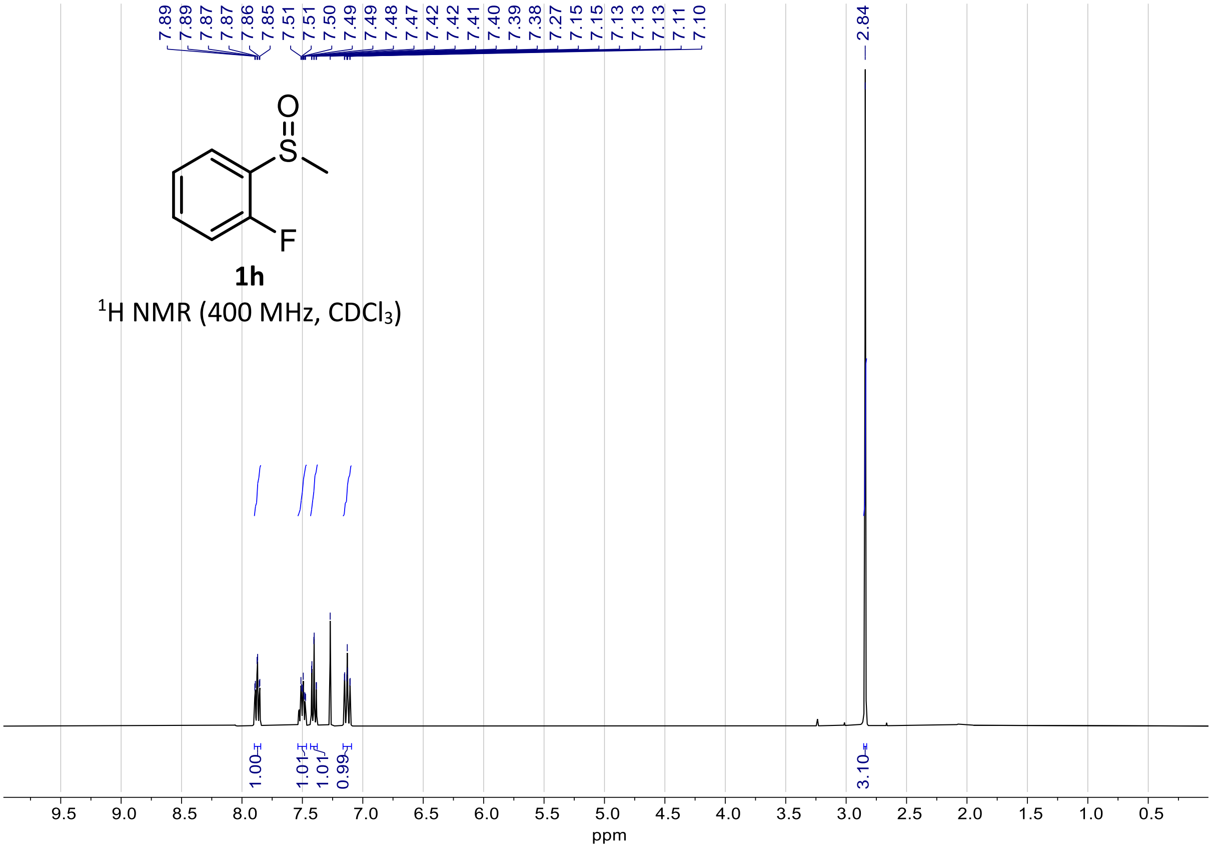

1h

^{1}H NMR (400 MHz, $CDCl_3$)

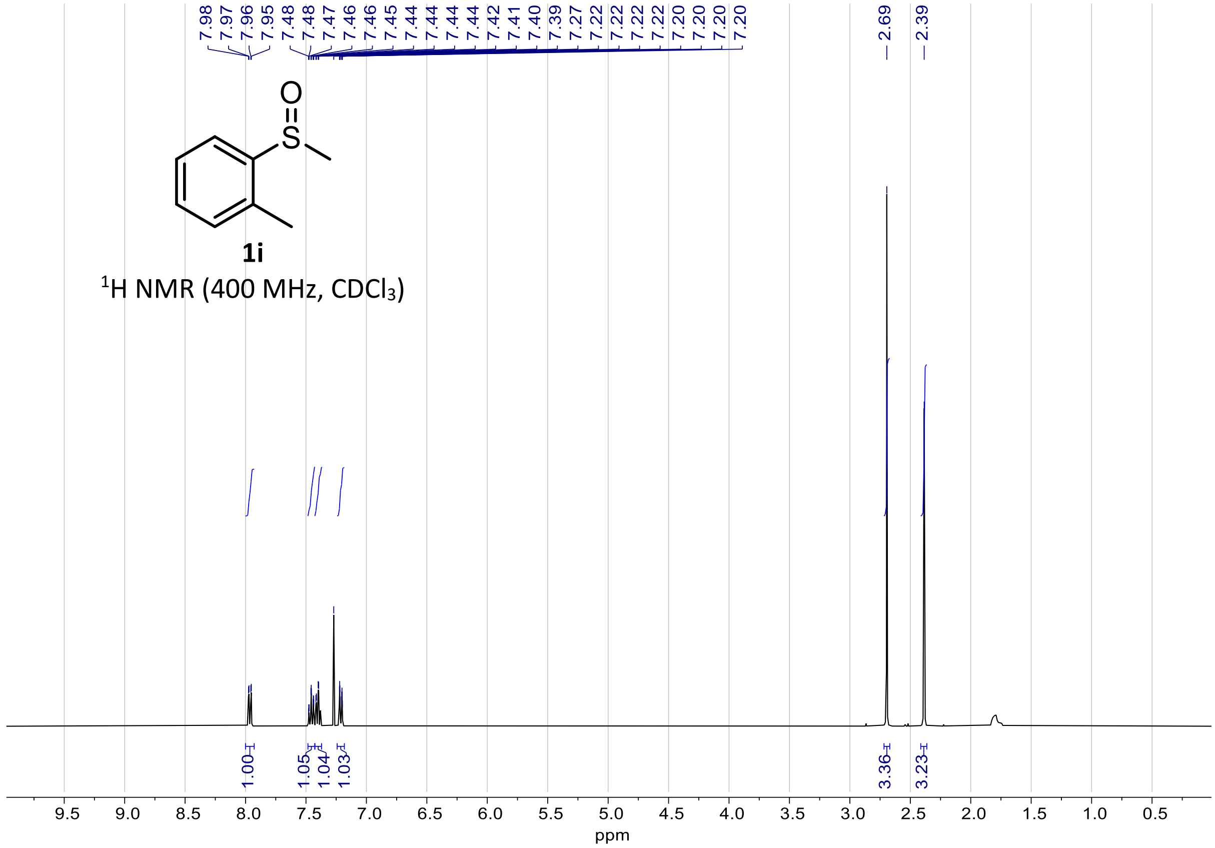
7.98 7.97 7.96 7.95 7.48 7.48 7.47 7.46 7.46 7.45 7.44 7.44 7.44 7.44 7.42 7.41 7.40 7.39 7.27 7.22 7.22 7.22 7.22 7.20 7.20 7.20 7.20
2.69
2.39
1i
^1H NMR (400 MHz, $CDCl_3$)
1.00
1.05
1.04
1.03
3.36
3.23
9.5 9.0 8.5 8.0 7.5 7.0 6.5 6.0 5.5 5.0 4.5 4.0 3.5 3.0 2.5 2.0 1.5 1.0 0.5
ppm

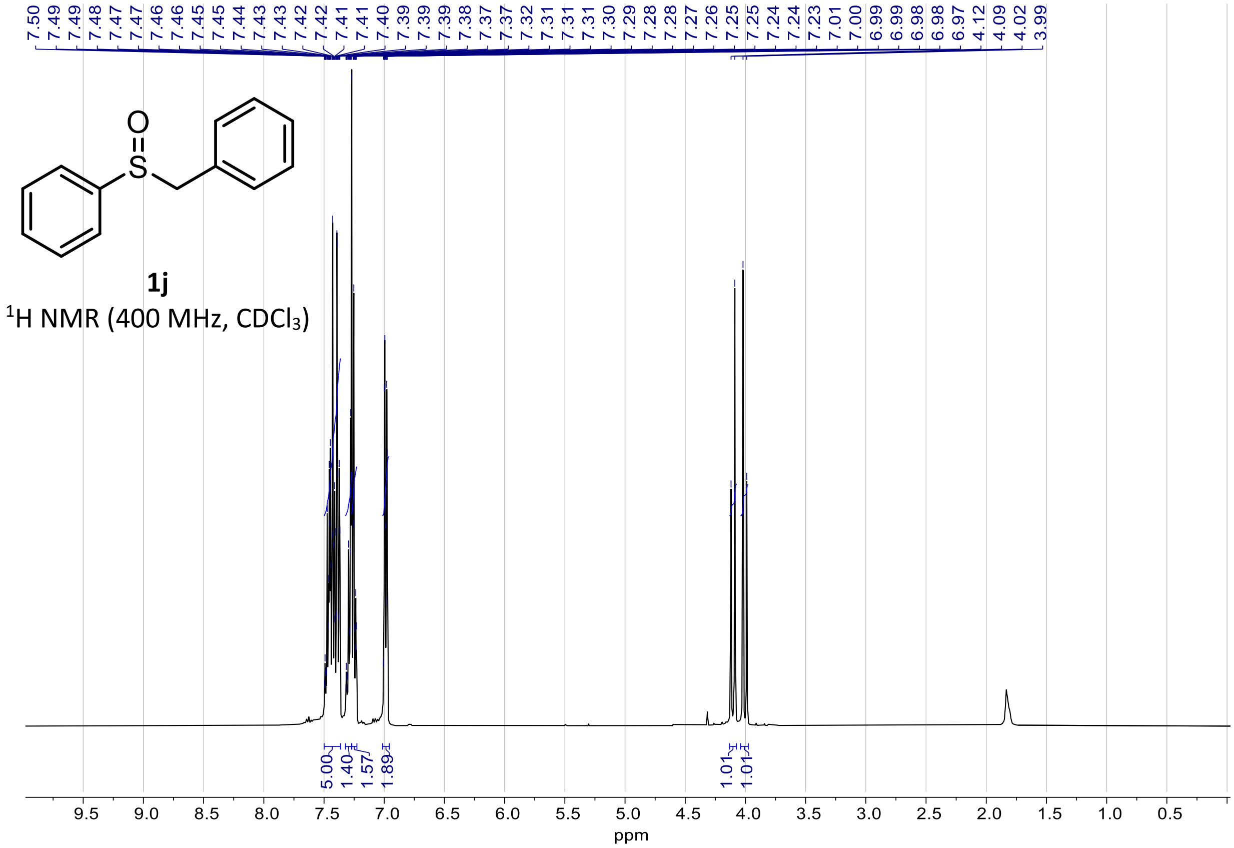
7.50 7.49 7.49 7.48 7.47 7.47 7.46 7.46 7.45 7.45 7.44 7.43 7.43 7.42 7.42 7.41 7.41 7.40 7.39 7.39 7.38 7.37 7.37 7.32 7.31 7.31 7.30 7.29 7.28 7.28 7.27 7.26 7.25 7.25 7.24 7.24 7.23 7.01 7.00 6.99 6.99 6.98 6.98 6.97 4.12 4.09 4.02 3.99
1j
^1H NMR (400 MHz, $CDCl_3$)
5.00
1.40
1.57
1.89
1.01
1.01
9.5 9.0 8.5 8.0 7.5 7.0 6.5 6.0 5.5 5.0 4.5 4.0 3.5 3.0 2.5 2.0 1.5 1.0 0.5
ppm

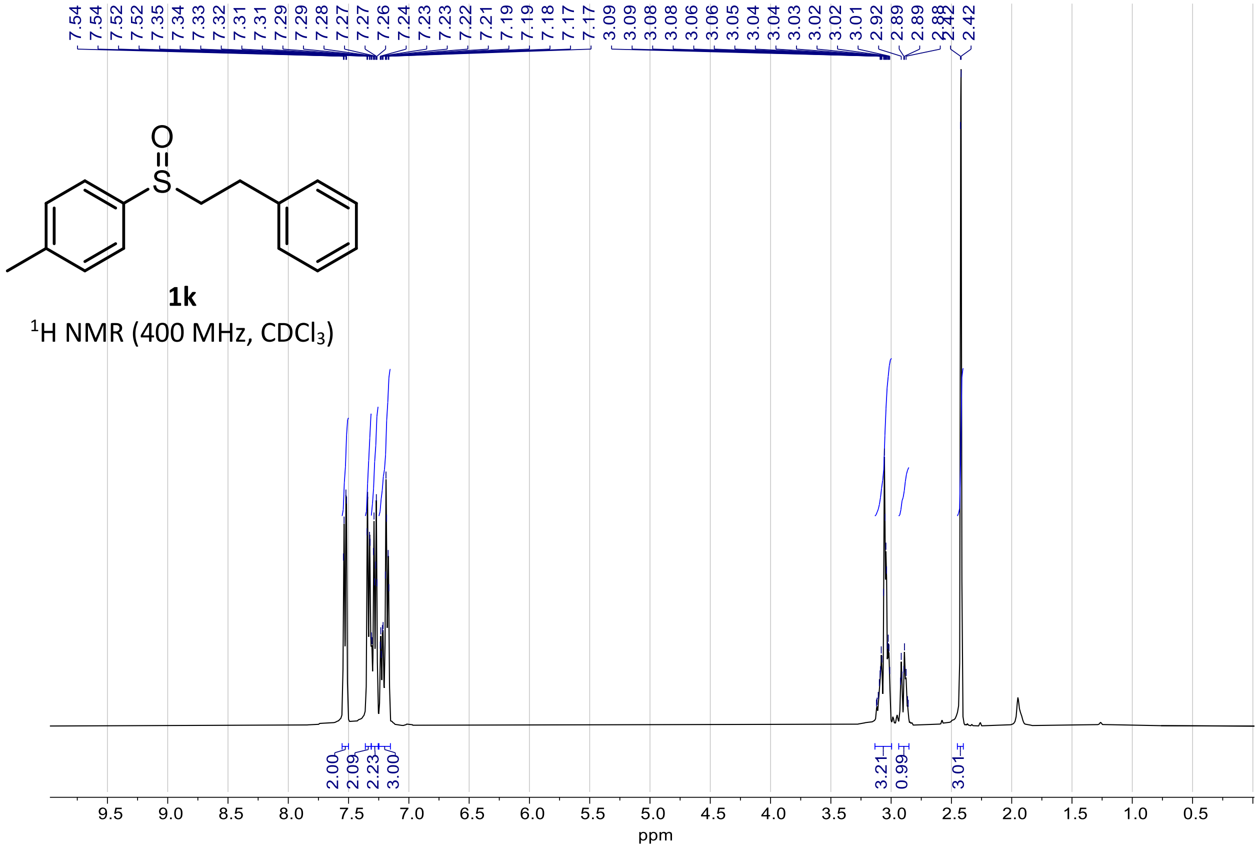
7.54 7.54 7.52 7.52 7.35 7.34 7.33 7.32 7.31 7.31 7.29 7.29 7.28 7.27 7.27 7.26 7.24 7.23 7.23 7.22 7.21 7.19 7.18 7.17 7.17 3.09 3.09 3.08 3.06 3.06 3.05 3.04 3.04 3.03 3.02 3.02 3.01 2.92 2.89 2.89 2.88 2.42 2.42
O
S
1k
^{1}H NMR (400 MHz, CDCl$_3$)
2.00
2.09
2.23
3.00
3.21
0.99
3.01
9.5 9.0 8.5 8.0 7.5 7.0 6.5 6.0 5.5 5.0 4.5 4.0 3.5 3.0 2.5 2.0 1.5 1.0 0.5
ppm

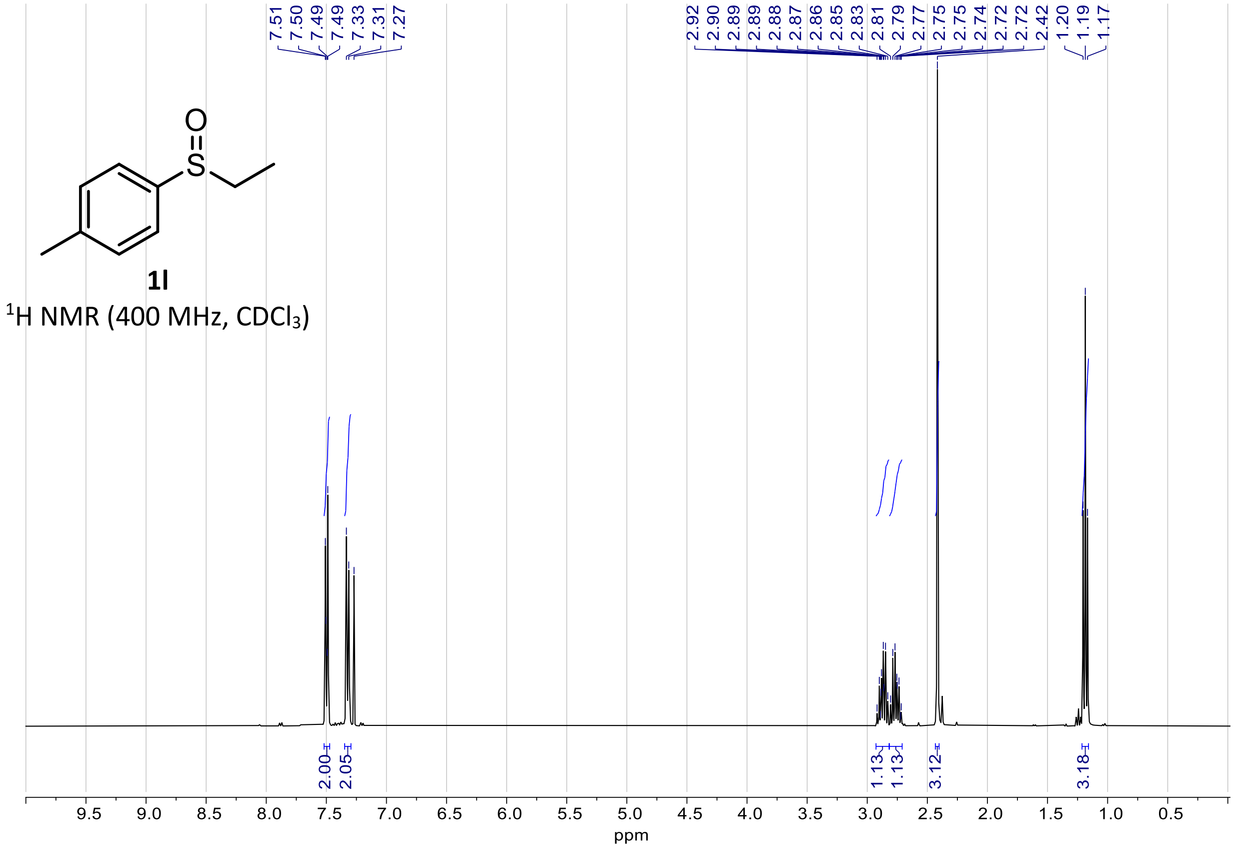
7.51 7.50 7.49 7.49 7.33 7.31 7.27 2.92 2.90 2.89 2.88 2.87 2.86 2.85 2.83 2.81 2.79 2.77 2.75 2.75 2.74 2.72 2.72 2.42 1.20 1.19 1.17
O
S
1l
^{1}H NMR (400 MHz, CDCl$_3$)
2.00
2.05
1.13
1.13
3.12
3.18
9.5 9.0 8.5 8.0 7.5 7.0 6.5 6.0 5.5 5.0 4.5 4.0 3.5 3.0 2.5 2.0 1.5 1.0 0.5
ppm

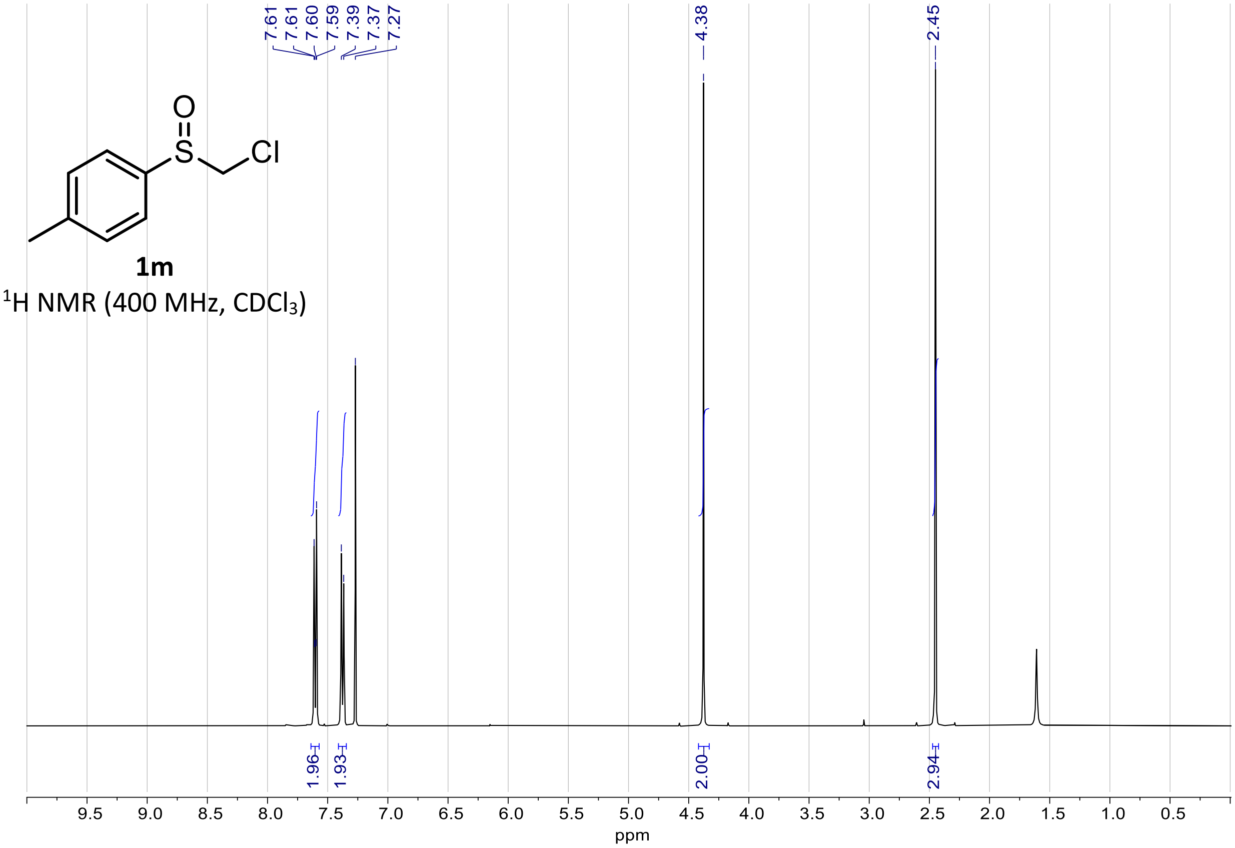

1m
1H NMR (400 MHz, CDCl3)
7.61
7.61
7.60
7.59
7.39
7.37
7.27
4.38
2.45
1.96
1.93
2.00
2.94
ppm

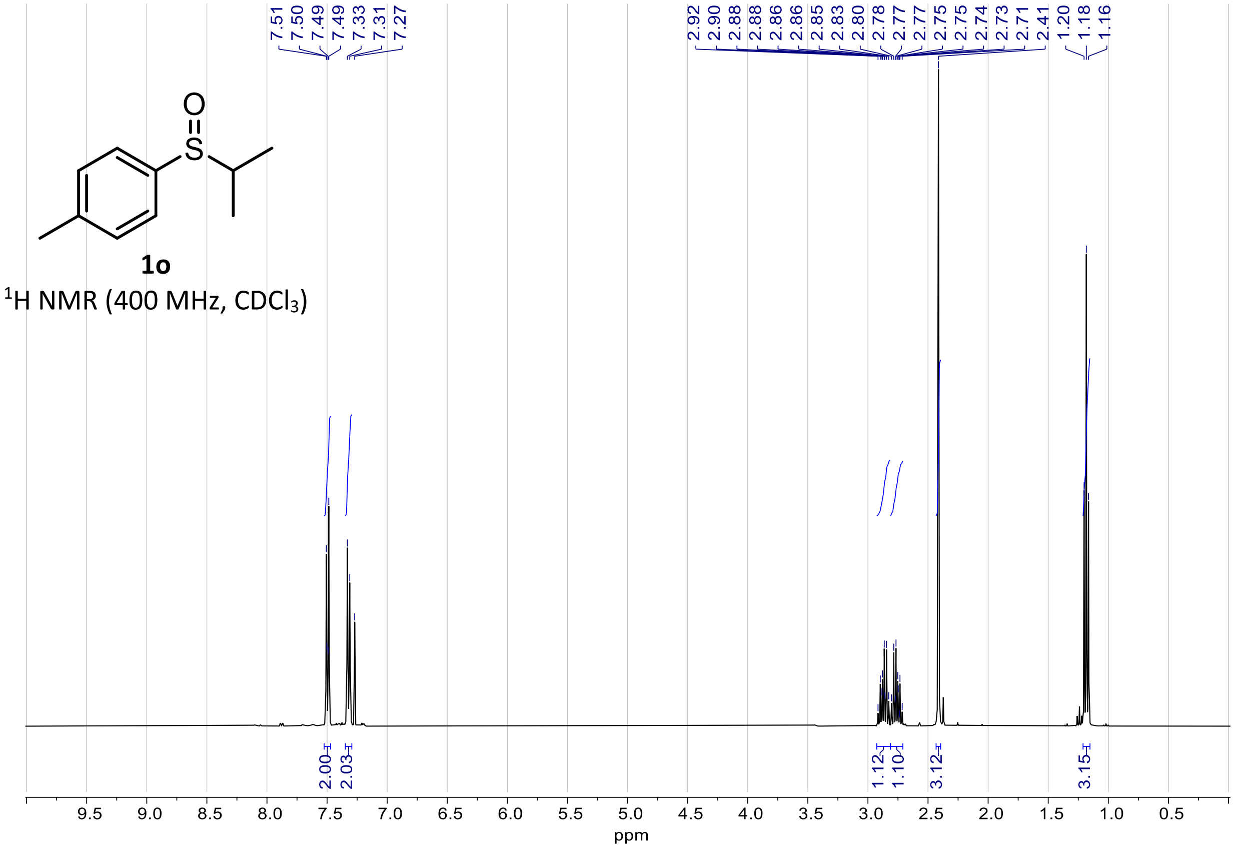

1o
1H NMR (400 MHz, CDCl3)
7.51
7.50
7.49
7.49
7.33
7.31
7.27
2.92
2.90
2.88
2.86
2.86
2.85
2.83
2.80
2.78
2.77
2.77
2.75
2.75
2.74
2.73
2.71
2.41
1.20
1.18
1.16
2.00
2.03
1.12
1.10
3.12
3.15
ppm

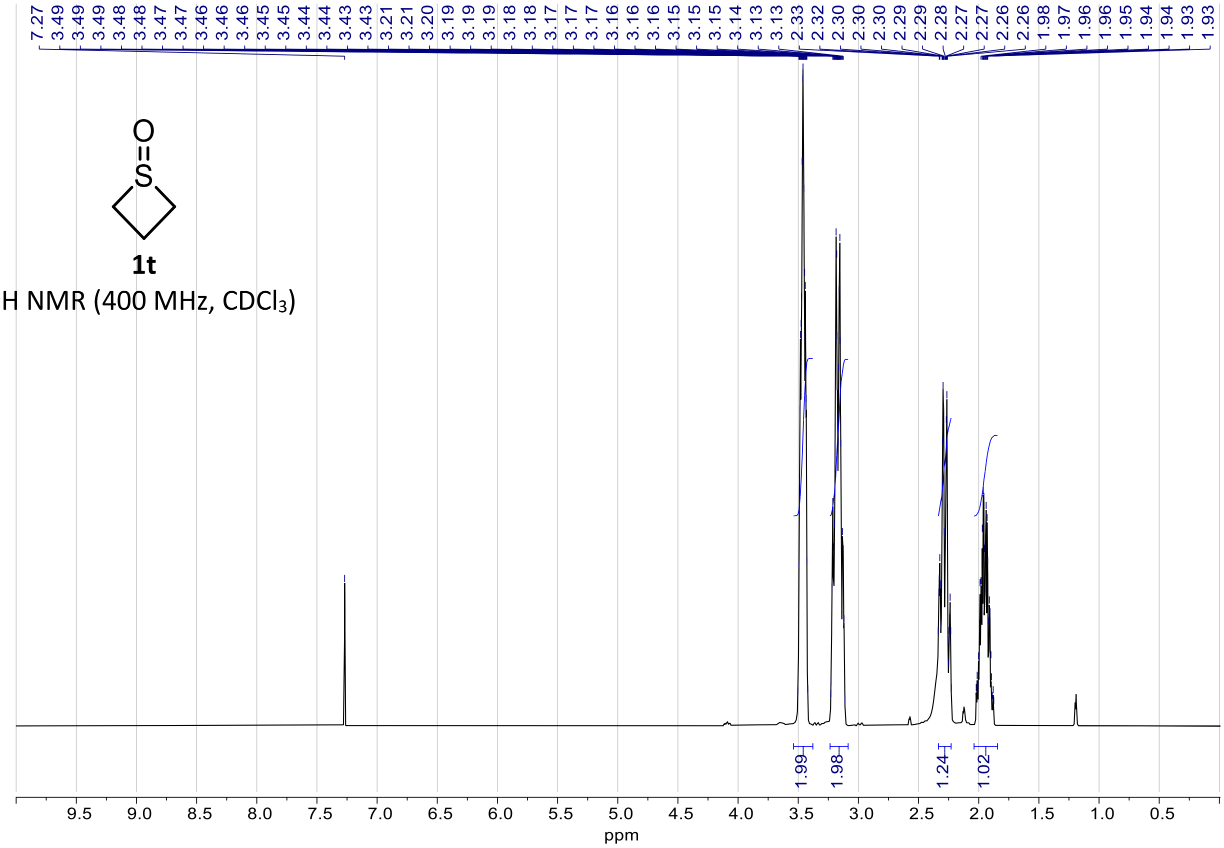

1t

^{1}H NMR (400 MHz, $CDCl_3$)

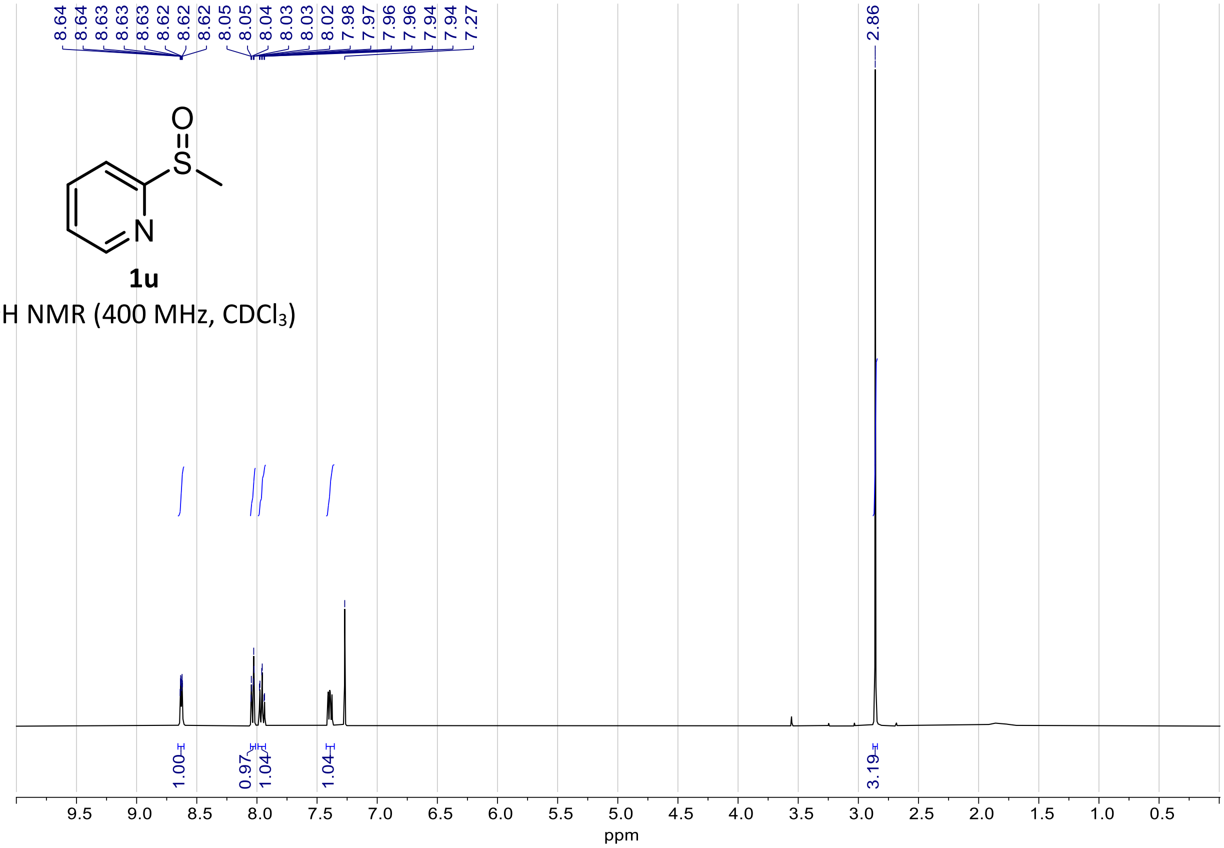

1u

^{1}H NMR (400 MHz, $CDCl_3$)

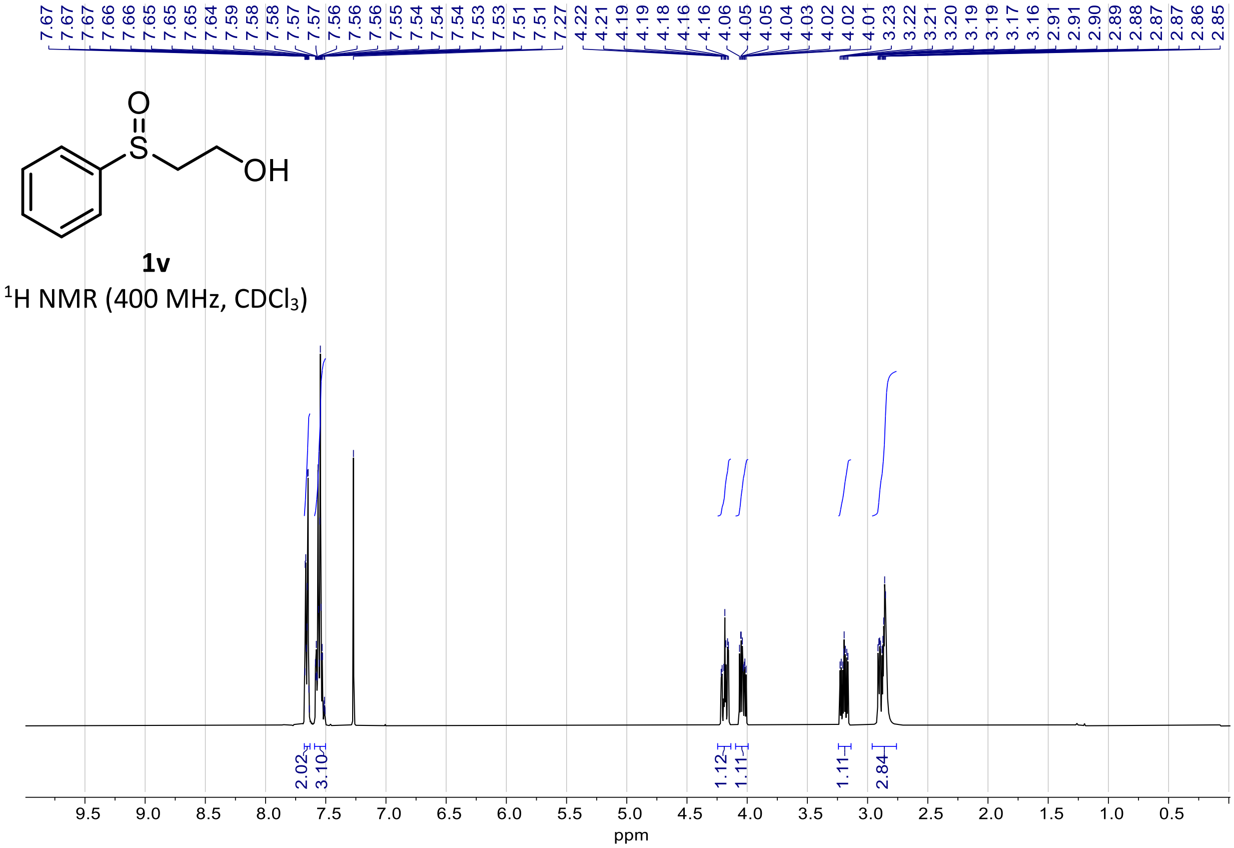
O
S
OH
1v
1H NMR (400 MHz, CDCl3)
2.02
3.10
1.12
1.11
1.11
2.84
ppm

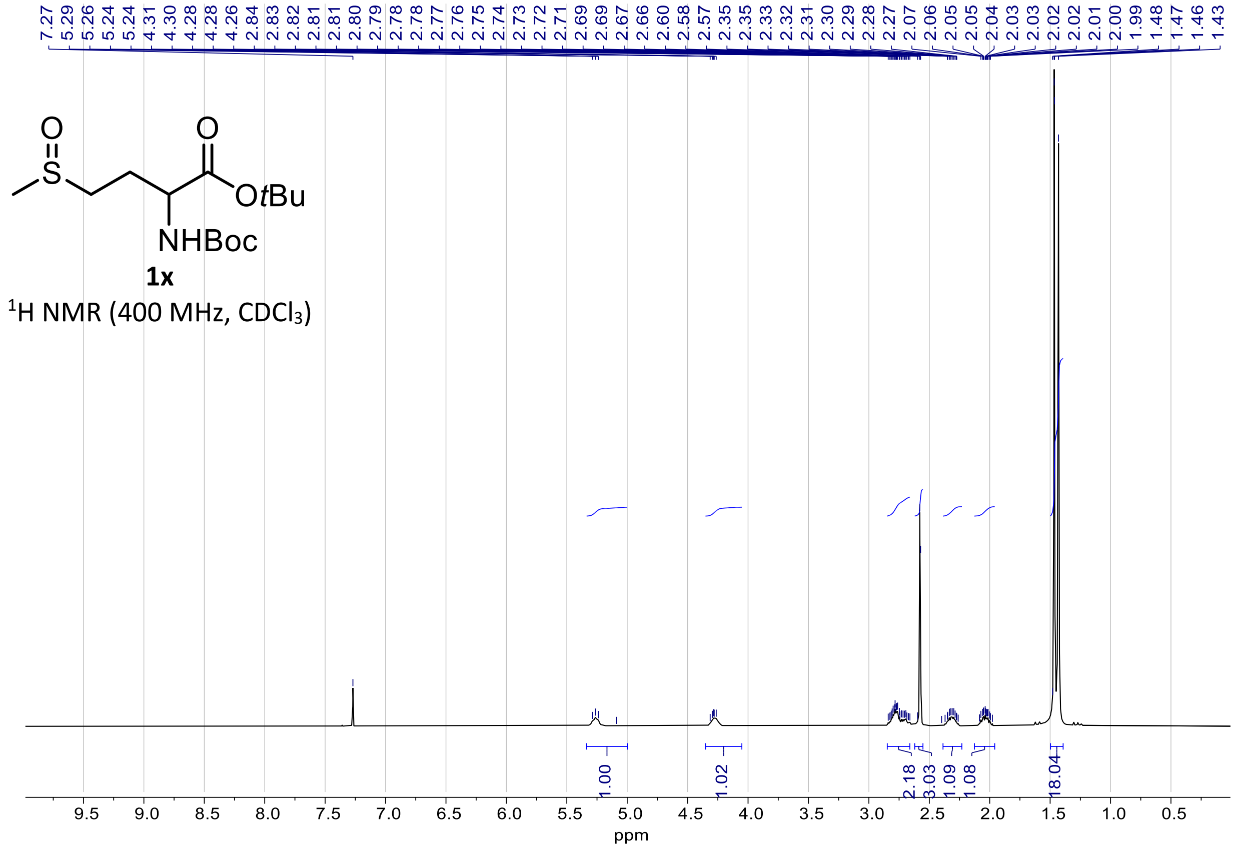
O
S
O
OtBu
NHBoc
1x
1H NMR (400 MHz, CDCl3)
1.00
1.02
2.18
3.03
1.09
1.08
18.04
ppm

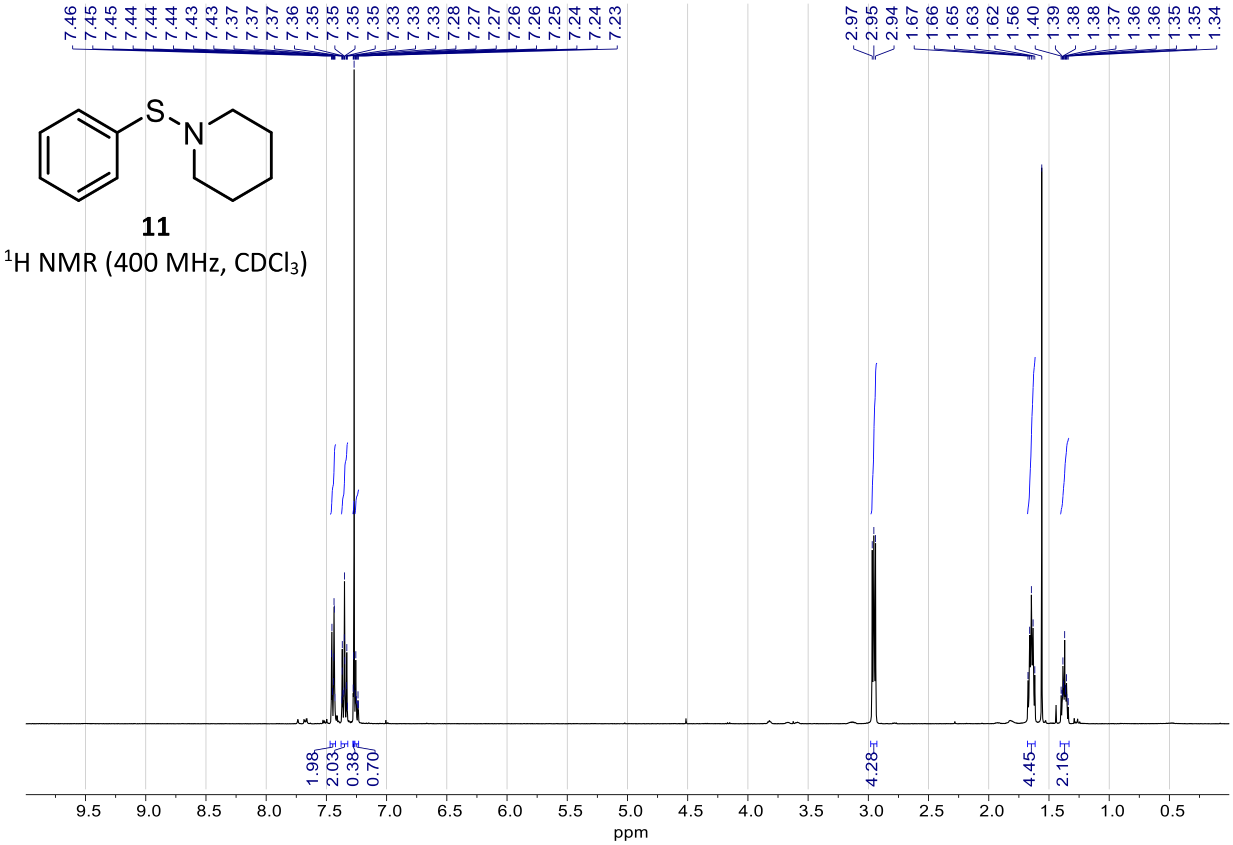
S
N
11
1H NMR (400 MHz, CDCl3)
9.5 9.0 8.5 8.0 7.5 7.0 6.5 6.0 5.5 5.0 4.5 4.0 3.5 3.0 2.5 2.0 1.5 1.0 0.5
ppm

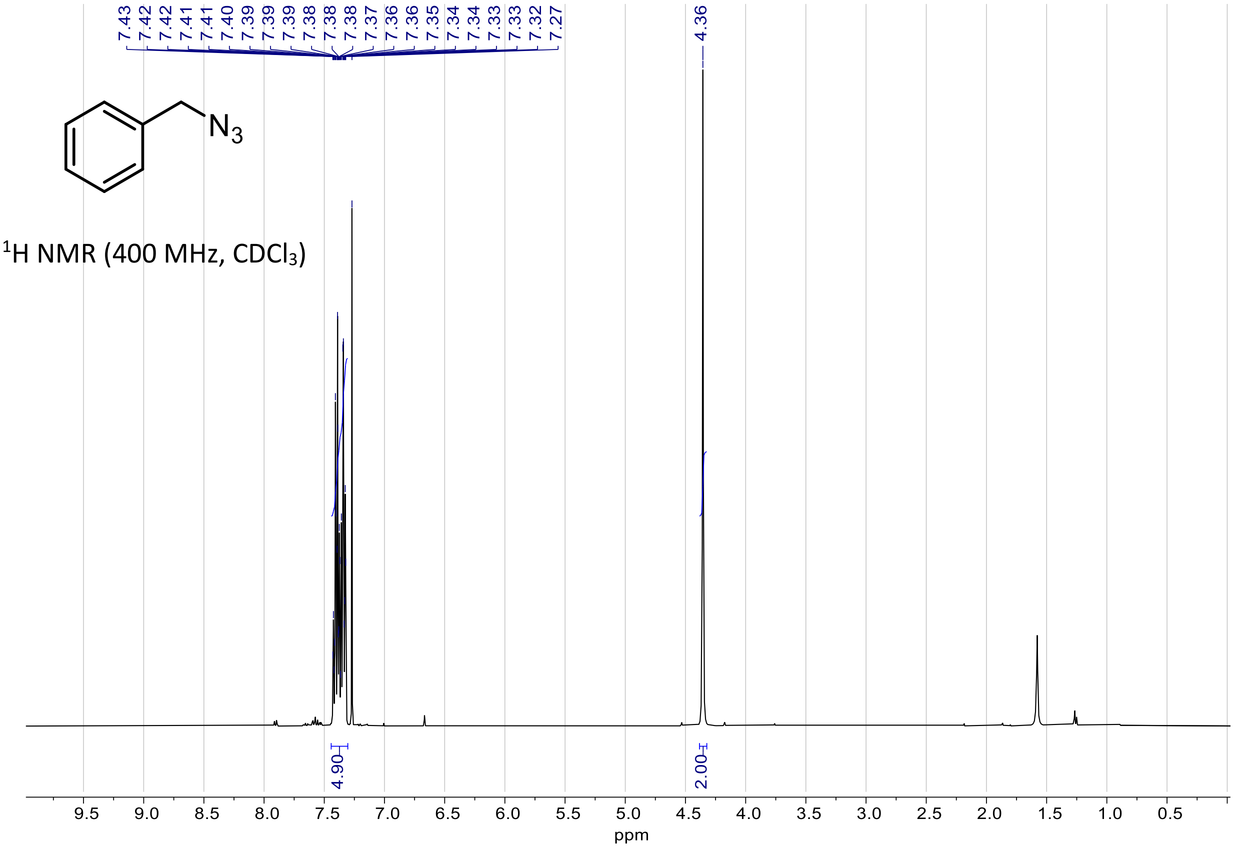
N3
1H NMR (400 MHz, CDCl3)
9.5 9.0 8.5 8.0 7.5 7.0 6.5 6.0 5.5 5.0 4.5 4.0 3.5 3.0 2.5 2.0 1.5 1.0 0.5
ppm
